# Forming cytoplasmic stress granules PURα suppresses mRNA translation initiation of IGFBP3 to promote esophageal squamous cell carcinoma progression

**DOI:** 10.1038/s41388-022-02426-3

**Published:** 2022-08-09

**Authors:** Lusong Tian, Xiufeng Xie, Urmi Das, Yuling Chen, Yulin Sun, Fang Liu, Haizhen Lu, Peng Nan, Ying Zhu, Xinglu Gu, Haiteng Deng, Jiuyong Xie, Xiaohang Zhao

**Affiliations:** 1 State Key Laboratory of Molecular Oncology, National Cancer Center/National Clinical Research Center for Cancer/Cancer Hospital, Chinese Academy of Medical Sciences and Peking Union Medical College, Beijing, 100021 China; 2 Department of Physiology & Pathophysiology, Max Rady College of Medicine, Rady Faculty of Health Sciences, University of Manitoba, Winnipeg, MB Canada; 3 MOE Key Laboratory of Bioinformatics, Center for Synthetic and Systematic Biology, School of Life Sciences, Tsinghua University, Beijing, 100084 China; 4 Department of Pathology, National Cancer Center/Cancer Hospital, Chinese Academy of Medical Sciences and Peking Union Medical College, Beijing, 100021 China

**Keywords:** Oncogenes, Oesophageal cancer, Translation

## Abstract

Esophageal squamous cell carcinoma (ESCC) is one of the most fatal malignancies worldwide. Recently, our group identified purine-rich element binding protein alpha (PURα), a single-stranded DNA/RNA-binding protein, to be significantly associated with the progression of ESCC. Additional immunofluorescence staining demonstrated that PURα forms cytoplasmic stress granules to suppress mRNA translation initiation. The expression level of cytoplasmic PURα in ESCC tumor tissues was significantly higher than that in adjacent epithelia and correlated with a worse patient survival rate by immunohistochemistry. Functionally, PURα strongly preferred to bind to UG-/U-rich motifs and mRNA 3´UTR by CLIP-seq analysis. Moreover, PURα knockout significantly increased the protein level of insulin-like growth factor binding protein 3 (IGFBP3). In addition, it was further demonstrated that PURα-interacting proteins are remarkably associated with translation initiation factors and ribosome-related proteins and that PURα regulates protein expression by interacting with translation initiation factors, such as PABPC1, eIF3B and eIF3F, in an RNA-independent manner, while the interaction with ribosome-related proteins is significantly dependent on RNA. Specifically, PURα was shown to interact with the mRNA 3´UTR of IGFBP3 and inhibit its expression by suppressing mRNA translation initiation. Together, this study identifies cytoplasmic PURα as a modulator of IGFBP3, which could be a promising therapeutic target for ESCC treatment.

## Introduction

Esophageal squamous cell carcinoma (ESCC), with a 5-year survival rate of approximately 15% to 20%, is one of the most lethal gastrointestinal malignancies worldwide [1–3]. Although some targeted anticancer drugs, such as gefitinib and PD-L1 blockers, have been adopted in the clinic [4], the mortality rate of ESCC is still relatively high owing to invasion and distant metastasis. Large-scale whole-exome sequencing (WES) of ESCC has identified some high-frequency gene mutations, including copy number alterations and somatic mutations [5–7]. In addition, epigenetic alterations in ESCC, such as DNA methylation and histone acetylation, have also been partially characterized [8]. Despite great advances in the genomic and epigenetic aspects of ESCC, the underlying mechanisms of tumor progression remain poorly understood. Hence, it is imperative to further investigate the mechanisms.

Purine-rich element binding protein alpha (PURα), encoded by *PURΑ*, is a single-stranded DNA/RNA-binding protein that is highly conserved from bacteria to humans [9]. *PURΑ* knockout in mice and *PURΑ* mutation in humans both result in severe neurological disease [10–14]. Abnormal PURα expression is also involved in the progression of several cancers, such as acute myeloid leukemia (AML) and prostate cancer [15, 16], and our previous results have shown that overexpression of PURα promotes ESCC progression [17]. Thus far, PURα has been implicated primarily in DNA replication, transcription and the cell cycle [18–23]. Recent reports have indicated that PURα in the cytoplasm encapsulates specific RNAs with some RNA-binding proteins to regulate mRNA transport [24–28], and emerging evidence suggests that PURα is a novel component of cytoplasmic stress granules [25, 29–31]. Stress granules are cytoplasmic RNA–protein complexes that form when translation initiation is limited [32, 33] and have been proposed to play an important role in neurodegenerative diseases and tumor progression [34, 35]. For example, PURα colocalized with mutant FUS in stress granules to modulate amyotrophic lateral sclerosis (ALS) pathology [25, 31]. In addition, PURα is known to associate with noncoding RNAs (e.g., TAR RNA [36], BC200 [26] and circSamd4 [37]). PURα strongly influences the development and progression of disease by regulating DNA replication, transcription and mRNA transport, but whether PURα participates in tumor progression by regulating mRNA-based processes remains unclear.

Here, we found that PURα participates in the formation of cytoplasmic stress granules and that the expression level of cytoplasmic PURα was significantly increased in ESCC tissues compared to nontumorous tissues and that ESCC patients with high expression levels of cytoplasmic PURα had a lower survival rate than those with low expression levels. We further revealed that PURα repressed the mRNA translation initiation of insulin-like growth factor binding protein 3 (IGFBP3) by forming cytoplasmic stress granules. In addition, knockdown of IGFBP3 significantly reversed the inhibitory effects of PURα loss on the cell proliferation, migration and invasion properties of KYSE170 ESCC cells. In brief, our results support that cytoplasmic PURα mediates ESCC progression by binding to the mRNA 3´UTR.

## Results

### Cytoplasmic PURα participates in the formation of stress granules and significantly correlates with ESCC progression

**Fig. 1:**
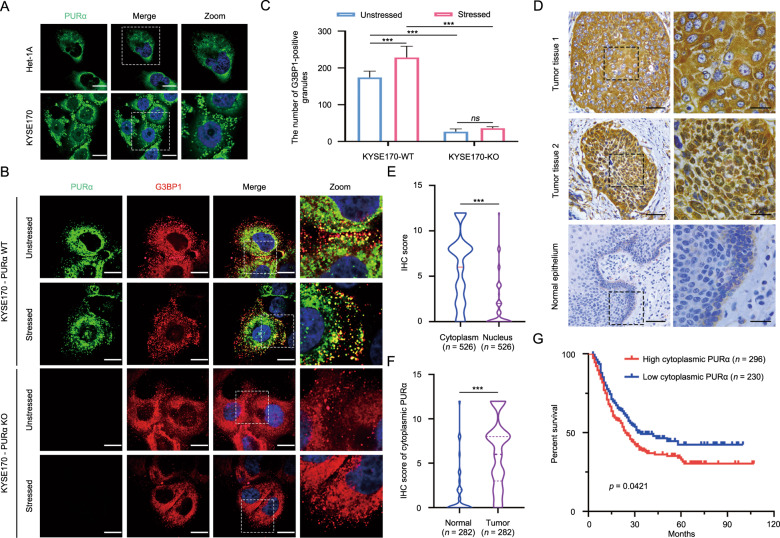
Cytoplasmic PURα participates in the formation of stress granules and significantly correlates with ESCC progression. **A** The localization of endogenous PURα in esophageal epithelium Het-1A and ESCC cancer (KYSE170) cells was visualized by immunofluorescence assay. PURα proteins dispersed in the cytoplasm as granules in KYSE170 cells or accumulated around the nucleus in Het1A cells. Scale bars: 30 μm. **B** The colocalization between endogenous PURα and the stress granule maker G3BP1 was visualized in wild-type (WT) and PURα-deficient KYSE170 (KO) cells by immunofluorescence staining. Scale bars: 30 μm. **C** The number of PURα/G3BP1-positive granules under stress conditions or not was calculated separately. ***p < 0.001; *ns*, not significant. **D** PURα expression in ESCC tumor tissues (first and second panels) and adjacent nontumor epithelia (third panel) was compared by immunohistochemical staining (IHC). PURα protein mainly located in cytoplasm (first panel) or nucleus (second panel) is shown. The representative region (black frame) at low magnification (40×, left) was amplified at high magnification (100×, right). Scale bars: 50 μm. **E** Violin plots of the statistical data regarding the IHC score of PURα protein in the cytoplasm and nucleus of tumor tissues (*n* = 526). ***p < 0.001 by Mann–Whitney test. **F** Violin plots of the statistical data regarding the IHC scores for cytoplasmic PURα in ESCC (tumor) and adjacent nontumor (normal) tissues (*n* = 282) were drawn. ***p < 0.001 by Mann–Whitney. **G** Kaplan–Meier analyses of overall survival. Patients with high cytoplasmic PURα expression (*n* = 296) had a significantly lower overall survival rate than patients with low cytoplasmic PURα expression (*n* = 230).

It has been commonly reported that PURα is involved in the progression of several cancers as a transcription factor [9, 18, 23]. Intriguingly, immunofluorescence staining indicated that there was the considerable cytoplasmic localization of PURα in ESCC cells and that cytoplasmic PURα was evenly dispersed as granules or accumulated around the nucleus in nongranules (Fig. 1A). Increasing evidence has reported that PURα is a core component of cytoplasmic stress granules [25, 29–31], suggesting that cytoplasmic PURα-positive granules in ESCC cells are likely a form of stress granules. To this end, the colocalization between PURα-positive granules and G3BP1, a well-known cytoplasmic stress granule maker [32, 33, 38], was further detected by immunofluorescence staining. It was observed that G3BP1 is localized in PURα-positive granules and that the number of PURα/G3BP1-positive granules under stress conditions markedly increased compared with those under native conditions, while the number of G3BP1-positive granules markedly decreased after the loss of PURα (Fig. 1B, C), demonstrating that cytoplasmic PURα in ESCC cells participates in the formation of stress granules. In addition, we also observed that there was more expression of PURα in the cytoplasm than in the nucleus in the ESCC tissues by immunohistochemical staining (*n* = 526) (Fig. 1D, E), and the protein fractionation analysis also indicated that PURα was additionally localized in the cytoplasm to a much greater extent than in the nucleus of ESCC cells (Fig. S1A). Furthermore, we compared the expression of cytoplasmic PURα in ESCC tissues (*n* = 282) and adjacent nontumorous epithelia (*n* = 282) and observed that cytoplasmic PURα expression was significantly increased in ESCC tissues (Fig. 1D, F). Importantly, Kaplan–Meier survival analysis of a total of 526 ESCC patients showed that ESCC patients with high expression levels of cytoplasmic PURα had a lower survival rate than those with low PURα (Fig. 1G), implying that cytoplasmic PURα is linked to ESCC progression. There were no correlations between PURα levels and sex, age, tumor differentiation or other factors (Supplementary Table S1).

### Extensive RNA targets of PURα in ESCC cells were revealed by CLIP-seq analysis

**Fig. 2:**
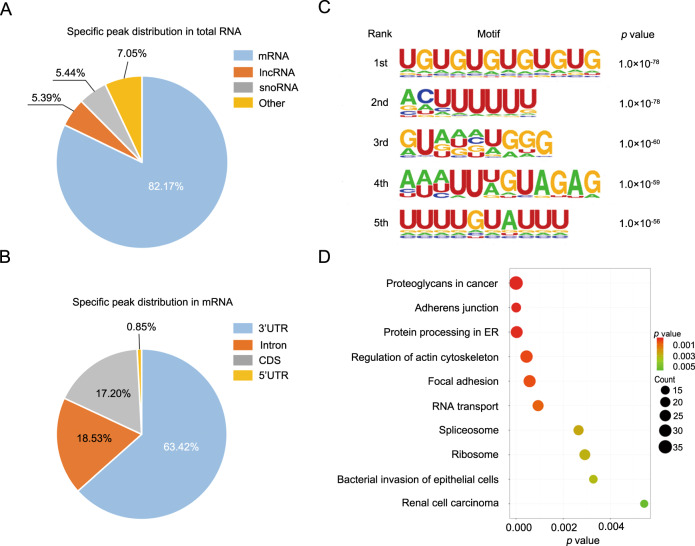
Extensive RNA targets of PURα in ESCC cells were revealed by CLIP-seq analysis. **A** The distribution of 2299 specific peaks bound by PURα in total RNA is shown in the pie diagram. **B** The distribution of 1889 specific peaks bound by PURα in mRNA is shown in the pie diagram. **C** Motif enrichment analysis was performed with the HOMER algorithm to determine the PURα binding sequence preference. The top 5 motifs are shown. **D** GO analysis was implemented with DAVID based on PURα-bound mRNA. The top 10 enriched pathways are shown.

Cytoplasmic stress granules are ribonucleoprotein granules and are involved in the regulation of RNA homeostasis [32, 33], so we speculated that cytoplasmic PURα likely modulates ESCC progression through interaction with mRNA. Cytoplasmic PURα notably affects brain development in humans and mice as an RNA-binding protein, but the characteristics of RNA bound to PURα have not been fully elucidated [10–14, 24, 26–28]. We thus first identified RNA targets of human PURα in ESCC KYSE510 cells by CLIP-seq analysis according to reported methods [39]. In total, high-throughput sequencing yielded ~38.5 and ~34.4 million raw reads from two independent replicated PURα CLIP-seq datasets (Supplementary Table S2). After discarding low-quality raw reads and normalization, ~17.0 and ~15.2 million clean reads were generated, respectively. Of these clean reads, 82.27% (~13.9 million) and 83.52% (~12.7 million) were unambiguously mapped to the human reference genome (hg38) (Supplementary Table S3). The vast majority of uniquely mapped reads (62.98% and 57.48%) mapped to introns. Then, PURα-binding sites were predicted with a peak calling algorithm as previously reported [40], and we ascertained that 5848- and 6367-specific peaks bound to PURα but not IgG (Fig. S1B). These specific peaks were matched to 3602 and 3813 genes, respectively. Among them, 2299 specific peaks coexisted in both PURα CLIP-seq datasets. Of the coexisting peaks, 1889 mapped to mRNAs (82.17%), 125 mapped to snoRNAs (5.44%) and 123 mapped to lncRNAs (5.39%) (Fig. 2A; Supplementary Table S4). Interestingly, most binding sites in mRNAs were enriched in the 3′UTRs (63.34%, Fig. 2B, Supplementary Table S4). To determine the PURα binding sequence preference, we analyzed the motif characteristics with the HOMER algorithm and found that PURα preferentially binds UG-/U-rich motifs (Fig. 2C). We also carried out an independent analysis using PIPE-CLIP [41], identifying the core binding sites as UUU (*E*-value < 1e-170, Fig. S1C) and UG motifs (*E*-value < 1e-11, Fig. S1B) based on the crosslinking-induced mutations (deletion or substitutions). To further identify the potential cellular functions that PURα regulates by binding to mRNA, we carried out Gene Ontology (GO) analysis using the database for annotation, visualization, and integrated discovery (DAVID). The PURα-binding genes were mainly associated with the regulation of cell adherens junctions and mRNA metabolism (Fig. 2D). In conclusion, PURα directly binds mRNA with a strong preference for 3’UTRs, and the PURα-binding sequence is enriched in UG-/U-rich motifs.

### PURα inhibits IGFBP3 protein expression by binding to its 3’UTR

**Fig. 3:**
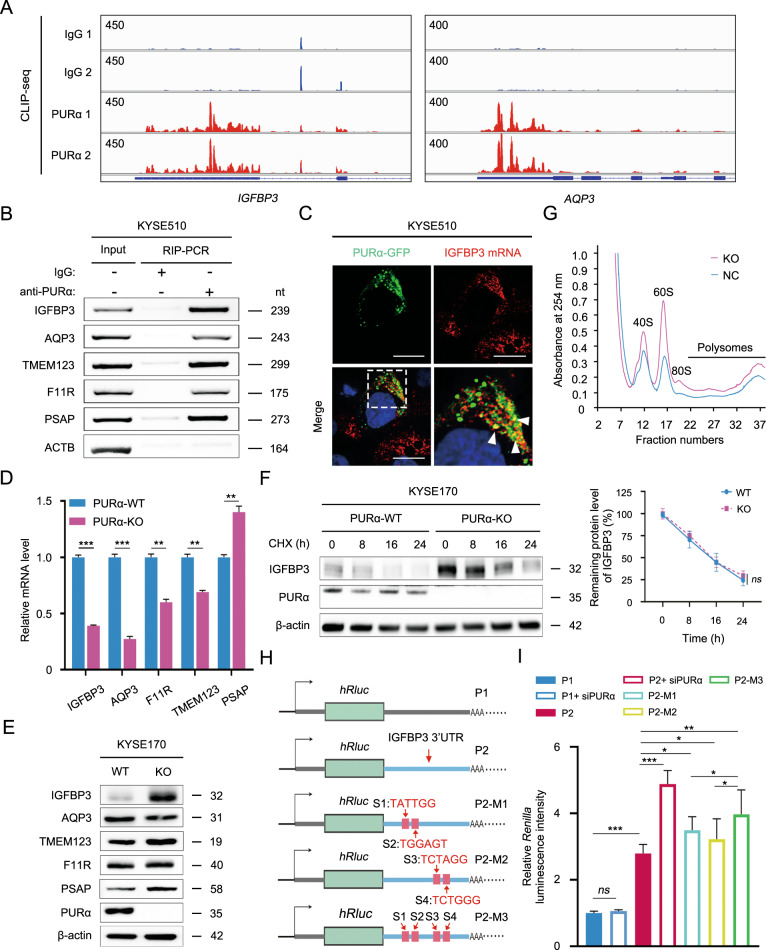
PURα inhibits IGFBP3 protein expression by binding to its 3’UTR. **A** Genome browser views of specific peaks bound by PURα in CLIP-seq were visualized with IGV software. The y-axis indicates the reads per million (RPM) value for the highest peak within the genome browser field for each CLIP-seq run. The annotated RefSeq gene structures are shown in blue, with thin horizontal lines indicating introns and thicker blocks indicating exons. **B** A RIP assay followed by PCR was performed to determine the interaction between PURα and the mRNAs of candidate genes in KYSE510 cells. Specific primers were designed according to the RNA sequences bound by PURα in CLIP-seq. ACTB is a negative control. The results are representative of at least three independent experiments. Nt: nucleotide. **C** The interaction between PURα and IGFBP3 mRNA 3’UTR was visualized using an RNA FISH assay in KYSE510 cells. Scale bars: 30 μm. **D** A qPCR assay was performed to examine the mRNA expression levels of candidate genes in wild-type and PURα-deficient KYSE170 cells. Representative data are presented as the mean ± SEM from three independent experiments. **E** The protein levels of candidate genes were assessed via western blotting in wild-type (WT) and PURα-deficient KYSE170 (KO) cells. **F** The effect of PURα on the protein stability of IGFBP3 was detected in PURα-wild type (WT) and PURα-deficient (KO) KYSE170 cells. WT and KO cells were treated with cycloheximide (CHX) for 0, 8, 16 and 24 h, and the protein level of IGFBP3 was determined by western blot analysis. Representative data are presented from three independent experiments. β-actin, loading control; Ns, not significant. **G** Plot of the absorbance profile of fractions obtained through sucrose gradients to isolate polysomes from KYSE170-WT and KYSE170-KO cells. Peaks and curves indicate the binding of RNA to the marked units of ribosomes or polysomes. **H** Schematic diagram of the psiCHECK™-2 luciferase reporter constructs containing the negative control sequence (P1) or 3’UTR of IGFBP3 mRNA with wild (P2) or mutation (P2-M1, P2-M2 and P2-M3). S1, S2, S3 and S4 indicate the mutated sites in the 3’UTR of IGFBP3 mRNA. The sequence of IGFBP3 3’UTR and mutation is shown in Fig. S3. **I** The relative *Renilla* luciferase activity of P1, P2, P2-M1, P2-M2 and P2-M3 was monitored in KYSE510 cells with or without PURα knockdown using siRNA. The *Renilla* luciferase data were normalized to firefly luciferase data. In all experiments mentioned above, the representative data are presented from at least three independent experiments. Ns not significant; *p < 0.05; **p < 0.01; ***p < 0.001.

To further investigate how cytoplasmic PURα mediates mRNA-based processes, the top 5 candidate genes (*IGFBP3*, *AQP3*, *TMEM123*, *F11R* and *PSAP*) were first selected based on PURα binding in CLIP-seq, and their binding regions were visualized in the integrative genomics viewer (IGV) (Fig. 3A; S2A). According to the sequences interacting with PURα, specific primers for candidate genes were designed for RIP-PCR, and the interactions between PURα and mRNA of the genes were then validated in comparison with the negative control *ACTB* in KYSE510, KYSE30 and KYSE170 cells (Fig. 3B, S2B). Excitingly, RNA FISH not only revealed colocalization between PURα and *IGFBP3* mRNA but also showed that this interaction took place in the cytoplasm (Fig. 3C, S2C). Meanwhile, we knocked out *PURA* in KYSE170 cells by CRISPR/Cas9 (Fig. S2D) and then examined the mRNA expression changes of the candidate genes (Fig. 3D). The mRNA expression levels of *IGFBP3, AQP3, F11R* and *TMEM123* were significantly decreased, while the mRNA level of *PSAP* was prominently increased (Fig. 3D). Unexpectedly, the protein levels of the candidate genes were not consistent with the mRNA levels. Especially for IGFBP3, the protein level was notably increased after knockout of *PURA* (Fig. 3E). This phenomenon was also observed in KYSE170 and KYSE510 cells when the expression level of PURα was knocked down by siRNA (Fig. S2F). However, mRNA and protein stability assays showed that PURα had no impact on the mRNA or protein stability of IGFBP3 (Fig. 3F, S2G). In addition, the expression levels of PURα were negatively correlated with IGFBP3 expression in ESCC cells (Fig. S2H, I). Considering that cytoplasmic stress granules are associated with mRNA translation [32, 33], we hypothesized that cytoplasmic PURα negatively regulated mRNA translation. To test this hypothesis, the polysome profiling assay was further performed, and we observed that knockout of PURα in KYSE170 cells resulted in a higher polysomal peak (Fig. 3G), indicating that deficiency of PURα notably enhanced mRNA translation. Given that PURα mainly binds to the mRNA 3’UTR, the PURα-interacting 3’UTR of *IGFBP3* mRNA was inserted into a dual-luciferase reporter to further investigate whether PURα regulated mRNA translation through the interaction with the mRNA 3’UTR (Figs. 3H, S3A, B). Consistently, knockdown of PURα strikingly increased *Renilla* luciferase activity of the *IGFBP3* reporter in comparison with the nonresponsive control without the *IGFBP3* 3’UTR (Fig. 3I), and mutating the PURα-binding sites also enhanced *Renilla* luciferase activity of the *IGFBP3* reporter (Fig. 3H, I, S3A, B). Together, our results preliminarily supported that cytoplasmic PURα inhibits *IGFBP3* protein expression by binding to its mRNA 3’UTR.

### Cytoplasmic PURα interacts with translation initiation factors directly to regulate protein expression

**Fig. 4:**
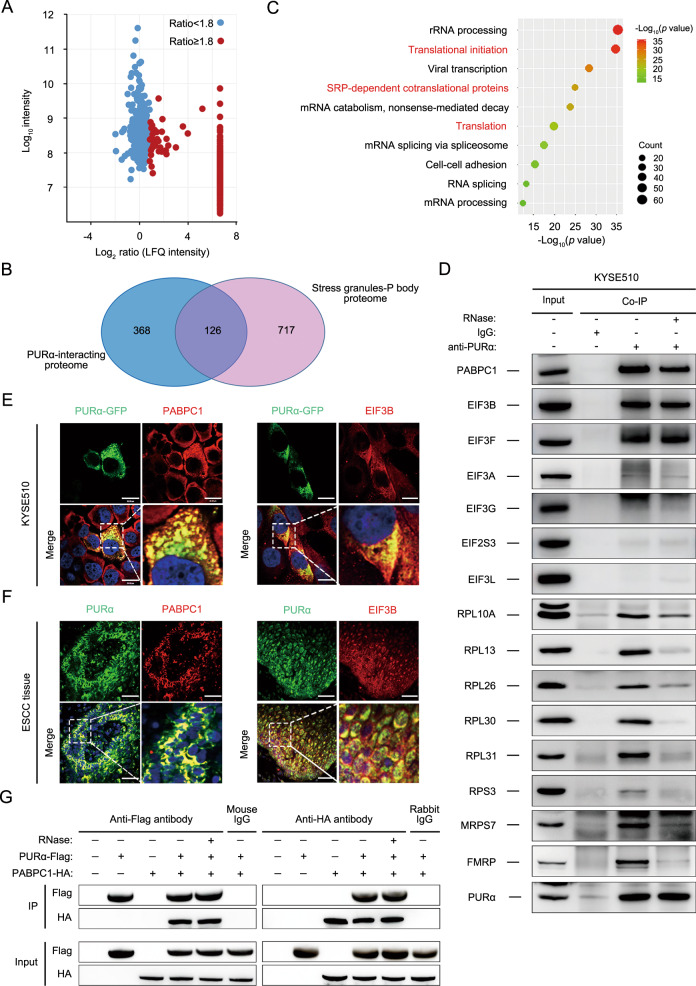
Cytoplasmic PURα interacts with translation initiation factors directly to regulate protein expression. **A** PURα-interacting proteins were identified in KYSE510 cells using a coimmunoprecipitation assay coupled to a label-free quantification (LFQ) proteomic technique. The *x*-axis indicates the log2-transformed LFQ intensity ratios, and the *y*-axis indicates the log10-transformed total intensities. The 494 PURα-interacting proteins (red dots) that met the following screening criteria (unique peptides ≥1 and LFQ intensity ratio ≥1.8) are shown. **B** AVenn diagram between PURα-interacting proteins and the proteome of stress granules and P-bodies was constructed. **C** GO analysis was implemented with DAVID based on PURα-interacting proteins. The top 10 enriched pathways are shown. **D** The interaction between PURα and candidate representative proteins associated with mRNA translation was verified by coimmunoprecipitation in KYSE510 cells. **E** The interaction between PURα and PABPC1 and between PURα and EIF3B was visualized via immunofluorescence staining in KYSE510 cells separately. Scale bars: 30 μm. **F** The interaction between PURα and PABPC1, and between PURα and EIF3B was visualized via immunofluorescence staining in ESCC tissue separately. Scale bars: 100 μm. **G** PURα-Flag and PABPC1-HA were overexpressed in 293 cells separately, and the interaction between exogenous PURα and PABPC1 was detected by a coimmunoprecipitation assay.

To better understand the mechanism of the cytoplasmic PURα effect, the protein networks interacting with PURα in KYSE510 cells were characterized using the label-free quantification (LFQ) proteomic technique. A total of 494 proteins significantly coimmunoprecipated with PURα compared to the control with the following screening criteria: unique peptides ≥1 and LFQ intensity ratio ≥1.8 (Fig. 4A; Supplementary Table S5). Of these proteins, some are known to be PURα-associating proteins, such as PURβ, CDK9, FMR1, and SYNCRIP [9, 18, 26–28], supporting the effectiveness of our experimental strategy to identify PURα-interacting proteins. Given that PURα remarkably intensifies the formation of stress granules as a core component [25, 29–31], the overlap between PURα-interacting proteins and the proteome of stress granules (http://rnagranuledb.lunenfeld.ca/) [42] was constructed, and 126 PURα-interacting proteins were identified to participate in stress granules (Fig. 4B), further suggesting that cytoplasmic PURα is involved in the formation of stress granules. Then, GO analysis was performed on the 494 proteins and showed that the PURα-coimmunoprecipitated proteins were largely enriched in two functional clusters: mRNA translation and mRNA splicing (Fig. 4C), which indicated that cytoplasmic PURα affects mRNA translation.

Furthermore, mRNA translation-related proteins among PURα-interacting proteins were mainly classified into two categories: translation initiation factors and ribosome-related proteins (Fig. S4A). Then, the top proteins from translation initiation factors and ribosome-related proteins were selected based on the LFQ intensity ratio and the score from HPLC–MS/MS analysis separately, such as PABPC1, EIF3B, EIF3F, RPL10A, RPL13 and so on, for follow-up exploration. PABPC1, EIF3B, EIF3F and other ribosome-associated proteins could be coimmunoprecipitated with PURα (Fig. 4D; S4B). Even in the presence of RNase, the coimmunoprecipitated proteins were still clearly detectable for PABPC1 and EIF3B and to lesser extents for the others. Although it is unclear whether the RNase effect was due to the loss of RNA bridges in general or specific RNA-dependent interactions in this assay, it is clear that a group of translation initiation factors interact directly with PURα independent of RNA. In addition, an immunofluorescence assay indicated that PURα and PABPC1 or EIF3B were both colocalized in the cytoplasm in ESCC cells and tissues, respectively, both in vitro and in vivo (Fig. 4E, F), implying that cytoplasmic PURα likely recruits translation initiation factors to modulate protein expression, consistent with the characteristics of cytoplasmic stress granules, which represent assemblies of mRNPs stalled in translation initiation [32, 33].

Next, PABPC1 was mainly focused on since it binds mRNA poly (A) and is involved in translation initiation [43, 44]. The PURα interaction with PABPC1 was further validated by transiently transfecting PURα-Flag and PABPC1-HA followed by a coimmunoprecipitation assay (Fig. 4G). Importantly, our data showed that knockdown of PABPC1 markedly decreased the protein levels of IGFBP3 and TMEM123 in KYSE170 and KYSE510 cells (Fig. S4C), supporting an essential role of PABPC1 in the translation of these two proteins. Together, these results demonstrated that PURα interacts with some translation initiation factors, especially PABPC1 and EIF3B, directly to regulate protein expression in the cytoplasm.

### Knockout of *PURA* markedly inhibits ESCC progression both in vitro and in vivo

**Fig. 5:**
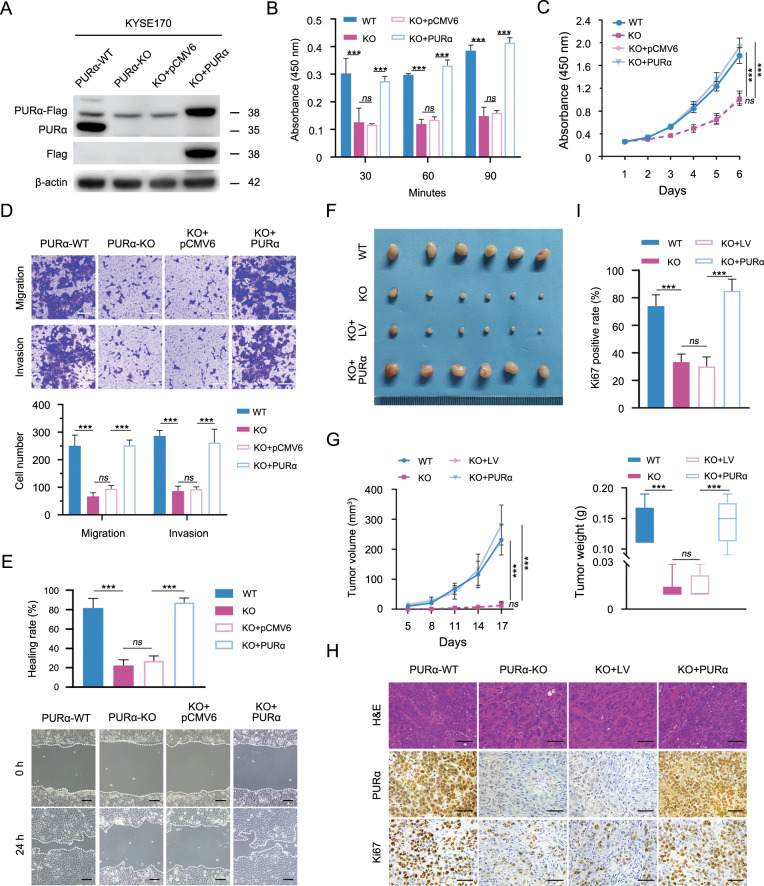
Knockout of PURα markedly inhibits ESCC progression both in vitro and in vivo. The expression of PURα in wild-type KYSE170 cells (WT) was knocked out by CRISPR/Cas9 to construct the PURα-deficient KYSE170 cells (KO). Next, PURα-deficient KYSE170 cells (KO) were transiently transfected with a PURα-containing vector (KO-PURα) to restore the expression of PURα or an empty vector control (KO-pCMV6). **A** The protein levels of PURα were assessed in wild-type (WT), PURα-deficient (KO), KO-pCMV6 and KO-PURα cells by western blotting. **B** An adhesion assay was performed to determine the effect of PURα on cell adhesion capability in fibronectin-coated 96-well plates. The absorbance at 450 nm represents the cell adhesion capability at each time point. **C** The proliferation capacity of WT, KO, KO-pCMV6 and KO-PURα was measured by CCK-8 assays separately. The differences between the growth curves were analyzed by two-way ANOVA followed by a multiple comparisons test. **D**, **E** The migration and invasion capacity of WT, KO, KO-pCMV6 and KO-PURα cells were tested with transwell assays and wound-healing assays, respectively. Scale bar: 100 μm. **F** Macroscopic images of xenograft tumors are shown. PURα-deficient KYSE170 cells were stably transfected with a lentiviral vector containing PURα (LV-PURα) or a negative control lentiviral vector (LV), and then these cells were subcutaneously injected into nude mice (*n* = 6). **G** The volume (left) and weight (right) of harvested xenograft tumors were measured. The differences in tumor volume or tumor weight from six biological repeats were analyzed by one-way ANOVA followed by Tukey’s multiple comparison tests. **H** The expression levels of PURα and Ki67 in xenograft tumors were detected by hematoxylin and eosin (H&E) and immunohistochemical staining. **I** The percentage of Ki67-positive cells per field in immunohistochemical staining (**H**) was calculated. In all experiments mentioned above, the representative data are presented as the mean ± SEM from at least three independent experiments. Ns not significant; *p < 0.05; ***p < 0.001.

Although cytoplasmic PURα interacts with translation initiation factors to regulate protein expression and is markedly related to ESCC progression, it remains poorly understood whether cytoplasmic PURα mediates ESCC progression by regulating mRNA translation. To this end, the effects of PURα on the cell phenotype in PURα-deficient KYSE170 cells were first assessed. The cell adhesion assay indicated that PURα loss notably attenuated cell adhesion capacity, while transient reconstitution of PURα expression in *PURA*-deficient KYSE170 cells completely restored this function, demonstrating that PURα regulates cell–cell adhesion (Fig. 5A, B). We also examined the impacts of PURα on cell proliferation, migration and invasion in *PURA*-deficient KYSE170 cells in vitro. Consistent with previous reports, the CCK-8 assay showed that KYSE170 cells lacking PURα had markedly lower proliferative ability than wild-type KYSE170 cells, and the reduction was effectively reversed when PURα was transiently overexpressed (Fig. 5C). The same tendency was also observed in both the Transwell and wound-healing assays (Fig. 5D, E), indicating that PURα markedly regulates cell migration and invasion capacities.

Furthermore, PURα-deficient KYSE170 cells were stably transfected with a lentiviral vector containing PURα (LV-PURα) or with a negative control lentiviral vector (LV), as confirmed by western blotting (Fig. S5A), and subcutaneously implanted into the back flanks of BALB/c nude mice to generate separate xenograft tumors. The xenograft tumors derived from wild-type KYSE170 cells were remarkably larger and heavier than those derived from PURα-deficient KYSE170 cells (Fig. 5F, G, S5B). However, the difference was fully eliminated when PURα was stably overexpressed in PURα-deficient KYSE170 cells (Fig. 5F, G). Moreover, we observed that the percentages of Ki67-positive cells were significantly decreased in xenograft tumors derived from PURα-deficient KYSE170 cells (Fig. 5H, I), consistent with the previous conclusion that PURα regulates cell growth in vivo.

### Cytoplasmic PURα regulates ESCC progression partially by IGFBP3

**Fig. 6:**
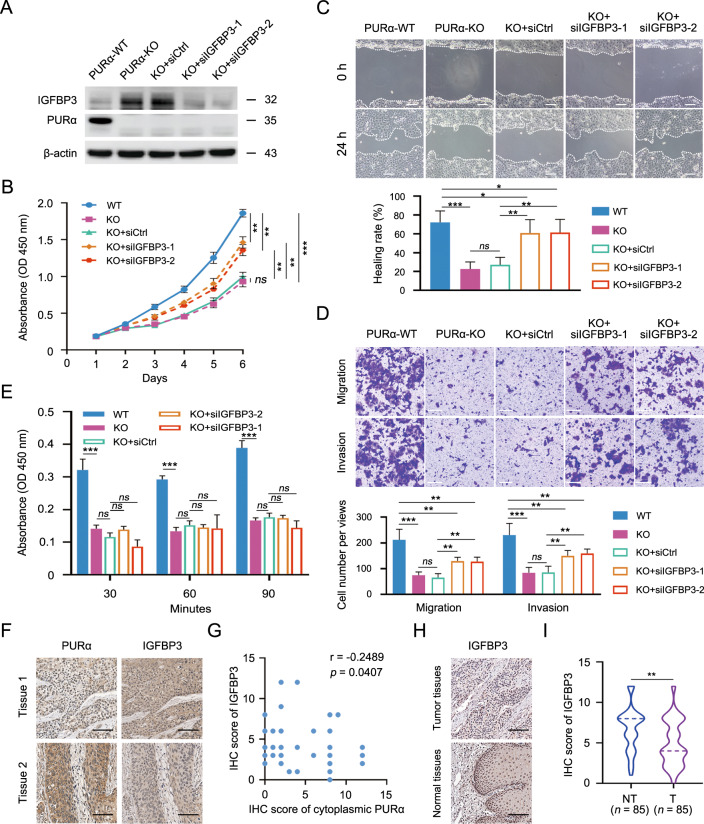
Cytoplasmic PURα regulates ESCC progression partially by IGFBP3. **A** The protein levels of IGFBP3 were assessed by western blotting. IGFBP3 expression was transiently knocked down by siRNA (KO + si-1 and KO + si-2) in PURα-deficient KYSE170 cells. The IGFBP3 protein level was normalized to the β-actin level, and the fold change relative to the control group is shown. **B** The proliferation of PURα-deficient KYSE170 cells transfected with IGFBP3 siRNAs and their controls was measured with a CCK-8 assay. The differences between the growth curves were analyzed by two-way ANOVA followed by a multiple comparisons test. **C**, **D** The effects of IGFBP3 on the migration and invasion capacities of KO + si-1 and KO + si-2 cells were determined with wound-healing (**C**) and Transwell (**D**) assays, respectively. Scale bar: 100 μm. **E** An adhesion assay was performed to determine the effect of IGFBP3 on the cell adhesion capability of PURα-deficient KYSE170 cells. The absorbance at 450 nm represents the cell adhesion capability at each time point. In all experiments mentioned above, the representative data are presented as the mean ± SEM from three independent experiments. Ns, not significant; *p < 0.05; **p < 0.01; ***p < 0.001. **F** Immunohistochemistry (IHC) of PURα and IGFBP3 in the same ESCC tissues is shown separately. Scale bars: 100 μm. **G** The Spearman correlation coefficient was computed based on the IHC score of IGFBP3 and cytoplasmic PURα in ESCC tissues (*n* = 68). **H** IHC of IGFBP3 in ESCC tissue (upper panel) and adjacent nontumorous tissue (bottom panel) was compared. Scale bars: 100 μm. **I** Violin plots of the IHC scores for IGFBP3 in ESCC (T) tissues (*n* = 85) and adjacent nontumor (NT) tissues (*n* = 85) were drawn. **p < 0.01 by Mann–Whitney rank test.

Based on the inhibitory effect of PURα on the protein expression of IGFBP3 in the cytoplasm and the regulation of IGFBP3 during the progression of several types of tumors [45–47], IGFBP3 was selected as a downstream mRNA target to investigate whether cytoplasmic PURα effects on ESCC progression. First, we knocked down the expression of IGFBP3 protein using siRNA (Fig. 6A). The knockdown of IGFBP3 significantly accelerated cell proliferation in PURα-deficient KYSE170 cells, although not completely to the wild-type levels in KYSE170 cells, as shown by a CCK-8 assay (Fig. 6B). Similarly, the migration and invasion abilities were also restored significantly when IGFBP3 was silenced in PURα-deficient KYSE170 cells (Fig. 6C, D). However, IGFBP3 depletion had no influence on cell adhesion in PURα-deficient KYSE170 cells, implying that there might be other target genes mediating the PURα regulation of cell adhesion (Fig. 6E). Importantly, IHC showed that ESCC tissues (*n* = 68) with higher expression levels of cytoplasmic PURα had lower expression of IGFBP3 (Fig. 6F, G), further supporting that PURα inhibits the mRNA translation of IGFBP3. Moreover, we also observed that IGFBP3 expression was significantly decreased in ESCC tissues (*n* = 85) compared to adjacent nontumorous epithelia (*n* = 85) (Fig. 6H, I), implying that IGFBP3 exhibits antitumor properties in ESCC cells. Taken together, these results demonstrate that IGFBP3 is specifically required for most of the PURα-promoting effects on ESCC progression, particularly cell proliferation, migration and invasion, which reveals that cytoplasmic PURα mediates ESCC progression by regulating mRNA translation.

## Discussion

ESCC is one of the most fatal malignancies worldwide. Despite advances in clinical and preclinical research [4–8], the prognosis of ESCC is still poor, and the underlying mechanisms of ESCC progression remain unclear. Here, our immunofluorescence assay indicated that cytoplasmic PURα participated in the formation of stress granules (Fig. 1A, B). Moreover, we found that cytoplasmic PURα levels were significantly higher in ESCC tissues than in adjacent nontumorous epithelia and that ESCC patients with high expression levels of cytoplasmic PURα had worse survival rates than those with low expression levels of cytoplasmic PURα (Fig. 1D–G). However, the mechanisms by which cytoplasmic PURα participates in ESCC progression are poorly understood. Emerging evidence has established that PURα is a core component of cytoplasmic stress granules [25, 29, 31], which are cytoplasmic RNA–protein complexes that form when translation initiation is limited [32, 33]. Given that PURα is a highly conserved RNA-binding protein [9] and cytoplasmic stress granules are involved in regulation of RNA homeostasis [32, 33], we speculate that cytoplasmic PURα likely mediates ESCC progression by regulating RNA-based processes.

PURα has been shown to interact with noncoding RNAs [9, 36, 37] and to transport certain mRNAs along microtubules to specific translation sites [27, 28], but the characteristics of RNA bound to PURα have not been fully elucidated. To accurately profile the RNA targets of human PURα, we first performed CLIP-seq and found that PURα mainly binds to the mRNA 3’UTR (Fig. 2A, B). In addition, motif enrichment analysis showed that PURα preferentially binds to UG-/U-rich sequences (Fig. 2C). Intriguingly, analysis of the homology among RNA species known to be bound by PURα revealed that each of these RNA species possesses a potential stem-and-loop structure and contains a U-rich motif that is partly disrupted and supplanted with G-rich sequences [26], implying that these structures are important for the interaction between PURα and RNA. Of course, there are also conflicting findings; for instance, several reports have confirmed that PURα preferentially binds to G-rich motifs to regulate amyotrophic lateral sclerosis (ALS) and frontotemporal dementia (FTD) [48]. Nevertheless, we have comprehensively characterized RNAs bound to PURα for the first time.

To further clarify the regulatory effect of PURα on mRNA, we detected the expression levels of candidate genes in PURα-deficient KYSE170 cells and discovered that the protein expression levels of the candidate genes were not consistent with the mRNA expression levels (Fig. 3D, E). In particular, the mRNA expression level of *IGFBP*3 significantly decreased after *PURA* knockout, while the protein level of *IGFBP3* increased. The observed effects on translation following PURα silencing could be global and not necessarily specific for IGFBP3. One possible reason could be the different time course of mRNA and protein level changes after silencing of *PURA*. Decreased mRNA level likely is interpreted by both low-transcription and high degradation rate. The mRNA stability assay indicated that PURα didn’t affect mRNA degradation of IGFBP3. As previous reports that PURα is a well-known transcription factor [9], the nascent RNA assay demonstrated that PURα mediated the transcription of several candidate genes, including IGFBP3 (Fig. S2E). Systematic studies quantifying transcripts and proteins has revealed that translation rates, translation rate modulation, regulation of protein’s half-life and protein synthesis delay contribute to the expression level of protein, except for mRNA transcripts. Sometimes mRNA and protein level don’t go with each other for many genes [49]. Moreover, the protein stability assay showed that PURα had no impact on the protein’s half-life of IGFBP3 (Fig. 3F). As a result of the regulation of mRNA translation by cytoplasmic stress granules, we hypothesized that PURα negatively regulated mRNA translation in the cytoplasm. To this end, polysome profiling analysis was performed, and knockout of PURα in KYSE170 cells resulted in a higher polysomal peak (Fig. 3G), indicating that loss of PURα significantly promoted mRNA translation. Interestingly, the deficiency of PURα also caused higher peaks of 40 S and 60 S ribosomal subunits (Fig. 3G), further uncovering that PURα likely modulates ribosomal biogenesis. In agreement with these data, the CLIP-seq showed that PURα notably binds to a subset of small nucleolar RNA (snoRNA), which are core components of small nucleolar ribonucleoprotein particles (snoRNPs) [50], and many PURα-interacting proteins were relevant to the core components of snoRNPs, such as NOP56 and NOP58. In view of the functions of snoRNPs in ribosomal RNA processing [51, 52], PURα may modulate ribosomal biogenesis to regulate mRNA translation. In summary, our results demonstrate that PURα regulates mRNA translation at multiple levels. In addition, a nascent RNA assay was also performed to determine whether PURα regulated the mRNA transcription of candidate genes since PURα is a well-known transcription factor [9]. As expected, the assay showed that PURα mediated the transcription of several candidate genes, including *IGFBP3* (Fig. S2E).

Despite increasing evidence indicating that PURα facilitates the formation of stress granules and that stress granules predominantly modulate mRNA translation initiation [25, 29, 31–33], the mechanistic basis by which cytoplasmic PURα inhibits mRNA translation remains poorly understood. To better determine this mechanism, we carried out label-free quantification (LFQ) proteomic analysis and found that PURα-interacting proteins are largely correlated with translation initiation factors and ribosome-related proteins (Fig. S4A). Many representative proteins were chosen and further verified by coimmunoprecipitation and immunofluorescence assays (Fig. 4C–F), which suggested that cytoplasmic PURα interacted with translation initiation factors directly to regulate mRNA translation. In addition, in line with previous reports [53, 54], we observed that some PURα-interacting proteins were involved in viral transcription (Fig. 4C). We have demonstrated a specific effect on translation through the binding of IGFBP3 3’ UTR by PURa. However, it also appears to have a global effect on the polysomes (Fig. 3G). Moreover, it interacts with multiple ribosomal proteins (Fig. 4D). Therefore, PURa likely acts on multiple targets in the translation machinery besides its direct binding to the mRNA.

Considering the negative regulation of PURα on the mRNA translation of IGFBP3 in the cytoplasm and the effect of IGFBP3 on tumor progression [45–47], we hypothesized that cytoplasmic PURα plays an oncogenic role by inhibiting the mRNA translation of IGFBP3 in ESCC. To test this hypothesis, we first validated that knockdown of IGFBP3 partly reversed the inhibitory effects of PURα loss on ESCC progression, especially cell proliferation, migration and invasion (Fig. 6A–D), and observed that the expression of PURα was negatively correlated with IGFBP3 expression in ESCC tissues (Fig. 6F, G), which further supported that IGFBP3 was partially required for PURα to regulate ESCC progression. Although we demonstrated that IGFBP3 expression was significantly decreased in ESCC tissues (Fig. 6H, I), which implies that IGFBP3 exhibits antitumor properties in ESCC cells, some reports have demonstrated that IGFBP3 could promote ESCC progression [55]. Growing evidence indicates that the effects of IGFBP3 are likely dependent on cell types, the cellular environment, IGFBP3 concentration, stress-related conditions and the availability of its binding partners [56, 57]. For example, as a result of a higher affinity for IGFs than IGF receptors (IGFRs), IGFBP3 can restrict access of IGFs to IGFRs and consequently inhibit their tumor-promoting effects [58, 59]. On the other hand, IGFBP3 also potentiate IGFs activity by presenting IGFs to the receptor through loss of binding affinity either by proteolysis or association with the cell surface receptor, and thereby promote cell survival and cell growth [56, 57]. Therefore, it is difficult to decide the ultimate outcome of either growth inhibition or cytoprotection of IGFBP3 in cancer progression. In any case, we showed that knockdown of IGFBP3 reversed the inhibitory effect of PURα loss on ESCC progression. In addition, we also found that knockdown of *IGFBP3* did not rescue the cell adhesion capacity, suggesting that there is another pathway correlated with PURα that mediates ESCC progression.

**Fig. 7:**
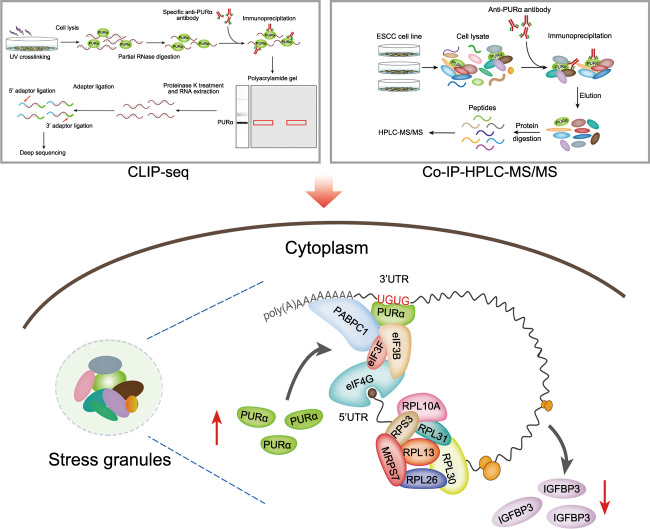
Schematic of the mechanism by which cytoplasmic PURα inhibits mRNA translation to mediate ESCC progression. As a novel component of cytoplasmic stress granules, PURα preferentially binds to UG-/U-rich motifs located in the 3’UTRs of mRNAs and recruits translation initiation factors to attenuate mRNA translation.

In conclusion, our study demonstrates that PURα participates in ESCC progression by inhibiting mRNA translation initiation (Fig. 7). Cytoplasmic PURα preferentially binds to UG-/U-rich motifs located in the 3’UTRs of mRNAs and recruits translation initiation factors to regulate mRNA translation (Fig. 7). These findings may prove clinically useful for developing a new therapeutic target for ESCC progression.

## Materials and methods

### Crosslinking immunoprecipitation and high-throughput sequencing (CLIP-seq)

Cells were washed with ice-cold PBS 3 times and then subjected to UV crosslinking with UVC radiation (254 nm) at 400 mJ/cm^2^. The crosslinked cells were scraped off the plate and collected by centrifugation at 1000 × *g* for 5 min. The cells were lysed in cold lysis buffer (1× PBS, 0.1% SDS, 0.5% NP-40 and 0.5% sodium deoxycholate) supplemented with 200 U/ml RNase inhibitor (Takara, Kyoto, Japan) and protease inhibitor cocktail (Roche, Basel, Switzerland) for 10 min. The cell lysates were cleared by centrifugation at 10,000 rpm for 20 min at 4 °C, and the supernatants were utilized for RNase digestion and immunoprecipitation. Then, RNase T1 (Thermo Fisher Scientific, Waltham, MA, USA) was added to the lysate to a final concentration of 1 U/μl, and the mixture was incubated at 22 °C for 15 min. For immunoprecipitation, 300 μl of lysate was incubated with 10 μg of anti-PURα antibody (Abcam, Cat# ab125200) or control IgG antibody overnight at 4 °C. The immunoprecipitates were further incubated with protein A Dynabeads for 3 h at 4 °C. After collection with a magnetic field and removal of the supernatants, the beads were sequentially washed twice with wash buffer (250 mM Tris 7.4, 750 mM NaCl, 10 mM EDTA, 0.1% SDS, 0.5% NP-40 and 0.5% sodium deoxycholate) and polynucleotide kinase (PNK) buffer (50 mM Tris, 20 mM EGTA and 0.5% NP-40). Protective on-bead digestion was performed by adding MNase (Thermo Fisher Scientific) to a final concentration of 1 U/ml followed by incubation at 37 °C for 15 min. After washing with PNK buffer as described above, dephosphorylation and phosphorylation were performed with calf intestinal alkaline phosphatase (New England Biolabs, Ipswich, MA, USA) and PNK, respectively. The immunoprecipitated protein–RNA complex was eluted from the beads by heat denaturing and resolved on a Novex Bis-Tris 4–12% precast polyacrylamide gel (Thermo Fisher Scientific). The protein–RNA complexes were cut from the gel, and RNA was extracted with TRIzol after digesting the proteins. The recovered RNA was used to generate a paired-end sequencing library with a TruSeq small RNA library preparation kit (Illumina, San Diego, CA, USA) following the manufacturer’s instructions. Libraries corresponding to 200–500 bp were purified, quantified and stored at −80 °C until they were used for sequencing. For high-throughput sequencing, the libraries were prepared following the manufacturer’s instructions and applied to an Illumina NextSeq 500 system for 151 bp paired-end sequencing by ABlife, Inc. (Wuhan, China).

For CLIP-seq data, adaptors and low-quality bases were trimmed from the raw sequencing reads using the FASTX-Toolkit (Version 0.0.13), and reads less than 16 nt in length were discarded. The clean reads were aligned to the human GRCH38 genome using TopHat2 with 2 mismatches [60].

After the reads were aligned onto the genome, we discarded the reads with multiple genomic locations due to ambiguous origination. Identically aligned reads were counted and merged as unique reads. The binding regions of PURα in the genome were identified using the “ABLIRC” strategy as previously reported [40]. The PURα and IgG samples were analyzed by the simulation independently. After simulation, the PURα peaks that overlapped with IgG peaks were removed. The target genes of PURα were finally determined by analyzing the locations of all the PURα binding peaks on the human genome, and the binding motifs of PURα were called with Homer software [61].

### Mass spectrometry (MS) and data analysis

For protein sample preparation, the whole-cell extracts were subjected to immunoprecipitation with a specific anti-PURα antibody or rabbit IgG conjugated to magnetic beads as described in the coimmunoprecipitation assay section, and the beads destined for MS analysis were washed in 0.1% NP-40 lysis buffer with no detergent. Then, the proteins coupled to the bead-Ig complex were treated with trypsin as described previously [62].

LC–MS/MS analysis was performed using an Ultimate 3000 RSLCnano system coupled online to a Q Exactive mass spectrometer (Thermo Fisher Scientific). Four microliters of sample was injected onto a trap column (Acclaim PepMap 100, 300 μm × 5 mm, C18, 5 μm, 100 Å; flow rate 30 μl/min). Subsequently, the peptides were separated on an analytical column (Acclaim PepMap RSLC, 75 μm × 50 cm, nano Viper, C18, 2 μm, 100 Å) with a gradient of 5% to 40% solvent B over 120 min [solvent A: 0.1% formic acid (FA), solvent B: 0.1% FA, 84% acetonitrile (ACN); flow rate 400 nl/min; column oven temperature 60 °C]. The eluted peptides were analyzed online with a Q Exactive mass spectrometer using a nanoelectrospray interface. Ionization (1.8 kV ionization potential) was performed with stainless-steel emitters. The peptide ions were obtained through the following data-dependent acquisition steps: (1) a full MS scan (mass-to-charge ratio (m/z) 400 to 1800) and (2) MS/MS. The MS resolution was 70,000 at m/z 400, the automatic gain control was 3 × 10^6^, and the maximum injection time 20 ms. For MS2, the resolution was 17,500 at m/z 400, the automatic gain control was 2 × 10^5^, the maximum injection time 100 ms, the isolation window m/z = 2, the normalized collision energy was 27, the underfill ratio was 1%, and the intensity threshold was 2.0 × 10^4^. The charge state was 2, and the dynamic exclusion time was 30 s.

For data analysis, the resulting MS/MS data were processed using Thermo Proteome Discoverer (PD, v2.4.1.15). The tandem mass spectra were searched against the Homo sapiens database concatenated with a reverse decoy database. Trypsin was specified as the cleavage enzyme, and up to 2 missed cleavages were allowed. The mass tolerance values for precursor ions and fragment ions were set as 10 ppm and 0.02 Da, respectively. Carbamidomethyl on Cys was specified as the fixed modification. Acetylation on the protein N-terminus and oxidation on Met were specified as the variable modifications. The peptide false discovery rate (FDR) was calculated using Percolator provided by PD. When the *q* value was smaller than 1%, the peptide spectrum match (PSM) was considered to be correct. Peptides assigned only to one given protein group were considered unique. The FDR was also set to 0.01 for protein identification. The peak areas of fragment ions were used to calculate the relative intensity of precursor ions for selected peptides. At least one peptide was selected for quantification of one protein. The means of the relative intensities of selected peptides represent the relative expression levels of the proteins.

### Statistical analysis

Statistical analysis was performed using GraphPad Prism version 8.0 software (GraphPad Software, San Diego, CA, USA). All data are presented as the mean ± SEM. Differences between two groups were compared by two-tailed Student’s *t* tests or the Mann–Whitney rank test. Differences among the means of three or more groups were analyzed by analysis of variance (ANOVA) followed by a multiple comparisons test. The correlation between two groups was examined using Pearson’s correlation. Survival analyses were performed by the Kaplan–Meier method and compared by the log-rank test. A *p* value less than 0.05 was considered to indicate statistical significance (*p < 0.05, **p < 0.01, ***p < 0.001).

## Supplementary information

Supplementary information

Supplementary Table S4

Supplementary Table S5

## Data Availability

The data that support the findings of this study are available from the corresponding authors upon reasonable request. The CLIP-seq and IP-MS data discussed in this paper have been deposited in the China National Center for Bioinformation and the ProteomeXchange Consortium via the iProX partner repository. They are accessible through accession number HRA001518 (at https://ngdc.cncb.ac.cn/gsa-human/s/DPjDdj0w) or password lqz8 (at https://www.iprox.cn/page/SSV024.html;url=1636634694876GP62).
