# Supplementary material for: Forming cytoplasmic stress granules PURα suppresses mRNA translation initiation of IGFBP3 to promote esophageal squamous cell carcinoma progression
Source: Oncogene. 2022 Aug 9;41(38):4336–48. doi: 10.1038/s41388-022-02426-3 (PMC9481463; doi:10.1038/s41388-022-02426-3)
Supplement: Supplementary file 1 — Supplementary information [file 41388_2022_2426_MOESM1_ESM.pdf]

Forming cytoplasmic stress granules PURα suppresses mRNA translation initiation of IGFBP3 to promote esophageal squamous cell carcinoma progression

Lusong Tian[1*], Xiufeng Xie[1*], Urmi Das[2], Yuling Chen[3], Yulin Sun[1], Fang Liu[1], Haizhen Lu[4], Peng Nan[1], Ying Zhu[1], Xinglu Gu[1], Haiteng Deng[3], Jiuyong Xie[2], Xiaohang Zhao[1]

[1]State Key Laboratory of Molecular Oncology, National Cancer Center/National Clinical Research Center for Cancer/Cancer Hospital, Chinese Academy of Medical Sciences and Peking Union Medical College, Beijing, China

[2]Department of Physiology & Pathophysiology, Max Rady College of Medicine, Rady Faculty of Health Sciences, University of Manitoba, Canada

[3]MOE Key Laboratory of Bioinformatics, Center for Synthetic and Systematic Biology, School of Life Sciences, Tsinghua University, Beijing, China

[4]Department of Pathology, National Cancer Center/Cancer Hospital, Chinese Academy of Medical Sciences and Peking Union Medical College, Beijing, China

Supplementary Materials and Methods

Supplementary Figures

Supplementary Figure S1. PURα preferentially binds UG-/U-rich motifs

Supplementary Figure S2. PURα inhibits IGFBP3 protein expression by binding to its 3’UTR.

Supplementary Figure S3. The sequence of IGFBP3 3’UTR and mutation was showed in psiCHECK™-2 luciferase reporter.

Supplementary Figure S4. PURα-interacting proteins are implicated in mRNA translation.

Supplementary Figure S5. Knockout of PURα markedly inhibits ESCC progression *in vivo*.

Supplementary Tables

Supplementary Table S1. Correlations between expression of cytoplasmic PURα and clinicopathological features in ESCC patients

Supplementary Table S2. Description of high-quality reads in CLIP-seq

Supplementary Table S3. Description of uniquely mapped reads to the reference genome in CLIP-seq

Supplementary Table S4. Specific peaks bound to PURα in CLIP-seq

Supplementary Table S5. PURα-interacting proteins

Supplementary Table S6. Related resource information

Supplementary Materials and Methods

Cell culture

The human ESCC cell lines KYSE30, KYSE150, KYSE170 and KYSE510 were generously provided by Dr. Shimada (Hyogo College of Medicine, Hyogo, Japan), and esophageal epithelial cells Het-1A (Cat# CRL-2692) and HEK-293 (Cat#CRL-1573) were obtained from ATCC separately. The cells were cultured in RPMI 1640 medium supplemented with 10% fetal bovine serum (HyClone, Logan, UT, USA), 100 U/ml penicillin and 100 μg/ml streptomycin at 37°C with 5% CO_2. All cell lines were authenticated by short tandem repeat (STR) analysis, and mycoplasma tests were negative.

Plasmid construction and transfection

The cDNA encoding human *PURA* gene (NM_005859) was cloned into the pCMV6-Myc-DDK-AC vector (OriGene, Rockville, MD, USA) to generate a recombinant vector. It was transiently transfected into cells with Lipofectamine 3000 (Thermo Fisher, Carlsbad, CA, USA). In addition, the cDNA of the human *PURA* gene was inserted into a lentiviral vector, Ubi-MCS-3FLAG-SV40-puromycin (GeneChem, Shanghai, China), to restore the expression of PURα in PURα-deficient KYSE170 cells.

For siRNA transfection, siRNAs targeting human *IGFBP3*, *PURA* and *PABPC1* were designed and synthesized by GenePharma (Shanghai, China). The specific target sequences of the siRNAs are listed in Supplementary Table S6. Then, 5×10^5 cells were plated in a 6-well plate and incubated at 37°C. When the cell confluence reached 50-60%, the cells were transfected with specific siRNA (100 pM) using Lipofectamine 2000 (Thermo Fisher).

CRISPR/Cas9 mediated *PURA* gene KO cell line

Guide RNAs (gRNAs) targeting the human *PURA* gene were first designed using the CRISPR design tool (http://cripor.tefor.net) (sgRNA 1:

TGGGGCACCCCGGCTCGGGC; sgRNA 2: AGCGGGTGGACATCCAGAAC) and cloned into the pVG-U6-gRNA-CMV-spCas9-Puro vector (Vigene Biosciences, Shandong, China). Then, 4×10^5 KYSE170 cells were plated in 6-well plates and cultured at 37°C with 5% CO_2. When the cell confluence reached 80%, the cells were transfected with the sgRNA vectors. Forty-eight hours after transfection, cell culture medium containing 4 μg/ml puromycin was added to a 6-well plate to screen specific cells successfully transfected with the recombinant sgRNA vector. Next, single clones were expanded and verified by PCR. Finally, the PURα-deficient KYSE170 cell line was constructed.

Coimmunoprecipitation assay

Dynabeads Protein G (Thermo Fisher Scientific) were resuspended thoroughly to obtain a homogeneous suspension, and 50 μl of Dynabeads Protein G were transferred to a 1.5 ml eppendorf tube at room temperature (RT). Then, the tube was placed on a magnet for 1 min, the supernatant was discarded, and 1 ml of citrate-phosphate buffer (pH 5.0) was added to wash the Dynabeads Protein G. Then, 10 μg of anti-PURα antibody (Abcam, Cat# ab125200) or rabbit IgG was added to 200 μl of PBS solution with 0.2% Tween, and the resulting mixture was transferred to prepared magnetic beads and incubated for 1.5 h RT with end-over-end rotation. The Dynabeads Protein G-Ig complex was washed twice in 1 ml of 0.2 M triethanolamine (pH 8.2) and incubated in 1 ml of fresh 0.2 M triethanolamine (pH 8.2) with 20 mM dimethyl pimelimidate (DMP×2HCl) for 30 min RT to crosslink antibody to the beads with end-over-end rotation. Subsequently, the beads were incubated in 50 mM Tris (pH 7.5) for 15 min to stop the crosslinking. After this step, the tube was placed on the magnet, the supernatant was discarded, and the crosslinked beads were washed three times with 1 ml of PBS solution with 0.2% Tween-20. Next, 1 ml of 0.1 M glycine (pH 2.5) was added to remove the non-covalently bound antibodies. Then, 500 μl of the whole-cell extract was transferred to the tube with the Dynabeads-Ig complex and incubated overnight at 4°C with end-over-end rotation. After immunoprecipitation, the tube was placed on the magnet for 2 min to collect the Dynabeads Protein

G-Ig-antigen complex at the tube wall. The beads were then washed 5 times with 1 ml of 0.1% NP-40 lysis buffer supplemented with protease inhibitors. Finally, 50 µl of 2× Laemmli sample buffer with 50 mM DTT was added to the tube, and the tube was incubated at 100°C for 5 min to obtain a precipitated protein sample for SDS-PAGE. All specific antibodies are listed in Supplementary Table S6.

Immunofluorescence assay

Cells growing on coverslips in 6-well plates were fixed with paraformaldehyde (4%, w/v) for 20 min and permeabilized for 20 min with Triton X-100 (0.2%, v/v). Next, the cell samples were blocked with 2% (w/v) bovine serum albumin (BSA) for 1 h RT. The cells were then incubated overnight with the appropriate primary antibodies at 4°C. All specific antibodies are listed in Supplementary Table S6. After this step, the cells were incubated with the corresponding fluorescent secondary antibodies RT for 1 h. Finally, the cell nuclei were stained with DAPI solution (Sigma-Aldrich, St. Louis, MO, USA). Fluorescence images were captured with a laser scanning confocal microscope (PerkinElmer).

Western blot analysis

Cells were washed with PBS 3 times, lysed with RIPA buffer (50 mM Tris-HCl, pH 8.0, with 150 mM NaCl, 1.0% NP-40, 0.5% sodium deoxycholate, and 0.1% SDS) containing protease inhibitor cocktail (Roche) for 30 min, and sonicated. The cell lysates were prepared by centrifugation at 12 000 rpm for 15 min. Then, 25 µg of each protein sample was electrophoresed on 4%-20% gradient SDS-PAGE gels, and the separated proteins were transferred to polyvinylidene difluoride membranes (Millipore, Bedford, MA, USA) by electroblotting. The membranes were blocked for 1 h with nonfat dry milk in PBS (5%, w/v) and incubated with primary antibodies at 4°C overnight. All specific antibodies are listed in Supplementary Table S6. The proteins were detected with horseradish peroxidase (HRP)-conjugated secondary antibodies and visualized with Renaissance Plus Reagent (Life Technologies, Grand Island, NY, USA).

RNA immunoprecipitation (RIP) assay

RNA RIP assays were performed using a Magna RIP^{TM} RNA-Binding Protein Immunoprecipitation Kit (Millipore) according to the manufacturer's instructions. Briefly, the cells were washed twice on 10 cm^2 plates with 10 ml of ice-cold PBS, scraped from each plate, and collected and lysed with RIP lysis buffer. Then, 50 μl of magnetic bead suspension was transferred to each tube, and 10 μg of anti-PURα or negative control rabbit IgG was added to each tube for immunoprecipitation. After crosslinking between the magnetic beads and the antibody, the RIP lysate was added to the bead-antibody complex in RIP immunoprecipitation buffer. For purification of RNA, all protein-RNA complexes were first incubated at 55°C for 30 min with shaking to digest the protein in proteinase K buffer. After incubation, the tubes were briefly centrifuged and placed on a magnetic separator to collect the supernatant containing RNA. Then, 850 μl of absolute ethanol with salt solution I, salt solution II, and precipitate enhancer was mixed with the aqueous phase containing RNA to collect the RNA, and the RNA pellets were resuspended in 20 μl of RNase-free water. Finally, the immunoprecipitated of RNA was analyzed by PCR. The primer sequences are shown in Supplementary Table S6.

RNA extraction, RT-PCR and quantitative PCR (qPCR) assays

Total RNA was extracted using TRIzol reagent (Thermo Fisher), and reverse transcription was performed with a HiFiScript cDNA Synthesis Kit (CWbiotech, Jiangsu, China). qPCR was carried out on an ABI QuantStudio 5 device (Applied Biosystems, Foster City, CA, USA) using SYBR Green Real-Time PCR Master Mix (Takara). The relative mRNA expression levels were determined according to the cycle threshold (Ct) and were normalized against the ACTB levels using the $2^{-\Delta\Delta Ct}$ formula. The primer sequences are shown in Supplementary Table S6.

RNA fluorescence in situ hybridization (FISH)

Cells growing on coverslips in 6-well plates were transiently transfected with

PURA-GFP with Lipofectamine 3000 (Thermo Fisher). Twenty-four hours after transfection, the cells were fixed with paraformaldehyde (4%, w/v) for 10 min and permeabilized for 5 min with Triton X-100 (0.2%, v/v). Then, 1 ml of 4% paraformaldehyde was added again, and the cells were washed twice with 1 ml of 1× PBS. Next, 1 ml of Wash Buffer A (Stellaris RNA FISH Wash Buffer A (Biosearch Technologies, Novato, CA, USA; Cat# SMF-WA1-60): nuclease-free water: deionized formamide = 2:7:1) was added to the 6-well plates, and the plates were incubated RT for 5 min. Within a humidified chamber, 100 μl of Hybridization Buffer [Stellaris RNA FISH Hybridization Buffer (Biosearch Technologies Cat# SMF-HB1-10): deionized formamide = 9:1] containing probes was dispensed onto the Parafilm. The probe sequences are listed in Supplementary Table S6. The coverslips were gently transferred, cell side down, onto the 100 μl drop of hybridization buffer containing the probes and incubated in the dark at 37°C for 8 h. After hybridization, the coverslips were gently transferred to a fresh 6-well plate containing 1 ml of wash buffer A and incubated in the dark at 37°C for 30 min. Then, 1 ml of 6-diamidino-2-phenylindole (DAPI) nuclear stain was added, and the coverslips were incubated in the dark at 37°C for 30 min to counterstain the nuclei. Finally, the DAPI staining buffer was aspirated, 1 ml of wash buffer B was added, and the cells were incubated RT for 5 min. The RNA FISH images were captured with a laser scanning confocal microscope (PerkinElmer, Waltham, MA, USA).

Nascent RNA analysis

Nascent RNA analysis was performed as previously reported [1]. First, 4-thiouridine (4sU) (Abcam, Waltham, MA, USA) was added to the cell culture medium to a final concentration of 1 mM and the RNA was labeled in a CO_2 incubator. After incubation for 30 min, total cellular RNA was isolated from the cells using TRIzol reagent (Thermo Fisher). For biotinylation of 4sU-labeled RNA, biotin-HPDP (Pierce) was mixed with 80 μg of total cellular RNA in biotinylation buffer, and the mixture was incubated RT for 1.5 h with rotation. The total cellular RNA containing biotinylated 4sU-labeled RNA was reprecipitated and collected. Then, denaturation of RNA

samples was performed at 65°C for 10 min followed by rapid cooling on ice for 5 min, and the biotinylated RNA was captured using µMACS streptavidin beads and columns (Miltenyi, Bergisch Gladbach, Germany). Finally, the labeled RNA was eluted with 100 µl of freshly prepared 100 mM dithiothreitol (DTT) and recovered from the washing fractions using RNeasy MinElute Spin columns (Qiagen, Dusseldorf, Germany). The nascent RNA was analyzed by qPCR. The primer sequences are shown in Supplementary Table S6.

Polysome profiling analysis

Polysome profiling analysis was performed as previously reported [2]. In brief, the cells were plated in 10 cm dish and total lysate was prepared by scraping in hypotonic buffer (1% Triton X-100, 20 mM Tris-HCl (pH 7.4), 2.5 mM $MgCl_2$, 150 mM NaCl, 1 mM DTT, 5 U/ml RNase inhibitor and protease inhibitor cocktail) supplemented with 0.1 mg/ml cycloheximide (CHX). Samples were centrifuged at 17000 g for 5 min at 4°C. Supernatants (cytosolic cell extracts) were collected and absorbance at 254 nm was measured. Approximately 20 OD_{260} of lysate was layered over 10% - 50% cold sucrose gradients in buffer (20 mM Tris-HCl (pH 7.4), 2.5 mM $MgCl_2$, 150 mM NaCl, 1 mM DTT, 5 U/ml RNase inhibitor and protease inhibitor cocktail). Gradients were centrifuged at 39000 rpm in a Beckman SW28 rotor for 2 h at 4°C. After centrifugation, placing sucrose gradients on Automated Density Fractionation System and proceeding with automated fractionation and collection of fractions (0.5 ml each), as described by the manufacturer. Collecting each fraction into individual eppendorf tubes with continuous monitoring of absorbance at 254 nm.

Cell proliferation assay

For the cell proliferation assay, cells were seeded at a density of 1500 cells per well in 96-well plates and cultured for 6 days. Every day at the same time, a total of 100 µl of serum-free medium containing 10 µl of Cell Counting Kit-8 (CCK-8) reagent (Biosharp, Anhui, China) was added to each well, and the cells were incubated for 1 h. Then, the absorbance at 450 nm was measured using a microplate reader to evaluate

cell viability. This experiment was performed in triplicate, and the results are presented as the mean ± SEM.

Cell migration and invasion assay

A cell migration and invasion assay was performed as described previously [3]. In brief, 5×10^4 cells were added to the upper chamber in 100 μl of serum-free RPMI 1640 medium, and 600 μl of RPMI 1640 medium with 10% fetal bovine serum was added to the lower chamber. After 24-48 h of incubation, the cells remaining inside the upper chamber were removed with a wet cotton swab, and the inserts were fixed with methanol for 10 min and then stained with 0.25% crystal violet. The invaded cells were counted in five randomly selected microscopic views. Cell invasion was measured using Transwell chambers coated with Matrigel (Corning, NY, USA) for a cell migration assay.

Cell adhesion assay

A cell adhesion assay was performed on fibronectin-coated 96-well plates as reported previously [4]. In brief, a 96-well plate was first coated with human fibronectin (Prospec, Ness Ziona, Israel), and 1×10^4 cells were seeded in the plate separately and incubated at 37°C for different times to determine the ability of the cells to adhere to fibronectin. Then, a total of 100 μl of RPMI 1640 medium containing 10 μl of CCK-8 reagent was added to each well, and the plate was incubated for 2 h. Finally, the absorbance at 450 nm was measured using a microplate reader to assess cell adhesion capacity.

Immunohistochemistry (IHC)

ESCC tissue microarrays (Superbiotek, Shanghai, China) were first deparaffinized in a series of gradient ethanol baths, rehydrated, and immersed in methanol containing 0.3% hydrogen peroxide for 10 min to block endogenous peroxidase RT. Subsequently, the microarrays were heated for 30 min in a pH 6.0 antigen retrieval solution to induce antigen retrieval and then incubated overnight with an anti-PURα

antibody (Abcam, Cat# ab79936) at 4°C. Staining was performed using a Prolink-2 Plus HRP rabbit polymer detection kit (Golden Bridge, Bothell, WA, USA) according to the manufacturer's instructions. Images were captured using Aperio ScanScope CS software (Aperio Technologies, Vista, CA, USA).

The results were evaluated based on the intensity and extent of staining by two senior pathologists independently (double blinded) as described previously [5]. Briefly, the PURα staining area was scored as follows: 0, <5% of the epithelial cells stained in the respective lesions; 1, 5–25% of the epithelial cells stained; 2, 26–50% of the epithelial cells stained; 3, 51–75% of the epithelial cells stained; and 4, ≥75% of the epithelial cells stained. The intensity was graded as follows: 0, negative; 1+, weak (yellow); 2+, moderate (light brown); and 3+, strong (dark brown). A final score between 0 and 12 was achieved by multiplication of the staining area and intensity scores. A staining index was used in which ≤4 was considered low expression and >4 was considered high expression. This study was approved from the Institutional Review Board of the Ethics Committee of National Cancer Center/Cancer Hospital, Chinese Academy of Medical Sciences and Peking Union Medical College (No. NCC1783) and performed in accordance with the guidelines of the Declaration of Helsinki.

Dual-luciferase reporter assay

First, the 3'UTR of *IGFBP3* mRNA bound to PURα was inserted into the psiCHECK™-2 vector (Promega, Madison, WI, USA) using the XhoI and NotI (New England Biolabs) restriction endonuclease to construct the P2 reporter. Moreover, specific sites bound to PURα were also mutated to generated mutation reporter P2-M1, P2-M2 and P2-M3 separately. Cells were plated into 24-well plates at a density of 5×10^4 cells per well, and the reporters and siRNAs targeting *PURA* were transfected into each well, respectively. After 48 h of transfection, luciferase activity was measured using a Dual-Luciferase Reporter Assay System (Promega) following the manufacturer's protocol. The Renilla luciferase data were normalized to the firefly luciferase data. All experiments were performed in triplicate, and the results are

presented as the mean ± SEM.

Xenografts

For the *in vivo* tumorigenesis assay, a total of 24 five-week-old female BALB/c nude mice were purchased from Beijing Huafukang Bioscience (Beijing, China), and the mice were allocated to different experimental groups based on randomization. A total of 1×10^6 negative control (NC), knockout (KO), KO+negative control lentivirus (LV) and KO+ PURα lentivirus (LV-PURα) cells were suspended in 100 μl of PBS separately and subcutaneously implanted into the back flanks of nude mice (each group, $n = 6$). The tumor volume was calculated according to $0.5\times\text{length}\times\text{width}^2$ every three days after injection. Seventeen days after injection, all mice were euthanized, and the xenograft tumors were excised and weighed. All experimental procedures were approved by the Institutional Animal Care and Use Committee (IACUC) of the Cancer Hospital of the Chinese Academy of Medical Sciences.

References

1. Rädle B, Rutkowski AJ, Ruzsics Z, Friedel CC, Koszinowski UH, Dölken L. Metabolic labeling of newly transcribed RNA for high resolution gene expression profiling of RNA synthesis, processing and decay in cell culture. ***J Vis Exp.*** 2013 Aug 8;(78):50195. doi: 10.3791/50195.
2. Faye MD, Graber TE, Holcik M. Assessment of selective mRNA translation in mammalian cells by polysome profiling. ***J Vis Exp.*** 2014 Oct 28;(92):e52295. doi: 10.3791/52295.
3. Gao J, Tian L, Sun Y, Li W, Zhao L, Sun Y, Jing Z, Zhou L, Liu F, Zhao X. PURα mediates epithelial-mesenchymal transition to promote esophageal squamous cell carcinoma progression by regulating Snail2. ***Cancer Lett.*** 2021 Feb 1;498:98-110. doi: 10.1016/j.canlet.2020.10.030.
4. Han G, Wu Z, Zhao N, Zhou L, Liu F, Niu F, Xu Y, Zhao X. Overexpression of stathmin plays a pivotal role in the metastasis of esophageal squamous cell carcinoma. ***Oncotarget.*** 2017 Jun 27;8(37):61742-61760. doi: 10.18632/oncotarget.18687.
5. Hao XP, Pretlow TG, Rao JS, Pretlow TP. Beta-catenin expression is altered in human colonic aberrant crypt foci. ***Cancer Res.*** 2001 Nov 15;61(22):8085-8.

Supplementary Figures

Figure S1

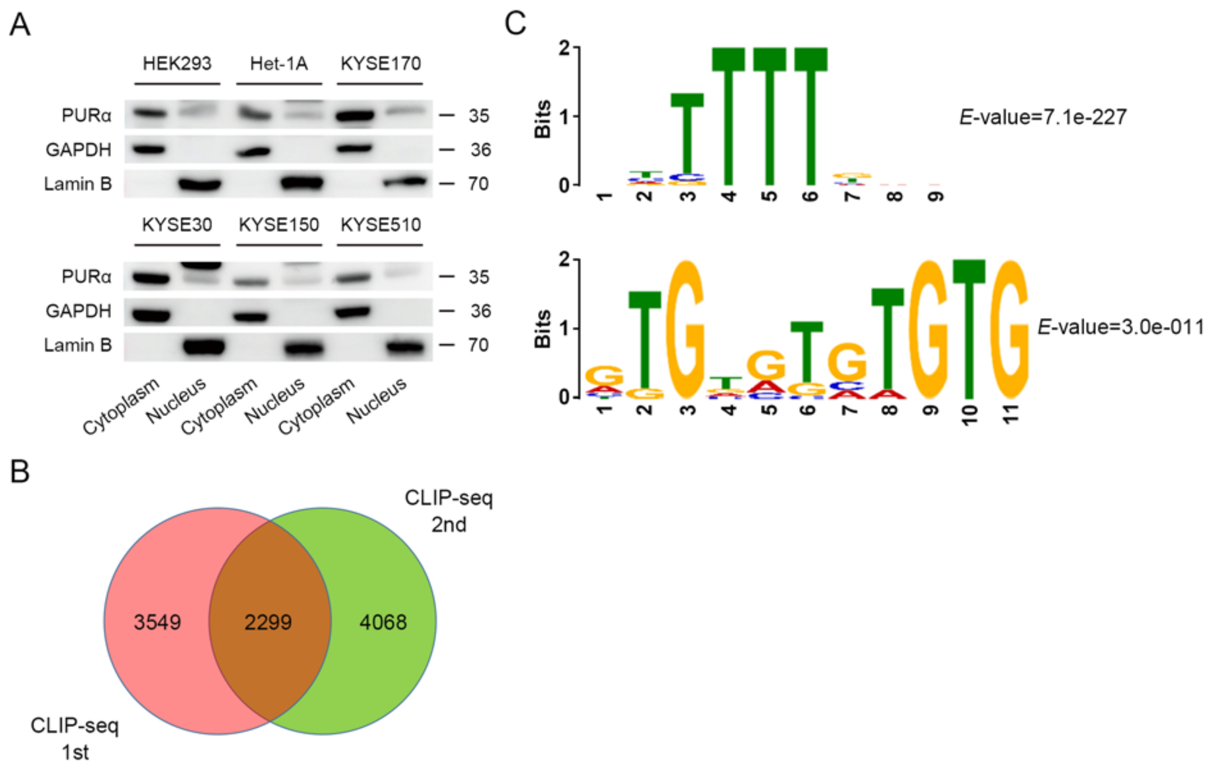

Figure S1 PURα preferentially binds UG-/U-rich motifs. **A** Protein fractionation analysis was performed to assess the distribution of endogenous PURα in HEK293, Het1A, KYSE170, KSYE30, KYSE150 and KYSE510 cells, respectively. **B** Venn diagram of the specific peaks bound to PURα in both PURα CLIP-seq datasets. **C** Top two motifs of the PURα binding sequences based on the PIPE-CLIP analysis of the crosslinking-induced mutations.

Figure S2

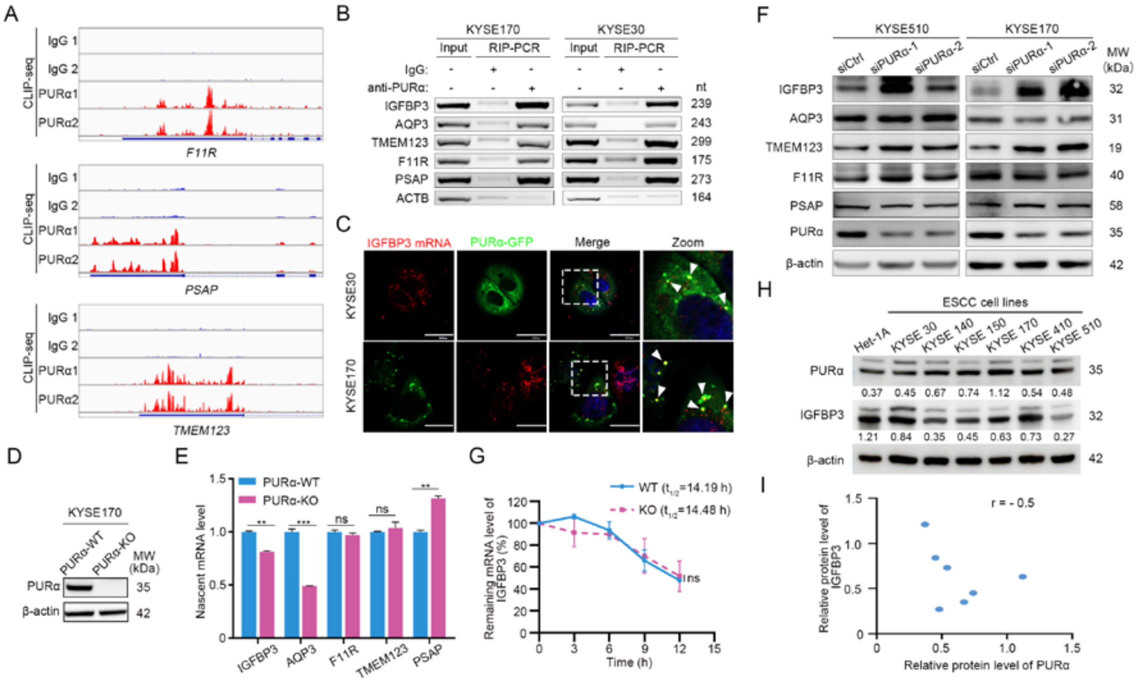

Figure S2 PURα inhibits IGFBP3 protein expression by binding to its 3'UTR. **A** Genome browser views of specific peaks bound to PURα from CLIP-seq were visualized with IGV software. The y axis indicates the reads per million (RPM) value for the highest peak within the genome browser field for each CLIP-seq run. Annotated RefSeq gene structures are shown in blue, with thin horizontal lines indicating introns and thicker blocks indicating exons. **B** A RIP assay followed by qPCR was performed to determine the interaction between PURα and mRNA of candidate genes in KYSE30 and KYSE170 cells separately. The results are representative of at least three independent experiments. ACTB, a negative control; nt: nucleotide. **C** The interaction between PURα and the IGFBP3 mRNA 3'UTR was visualized using an RNA FISH assay in KYSE30 and KYSE170 cells separately. Scale bars: 30 μm. **D** The efficiency of PURα knockout in KYSE170 cells was verified by western blot analysis. ß-actin, a loading control. **E** The nascent mRNA expression levels of candidate genes were assessed by detecting newly transcribed RNAs labeled with 4sU in wild-type and PURα-deficient KYSE170 cells separately.

Representative data are presented as the mean ± SEM from three independent experiments. **F** The protein expression level of candidate genes were assessed by western blotting when the expression of PURα was transiently knocked down by siRNA (Si-1 and Si-2) in KYSE170 and KYSE510 cell lines. siCtrl, scrambled siRNA controls. **G** The effect of PURα on the mRNA stability of IGFBP3 was detected in PURα-wild type (WT) and PURα-deficient (KO) KYSE170 . The cells were treated with actinomycin D (ActD) for 0, 3, 6, 9 and 12 hours, and the mRNA level of IGFBP3 was determined by qPCR and normalized to the ACTB mRNA level. **H** The protein levels of PURα and IGFBP3 in ESCC cell lines were examined by western blotting, respectively. The ratio under each lane indicates that PURα and IGFBP3 expression was normalized to β-actin expression. **I** The Spearman correlation coefficient was computed based on the relative protein level of PURα and IGFBP3 in ESCC cell lines.

Figure S3

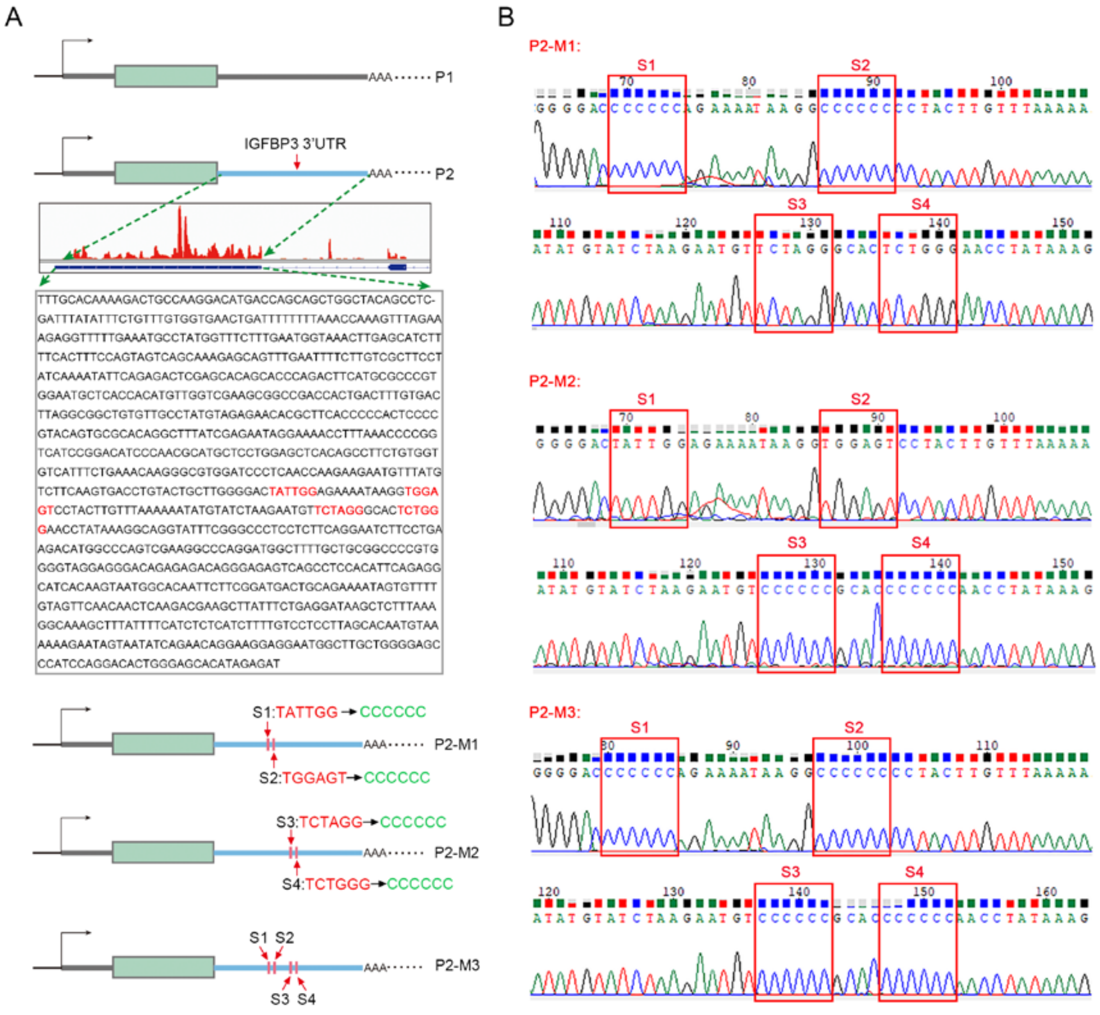

Figure S3 The sequence of IGFBP3 3'UTR and mutation site was showed in psiCHECK™-2 luciferase reporter. A Schematic diagram of the psiCHECK™-2 luciferase reporter constructs containing the vector sequence (P1) or 3'UTR of IGFBP3 mRNA (P2). The green arrows indicate the sequence of the IGFBP3 mRNA 3'UTR that was chosen and cloned according to CLIP-seq (bottom panel). The red arrows indicate mutation site bound to PURα in IGFBP3 mRNA 3'UTR. **B** The sequence of mutation site in psiCHECK™-2 luciferase reporter was showed separately.

Figure S4

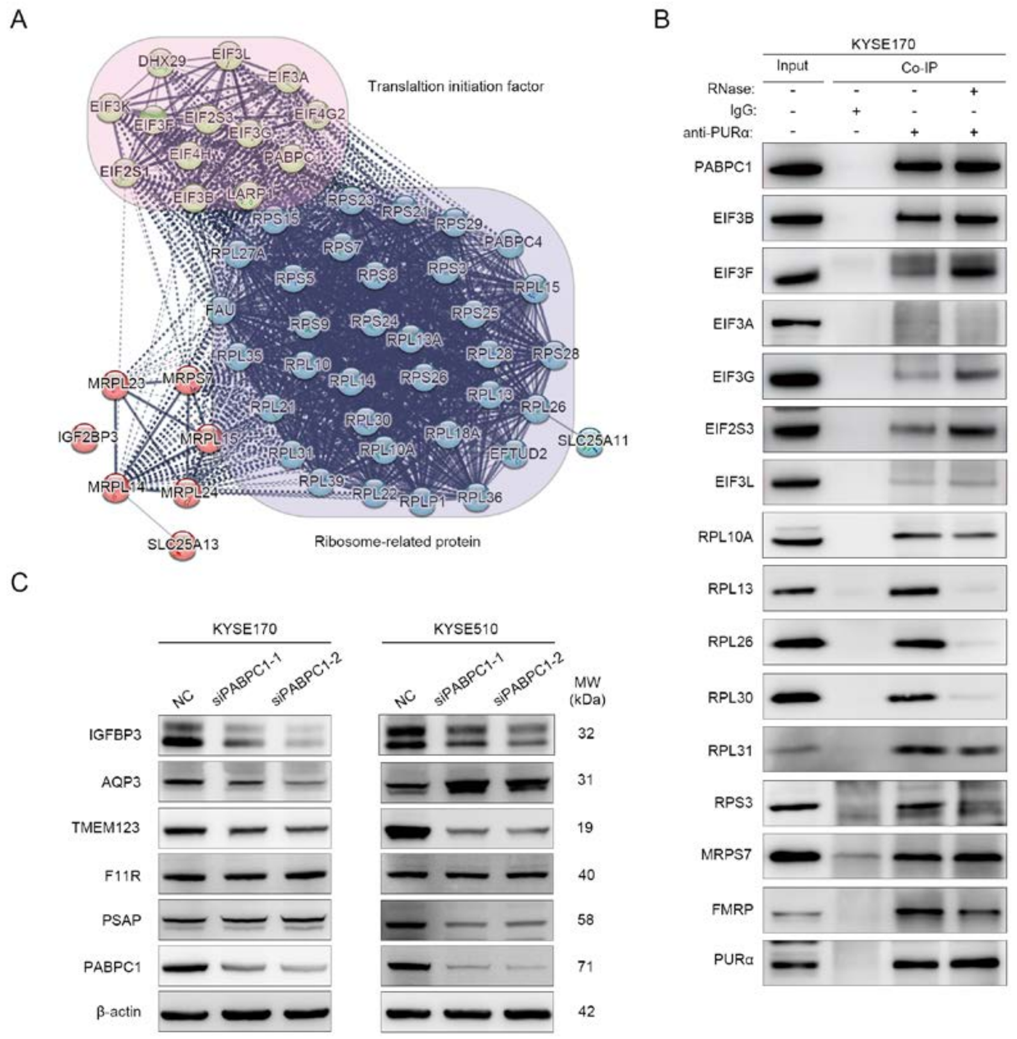

Figure S4 PURα-interacting proteins are implicated in mRNA translation. A An interaction network of mRNA translation-associated proteins interacted with PURα was constructed and clustered with the Search Tool for the Retrieval of Interacting Genes/Proteins (STRING). **B** The interactions between PURα and some representative proteins associated with mRNA translation were verified by coimmunoprecipitation in KYSE170. **C** The protein level of candidate genes were assessed via western blotting after knockdown of PABPC1 by siRNA in KYSE170 and KYSE510, respectively.

Figure S5

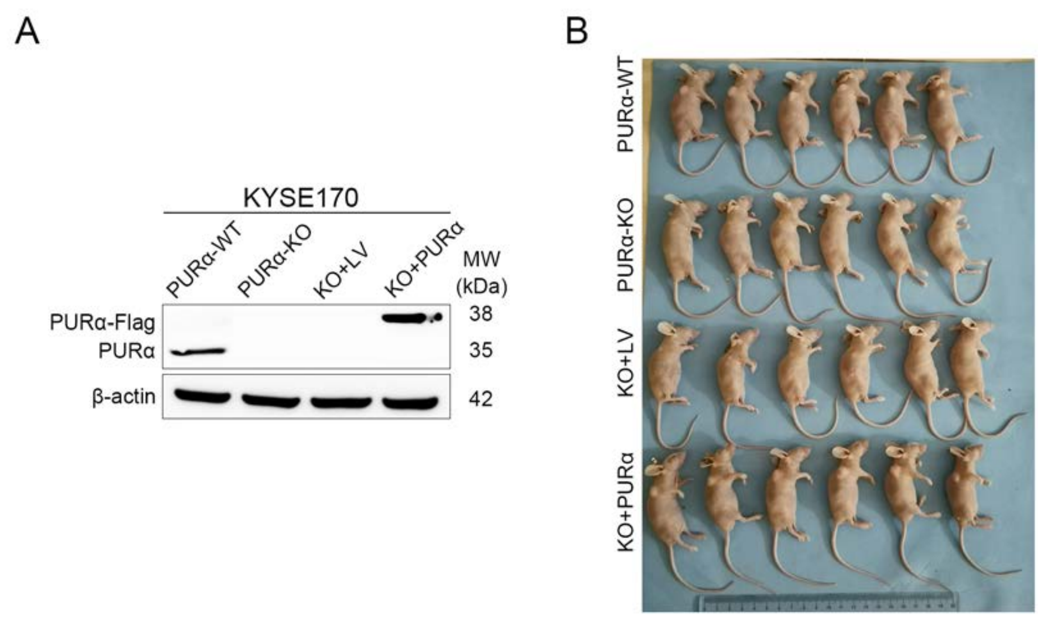

Figure S5 Knockout of PURα markedly inhibits ESCC progression *in vivo*. PURα-deficient KYSE170 cells were stably transfected with a lentiviral vector containing PURα (LV-PURα) or a negative control lentiviral vector (LV). **A**.The protein levels of PURα were assessed in wild-type (WT), PURα-deficient (KO), KO-LV and KO-LV PURα cells by western blotting, respectively. **B** WT, KO, KO+LV and KO+LV-PURα cells were injected subcutaneously into nude mice (n = 6) respectively. The mice were euthanized on 17 days after injection, and tumor formation in the mice is shown.

Supplementary Tables

Table S1 Correlations between PURα expression and clinicopathological features in ESCC patients

	PURα expression levels		p
	High (n = 296)	Low (n = 230)	
Sex			0.9636
Male/female	213/83	170/59	
Age			0.7990
<65/≥65 years	157/137	124/106	
Differentiation			0.4446
Good/Moderate/Poor	63/39/13	51/38/10	
Depth of the tumors			0.1845
T1+T2/T3+T4	205/85	175/43	
Lymph node metastasis			0.1011
Negative/N1-N3	193/102	107/119	

Table S2 Description of high-quality reads in CLIP-seq

Sample	Raw reads	Clean reads	Total mapped reads	Uniquely mapped reads	Uniquely mapped reads without duplication
IgG_1st	14549964	2957460	1786695	460155	213623
IgG_2nd	28898262	4610051	2994447	578640	250351
PURα_1st	38557102	17000812	13986960	5667437	2783683
PURα_2nd	34409870	15231429	12721579	4652791	2280046

The raw reads value includes the total reads yielded by high-throughput sequencing; the clean reads value refers to the high-quality reads after removal of self-connected adapters and low-quality bases and reads; the total mapped reads refers to the clean reads that accurately mapped to the human reference genome (hg38); the uniquely mapped reads are the clean reads that were uniquely mapped to one genomic location; and the uniquely mapped reads without duplication are the uniquely mapped reads minus the reads that were duplicated as a result of PCR.

Table S3 Description of uniquely mapped reads to the reference genome in CLIP-seq

Sample	5'UTR	3'UTR	CDS	Nc_exon	Introns	Intergenic	Antisense
IgG_1st	3245 (1.52%)	27858 (13.04%)	49483 (23.16%)	10543 (4.94%)	71868 (33.64%)	22851 (10.7%)	27775 (13.0%)
IgG_2nd	4643 (1.85%)	33371 (13.33%)	59947 (23.95%)	13268 (5.3%)	78525 (31.37%)	24182 (9.66%)	36415 (14.55%)
PURα_1st	16495 (0.59%)	261436 (9.39%)	198603 (7.13%)	66543 (2.39%)	1753081 (62.98%)	292566 (10.51%)	194959 (7%)
PURα_2nd	16075 (0.71%)	262379 (11.51%)	190451 (8.35%)	66758 (2.93%)	1310632 (57.48%)	236549 (10.37%)	197202 (8.65%)

The distribution of uniquely mapped reads without duplication on the reference genome was mainly divided into seven regions, including the 5'UTR, 3'UTR, CDS, nc_exon, introns, intergenic regions and antisense strand.

Table S6 Related resource information.

Antibodies	Source	Catalog number and identifier
PURA for WB, HIC & IF	Abcam, Waltham, MA	ab79936, RRID: AB_2253242
PURA for IP	Abcam	ab125200, RRID: AB_10973560
IGFBP3	Abcam	ab193910
AQP3	Abcam	ab125219, RRID: AB_11000698
PABPC1	Abcam	ab21060, RRID: AB_777008
PABPC1	Proteintech, Rosemont, IL	66809-1-Ig, RRID:AB_2882152
TMEM123	Abclonal, Wuhan, China	A17612, RRID: AB_2770635
F11R	Abclonal	A1241, RRID: AB_2759253
PSAP	Abclonal	A1819, RRID: AB_2763856
RPL10A	ABclonal	A5925, RRID: AB_2766664
RPL13	ABclonal	A6723, RRID: AB_2767307
RPL31	ABclonal	A17527, RRID: AB_2772081
RPL30	ABclonal	A13690, RRID: AB_2760550
RPS3	ABclonal	A4872, RRID: AB_2863369
RPL26	ABclonal	A16680, RRID: AB_2772076
MRPS7	ABclonal	A17150, RRID: AB_2770441
EIF3A	ABclonal	A0573, RRID: AB_2757272
EIF3B	ABclonal	A10259, RRID: AB_2757786
EIF3F	ABclonal	A7023, RRID: AB_2767579
EIF3G	ABclonal	A4240, RRID: AB_2765578
EIF3L	ABclonal	A9972, RRID: AB_2769294
EIF2S3	ABclonal	A6581, RRID: AB_2767174
FMRP	ABclonal	A6092, RRID: AB_2766744
EIF3B	Santa Cruz Biotech., CA	sc-137214, RRID:AB_2277705
β-Actin	Sigma-Aldrich, St. Louis, MO	A5316, RRID: AB_476743
Flag	MBL, Sakae Naka-ku Nagoya, Japan	M185-3L, RRID:AB_11123930
HA	CST, Danvers, MA	3724s, RRID:AB_1549585
HRP-conjugated mouse secondary antibody	CST	58802, RRID:AB_2799549
HRP-conjugated rabbit secondary antibody	CST	93702, RRID:AB_2800208

WB, western blot analysis; IHC, immunohistochemistry; IF, immunofluorescence taining; IP, immunoprecipitation; MBL, Medical & Biological Laboratories; CST, Cell Signaling Technology; RRID, Research Resource Identifier (https://scicrunch.org/resources)

Primer sequences and oligonucleotides
IGFBP3 qPCR primer, Forward: CGCGCCAGGAAATGCTAGTG
IGFBP3 qPCR primer, Reverse: TTTGTAGCGCTGGCTGTCTT
AQP3 qPCR primer, Forward: ACCTACCCCTCTGGACACTT
AQP3 qPCR primer, Reverse: GGACGGGGTTGTTGTAGGG
TMEM123 qPCR primer, Forward: TCTGGGCTTCCACACAACTC
TMEM123 qPCR primer, Reverse: AGGTTTCATGGTGGTGACCG
F11R qPCR primer, Forward: GTGCCTACTCGGGCTTTTCT
F11R qPCR primer, Reverse: AAGGTCACCCGGTCCTCATA
PSAP qPCR primer, Forward: TGATGCACATGCAACCCAAG
PSAP qPCR primer, Reverse: GACTTTGCTGGGACCTCGTG
ACTB qPCR primer, Forward: CATGTACGTTGCTATCCAGGC
ACTB qPCR primer, Reverse:CTCCTTAATGTCACGCACGAT
IGFBP3 RIP-PCR primer, Forward: GCTGGCTACAGCCTCGATT
IGFBP3 RIP-PCR primer, Reverse: CATGTGGTGAGCATTCCACG
AQP3 RIP-PCR primer, Forward: TGGACCTTGCCCAAATAGCA
AQP3 RIP-PCR primer, Reverse: GTCTCTGGGCTCCCCCAATA
TMEM123 RIP-PCR primer, Forward: AAGACAGAATGCCATCTGGGC
TMEM123 RIP-PCR primer, Reverse: GATCCCTAACAGGGCTAAGCA
F11R RIP-PCR primer, Forward: AATCTGCACTCAACTGCCCA
F11R RIP-PCR primer, Reverse: AGATGGAAGGACCCCAGTGT
PSAP RIP-PCR primer, Forward: CCTTGTTCTGAGCCCTGACAT
PSAP RIP-PCR primer, Reverse: TGAACCAGGGACAGAGAACC
ACTB RIP-PCR primer, Forward: CTCACCATGGATGATGATATCGC
ACTB RIP-PCR primer, Reverse: TAGGAATCCTTCTGACCCATGC
siRNA negative control sense (5'-3'):UUCUCCGAAGGUGUCACGUTT
siRNA negative control antisense (5'-3'):ACGUGACACGUUCGGAGAATT
PURα silencing siRNA -1 sense (5'-3'): GCGAGAACCGCAAGUACUATT
PURα silencing siRNA -1 antisense (5'-3'): UAGUACUUGCGGUUCUCGCTT
PURα silencing siRNA -2 sense (5'-3'): CCACCUAUCGCAACUCCAUTT
PURα silencing siRNA -2 antisense (5'-3'): AUGGAGUUGCGAUAGGUGGTT
PABPC1 silencing siRNA -1 sense (5'-3'): GCUCCUAAAUGAUCGCAAATT
PABPC1 silencing siRNA -1 antisense (5'-3'): UUUGCGAUCAUUUAGGAGCTT
PABPC1 silencing siRNA -1 sense (5'-3'): GACCACCAUUUAGUACUAUTT
PABPC1 silencing siRNA -1 antisense (5'-3'): AUAGUACUAAAUGGUGGUCTT
IGFBP3 silencing siRNA -1 sense (5'-3'): GCCAGGAAAUGCUAGUGAGTT
IGFBP3 silencing siRNA -1 antisense (5'-3'): CUCACUAGCAUUUCCUGGCTT
IGFBP3 silencing siRNA -2 sense (5'-3'): UCAAGAAAGGGCAUGCUAATT
IGFBP3 silencing siRNA -2 antisense (5'-3'): UUAGCAUGCCCUUUCUUGATT

**Primer sequences and oligonucleotides**
Stellaris RNA FISH probe, human IGFBP3 3'UTR_1: ATTAACCTTGCGGCAGGC
Stellaris RNA FISH probe, human IGFBP3 3'UTR_2: AGGCATATTTGAGCTCCA
Stellaris RNA FISH probe, human IGFBP3 3'UTR_3: TGGCAGTCTTTTGTGCAA
Stellaris RNA FISH probe, human IGFBP3 3'UTR_4: CTCTTTGCTGACTACTGG
Stellaris RNA FISH probe, human IGFBP3 3'UTR_5: TGAAGTCTGGGTGCTGTG
Stellaris RNA FISH probe, human IGFBP3 3'UTR_6: GTGAGCATTCCACGGGCG
Stellaris RNA FISH probe, human IGFBP3 3'UTR_7: CCGCTTCGACCAACATGT
Stellaris RNA FISH probe, human IGFBP3 3'UTR_8: GTCACAAAGTCAGTGGTC
Stellaris RNA FISH probe, human IGFBP3 3'UTR_9: CATAGGCAACACAGCCGC
Stellaris RNA FISH probe, human IGFBP3 3'UTR_10: CGATAAAGCCTGTGCGCA
Stellaris RNA FISH probe, human IGFBP3 3'UTR_11: GACCGGGGTTTAAAGGTT
Stellaris RNA FISH probe, human IGFBP3 3'UTR_12: AGCATGCGTTGGGATGTC
Stellaris RNA FISH probe, human IGFBP3 3'UTR_13: GAAGGCTGTGAGCTCCAG
Stellaris RNA FISH probe, human IGFBP3 3'UTR_14: TCTTGGTTGAGGGATCCA
Stellaris RNA FISH probe, human IGFBP3 3'UTR_15: ATAGTCCCCAAGCAGTAC
Stellaris RNA FISH probe, human IGFBP3 3'UTR_16: CTCCACCTTATTTTCTCC
Stellaris RNA FISH probe, human IGFBP3 3'UTR_17: CCTTTATAGGTTCCCAGA
Stellaris RNA FISH probe, human IGFBP3 3'UTR_18**:** ACTGGGCCATGTCTTCAG
Stellaris RNA FISH probe, human IGFBP3 3'UTR_19: CCTCTGAATGTGGAGGCT
Stellaris RNA FISH probe, human IGFBP3 3'UTR_20: TTGTGCCATTACTTGTGA
Stellaris RNA FISH probe, human IGFBP3 3'UTR_21: TTTTCTGCAGTCATCCGA
Stellaris RNA FISH probe, human IGFBP3 3'UTR_22: GCTTCGTCTTGAGTTGTT
Stellaris RNA FISH probe, human IGFBP3 3'UTR_23: CTCCTTCCTGTTCTGATA
Stellaris RNA FISH probe, human IGFBP3 3'UTR_24: AGTGTCCTGGATGGGCTC
Stellaris RNA FISH probe, human IGFBP3 3'UTR_25: GGGGCTATGTTGTATACA
Stellaris RNA FISH probe, human IGFBP3 3'UTR_26: ATGGCCACAGTTGTATCA
Stellaris RNA FISH probe, human IGFBP3 3'UTR_27: GTGAGCTCCTTTCCTCAG
Stellaris RNA FISH probe, human IGFBP3 3'UTR_28: AGAGCAGCCCAGTCTCTG
Stellaris RNA FISH probe, human IGFBP3 3'UTR_29: CTTCTTGGGTTTGGCCTC
Stellaris RNA FISH probe, human IGFBP3 3'UTR_30: TGAGCCTGACTTTGCCAG
Stellaris RNA FISH probe, human IGFBP3 3'UTR_31: CAGCAGGGCAGAGTCTCC
Stellaris RNA FISH probe, human IGFBP3 3'UTR_32: GTGTCCACACCGAGGTCT
Stellaris RNA FISH probe, human IGFBP3 3'UTR_33: GGAGAGCTCTATGCAGCG
Stellaris RNA FISH probe, human IGFBP3 3'UTR_34: GAGACCCCTCTGTTTTCA
Stellaris RNA FISH probe, human IGFBP3 3'UTR_35: TAGGTAGGCAGAATGTCT
