# Supplementary material for: Forming cytoplasmic stress granules PURα suppresses mRNA translation initiation of IGFBP3 to promote esophageal squamous cell carcinoma progression
Source: Oncogene. 2022 Aug 9;41(38):4336–48. doi: 10.1038/s41388-022-02426-3 (PMC9481463; doi:10.1038/s41388-022-02426-3)
Supplement: Supplementary file 2 — Supplementary Table S4 [file 41388_2022_2426_MOESM2_ESM.pdf]

Supplementary Table S4_Specific Peaks bound to PURa in CLIP-seq_PURa vs IgG

Specific peaks bound to PURα in CLIP-seq

Chr	Start	End	Tags	Strand	Length	Gene	Description	Genetype
chr7	45912779	45913715	337	-	1056	IGFBP3	insulin like growth factor binding protein 3	mRNA
chr7	45913716	45913834	337	-	1056	IGFBP3	insulin like growth factor binding protein 3	mRNA
chr9	33441189	33441994	259	-	1023	AQP3	aquaporin 3 (Gill blood group)	mRNA
chr8	128041242	128041957	258	+	739	PVT1		lncRNA
chr11	102396665	102396968	222	-	824	TMEM123	transmembrane protein 123	mRNA
chr11	102396970	102397353	222	-	824	TMEM123	transmembrane protein 123	mRNA
chr11	102397354	102397485	222	-	824	TMEM123	transmembrane protein 123	mRNA
chr8	128040611	128040908	220	+	609	PVT1		lncRNA
chr8	128040918	128041217	220	+	609	PVT1		lncRNA
chr9	129339500	129339747	219	+	1037	LINC01503		lncRNA
chr9	129339749	129340059	219	+	1037	LINC01503		lncRNA
chr9	129340060	129340299	219	+	1037	LINC01503		lncRNA
chr9	129340336	129340527	219	+	1037	LINC01503		lncRNA
chr1	109100164	109100622	214	+	502	SCARNA2		scaRNA
chr17	41518466	41518867	189	-	674	KRT15	keratin 15	mRNA
chr1	209431509	209432191	165	+	685	MIR205HG		lncRNA
chr8	128042245	128042560	157	+	764	PVT1		lncRNA
chr8	128039608	128039974	154	+	644	PVT1		lncRNA
chr1	160997625	160997963	135	-	340	F11R	F11 receptor	mRNA
chr1	43596959	43597330	133	+	588	PTPRF	protein tyrosine phosphatase receptor type F	mRNA
chr10	71816296	71816566	133	-	628	PSAP	prosaposin	mRNA
chr10	71816567	71816923	133	-	628	PSAP	prosaposin	mRNA
chr3	195746895	195747207	130	-	580	MUC4	mucin 4, cell surface associated	mRNA
chr3	195747235	195747380	130	-	580	MUC4	mucin 4, cell surface associated	mRNA
chr6	7583435	7583518	128	+	878	DSP	desmoplakin	mRNA
chr11	70421914	70422157	123	+	744	CTTN	cortactin	mRNA
chr11	70422158	70422290	123	+	744	CTTN	cortactin	mRNA
chr11	70422291	70422577	123	+	744	CTTN	cortactin	mRNA
chr20	37518009	37518190	120	-	719	BLCAP	BLCAP apoptosis inducing factor	mRNA
chr20	37518328	37518512	120	-	719	BLCAP	BLCAP apoptosis inducing factor	mRNA
chr1	28508829	28509037	117	+	520	SNHG3		lncRNA
chr1	28509075	28509216	117	+	520	SNHG3		lncRNA
chr1	28509231	28509324	117	+	520	SNHG3		lncRNA
chr6	122444259	122444570	116	-	344	SERINC1	serine incorporator 1	mRNA
chr8	128033139	128033446	113	+	673	PVT1		lncRNA
chr9	129340537	129340785	107	+	479	LINC01503		lncRNA
chr9	129340810	129341015	107	+	479	LINC01503		lncRNA
chr1	205713772	205713943	106	-	679	NUCKS1	nuclear casein kinase and cyclin-dependent kinase substrate 1	mRNA
chr1	205714103	205714217	106	-	679	NUCKS1	nuclear casein kinase and cyclin-dependent kinase substrate 1	mRNA
chr1	205714344	205714436	106	-	679	NUCKS1	nuclear casein kinase and cyclin-dependent kinase substrate 1	mRNA
chr8	11844384	11844428	103	-	676	CTSB	cathepsin B	mRNA
chr8	11844429	11844637	103	-	676	CTSB	cathepsin B	mRNA
chr8	11844710	11844978	103	-	676	CTSB	cathepsin B	mRNA
chr8	11844991	11845059	103	-	676	CTSB	cathepsin B	mRNA
chr5	882730	883050	100	-	854	BRD9	bromodomain containing 9	mRNA
chr8	119245265	119245390	98	+	388	MAL2	mal, T cell differentiation protein 2	mRNA
chr8	119245393	119245652	98	+	388	MAL2	mal, T cell differentiation protein 2	mRNA
chr17	59839906	59840184	97	+	490	VMP1	vacuole membrane protein 1	mRNA
chr12	6346817	6347300	97	-	509	SCNN1A	sodium channel epithelial 1 alpha subunit	mRNA
chr11	70420336	70420615	96	+	446	CTTN	cortactin	mRNA
chr1	161072130	161072569	93	-	451	NECTIN4	nectin cell adhesion molecule 4	mRNA
chr3	196049807	196050186	93	-	659	TFRC	transferrin receptor	mRNA
chr3	196050332	196050452	93	-	659	TFRC	transferrin receptor	mRNA
chr1	160996232	160996523	92	-	357	F11R	F11 receptor	mRNA
chr1	54851750	54852055	92	-	470	DHCR24	24-dehydrocholesterol reductase	mRNA
chr8	97851472	97851593	91	+	436	LAPTM4B	lysosomal protein transmembrane 4 beta	mRNA
chr8	97851599	97851831	91	+	436	LAPTM4B	lysosomal protein transmembrane 4 beta	mRNA
chr11	70408269	70408361	89	+	449	CTTN	cortactin	mRNA
chr12	49762872	49763049	89	+	412	TMBIM6	transmembrane BAX inhibitor motif containing 6	mRNA
chr12	49763057	49763174	89	+	412	TMBIM6	transmembrane BAX inhibitor motif containing 6	mRNA
chr16	68834529	68834670	89	+	625	CDH1	cadherin 1	mRNA
chr16	68834672	68834750	89	+	625	CDH1	cadherin 1	mRNA

chr16	68834764	68835011	89	+	625	CDH1	cadherin 1	mRNA
chr16	31187071	31187250	87	+	394	FUS	FUS RNA binding protein	mRNA
chr16	31187252	31187415	87	+	394	FUS	FUS RNA binding protein	mRNA
chr1	160998147	160998350	85	-	484	F11R	F11 receptor	mRNA
chr1	160998354	160998630	85	-	484	F11R	F11 receptor	mRNA
chr12	12916594	12916793	83	+	384	GPRC5A	G protein-coupled receptor, class C, group 5, member A	mRNA
chr12	12916794	12916939	83	+	384	GPRC5A	G protein-coupled receptor, class C, group 5, member A	mRNA
chr8	118356966	118357075	82	-	514	SAMD12	sterile alpha motif domain containing 12	mRNA
chr1	156742129	156742524	82	-	422	HDGF	heparin binding growth factor	mRNA
chr11	62516343	62516494	81	-	491	AHNAK	AHNAK nucleoprotein	mRNA
chr8	128007443	128007585	81	+	462	PVT1		lncRNA
chr8	119244794	119245048	80	+	276	MAL2	mal, T cell differentiation protein 2	mRNA
chr6	44253488	44253843	80	+	396	HSP90AB1	heat shock protein 90 alpha family class B member 1	mRNA
chr1	54850883	54851273	80	-	482	DHCR24	24-dehydrocholesterol reductase	mRNA
chr11	102398424	102398619	78	-	196	TMEM123	transmembrane protein 123	mRNA
chr17	41513764	41514125	78	-	398	KRT15	keratin 15	mRNA
chr14	102081399	102081821	78	-	435	HSP90AA1	heat shock protein 90 alpha family class A member 1	mRNA
chr7	45912383	45912576	77	-	392	IGFBP3	insulin like growth factor binding protein 3	mRNA
chr7	45912608	45912771	77	-	392	IGFBP3	insulin like growth factor binding protein 3	mRNA
chr8	119243416	119243661	76	+	353	MAL2	mal, T cell differentiation protein 2	mRNA
chr2	85320914	85321223	75	-	310	TGOLN2	trans-golgi network protein 2	mRNA
chr3	41239653	41239736	72	+	289	CTNNB1	catenin beta 1	mRNA
chr3	41239794	41239916	72	+	289	CTNNB1	catenin beta 1	mRNA
chr16	87830024	87830195	72	-	351	SLC7A5	solute carrier family 7 member 5	mRNA
chr16	87830218	87830374	72	-	351	SLC7A5	solute carrier family 7 member 5	mRNA
chr8	118042615	118042857	71	-	513	EXT1	exostosin glycosyltransferase 1	mRNA
chr12	12913264	12913619	70	+	475	GPRC5A	G protein-coupled receptor, class C, group 5, member A	mRNA
chr5	43290826	43291089	69	-	382	HMGCS1	3-hydroxy-3-methylglutaryl-CoA synthase 1	mRNA
chr1	209787225	209787488	69	-	409	IRF6	interferon regulatory factor 6	mRNA
chr6	7579501	7579830	68	+	372	DSP	desmoplakin	mRNA
chr15	98958600	98958979	67	+	406	IGF1R	insulin like growth factor 1 receptor	mRNA
chr1	153981820	153981938	67	-	406	RAB13	RAB13, member RAS oncogene family	mRNA
chr1	153981939	153982078	67	-	406	RAB13	RAB13, member RAS oncogene family	mRNA
chr1	173866609	173866713	66	-	188	SNORD76		snoRNA
chr5	151663074	151663177	66	-	440	SPARC	secreted protein acidic and cysteine rich	mRNA
chr5	151663180	151663352	66	-	440	SPARC	secreted protein acidic and cysteine rich	mRNA
chr5	151663377	151663419	66	-	440	SPARC	secreted protein acidic and cysteine rich	mRNA
chr17	59838854	59839031	64	+	320	VMP1	vacuole membrane protein 1	mRNA
chr8	128034910	128035140	64	+	367	PVT1		lncRNA
chr19	46774908	46775179	64	-	273	SLC1A5	solute carrier family 1 member 5	mRNA
chr14	39180764	39180962	63	+	439	PNN	pinin, desmosome associated protein	mRNA
chr11	62516716	62516755	62	-	397	AHNAK	AHNAK nucleoprotein	mRNA
chr11	62516767	62517112	62	-	397	AHNAK	AHNAK nucleoprotein	mRNA
chr14	102050627	102050775	62	+	149	DYNC1H1	dynein cytoplasmic 1 heavy chain 1	mRNA
chr2	47386571	47386728	62	+	158	EPCAM	epithelial cell adhesion molecule	mRNA
chr12	12916963	12917236	62	+	274	GPRC5A	G protein-coupled receptor, class C, group 5, member A	mRNA
chr17	1344791	1345028	62	-	297	YWHAE	tyrosine 3-monooxygenase/tryptophan 5-monooxygenase activation protein eps	mRNA
chr17	41515487	41515692	61	-	349	KRT15	keratin 15	mRNA
chr19	40939166	40939238	61	+	388	CYP2B7P		pseudo
chr19	40939390	40939523	61	+	388	CYP2B7P		pseudo
chr6	109368075	109368308	61	-	250	CD164	CD164 molecule	mRNA
chr11	62624831	62624903	60	-	290	GANAB	glucosidase II alpha subunit	mRNA
chr12	49764431	49764610	60	+	313	TMBIM6	transmembrane BAX inhibitor motif containing 6	mRNA
chr12	49764618	49764721	60	+	313	TMBIM6	transmembrane BAX inhibitor motif containing 6	mRNA
chr11	57817005	57817345	60	+	511	CTNND1	catenin delta 1	mRNA
chr11	71484619	71484925	59	+	307	NADSYN1	NAD synthetase 1	mRNA
chr6	106971336	106971533	59	-	202	CD24	CD24 molecule	mRNA
chr1	116404440	116404749	59	+	360	ATP1A1	ATPase Na+/K+ transporting subunit alpha 1	mRNA
chr2	85544544	85544725	59	+	402	MAT2A	methionine adenosyltransferase 2A	mRNA
chr5	179705678	179705852	58	+	175	CANX	calnexin	mRNA
chr3	105574715	105574836	58	+	338	ALCAM	activated leukocyte cell adhesion molecule	mRNA
chr1	205715664	205715958	58	-	394	NUCKS1	nuclear casein kinase and cyclin-dependent kinase substrate 1	mRNA
chr8	11844018	11844167	57	-	518	CTSB	cathepsin B	mRNA

chr8	128054653	128054801	57	+	366	PVT1		lncRNA
chr12	6510293	6510570	57	+	350	SCARNA10		scaRNA
chr6	106970159	106970308	57	-	231	CD24	CD24 molecule	mRNA
chr10	100362423	100362627	57	+	244	SCD	stearoyl-CoA desaturase (delta-9-desaturase)	mRNA
chr2	10783908	10784118	57	-	270	PDIA6	protein disulfide isomerase family A member 6	mRNA
chr3	194405089	194405214	57	-	362	ATP13A3	ATPase 13A3	mRNA
chr8	117989580	117989853	56	-	297	EXT1	exostosin glycosyltransferase 1	mRNA
chr10	72275138	72275466	56	+	329	DDIT4	DNA-damage-inducible transcript 4	mRNA
chr11	102231071	102231232	56	+	424	YAP1	Yes associated protein 1	mRNA
chr9	93565865	93566073	55	+	209	FAM120A	family with sequence similarity 120A	mRNA
chr12	48936217	48936265	55	-	426	ARF3	ADP ribosylation factor 3	mRNA
chr12	48936294	48936423	55	-	426	ARF3	ADP ribosylation factor 3	mRNA
chr14	55672928	55673012	54	+	85	KTN1	kinectin 1	mRNA
chr8	128006793	128006955	54	+	328	PVT1		lncRNA
chr11	93721718	93721866	54	+	149	SCARNA9		scaRNA
chr17	59840952	59841166	54	+	345	VMP1	vacuole membrane protein 1	mRNA
chr3	194403688	194403866	53	-	191	ATP13A3	ATPase 13A3	mRNA
chr14	21210938	21211157	53	-	220	HNRNPC	heterogeneous nuclear ribonucleoprotein C	mRNA
chr17	58004979	58005020	53	-	313	SRSF1	serine and arginine rich splicing factor 1	mRNA
chr17	58005038	58005220	53	-	313	SRSF1	serine and arginine rich splicing factor 1	mRNA
chr20	44499259	44499317	52	-	322	SERINC3	serine incorporator 3	mRNA
chr20	44498996	44499119	52	-	322	SERINC3	serine incorporator 3	mRNA
chr9	134339817	134340099	52	+	580	RXRA	retinoid X receptor, alpha	mRNA
chr16	19119112	19119375	52	+	264	ITPRIPL2	inositol 1,4,5-trisphosphate receptor interacting protein-like 2	mRNA
chr12	12914239	12914465	52	+	290	GPRC5A	G protein-coupled receptor, class C, group 5, member A	mRNA
chr8	100918956	100919246	52	-	340	YWHAZ	tyrosine 3-monooxygenase/tryptophan 5-monooxygenase activation protein zet	mRNA
chr2	231454951	231455004	52	-	362	NCL	nucleolin	mRNA
chr16	2771149	2771306	52	+	371	SRRM2	serine/arginine repetitive matrix 2	mRNA
chr7	74196253	74196339	52	+	614	EIF4H	eukaryotic translation initiation factor 4H	mRNA
chr20	45326114	45326297	51	-	193	SDC4	syndecan 4	mRNA
chr9	129739261	129739554	51	-	294	PTGES	prostaglandin E synthase	mRNA
chr5	179728590	179728928	51	+	345	CANX	calnexin	mRNA
chr6	7584730	7584928	50	+	370	DSP	desmoplakin	mRNA
chr1	183144273	183144443	50	+	171	LAMC1	laminin, gamma 1 (formerly LAMB2)	mRNA
chr8	123014521	123014755	50	-	286	DERL1	derlin 1	mRNA
chr19	35123440	35123619	50	+	324	FXYD3	FXYD domain containing ion transport regulator 3	mRNA
chr14	75132584	75132655	50	-	325	TMED10	transmembrane p24 trafficking protein 10	mRNA
chr14	75132658	75132831	50	-	325	TMED10	transmembrane p24 trafficking protein 10	mRNA
chr19	38291927	38292059	50	+	333	SPINT2	serine peptidase inhibitor, Kunitz type 2	mRNA
chr19	38292062	38292156	50	+	333	SPINT2	serine peptidase inhibitor, Kunitz type 2	mRNA
chr20	45479013	45479225	49	+	261	WFDC2	WAP four-disulfide core domain 2	mRNA
chr20	36094149	36094211	49	+	383	EPB41L1	erythrocyte membrane protein band 4.1 like 1	mRNA
chr20	36094302	36094449	49	+	383	EPB41L1	erythrocyte membrane protein band 4.1 like 1	mRNA
chr20	36048033	36048187	49	-	227	NORAD		lncRNA
chr2	233772975	233773165	49	+	191	UGT1A8	UDP glucuronosyltransferase 1 family, polypeptide A8	mRNA
chr12	56597506	56597601	49	-	213	BAZ2A	bromodomain adjacent to zinc finger domain 2A	mRNA
chr12	56597603	56597718	49	-	213	BAZ2A	bromodomain adjacent to zinc finger domain 2A	mRNA
chr1	119913318	119913491	49	-	238	NOTCH2	notch receptor 2	mRNA
chr1	205714570	205714734	49	-	272	NUCKS1	nuclear casein kinase and cyclin-dependent kinase substrate 1	mRNA
chr17	41515884	41516010	48	-	137	KRT15	keratin 15	mRNA
chr10	32911966	32912124	48	-	159	ITGB1	integrin subunit beta 1	mRNA
chr8	118110051	118110141	48	-	282	EXT1	exostosin glycosyltransferase 1	mRNA
chr17	47682459	47682550	48	+	148	KPNB1	karyopherin subunit beta 1	mRNA
chr14	75134354	75134607	48	-	254	TMED10	transmembrane p24 trafficking protein 10	mRNA
chr2	10791794	10791925	47	-	132	PDIA6	protein disulfide isomerase family A member 6	mRNA
chr20	44498755	44498824	47	-	235	SERINC3	serine incorporator 3	mRNA
chr20	44498825	44498884	47	-	235	SERINC3	serine incorporator 3	mRNA
chr20	44498886	44498989	47	-	235	SERINC3	serine incorporator 3	mRNA
chr19	47720642	47720770	47	+	400	EHD2	EH domain containing 2	mRNA
chr14	102081031	102081398	47	-	492	HSP90AA1	heat shock protein 90 alpha family class A member 1	mRNA
chr11	10801467	10801607	46	-	150	SNORD97		snoRNA
chrX	20136064	20136135	46	-	218	SCARNA9L		scaRNA
chrX	20136155	20136267	46	-	218	SCARNA9L		scaRNA
chr13	48261307	48261482	46	+	403	ITM2B	integral membrane protein 2B	mRNA

chr13	48261528	48261637	46	+	403	ITM2B	integral membrane protein 2B	mRNA
chr1	154206946	154207114	46	-	325	C1orf43	chromosome 1 open reading frame 43	mRNA
chr7	129832390	129832634	46	-	364	UBE2H	ubiquitin conjugating enzyme E2 H	mRNA
chr11	18407320	18407826	46	+	509	LDHA	lactate dehydrogenase A	mRNA
chr8	11838558	11838778	45	+	392	FDFT1	farnesyl-diphosphate farnesyltransferase 1	mRNA
chr4	186706562	186706835	45	-	274	FAT1	FAT atypical cadherin 1	mRNA
chr13	45337197	45337320	45	-	124	TPT1	tumor protein, translationally-controlled 1	mRNA
chr8	117979481	117979527	45	-	242	EXT1	exostosin glycosyltransferase 1	mRNA
chr20	49634157	49634347	45	-	191	B4GALT5	UDP-Gal:betaGlcNAc beta 1,4- galactosyltransferase, polypeptide 5	mRNA
chr6	30899889	30900129	45	+	241	DDR1	discoidin domain receptor tyrosine kinase 1	mRNA
chr19	3981338	3981452	44	-	115	EEF2	eukaryotic translation elongation factor 2	mRNA
chr11	102401538	102401692	44	-	155	TMEM123	transmembrane protein 123	mRNA
chr11	34076250	34076474	44	+	240	CAPRIN1	cell cycle associated protein 1	mRNA
chr11	62517725	62517927	44	-	203	AHNAK	AHNAK nucleoprotein	mRNA
chr1	12582607	12582844	44	-	415	DHRS3	dehydrogenase/reductase 3	mRNA
chr8	127999773	127999906	44	+	157	PVT1		lncRNA
chr3	108047127	108047292	44	-	176	CD47	CD47 molecule	mRNA
chr8	116846005	116846069	44	-	183	RAD21	RAD21 cohesin complex component	mRNA
chr8	116846078	116846144	44	-	183	RAD21	RAD21 cohesin complex component	mRNA
chr16	18791926	18792143	44	-	239	ARL6IP1	ADP ribosylation factor like GTPase 6 interacting protein 1	mRNA
chr7	106091017	106091220	44	-	252	SYPL1	synaptophysin like 1	mRNA
chr1	42177638	42177842	44	-	253	FOXJ3	forkhead box J3	mRNA
chr1	167789312	167789404	44	+	267	MPZL1	myelin protein zero like 1	mRNA
chr1	167789461	167789556	44	+	267	MPZL1	myelin protein zero like 1	mRNA
chr10	87754440	87754687	44	-	273	ATAD1	ATPase family AAA domain containing 1	mRNA
chr11	1752754	1753035	44	-	282	CTSD	cathepsin D	mRNA
chr11	71434470	71434714	44	-	403	DHCR7	7-dehydrocholesterol reductase	mRNA
chr11	71434817	71434872	44	-	403	DHCR7	7-dehydrocholesterol reductase	mRNA
chr2	241352202	241352330	44	+	420	SEPTIN2	septin 2	mRNA
chr1	183142618	183142763	43	+	312	LAMC1	laminin, gamma 1 (formerly LAMB2)	mRNA
chr14	92042318	92042384	43	+	241			no annotation
chr14	92042452	92042513	43	+	241			no annotation
chr1	20775523	20775667	43	-	145	HP1BP3		mRNA
chr11	62528909	62529093	43	-	189	AHNAK	AHNAK nucleoprotein	mRNA
chr8	118078847	118078956	43	-	201	EXT1	exostosin glycosyltransferase 1	mRNA
chr17	16439730	16439866	43	+	387	SNHG29		lncRNA
chr19	49497718	49497802	43	+	92	SNORD35B		snoRNA
chr1	173867653	173867744	43	-	287	SNORD74		snoRNA
chr17	16439498	16439727	43	+	387	SNORD49B		snoRNA
chr12	6967333	6967396	43	-	306	SCARNA12		scaRNA
chr12	6967446	6967606	43	-	306	SCARNA12		scaRNA
chr19	54461820	54461992	43	+	201	LENG8	leukocyte receptor cluster member 8	mRNA
chr3	160501478	160501708	43	-	232	KPNA4	karyopherin subunit alpha 4	mRNA
chr1	207794623	207794753	43	+	263	CD46	CD46 molecule	mRNA
chr3	49534632	49534730	43	+	319	DAG1	dystroglycan 1	mRNA
chr3	48467886	48467994	43	-	332	SHISA5	shisa family member 5	mRNA
chr3	48468079	48468217	43	-	332	SHISA5	shisa family member 5	mRNA
chr10	71820239	71820335	42	-	97	PSAP	prosaposin	mRNA
chr1	23693806	23693913	42	+	108	RPL11	ribosomal protein L11	mRNA
chr6	7580672	7580921	42	+	250	DSP	desmoplakin	mRNA
chr4	186707201	186707366	42	-	305	FAT1	FAT atypical cadherin 1	mRNA
chr4	186618197	186618463	42	-	368	FAT1	FAT atypical cadherin 1	mRNA
chr21	39342497	39342570	42	-	217	HMGN1	high mobility group nucleosome binding domain 1	mRNA
chr21	39342574	39342692	42	-	217	HMGN1	high mobility group nucleosome binding domain 1	mRNA
chr11	1746050	1746144	42	-	331	IFITM10	interferon induced transmembrane protein 10	mRNA
chr11	62532524	62532729	42	-	341	AHNAK	AHNAK nucleoprotein	mRNA
chr1	173864363	173864446	42	-	84	SNORD47		snoRNA
chr15	66503244	66503314	42	-	88	SNORD18A		snoRNA
chr2	231460363	231460438	42	-	100	SNORD82		snoRNA
chr20	2654211	2654286	42	+	114	SNORD110		snoRNA
chr19	40941766	40941818	42	+	273	CYP2B7P		pseudo
chr1	203740129	203740222	42	+	266	ATP2B4	ATPase plasma membrane Ca2+ transporting 4	mRNA
chr1	203740227	203740328	42	+	266	ATP2B4	ATPase plasma membrane Ca2+ transporting 4	mRNA
chr1	26864158	26864456	42	+	299	SFN	stratifin	mRNA

chr1	214645916	214646132	41	+	283	CENPF	centromere protein F	mRNA
chr11	70371325	70371521	41	+	197	PPFIA1	PTPRF interacting protein alpha 1	mRNA
chr11	62529507	62529650	41	-	244	AHNAK	AHNAK nucleoprotein	mRNA
chr1	44778389	44778459	41	+	71	SNORD38B		snoRNA
chr1	75787847	75787971	41	+	142	SNORD45A		snoRNA
chr3	195568706	195568828	41	-	131	APOD	apolipoprotein D	mRNA
chr1	207794861	207795029	41	+	173	CD46	CD46 molecule	mRNA
chr17	1345298	1345499	41	-	202	YWHAE	tyrosine 3-monooxygenase/tryptophan 5-monooxygenase activation protein eps	mRNA
chr2	85544933	85545018	41	+	205	MAT2A	methionine adenosyltransferase 2A	mRNA
chr17	40457259	40457449	41	+	210	IGFBP4	insulin-like growth factor binding protein 4	mRNA
chr17	40457450	40457468	41	+	210	IGFBP4	insulin-like growth factor binding protein 4	mRNA
chr1	205715018	205715096	41	-	210	NUCKS1	nuclear casein kinase and cyclin-dependent kinase substrate 1	mRNA
chr11	110230506	110230688	41	-	237	RDX	radixin, transcript variant 4	mRNA
chr20	3805709	3805936	41	+	271	CDC25B	cell division cycle 25B	mRNA
chr20	51600691	51600740	41	-	284	ATP9A	ATPase phospholipid transporting 9A	mRNA
chr20	51600755	51600879	41	-	284	ATP9A	ATPase phospholipid transporting 9A	mRNA
chr15	98961229	98961402	41	+	420	IGF1R	insulin like growth factor 1 receptor	mRNA
chr9	130135427	130135605	41	+	454	GPR107	G protein-coupled receptor 107	mRNA
chr9	130135779	130135880	41	+	454	GPR107	G protein-coupled receptor 107	mRNA
chr8	11845060	11845214	40	-	163	CTSB	cathepsin B	mRNA
chr14	55612303	55612389	40	+	271	KTN1	kinectin 1	mRNA
chr15	66502021	66502091	40	-	133	SNORD18B		snoRNA
chr1	171592262	171592362	40	+	228	PRRC2C	proline rich coiled-coil 2C	mRNA
chr21	25880955	25881059	40	-	230	APP	amyloid beta precursor protein	mRNA
chr21	25881060	25881160	40	-	230	APP	amyloid beta precursor protein	mRNA
chr17	35725561	35725665	40	+	247	AP2B1	adaptor related protein complex 2 subunit beta 1	mRNA
chr2	20032707	20032756	40	-	350	LAPTM4A	lysosomal protein transmembrane 4 alpha	mRNA
chr2	20032760	20033024	40	-	350	LAPTM4A	lysosomal protein transmembrane 4 alpha	mRNA
chr17	78971428	78971488	39	-	327	LGALS3BP	galectin 3 binding protein	mRNA
chr17	78971489	78971727	39	-	327	LGALS3BP	galectin 3 binding protein	mRNA
chr8	116857379	116857473	39	-	114	RAD21	RAD21 cohesin complex component	mRNA
chr5	882176	882268	39	-	213	BRD9	bromodomain containing 9	mRNA
chr1	44777841	44777910	39	+	72	SNORD38A		snoRNA
chr22	39315207	39315304	39	-	99	SNORD83A		snoRNA
chr1	173865622	173865820	39	-	199	SNORD78		snoRNA
chr11	62852906	62853016	39	-	203	SNORD22		snoRNA
chr5	151662611	151662795	39	-	185	SPARC	secreted protein acidic and cysteine rich	mRNA
chr10	96521193	96521321	39	-	195	TM9SF3	transmembrane 9 superfamily member 3	mRNA
chr4	105970432	105970560	39	+	200	NPNT	nephronectin	mRNA
chr4	105970562	105970631	39	+	200	NPNT	nephronectin	mRNA
chr22	36266405	36266606	39	+	204	APOL1	apolipoprotein L1	mRNA
chr9	111933295	111933510	39	+	217	UGCG	UDP-glucose ceramide glucosyltransferase	mRNA
chr2	47160379	47160539	39	-	225	CALM2	calmodulin 2	mRNA
chr9	128694822	128695139	39	+	318	SET	SET nuclear proto-oncogene	mRNA
chr19	583064	583082	39	+	435	BSG	basigin (Ok blood group)	mRNA
chr3	194405993	194406094	38	-	324	ATP13A3	ATPase 13A3	mRNA
chr19	48966561	48966878	38	+	318	FTL	ferritin light chain	mRNA
chr5	179614797	179614959	38	-	163	HNRNPH1	heterogeneous nuclear ribonucleoprotein H1	mRNA
chr11	62853874	62853968	38	-	96	SNORD29		snoRNA
chr1	173865966	173866041	38	-	115	SNORD44		snoRNA
chr9	69715234	69715374	38	-	142	PTAR1	protein prenyltransferase alpha subunit repeat containing 1	mRNA
chr5	151504125	151504233	38	-	157	FAT2	FAT atypical cadherin 2	mRNA
chr1	227919700	227919888	38	-	214	WNT9A	wingless-type MMTV integration site family, member 9A	mRNA
chr5	159158106	159158176	38	-	251	RNF145	ring finger protein 145	mRNA
chr5	159158195	159158356	38	-	251	RNF145	ring finger protein 145	mRNA
chr14	55629956	55630097	37	+	142	KTN1	kinectin 1	mRNA
chr11	62570964	62571108	37	-	145	EEF1G	eukaryotic translation elongation factor 1 gamma	mRNA
chr11	71437795	71437943	37	-	161	DHCR7	7-dehydrocholesterol reductase	mRNA
chr7	45917212	45917374	37	-	163	IGFBP3	insulin like growth factor binding protein 3	mRNA
chr20	44498607	44498748	37	-	142	SERINC3	serine incorporator 3	mRNA
chr8	118012788	118012887	37	-	184	EXT1	exostosin glycosyltransferase 1	mRNA
chr17	72553774	72553971	37	-	275	LINC00673		lncRNA
chr9	133350846	133350912	37	+	67	SNORD36C		snoRNA
chr1	173864813	173864905	37	-	173	SNORD80		snoRNA

chr19	40933812	40933968	37	+	317	CYP2B7P		pseudo
chr5	151663420	151663572	37	-	180	SPARC	secreted protein acidic and cysteine rich	mRNA
chr6	33203908	33204095	37	+	188	SLC39A7	solute carrier family 39 member 7	mRNA
chr17	38919803	38919943	37	+	222	LASP1	LIM and SH3 protein 1	mRNA
chr15	72199028	72199099	37	-	252	PKM	pyruvate kinase M1/2	mRNA
chr20	49634972	49635147	37	-	258	B4GALT5	UDP-Gal:betaGlcNAc beta 1,4- galactosyltransferase, polypeptide 5	mRNA
chr20	49635150	49635199	37	-	258	B4GALT5	UDP-Gal:betaGlcNAc beta 1,4- galactosyltransferase, polypeptide 5	mRNA
chr1	154584452	154584486	37	-	284	ADAR	adenosine deaminase RNA specific	mRNA
chr13	21147645	21147717	37	+	381	SAP18	Sin3A associated protein 18	mRNA
chr13	21147722	21147789	37	+	381	SAP18	Sin3A associated protein 18	mRNA
chr11	62880946	62881234	36	+	520	SLC3A2	solute carrier family 3 member 2	mRNA
chr8	117938884	117939033	36	-	156	EXT1	exostosin glycosyltransferase 1	mRNA
chr17	16439973	16440142	36	+	192	SNORD49A		snoRNA
chr1	209785855	209785909	36	-	237	IRF6	interferon regulatory factor 6	mRNA
chr5	171410621	171410683	36	+	249	NPM1	nucleophosmin 1	mRNA
chr5	171410761	171410869	36	+	249	NPM1	nucleophosmin 1	mRNA
chr17	58089788	58089934	36	+	291	DYNLL2	dynein, light chain, LC8-type 2	mRNA
chr17	58089935	58089996	36	+	291	DYNLL2	dynein, light chain, LC8-type 2	mRNA
chr14	103136378	103136501	36	+	305	TNFAIP2	TNF alpha induced protein 2	mRNA
chr6	7558115	7558249	35	+	143	DSP	desmoplakin	mRNA
chr5	181242215	181242345	35	-	238	RACK1	receptor for activated C kinase 1	mRNA
chr19	36215292	36215475	35	+	222	ZNF146	zinc finger protein 146	mRNA
chr5	181243310	181243378	35	-	69	SNORD95		snoRNA
chr9	133349395	133349470	35	+	76	SNORD24		snoRNA
chr6	132817218	132817348	35	+	151	SNORA33		snoRNA
chr3	186786674	186786852	35	+	180	SNORA81		snoRNA
chr1	28508545	28508762	35	+	269	SNORA73B		snoRNA
chr10	73917074	73917158	35	+	140	PLAU	plasminogen activator	mRNA
chr6	30493572	30493689	35	+	180	HLA-E	major histocompatibility complex, class I, E	mRNA
chr5	179614538	179614723	35	-	186	HNRNPH1	heterogeneous nuclear ribonucleoprotein H1	mRNA
chr16	18793070	18793124	35	-	196	ARL6IP1	ADP ribosylation factor like GTPase 6 interacting protein 1	mRNA
chr16	18793126	18793248	35	-	196	ARL6IP1	ADP ribosylation factor like GTPase 6 interacting protein 1	mRNA
chr19	38292205	38292366	35	+	253	SPINT2	serine peptidase inhibitor, Kunitz type 2	mRNA
chr19	38292371	38292414	35	+	253	SPINT2	serine peptidase inhibitor, Kunitz type 2	mRNA
chr19	42377801	42377980	35	+	263	MEGF8	multiple EGF like domains 8	mRNA
chr13	106559090	106559203	34	-	241	ARGLU1	arginine and glutamate rich 1	mRNA
chr11	62517929	62518198	34	-	270	AHNAK	AHNAK nucleoprotein	mRNA
chr1	173866877	173866939	34	-	63	SNORD75		snoRNA
chr1	75789446	75789552	34	+	107	SNORD45B		snoRNA
chr15	66501251	66501318	34	-	109	SNORD18C		snoRNA
chr10	5458030	5458166	34	+	150	NET1	neuroepithelial cell transforming 1	mRNA
chr1	6186293	6186470	34	-	180	RPL22	ribosomal protein L22	mRNA
chr11	130144427	130144584	34	+	184	APLP2	amyloid beta precursor like protein 2	mRNA
chr6	30626108	30626271	34	+	189	MRPS18B	mitochondrial ribosomal protein S18B	mRNA
chr18	3458116	3458275	34	+	203	TGIF1	TGFB induced factor homeobox 1	mRNA
chr1	209786167	209786325	34	-	211	IRF6	interferon regulatory factor 6	mRNA
chr22	41907087	41907280	34	+	218	SREBF2	sterol regulatory element binding transcription factor 2	mRNA
chr15	98961403	98961440	34	+	223	IGF1R	insulin like growth factor 1 receptor	mRNA
chr11	71434873	71435133	34	-	261	DHCR7	7-dehydrocholesterol reductase	mRNA
chr11	1753177	1753449	34	-	296	CTSD	cathepsin D	mRNA
chrX	16844835	16845087	34	-	316	RBBP7	RB binding protein 7, chromatin remodeling factor	mRNA
chr7	45916566	45916667	33	-	105	IGFBP3	insulin like growth factor binding protein 3	mRNA
chr18	35689202	35689290	33	+	119	GALNT1	polypeptide N-acetylgalactosaminyltransferase 1	mRNA
chr1	116398620	116398735	33	+	170	ATP1A1	ATPase Na+/K+ transporting subunit alpha 1	mRNA
chr1	116398749	116398789	33	+	170	ATP1A1	ATPase Na+/K+ transporting subunit alpha 1	mRNA
chr8	39077227	39077297	33	+	185	ADAM9	ADAM metallopeptidase domain 9	mRNA
chr8	39077304	39077411	33	+	185	ADAM9	ADAM metallopeptidase domain 9	mRNA
chr6	47005508	47005697	33	-	190	ADGRF1	adhesion G protein-coupled receptor F1	mRNA
chr19	3982506	3982573	33	-	68	SNORD37		snoRNA
chr6	132815307	132815389	33	+	92	SNORD101		snoRNA
chr22	39319050	39319113	33	-	113	SNORD43		snoRNA
chr1	75787070	75787225	33	+	156	SNORD45C		snoRNA
chr9	129738333	129738429	33	-	97	PTGES	prostaglandin E synthase	mRNA
chr8	100920401	100920539	33	-	139	YWHAZ	tyrosine 3-monooxygenase/tryptophan 5-monooxygenase activation protein zet	mRNA

chr12	6237762	6237920	33	+	174	CD9	CD9 molecule	mRNA
chr1	169131742	169131879	33	+	185	ATP1B1	ATPase Na+/K+ transporting subunit beta 1	mRNA
chr16	87830558	87830574	33	-	187	SLC7A5	solute carrier family 7 member 5	mRNA
chr12	88198204	88198258	33	+	204	TMTC3	transmembrane O-mannosyltransferase targeting cadherins 3	mRNA
chr12	88198262	88198356	33	+	204	TMTC3	transmembrane O-mannosyltransferase targeting cadherins 3	mRNA
chr3	196829487	196829741	33	+	255	PAK2	p21 (RAC1) activated kinase 2	mRNA
chr2	230818841	230818915	33	+	258	CAB39	calcium binding protein 39	mRNA
chr2	230818921	230819098	33	+	258	CAB39	calcium binding protein 39	mRNA
chr9	112221641	112221716	33	-	281	PTBP3	polypyrimidine tract binding protein 3	mRNA
chr9	112221719	112221869	33	-	281	PTBP3	polypyrimidine tract binding protein 3	mRNA
chr12	125029781	125029937	33	+	282	BRI3BP	BRI3 binding protein	mRNA
chr17	38921477	38921744	33	+	312	LASP1	LIM and SH3 protein 1	mRNA
chr1	45511036	45511167	33	-	409	PRDX1	peroxiredoxin 1	mRNA
chr1	45511173	45511414	33	-	409	PRDX1	peroxiredoxin 1	mRNA
chr22	38485482	38485522	33	-	258	DDX17	DEAD-box helicase 17	mRNA
chr11	35229128	35229407	32	+	314	CD44	CD44 molecule	mRNA
chr14	55671784	55671877	32	+	117	KTN1	kinectin 1	mRNA
chr8	39026676	39026810	32	+	152	ADAM9	ADAM metallopeptidase domain 9	mRNA
chr3	184324877	184325114	32	+	238	EIF4G1	eukaryotic translation initiation factor 4 gamma 1	mRNA
chr3	121695488	121695667	32	-	364	GOLGB1	golgin B1	mRNA
chr20	36093175	36093263	32	+	168	EPB41L1	erythrocyte membrane protein band 4.1 like 1	mRNA
chr8	118296799	118296876	32	-	240	SAMD12	sterile alpha motif domain containing 12	mRNA
chr5	181241815	181241891	32	-	77	SNORD96A		snoRNA
chr18	724125	724203	32	-	81	YES1	YES proto-oncogene 1	mRNA
chr1	235111003	235111078	32	-	125	TOMM20	translocase of outer mitochondrial membrane 20	mRNA
chr22	36266918	36267041	32	+	134	APOL1	apolipoprotein L1	mRNA
chr16	68698884	68699012	32	+	138	CDH3	cadherin 3	mRNA
chr1	183145344	183145475	32	+	164	LAMC1	laminin, gamma 1 (formerly LAMB2)	mRNA
chr17	48059835	48060026	32	+	192	NFE2L1	nuclear factor, erythroid 2 like 1	mRNA
chr15	60347338	60347492	32	-	215	ANXA2	annexin A2	mRNA
chr15	60347496	60347552	32	-	215	ANXA2	annexin A2	mRNA
chr14	64744153	64744206	32	+	227	PLEKHG3	pleckstrin homology and RhoGEF domain containing G3	mRNA
chr17	59840463	59840561	32	+	232	VMP1	vacuole membrane protein 1	mRNA
chr10	71299955	71300183	32	+	250	UNC5B	unc-5 netrin receptor B	mRNA
chr10	71300346	71300474	32	+	275	UNC5B	unc-5 netrin receptor B	mRNA
chr5	179726679	179726759	31	+	81	CANX	calnexin	mRNA
chr8	119240164	119240320	31	+	157	MAL2	mal, T cell differentiation protein 2	mRNA
chrX	53561780	53561924	31	-	170	HUWE1	HECT, UBA and WWE domain containing E3 ubiquitin protein ligase 1	mRNA
chr8	39042070	39042117	31	+	173	ADAM9	ADAM metallopeptidase domain 9	mRNA
chr3	196075240	196075360	31	-	203	TFRC	transferrin receptor	mRNA
chr11	71438941	71439083	31	-	206	DHCR7	7-dehydrocholesterol reductase	mRNA
chr9	19378707	19378918	31	-	224	RPS6	ribosomal protein S6	mRNA
chr3	49531186	49531256	31	+	378	DAG1	dystroglycan 1	mRNA
chr2	231456443	231456523	31	-	82	SNORD20		snoRNA
chr11	62854610	62854689	31	-	89	SNORD28		snoRNA
chr11	123058075	123058161	31	-	90	SNORD14E		snoRNA
chr3	39411053	39411147	31	+	154	SNORA62		snoRNA
chr3	39411155	39411206	31	+	154	SNORA62		snoRNA
chr14	49895558	49895675	31	+	138	ARF6	ADP-ribosylation factor 6	mRNA
chr1	162523153	162523239	31	+	143	UHMK1	U2AF homology motif kinase 1	mRNA
chr6	75255641	75255788	31	-	148	TMEM30A	transmembrane protein 30A	mRNA
chr17	29261371	29261468	31	-	167	NUFIP2	nuclear FMR1 interacting protein 2	mRNA
chr2	138571924	138572109	31	+	190	SPOPL	speckle type BTB/POZ protein like	mRNA
chr19	582774	582993	31	+	220	BSG	basigin (Ok blood group)	mRNA
chr16	66723203	66723370	31	-	236	DYNC1LI2	dynein cytoplasmic 1 light intermediate chain 2	mRNA
chr5	141516341	141516565	31	-	248	DIAPH1	diaphanous related formin 1	mRNA
chr11	69651751	69651925	31	+	279	CCND1	cyclin D1	mRNA
chr16	85806737	85806878	30	+	199	COX4I1	cytochrome c oxidase subunit 4I1	mRNA
chr8	116858351	116858458	30	-	108	RAD21	RAD21 cohesin complex component	mRNA
chr8	39023170	39023277	30	+	156	ADAM9	ADAM metallopeptidase domain 9	mRNA
chr8	39023279	39023325	30	+	156	ADAM9	ADAM metallopeptidase domain 9	mRNA
chr16	85804936	85805104	30	+	169	COX4I1	cytochrome c oxidase subunit 4I1	mRNA
chr17	7890570	7890683	30	+	172	CHD3	chromodomain helicase DNA binding protein 3	mRNA
chr8	39017218	39017277	30	+	197	ADAM9	ADAM metallopeptidase domain 9	mRNA

chr8	39017313	39017396	30	+	197	ADAM9	ADAM metallopeptidase domain 9	mRNA
chr19	40688692	40688774	30	-	191	NUMBL	NUMB like endocytic adaptor protein	mRNA
chr19	40688775	40688882	30	-	191	NUMBL	NUMB like endocytic adaptor protein	mRNA
chr7	44005005	44005167	30	-	229	SPDYE1	speedy/RINGO cell cycle regulator family member E1	mRNA
chr7	47351882	47351972	30	-	246	TNS3	tensin 3	mRNA
chr18	35246782	35246835	30	+	287	ZNF397	zinc finger protein 397	mRNA
chr3	48425838	48425958	30	-	298	PLXNB1	plexin B1	mRNA
chr9	133350094	133350164	30	+	71	SNORD36B		snoRNA
chr3	184325695	184325771	30	+	78	SNORD66		snoRNA
chr5	138561033	138561110	30	-	78	SNORD63		snoRNA
chr19	49489965	49490047	30	+	88	SNORD32A		snoRNA
chr12	56645012	56645094	30	-	105	SNORD59A		snoRNA
chr17	38852832	38852994	30	-	195	SNORA21		snoRNA
chr15	98959955	98960038	30	+	84	IGF1R	insulin like growth factor 1 receptor	mRNA
chr12	12917244	12917341	30	+	98	GPRC5A	G protein-coupled receptor, class C, group 5, member A	mRNA
chr1	183144964	183145107	30	+	144	LAMC1	laminin, gamma 1 (formerly LAMB2)	mRNA
chr12	120699143	120699270	30	+	144	MLEC	malectin	mRNA
chr9	34087834	34087939	30	-	145	DCAF12	DDB1 and CUL4 associated factor 12	mRNA
chr4	99062667	99062783	30	+	153	METAP1	methionyl aminopeptidase 1	mRNA
chr16	18792911	18793017	30	-	153	ARL6IP1	ADP ribosylation factor like GTPase 6 interacting protein 1	mRNA
chr16	18793026	18793063	30	-	153	ARL6IP1	ADP ribosylation factor like GTPase 6 interacting protein 1	mRNA
chr1	112913654	112913820	30	-	249	SLC16A1	solute carrier family 16 member 1	mRNA
chr14	61278932	61278997	30	-	251	TMEM30B	transmembrane protein 30B	mRNA
chr14	61279107	61279180	30	-	251	TMEM30B	transmembrane protein 30B	mRNA
chr14	55672629	55672701	29	+	73	KTN1	kinectin 1	mRNA
chr8	39025802	39025884	29	+	83	ADAM9	ADAM metallopeptidase domain 9	mRNA
chr5	179719667	179719781	29	+	115	CANX	calnexin	mRNA
chr1	209616470	209616624	29	-	155	LAMB3	laminin subunit beta 3	mRNA
chr10	71819713	71819900	29	-	188	PSAP	prosaposin	mRNA
chrX	136879191	136879273	29	-	83	RBMX	RNA binding motif protein X-linked	mRNA
chr16	87738579	87738663	29	-	234	KLHDC4	kelch domain containing 4	mRNA
chrX	151973532	151973751	29	-	235	GABRE	gamma-aminobutyric acid type A receptor epsilon subunit	mRNA
chr18	3594772	3594912	29	+	268	DLGAP1	DLG associated protein 1	mRNA
chr5	139284162	139284241	29	+	283	MATR3	matrin 3	mRNA
chr4	151103827	151103889	29	+	63	SNORD73A		snoRNA
chr6	31541100	31541172	29	-	73	SNORD84		snoRNA
chr18	49491663	49491768	29	-	106	SNORD58B		snoRNA
chr10	78040614	78040687	29	+	74	RPS24	ribosomal protein S24	mRNA
chr2	38068867	38068966	29	-	100	CYP1B1	cytochrome P450 family 1 subfamily B member 1	mRNA
chr1	1056001	1056113	29	+	120	AGRN	agrin	mRNA
chr2	135783853	135783977	29	+	126	UBXN4	UBX domain protein 4	mRNA
chr3	128070882	128071010	29	+	129	SEC61A1	SEC61 translocon alpha 1 subunit	mRNA
chr6	17615586	17615716	29	-	131	NUP153	nucleoporin 153	mRNA
chr6	31268915	31269047	29	-	134	HLA-C	major histocompatibility complex	mRNA
chr18	723426	723558	29	-	135	YES1	YES proto-oncogene 1	mRNA
chr18	723272	723413	29	-	143	YES1	YES proto-oncogene 1	mRNA
chr6	109367577	109367726	29	-	150	CD164	CD164 molecule	mRNA
chr1	20652215	20652372	29	-	158	DDOST	dolichyl-diphosphooligosaccharide--protein glycosyltransferase non-catalytic s	mRNA
chr12	130876191	130876259	29	+	160	RAN	RAN, member RAS oncogene family	mRNA
chr17	50751097	50751173	29	+	178	LUC7L3	LUC7 like 3 pre-mRNA splicing factor	mRNA
chr1	159918349	159918510	29	-	201	TAGLN2	transgelin 2	mRNA
chr9	5785890	5786106	29	-	217	ERMP1	endoplasmic reticulum metallopeptidase 1	mRNA
chr11	58618503	58618636	29	+	241	ZFP91	ZFP91 zinc finger protein	mRNA
chr6	107871577	107871717	29	-	244	SEC63	SEC63 homolog, protein translocation regulator	mRNA
chr19	1272581	1272754	29	+	264	CIRBP	cold inducible RNA binding protein	mRNA
chr6	117707146	117707224	29	+	287	NUS1	nuclear undecaprenyl pyrophosphate synthase 1 homolog (S. cerevisiae)	mRNA
chr6	117707227	117707265	29	+	287	NUS1	nuclear undecaprenyl pyrophosphate synthase 1 homolog (S. cerevisiae)	mRNA
chr12	108645265	108645317	29	-	317	CORO1C	coronin 1C	mRNA
chr12	108645329	108645424	29	-	317	CORO1C	coronin 1C	mRNA
chr12	108645554	108645572	29	-	317	CORO1C	coronin 1C	mRNA
chr1	8861005	8861429	29	-	425	ENO1	enolase 1	mRNA
chr8	11847051	11847168	28	-	118	CTSB	cathepsin B	mRNA
chr1	114730507	114730648	28	-	142	CSDE1	cold shock domain containing E1	mRNA
chr8	116726061	116726162	28	-	148	EIF3H	eukaryotic translation initiation factor 3 subunit H	mRNA

chr1	183110492	183110606	28	+	189	LAMC1	laminin, gamma 1 (formerly LAMB2)	mRNA
chr8	118247959	118248058	28	-	102	SAMD12	sterile alpha motif domain containing 12	mRNA
chr2	85543489	85543545	28	+	161	MAT2A	methionine adenosyltransferase 2A	mRNA
chr11	64306472	64306576	28	+	171	ESRRA	estrogen related receptor alpha	mRNA
chr7	55067991	55068033	28	+	244	EGFR	epidermal growth factor receptor	mRNA
chr6	31835262	31835326	28	+	67	SNORD48		snoRNA
chr12	56643682	56643750	28	-	73	SNORD59B		snoRNA
chr6	31536373	31536449	28	-	77	SNORD117		snoRNA
chr11	93731495	93731572	28	-	78	SNORD6		snoRNA
chr17	2330283	2330364	28	-	82	SNORD91A		snoRNA
chr18	49489233	49489309	28	-	90	SNORD58C		snoRNA
chr11	93733214	93733294	28	-	104	SNORD5		snoRNA
chr1	44775863	44775938	28	+	105	SNORD55		snoRNA
chr20	2462955	2463035	28	-	114	SNORD119		snoRNA
chr17	28722583	28722652	28	+	129	SNORD4A		snoRNA
chr11	75400390	75400444	28	+	150	SNORD15A		snoRNA
chr11	75400476	75400538	28	+	150	SNORD15A		snoRNA
chr4	173322285	173322360	28	+	76	GALNT7	polypeptide N-acetylgalactosaminyltransferase 7	mRNA
chr16	68835369	68835470	28	+	124	CDH1	cadherin 1	mRNA
chr16	16142142	16142253	28	+	128	ABCC1	ATP binding cassette subfamily C member 1	mRNA
chr12	1787780	1787953	28	+	175	ADIPOR2	adiponectin receptor 2	mRNA
chr1	205715444	205715611	28	-	184	NUCKS1	nuclear casein kinase and cyclin-dependent kinase substrate 1	mRNA
chr1	119913625	119913722	28	-	196	NOTCH2	notch receptor 2	mRNA
chr17	29262813	29262899	28	-	220	NUFIP2	nuclear FMR1 interacting protein 2	mRNA
chr17	29262903	29262968	28	-	220	NUFIP2	nuclear FMR1 interacting protein 2	mRNA
chr5	1462139	1462202	28	-	222	LPCAT1	lysophosphatidylcholine acyltransferase 1	mRNA
chr4	6696899	6697013	27	+	257	S100P	S100 calcium binding protein P	mRNA
chr4	6697016	6697061	27	+	257	S100P	S100 calcium binding protein P	mRNA
chr22	39522500	39522662	27	+	303	ATF4	activating transcription factor 4	mRNA
chr3	49360176	49360382	27	-	207	RHOA	ras homolog family member A	mRNA
chr11	111296108	111296207	27	-	149	COLCA1	colorectal cancer associated 1	mRNA
chr14	64186473	64186579	27	+	157	SYNE2	spectrin repeat containing nuclear envelope protein 2	mRNA
chr1	54853453	54853609	27	-	177	DHCR24	24-dehydrocholesterol reductase	mRNA
chr10	119049804	119049985	27	-	186	EIF3A	eukaryotic translation initiation factor 3 subunit A	mRNA
chr1	39568801	39568900	27	-	207	PABPC4	poly(A) binding protein cytoplasmic 4	mRNA
chr17	2329116	2329208	27	-	137	SNORD91B		snoRNA
chr1	93311197	93311289	27	-	109	CCDC18-AS1		lncRNA
chr1	233384645	233384756	27	+	114	MAP3K21	mitogen-activated protein kinase kinase kinase 21	mRNA
chr14	75134282	75134351	27	-	120	TMED10	transmembrane p24 trafficking protein 10	mRNA
chr19	53874630	53874762	27	+	138	MYADM	myeloid associated differentiation marker	mRNA
chr17	50751354	50751504	27	+	151	LUC7L3	LUC7 like 3 pre-mRNA splicing factor	mRNA
chr5	138934330	138934482	27	+	153	CTNNA1	catenin alpha 1	mRNA
chr12	48936802	48936921	27	-	165	ARF3	ADP ribosylation factor 3	mRNA
chr9	121340812	121340976	27	-	167	STOM	stomatin	mRNA
chr15	77046057	77046206	27	-	180	TSPAN3	tetraspanin 3	mRNA
chr2	20202187	20202295	27	-	187	SDC1	syndecan 1	mRNA
chr13	29512318	29512460	27	-	196	SLC7A1	solute carrier family 7 member 1	mRNA
chr2	200823394	200823541	27	+	199	BZW1	basic leucine zipper and W2 domains 1	mRNA
chr17	40457482	40457687	27	+	206	IGFBP4	insulin-like growth factor binding protein 4	mRNA
chr12	48938464	48938568	27	-	213	ARF3	ADP ribosylation factor 3	mRNA
chr12	48938570	48938676	27	-	213	ARF3	ADP ribosylation factor 3	mRNA
chr14	69714553	69714661	27	+	223	SUSD6	sushi domain containing 6	mRNA
chr11	2396940	2397067	27	+	299	CD81	CD81 molecule	mRNA
chr7	131502621	131502678	27	-	192	PODXL	podocalyxin like	mRNA
chr7	131502680	131502811	27	-	192	PODXL	podocalyxin like	mRNA
chr14	55627911	55628028	26	+	118	KTN1	kinectin 1	mRNA
chr14	55634525	55634658	26	+	134	KTN1	kinectin 1	mRNA
chr2	218234352	218234454	26	+	143	ARPC2	actin related protein 2/3 complex subunit 2	mRNA
chr15	76948409	76948471	26	+	144	RCN2	reticulocalbin 2	mRNA
chr12	56643402	56643587	26	-	186	ATP5F1B	ATP synthase F1 subunit beta	mRNA
chr1	156744162	156744348	26	-	187	HDGF	heparin binding growth factor	mRNA
chr3	105524417	105524491	26	+	233	ALCAM	activated leukocyte cell adhesion molecule	mRNA
chr2	61537639	61537809	26	-	171	XPO1	exportin 1	mRNA
chr19	6717539	6717624	26	+	86	C3	complement C3	mRNA

chr8	118292322	118292436	26	-	115	SAMD12	sterile alpha motif domain containing 12	mRNA
chr19	40687906	40688041	26	-	137	NUMBL	NUMB like endocytic adaptor protein	mRNA
chr17	59837965	59838108	26	+	144	VMP1	vacuole membrane protein 1	mRNA
chr8	117957785	117957885	26	-	178	EXT1	exostosin glycosyltransferase 1	mRNA
chr12	54285911	54285967	26	+	217	HNRNPA1	heterogeneous nuclear ribonucleoprotein A1	mRNA
chr16	87741814	87742004	26	-	271	KLHDC4	kelch domain containing 4	mRNA
chr18	49491282	49491348	26	-	85	SNORD58A		snoRNA
chr16	58548498	58548622	26	-	125	SNORA46		snoRNA
chr3	39408398	39408539	26	+	168	SNORA6		snoRNA
chr11	75404420	75404570	26	+	172	SNORD158		snoRNA
chr2	86135869	86136006	26	+	185	SNORD94		snoRNA
chr3	149173144	149173239	26	-	96	HPS3	HPS3 biogenesis of lysosomal organelles complex 2 subunit 1	mRNA
chr11	70435266	70435365	26	+	105	CTTN	cortactin	mRNA
chr3	194402947	194403050	26	-	105	ATP13A3	ATPase 13A3	mRNA
chr5	168549666	168549775	26	-	110	PANK3	pantothenate kinase 3	mRNA
chr3	105575926	105576040	26	+	115	ALCAM	activated leukocyte cell adhesion molecule	mRNA
chr16	18791691	18791735	26	-	129	ARL6IP1	ADP ribosylation factor like GTPase 6 interacting protein 1	mRNA
chr16	18791749	18791815	26	-	129	ARL6IP1	ADP ribosylation factor like GTPase 6 interacting protein 1	mRNA
chr6	47232051	47232158	26	-	137	TNFRSF21	tumor necrosis factor receptor superfamily, member 21	mRNA
chr1	235111208	235111348	26	-	141	TOMM20	translocase of outer mitochondrial membrane 20	mRNA
chr17	5024230	5024354	26	+	150	KIF1C	kinesin family member 1C	mRNA
chr1	31908756	31908913	26	-	158	PTP4A2	protein tyrosine phosphatase 4A2	mRNA
chr6	122444756	122444916	26	-	161	SERINC1	serine incorporator 1	mRNA
chr16	56362014	56362098	26	-	168	AMFR	autocrine motility factor receptor	mRNA
chr1	40073334	40073439	26	-	186	PPT1	palmitoyl-protein thioesterase 1	mRNA
chr18	31548623	31548716	26	+	194	DSG2	desmoglein 2	mRNA
chr9	111933600	111933648	26	+	196	UGCG	UDP-glucose ceramide glucosyltransferase	mRNA
chr11	64823848	64824016	26	-	211	CDC42BPG	CDC42 binding protein kinase gamma (DMPK-like)	mRNA
chr8	97852241	97852337	26	+	215	LAPTM4B	lysosomal protein transmembrane 4 beta	mRNA
chr8	97852361	97852410	26	+	215	LAPTM4B	lysosomal protein transmembrane 4 beta	mRNA
chr15	98963364	98963523	26	+	293	IGF1R	insulin like growth factor 1 receptor	mRNA
chr6	36601961	36602272	26	+	312	SRSF3	serine and arginine rich splicing factor 3	mRNA
chr12	49761703	49761779	25	+	77	TMBIM6	transmembrane BAX inhibitor motif containing 6	mRNA
chr16	87841049	87841155	25	-	123	SLC7A5	solute carrier family 7 member 5	mRNA
chr1	116398929	116399016	25	+	154	ATP1A1		mRNA
chr12	110343358	110343418	25	+	211	ATP2A2	ATPase sarcoplasmic/endoplasmic reticulum Ca2+ transporting 2	mRNA
chr1	43588772	43589000	25	+	271	PTPRF	protein tyrosine phosphatase receptor type F	mRNA
chr11	35194673	35194718	25	+	130	CD44	CD44 molecule	mRNA
chr8	118039936	118040045	25	-	152	EXT1	exostosin glycosyltransferase 1	mRNA
chr19	47720444	47720632	25	+	189	EHD2	EH domain containing 2	mRNA
chr7	47352237	47352366	25	-	202	TNS3	tensin 3	mRNA
chr6	85677576	85677658	25	-	83	SNORD50B		snoRNA
chr16	70529512	70529585	25	+	89	SNORD111B		snoRNA
chr2	28927982	28928060	25	+	122	SNORD53B		snoRNA
chr1	44776479	44776589	25	+	147	SNORD46		snoRNA
chr22	39312897	39312970	25	-	74	RPL3	ribosomal protein L3	mRNA
chr11	102398815	102398891	25	-	80	TMEM123	transmembrane protein 123	mRNA
chr12	112508317	112508409	25	+	104	PTPN11	protein tyrosine phosphatase non-receptor type 11	mRNA
chr14	69771536	69771644	25	+	114	SRSF5	serine and arginine rich splicing factor 5	mRNA
chr6	47232710	47232789	25	-	135	TNFRSF21	tumor necrosis factor receptor superfamily, member 21	mRNA
chr16	18792568	18792621	25	-	148	ARL6IP1	ADP ribosylation factor like GTPase 6 interacting protein 1	mRNA
chr9	5785630	5785767	25	-	149	ERMP1	endoplasmic reticulum metallopeptidase 1	mRNA
chr4	105970119	105970173	25	+	155	NPNT	nephronectin	mRNA
chr4	105970186	105970235	25	+	155	NPNT	nephronectin	mRNA
chr8	97852048	97852201	25	+	166	LAPTM4B	lysosomal protein transmembrane 4 beta	mRNA
chr2	128190780	128190951	25	+	172	UGGT1	UDP-glucose glycoprotein glucosyltransferase 1	mRNA
chr2	86777291	86777440	25	+	175	RMND5A	required for meiotic nuclear division 5 homolog A	mRNA
chr5	140706172	140706311	25	+	179	ZMAT2	zinc finger matrin-type 2	mRNA
chr8	97851933	97851967	25	+	189	LAPTM4B	lysosomal protein transmembrane 4 beta	mRNA
chr8	97851968	97852032	25	+	189	LAPTM4B	lysosomal protein transmembrane 4 beta	mRNA
chr9	129739076	129739256	25	-	206	PTGES	prostaglandin E synthase	mRNA
chr17	57006172	57006376	25	+	207	SCPEP1	serine carboxypeptidase 1	mRNA
chr14	55661537	55661606	24	+	70	KTN1	kinectin 1	mRNA
chr5	10394081	10394143	24	+	70	MARCHF6	membrane associated ring-CH-type finger 6	mRNA

chr14	55633234	55633321	24	+	88	KTN1	kinectin 1	mRNA
chr14	55636448	55636536	24	+	89	KTN1	kinectin 1	mRNA
chr10	71821875	71822007	24	-	133	PSAP	prosaposin	mRNA
chr6	137203546	137203675	24	-	179	IFNGR1	interferon gamma receptor 1	mRNA
chr1	183130343	183130549	24	+	207	LAMC1	laminin, gamma 1 (formerly LAMB2)	mRNA
chr22	17025077	17025131	24	-	104			no annotation
chr22	29291001	29291079	24	+	139	EWSR1	EWS RNA binding protein 1	mRNA
chr11	62531254	62531364	24	-	149	AHNAK	AHNAK nucleoprotein	mRNA
chr8	118288852	118288965	24	-	159	SAMD12	sterile alpha motif domain containing 12	mRNA
chr11	1745744	1745847	24	-	209	IFITM10	interferon induced transmembrane protein 10	mRNA
chr3	52692742	52692804	24	+	63	SNORD69		snoRNA
chr20	2656937	2657012	24	+	76	SNORD57		snoRNA
chr17	76561635	76561703	24	+	78	SNORD1A		snoRNA
chr2	202291334	202291420	24	+	88	SNORD11B		snoRNA
chr2	28927066	28927150	24	+	91	SNORD53		snoRNA
chr1	30968160	30968235	24	-	95	SNORD103C		snoRNA
chr11	123059326	123059445	24	-	120	SNORD14C		snoRNA
chr4	82895853	82895926	24	-	131	SNORD143		snoRNA
chr17	28720498	28720636	24	+	170	SNORD42B		snoRNA
chr19	40943749	40943799	24	+	88	CYP2B7P		pseudo
chr20	51597494	51597573	24	-	80	ATP9A	ATPase phospholipid transporting 9A	mRNA
chr14	21210586	21210680	24	-	97	HNRNPC	heterogeneous nuclear ribonucleoprotein C	mRNA
chr16	19121087	19121174	24	+	97	ITPRIPL2	inositol 1,4,5-trisphosphate receptor interacting protein-like 2	mRNA
chr1	26854797	26854897	24	+	102	ZDHHC18	zinc finger DHHC-type containing 18	mRNA
chr19	15160331	15160442	24	-	113	NOTCH3	notch receptor 3	mRNA
chr7	140453252	140453292	24	-	119	MKRN1	makorin ring finger protein 1	mRNA
chr7	140453335	140453370	24	-	119	MKRN1	makorin ring finger protein 1	mRNA
chr14	75131804	75131930	24	-	127	TMED10	transmembrane p24 trafficking protein 10	mRNA
chr20	45325971	45326097	24	-	127	SDC4	syndecan 4	mRNA
chr6	7289522	7289652	24	-	133	SSR1	signal sequence receptor subunit 1	mRNA
chr11	10307205	10307340	24	+	136	ADM	adrenomedullin	mRNA
chr22	45600839	45600983	24	+	145	FBLN1	fibulin 1	mRNA
chr19	53874496	53874627	24	+	145	MYADM	myeloid associated differentiation marker	mRNA
chr3	108046054	108046099	24	-	179	CD47	CD47 molecule	mRNA
chr12	113390224	113390366	24	+	216	PLBD2	phospholipase B domain containing 2	mRNA
chr1	10419689	10419847	24	+	257	PGD	phosphogluconate dehydrogenase	mRNA
chr16	1701024	1701184	24	+	262	JPT2	Jupiter microtubule associated homolog 2	mRNA
chr14	67677026	67677116	24	-	330	RDH11	retinol dehydrogenase 11	mRNA
chr15	78897048	78897220	24	+	342	MORF4L1	mortality factor 4 like 1	mRNA
chr15	78897267	78897328	24	+	342	MORF4L1	mortality factor 4 like 1	mRNA
chr22	38483521	38483620	24	-	101	DDX17	DEAD-box helicase 17	mRNA
chr6	113860605	113860679	23	+	273	MARCKS	myristoylated alanine-rich protein kinase C substrate	mRNA
chr17	58005356	58005410	23	-	300	SRSF1	serine and arginine rich splicing factor 1	mRNA
chr14	55675834	55675918	23	+	85	KTN1	kinectin 1	mRNA
chr1	43620873	43620992	23	+	120	PTPRF	protein tyrosine phosphatase receptor type F	mRNA
chr5	133097166	133097286	23	+	121	HSPA4	heat shock protein family A (Hsp70) member 4	mRNA
chr12	110327563	110327674	23	+	133	ATP2A2	ATPase sarcoplasmic/endoplasmic reticulum Ca2+ transporting 2	mRNA
chr16	85805709	85805864	23	+	158	COX4I1	cytochrome c oxidase subunit 4I1	mRNA
chr10	32919884	32919984	23	-	178	ITGB1	integrin subunit beta 1	mRNA
chr8	100721390	100721472	23	-	191	PABPC1	poly(A) binding protein cytoplasmic 1	mRNA
chr10	119042006	119042212	23	-	207	EIF3A	eukaryotic translation initiation factor 3 subunit A	mRNA
chr8	118038993	118039113	23	-	121	EXT1	exostosin glycosyltransferase 1	mRNA
chr21	38808461	38808601	23	+	145	ETS2	ETS proto-oncogene 2	mRNA
chr1	20774287	20774433	23	-	147	HP1BP3	heterochromatin protein 1 binding protein 3	mRNA
chr1	20774611	20774699	23	-	159	HP1BP3	heterochromatin protein 1 binding protein 3	mRNA
chr9	129338864	129339032	23	+	235	LINC01503		lncRNA
chr9	122880223	122880306	23	-	84	SNORD90		snoRNA
chr2	202293057	202293141	23	+	90	SNORD11		snoRNA
chr15	66502809	66502911	23	-	104	SNORD16		snoRNA
chr11	93721603	93721712	23	+	112	SCARNA9		scaRNA
chr14	95533567	95533629	23	-	188	SCARNA13		scaRNA
chr9	112218970	112219069	23	-	103	PTBP3	polypyrimidine tract binding protein 3	mRNA
chr6	106970836	106970914	23	-	106	CD24	CD24 molecule	mRNA
chr6	106970924	106970939	23	-	106	CD24	CD24 molecule	mRNA

chr7	135928959	135929034	23	-	117	MTPN	myotrophin	mRNA
chr2	20201983	20202097	23	-	126	SDC1	syndecan 1	mRNA
chr16	81380504	81380612	23	+	127	GAN	gigaxonin	mRNA
chr1	169132215	169132330	23	+	127	ATP1B1	ATPase Na+/K+ transporting subunit beta 1	mRNA
chr20	3805532	3805616	23	+	149	CDC25B	cell division cycle 25B	mRNA
chr1	32043358	32043489	23	+	161	KHDRBS1	KH RNA binding domain containing, signal transduction associated 1	mRNA
chr15	68207990	68208077	23	-	163	CLN6	CLN6 transmembrane ER protein	mRNA
chr8	22005431	22005611	23	+	181	XPO7	exportin 7	mRNA
chr17	4733751	4733872	23	-	181	CXCL16	C-X-C motif chemokine ligand 16	mRNA
chr2	65269345	65269395	23	+	186	ACTR2	actin related protein 2	mRNA
chr8	125021916	125022085	23	+	187	SQLE	squalene epoxidase	mRNA
chr15	98959655	98959767	23	+	205	IGF1R	insulin like growth factor 1 receptor	mRNA
chr2	201380401	201380516	23	-	208	TRAK2	trafficking kinesin protein 2	mRNA
chr17	5024627	5024716	23	+	210	KIF1C	kinesin family member 1C	mRNA
chr5	10434263	10434357	23	+	211	MARCHF6	membrane associated ring-CH-type finger 6	mRNA
chr1	42178162	42178266	23	-	249	FOXJ3	forkhead box J3	mRNA
chr9	128695190	128695305	23	+	294	SET	SET nuclear proto-oncogene	mRNA
chr15	64156047	64156145	23	-	298	PPIB	peptidylprolyl isomerase B (cyclophilin B)	mRNA
chr11	62571837	62571901	22	-	65	EEF1G	eukaryotic translation elongation factor 1 gamma	mRNA
chr12	103931520	103931623	22	+	104	HSP90B1	heat shock protein 90 beta family member 1	mRNA
chr1	114726982	114727090	22	-	109	CSDE1	cold shock domain containing E1	mRNA
chr11	130122369	130122425	22	+	210	APLP2	amyloid beta precursor like protein 2	mRNA
chr10	119042359	119042512	22	-	247	EIF3A	eukaryotic translation initiation factor 3 subunit A	mRNA
chr6	30616608	30616642	22	-	180	PPP1R10	protein phosphatase 1 regulatory subunit 10	mRNA
chr11	65085812	65085919	22	+	122	ZFPL1	zinc finger protein like 1	mRNA
chr11	62529113	62529234	22	-	122	AHNAK	AHNAK nucleoprotein	mRNA
chr20	49694844	49694935	22	-	124	B4GALT5	UDP-Gal:betaGlcNAc beta 1,4- galactosyltransferase, polypeptide 5	mRNA
chr19	47721068	47721130	22	+	138	EHD2	EH domain containing 2	mRNA
chr1	43596749	43596889	22	+	141	PTPRF	protein tyrosine phosphatase receptor type F	mRNA
chr1	42850072	42850211	22	+	150	ZNF691	zinc finger protein 691	mRNA
chr18	2686661	2686767	22	+	151	SMCHD1	structural maintenance of chromosomes flexible hinge domain containing 1	mRNA
chr1	70231445	70231545	22	+	120	SRSF11	serine and arginine rich splicing factor 1	mRNA
chr8	56073836	56073910	22	-	75	SNORD54		snoRNA
chr17	28723688	28723765	22	+	83	SNORD4B		snoRNA
chr1	92837288	92837392	22	+	105	SNORD21		snoRNA
chr14	20323178	20323328	22	-	151	SNORA79B		snoRNA
chr21	8392878	8393113	22	+	236	RNA18SN3		rRNA
chr21	8209842	8210079	22	+	249	RNA18SN2		rRNA
chr5	179836931	179837005	22	+	76	SQSTM1	sequestosome 1	mRNA
chr11	33709235	33709325	22	-	91	CD59	CD59 molecule	mRNA
chr16	19116681	19116776	22	+	96	ITPRIPL2	inositol 1,4,5-trisphosphate receptor interacting protein-like 2	mRNA
chr1	209786515	209786619	22	-	105	IRF6	interferon regulatory factor 6	mRNA
chr1	22091815	22091913	22	+	106	CDC42	Cell Division Cycle 42	mRNA
chr3	149173560	149173678	22	-	126	HPS3	HPS3 biogenesis of lysosomal organelles complex 2 subunit 1	mRNA
chr6	34587732	34587773	22	-	138	ILRUN	inflammation and lipid regulator with UBA-like and NBR1-like domains	mRNA
chr6	34587776	34587845	22	-	138	ILRUN	inflammation and lipid regulator with UBA-like and NBR1-like domains	mRNA
chr9	133362844	133362987	22	-	144	SURF4	surfeit 4	mRNA
chr17	37086315	37086420	22	-	144	ACACA	acetyl-CoA carboxylase alpha	mRNA
chr17	47682892	47683041	22	+	150	KPNB1	karyopherin subunit beta 1	mRNA
chr9	130139331	130139450	22	+	151	GPR107	G protein-coupled receptor 107	mRNA
chr9	33112008	33112100	22	-	157	B4GALT1	beta-1,4-galactosyltransferase 1	mRNA
chr12	48937017	48937127	22	-	170	ARF3	ADP ribosylation factor 3	mRNA
chr10	114434303	114434377	22	-	171	ABLIM1	actin binding LIM protein 1	mRNA
chr2	218254115	218254155	22	+	180	ARPC2	actin related protein 2/3 complex subunit 2	mRNA
chr1	53246893	53247056	22	-	180	**LRP8**	LDL receptor related protein 8	mRNA
chr1	1785392	1785454	22	-	186	GNB1	G protein subunit beta 1	mRNA
chr14	70371170	70371366	22	-	230	SYNJ2BP-COX	SYNJ2BP-COX16 readthrough	mRNA
chr3	152464729	152464862	22	+	264	MBNL1	muscleblind like splicing regulator 1	mRNA
chr8	39054480	39054524	21	+	45	ADAM9	ADAM metallopeptidase domain 9	mRNA
chr14	55671565	55671627	21	+	63	KTN1	kinectin 1	mRNA
chr5	10426389	10426489	21	+	101	MARCHF6	membrane associated ring-CH-type finger 6	mRNA
chr1	230250456	230250560	21	+	105	GALNT2	polypeptide N-acetylgalactosaminyltransferase 2	mRNA
chr18	36109618	36109683	21	-	128	SLC39A6	solute carrier family 39 member 6	mRNA
chr3	184324200	184324347	21	+	148	EIF4G1	eukaryotic translation initiation factor 4 gamma 1	mRNA

chr19	48913053	48913128	21	+	187	NUCB1	nucleobindin 1	mRNA
chr8	47807136	47807299	21	-	200	PRKDC	protein kinase, DNA-activated, catalytic subunit	mRNA
chr18	3451868	3451953	21	+	141	TGIF1	TGFB induced factor homeobox 1	mRNA
chr8	118285059	118285108	21	-	101	SAMD12	sterile alpha motif domain containing 12	mRNA
chrX	120438720	120438854	21	-	135	LAMP2	lysosomal associated membrane protein 2	mRNA
chr1	1391160	1391243	21	-	153	CCNL2	cyclin L2	mRNA
chr18	3455220	3455281	21	+	172	TGIF1	TGFB induced factor homeobox 1	mRNA
chr7	97971624	97971728	21	-	157	CZ1P-ASNS		lncRNA
chr5	86620506	86620573	21	+	86	SNORD138		snoRNA
chr17	28723417	28723492	21	+	91	SNORD42A		snoRNA
chr19	50802328	50802416	21	-	118	SNORD88C		snoRNA
chr5	40832642	40832732	21	-	128	SNORD72		snoRNA
chr9	127448500	127448637	21	-	148	SNORA65		snoRNA
chr1	28580919	28581053	21	-	174	SNORA16A		snoRNA
chr2	202206714	202206802	21	-	89	SUMO1	small ubiquitin like modifier 1	mRNA
chr6	75253308	75253394	21	-	90	TMEM30A	transmembrane protein 30A	mRNA
chr10	87754296	87754390	21	-	101	ATAD1	ATPase family AAA domain containing 1	mRNA
chr3	128620028	128620101	21	-	111	RPN1	ribophorin I	mRNA
chr12	111809692	111809760	21	+	120	ALDH2	aldehyde dehydrogenase 2 family member	mRNA
chr6	79201635	79201726	21	-	122	HMGN3	high mobility group nucleosomal binding domain 3	mRNA
chr11	57816697	57816773	21	+	129	CTNND1	catenin delta 1	mRNA
chr6	34588848	34588959	21	-	136	ILRUN	inflammation and lipid regulator with UBA-like and NBR1-like domains	mRNA
chr2	37105709	37105763	21	-	138	EIF2AK2	eukaryotic translation initiation factor 2 alpha kinase 2	mRNA
chr2	37105769	37105833	21	-	138	EIF2AK2	eukaryotic translation initiation factor 2 alpha kinase 2	mRNA
chr6	7287549	7287691	21	-	143	SSR1	signal sequence receptor subunit 1	mRNA
chr11	121633103	121633249	21	+	148	SORL1	sortilin related receptor 1	mRNA
chr6	99400161	99400240	21	-	164	PNISR	PNN interacting serine and arginine rich protein	mRNA
chr3	49534191	49534262	21	+	179	DAG1	dystroglycan 1	mRNA
chr12	49763501	49763681	21	+	181	TMBIM6	transmembrane BAX inhibitor motif containing 7	mRNA
chr3	31637420	31637462	21	+	183	STT3B	STT3 oligosaccharyltransferase complex catalytic subunit B	mRNA
chr1	35715234	35715359	21	-	186	C1orf216	chromosome 1 open reading frame 216	mRNA
chr1	225497342	225497425	21	-	208	ENAH	ENAH actin regulator	mRNA
chr2	131051855	131051947	21	-	226	FAM168B	family with sequence similarity 168 member B	mRNA
chrX	41348613	41348762	21	+	238	DDX3X	DEAD-box helicase 3 X-linked	mRNA
chr12	53041270	53041420	21	+	238	EIF4B	eukaryotic translation initiation factor 4B	mRNA
chr13	113264490	113264627	21	+	247	CUL4A	cullin 4A	mRNA
chr8	100717772	100717888	20	-	117	PABPC1	poly(A) binding protein cytoplasmic 1	mRNA
chr12	21654542	21654659	20	-	118	LDHB	lactate dehydrogenase B	mRNA
chr1	30995064	30995178	20	-	126	PUM1	pumilio RNA binding family member 1	mRNA
chr11	10802293	10802421	20	-	129	EIF4G2	eukaryotic translation initiation factor 4 gamma 2	mRNA
chr6	31626070	31626162	20	+	140	PRRC2A	proline rich coiled-coil 2A	mRNA
chr12	57718145	57718359	20	+	215	OS9	OS9 endoplasmic reticulum lectin	mRNA
chr14	92045580	92045702	20	+	141			no annotation
chr12	102383317	102383376	20	+	128			no annotation
chr5	139686663	139686753	20	+	139			no annotation
chr14	38194203	38194235	20	-	140			no annotation
chr19	51355315	51355371	20	-	62	ETFB	electron transfer flavoprotein subunit beta	mRNA
chr8	118279150	118279214	20	-	116	SAMD12	sterile alpha motif domain containing 12	mRNA
chr19	15331024	15331091	20	-	134	BRD4	bromodomain containing 4	mRNA
chr20	44209672	44209756	20	-	134	OSER1	oxidative stress responsive serine rich 1	mRNA
chr6	169649755	169649886	20	-	179	WDR27	WD repeat domain 27	mRNA
chr15	85384870	85385042	20	+	179	AKAP13	A-kinase anchoring protein 13	mRNA
chr14	19302662	19302785	20	-	124	LINC01296		lncRNA
chr17	72557577	72557695	20	-	150	LINC00673		lncRNA
chr2	121530887	121531010	20	+	148	RNU4ATAC		snRNA
chr8	66922473	66922551	20	-	79	SNORD87		snoRNA
chr3	52690755	52690817	20	+	99	SNORD19B		snoRNA
chr1	28580380	28580513	20	-	134	SNORA44		snoRNA
chr1	28579755	28579896	20	-	146	SNORA61		snoRNA
chr19	11450904	11450967	20	+	64	PRKCSH	protein kinase C substrate 80K-H	mRNA
chr1	43622436	43622508	20	+	73	PTPRF	protein tyrosine phosphatase receptor type F	mRNA
chr17	44206436	44206523	20	-	88	UBTF	upstream binding transcription factor	mRNA
chr8	56960180	56960257	20	-	93	IMPAD1	inositol monophosphatase domain containing 1	mRNA
chr12	46358290	46358385	20	-	96	SLC38A2	solute carrier family 38 member 2	mRNA

chr1	20743271	20743353	20	-	105	HP1BP3	heterochromatin protein 1 binding protein 3	mRNA
chr1	43622376	43622434	20	+	106	PTPRF	protein tyrosine phosphatase receptor type F	mRNA
chr1	234606162	234606231	20	-	112	IRF2BP2	interferon regulatory factor 2 binding protein 2	mRNA
chr12	64695436	64695528	20	+	126	RASSF3	Ras association domain family member 3	mRNA
chr4	39780234	39780346	20	+	126	UBE2K	ubiquitin conjugating enzyme E2 K	mRNA
chr14	77506036	77506162	20	-	130	SPTLC2	serine palmitoyltransferase long chain base subunit 2	mRNA
chr17	40194279	40194395	20	+	132	RAPGEFL1	Rap guanine nucleotide exchange factor like 1	mRNA
chr19	15159633	15159740	20	-	133	NOTCH3	notch receptor 3	mRNA
chr7	155309609	155309740	20	+	136	INSIG1	insulin induced gene 1	mRNA
chr7	88279212	88279316	20	-	152	STEAP4	STEAP4 metalloreductase	mRNA
chr1	1785995	1786044	20	-	157	GNB1	G protein subunit beta 1	mRNA
chr5	97032728	97032785	20	+	160	LNPEP	leucyl and cystinyl aminopeptidase	mRNA
chr8	27598022	27598077	20	-	164	CLU	clusterin	mRNA
chr7	44834271	44834360	20	-	172	H2AZ2	H2A.Z variant histone 2	mRNA
chr8	30678701	30678801	20	-	177	GSR	glutathione-disulfide reductase	mRNA
chr6	82366370	82366478	20	+	192	TPBG	trophoblast glycoprotein	mRNA
chr2	26395387	26395482	20	+	198	SELENOI	selenoprotein I	mRNA
chr1	207360131	207360299	20	+	298	CD55	CD55 molecule	mRNA
chr3	112638173	112638250	19	-	81	CCDC80	coiled-coil domain containing 80	mRNA
chr15	90476695	90476803	19	+	109	IQGAP1	IQ motif containing GTPase activating protein 1	mRNA
chr1	116384013	116384059	19	+	112	ATP1A1	ATPase Na+/K+ transporting subunit alpha 1	mRNA
chr1	116384065	116384124	19	+	112	ATP1A1	ATPase Na+/K+ transporting subunit alpha 1	mRNA
chr2	10802498	10802623	19	-	126	PDIA6	protein disulfide isomerase family A member 6	mRNA
chr8	125021777	125021900	19	+	126	SQLE	squalene epoxidase	mRNA
chr1	23692608	23692759	19	+	152	RPL11	ribosomal protein L11	mRNA
chr11	62530347	62530410	19	-	111	AHNAK	AHNAK nucleoprotein	mRNA
chr1	1052808	1052914	19	+	122	AGRN	agrin	mRNA
chr12	52237862	52237915	19	+	133	KRT7	keratin 7	mRNA
chr19	40688178	40688245	19	-	147	NUMBL	NUMB like endocytic adaptor protein	mRNA
chrX	74265122	74265190	19	-	69	FTX		lncRNA
chr19	12706448	12706518	19	-	71	SNORD41		snoRNA
chr14	20326452	20326523	19	-	75	SNORD126		snoRNA
chr1	173864162	173864243	19	-	93	SNORD81		snoRNA
chr1	155919908	155920018	19	-	131	SNORA80E		snoRNA
chr6	159680357	159680446	19	-	90	SOD2	superoxide dismutase 2	mRNA
chr14	75133322	75133412	19	-	91	TMED10	transmembrane p24 trafficking protein 10	mRNA
chr1	154582305	154582357	19	-	96	ADAR	adenosine deaminase RNA specific	mRNA
chr11	65854946	65855040	19	-	99	CFL1	cofilin 1	mRNA
chr5	97034494	97034584	19	+	100	LNPEP	leucyl and cystinyl aminopeptidase	mRNA
chr2	54972193	54972284	19	-	105	RTN4	reticulon 4	mRNA
chr11	57552125	57552233	19	-	114	UBE2L6	ubiquitin conjugating enzyme E2 L6	mRNA
chr16	70249596	70249673	19	-	115	EXOSC6	exosome component 6	mRNA
chr5	151806330	151806420	19	+	116	G3BP1	G3BP stress granule assembly factor 1	mRNA
chr10	5457879	5457996	19	+	118	NET1	neuroepithelial cell transforming 1	mRNA
chr17	4959282	4959399	19	-	118	SPAG7	sperm associated antigen 7	mRNA
chrX	68533030	68533094	19	+	121	YIPF6	Yip1 domain family member 6	mRNA
chr1	207795031	207795147	19	+	134	CD46	CD46 molecule	mRNA
chr10	96522117	96522251	19	-	135	TM9SF3	transmembrane 9 superfamily member 3	mRNA
chr3	128813685	128813808	19	+	137	RAB7A	RAB7A, member RAS oncogene family	mRNA
chr1	152032537	152032673	19	-	137	S100A11	S100 calcium binding protein A11	mRNA
chr17	759832	759926	19	-	144	GLOD4	glyoxalase domain containing 4	mRNA
chr3	14486915	14486976	19	+	146	SLC6A6	solute carrier family 6 member 6	mRNA
chr3	177025061	177025138	19	-	148	TBL1XR1	transducin beta like 1 X-linked receptor 1	mRNA
chr3	177025164	177025205	19	-	148	TBL1XR1	transducin beta like 1 X-linked receptor 1	mRNA
chr2	9488618	9488708	19	-	149	IAH1	isoamyl acetate hydrolyzing esterase 1 (putative)	mRNA
chr8	142682527	142682615	19	+	150	PSCA	prostate stem cell antigen	mRNA
chr8	43122941	43122982	19	+	150	POMK	protein O-mannose kinase	mRNA
chr17	35266313	35266466	19	+	155	SLFN5	schlafen family member 5	mRNA
chr9	121340174	121340235	19	-	155	STOM	stomatin	mRNA
chr19	42378159	42378240	19	+	164	MEGF8	multiple EGF like domains 8	mRNA
chr9	123103881	123103926	19	+	166	RABGAP1	RAB GTPase activating protein 1	mRNA
chr3	49534264	49534430	19	+	167	DAG1	dystroglycan 1	mRNA
chr1	167788236	167788302	19	+	171	MPZL1	myelin protein zero like 1	mRNA
chr1	167788311	167788377	19	+	171	MPZL1	myelin protein zero like 1	mRNA

chr2	20250342	20250390	19	-	172	PUM2	pumilio RNA binding family member 2	mRNA
chr11	119664486	119664564	19	-	183	NECTIN1	nectin cell adhesion molecule 1	mRNA
chr20	32332651	32332754	19	+	200	KIF3B	kinesin family member 3B	mRNA
chr1	116988839	116988900	19	+	214	PTGFRN	prostaglandin F2 receptor inhibitor	mRNA
chrX	147949571	147949618	19	+	216	FMR1	FMRP translational regulator 1	mRNA
chr19	48197350	48197481	19	+	236	ZSWIM9	zinc finger SWIM-type containing 9	mRNA
chr1	1055393	1055646	19	+	275	AGRN	agrin	mRNA
chr4	55425375	55425469	18	+	95	TMEM165	transmembrane protein 165	mRNA
chr1	39569862	39569924	18	-	63	PABPC4	poly(A) binding protein cytoplasmic 4	mRNA
chr11	62884456	62884525	18	+	70	SLC3A2	solute carrier family 3 member 2	mRNA
chr1	244858728	244858807	18	-	80	HNRNPU	heterogeneous nuclear ribonucleoprotein U	mRNA
chr1	183134723	183134809	18	+	87	LAMC1	laminin, gamma 1 (formerly LAMB2)	mRNA
chr4	118308437	118308542	18	-	106	PRSS12	protease, serine, 12 (neurotrypsin, motopsin)	mRNA
chr19	38283717	38283797	18	+	109	SPINT2	serine peptidase inhibitor, Kunitz type 2	mRNA
chr5	141574066	141574139	18	-	109	DIAPH1	diaphanous related formin 1	mRNA
chr10	32920273	32920321	18	-	113	ITGB1	integrin subunit beta 1	mRNA
chr9	87730373	87730498	18	+	126	CTSL	cathepsin L	mRNA
chr1	55146997	55147034	18	-	133	USP24	ubiquitin specific peptidase 24	mRNA
chr12	102383801	102383853	18	+	120			no annotation
chr14	104858724	104858836	18	-	156			no annotation
chr1	207767464	207767534	18	+	75	CD46	CD46 molecule	mRNA
chr1	103554374	103554446	18	+	94	RNPC3	RNA binding region (RNP1, RRM) containing 3	mRNA
chr22	46382095	46382198	18	-	105	CELSR1	cadherin EGF LAG seven-pass G-type receptor 1	mRNA
chr2	61536942	61537039	18	-	120	XPO1	exportin 1	mRNA
chr22	38488801	38488918	18	-	138	DDX17	DEAD-box helicase 17	mRNA
chr13	113215029	113215120	18	+	289	CUL4A	cullin 4A	mRNA
chr20	17962711	17962946	18	-	236	SNORD17		snoRNA
chr21	8217555	8217835	18	+	295	RNA18SN2		rRNA
chr7	121349777	121349846	18	-	70	FAM3C	family with sequence similarity 3 member C	mRNA
chr18	21867869	21867924	18	+	90	MIB1	mindbomb E3 ubiquitin protein ligase 1	mRNA
chr19	5206129	5206164	18	-	90	PTPRS	protein tyrosine phosphatase receptor type S	mRNA
chr5	80141115	80141191	18	-	90	SERINC5	serine incorporator 5	mRNA
chr19	48382957	48383050	18	-	97	KDELR1	KDEL endoplasmic reticulum protein retention receptor 1	mRNA
chr19	53876312	53876406	18	+	99	MYADM	myeloid associated differentiation marker	mRNA
chr1	9729917	9730018	18	-	102	CLSTN1	calsyntenin 1	mRNA
chr14	69462001	69462106	18	+	106	SLC39A9	solute carrier family 39 member 9	mRNA
chr3	49534524	49534622	18	+	110	DAG1	dystroglycan 1	mRNA
chr3	49534623	49534631	18	+	110	DAG1	dystroglycan 1	mRNA
chr16	19118499	19118543	18	+	111	ITPRIPL2	inositol 1,4,5-trisphosphate receptor interacting protein-like 2	mRNA
chr4	55425484	55425595	18	+	115	TMEM165	transmembrane protein 165	mRNA
chr10	100549793	100549881	18	+	116	HIF1AN	hypoxia inducible factor 1 subunit alpha inhibitor	mRNA
chr2	96185069	96185126	18	-	116	STARD7	StAR related lipid transfer domain containing 7	mRNA
chr2	9584359	9584440	18	-	118	YWHAQ	tyrosine 3-monooxygenase/tryptophan 5-monooxygenase activation protein the	mRNA
chr16	10529028	10529092	18	-	123	EMP2	epithelial membrane protein 2	mRNA
chr7	23505078	23505163	18	-	123	TRA2A	transformer 2 alpha homolog	mRNA
chr19	11133617	11133743	18	+	128	LDLR	low density lipoprotein receptor	mRNA
chr1	203852437	203852565	18	+	130	ZC3H11A	zinc finger CCCH-type containing 11A	mRNA
chr14	54427774	54427841	18	-	136	CNIH1	cornichon family AMPA receptor auxiliary protein 1	mRNA
chr19	50310219	50310360	18	+	142	MYH14	myosin heavy chain 14	mRNA
chr1	207793646	207793793	18	+	148	CD46	CD46 molecule	mRNA
chr19	48923153	48923268	18	+	149	NUCB1	nucleobindin 1	mRNA
chr4	56933986	56934025	18	+	150	REST	RE1 silencing transcription factor	mRNA
chr9	92032289	92032412	18	-	150	SPTLC1	serine palmitoyltransferase long chain base subunit 1	mRNA
chr12	57095800	57095943	18	-	151	STAT6	signal transducer and activator of transcription 6	mRNA
chr10	87753188	87753308	18	-	154	ATAD1	ATPase family AAA domain containing 1	mRNA
chr3	197043326	197043381	18	-	156	DLG1	discs large MAGUK scaffold protein 1	mRNA
chr5	6671320	6671417	18	+	197	SRD5A1	steroid 5 alpha-reductase 1	mRNA
chr11	319740	319870	17	-	276	IFITM3	interferon induced transmembrane protein 3	mRNA
chr8	144789768	144789829	17	-	99	RPL8	ribosomal protein L8	mRNA
chr8	100718245	100718280	17	-	36	PABPC1	poly(A) binding protein cytoplasmic 1	mRNA
chr15	100905522	100905589	17	+	68	ALDH1A3	aldehyde dehydrogenase 1 family member A3	mRNA
chrX	53563745	53563821	17	-	77	HUWE1	HECT, UBA and WWE domain containing E3 ubiquitin protein ligase 1	mRNA
chr12	52517901	52517992	17	-	97	KRT5	keratin 5	mRNA
chr17	75729287	75729390	17	+	104	ITGB4	integrin subunit beta 4	mRNA

chr1	186326103	186326163	17	-	111	TPR	translocated promoter region	mRNA
chr1	186326165	186326213	17	-	111	TPR	translocated promoter region	mRNA
chr3	128626732	128626832	17	-	127	RPN1	ribophorin I	mRNA
chr3	112638102	112638149	17	-	133	CCDC80	coiled-coil domain containing 80	mRNA
chr2	172469188	172469302	17	+	177	ITGA6	integrin subunit alpha 6	mRNA
chr1	43619321	43619408	17	+	200	PTPRF	protein tyrosine phosphatase receptor type F	mRNA
chr11	83422633	83422677	17	-	68			no annotation
chr13	109269750	109269835	17	-	163			no annotation
chr22	17026896	17026995	17	-	100			no annotation
chr17	59839571	59839672	17	+	117	VMP1	vacuole membrane protein 1	mRNA
chr17	17821094	17821161	17	-	129	SREBF1	sterol regulatory element binding transcription factor 1	mRNA
chr12	76068630	76068700	17	-	133	NAP1L1	nucleosome assembly protein 1 like 1	mRNA
chr11	33140342	33140436	17	-	142	CSTF3	cleavage stimulation factor subunit 3	mRNA
chr15	100883033	100883102	17	+	153	ALDH1A3	aldehyde dehydrogenase 1 family member A3	mRNA
chr9	28533	28679	17	-	161	WASHC1	WASH complex subunit 1	mRNA
chr8	23568147	23568285	17	+	168	SLC25A37	solute carrier family 25 member 37	mRNA
chr8	127993071	127993141	17	+	72	PVT1		lncRNA
chr17	72557785	72557932	17	-	148	LINC00673		lncRNA
chr19	10107662	10107724	17	+	63	SNORD105		snoRNA
chr9	33934300	33934368	17	-	69	SNORD121B		snoRNA
chr2	206161881	206161950	17	+	70	SNORD51		snoRNA
chr16	70538031	70538100	17	+	95	SNORD111		snoRNA
chr20	62388441	62388498	17	+	58	RPS21	ribosomal protein S21	mRNA
chr4	112193795	112193855	17	+	69	FAM241A	family with sequence similarity 241 member A	mRNA
chr2	177219638	177219690	17	+	75	HNRNPA3	heterogeneous nuclear ribonucleoprotein A3	mRNA
chr3	133589805	133589853	17	+	84	CDV3	CDV3 homolog	mRNA
chr3	133589854	133589888	17	+	84	CDV3	CDV3 homolog	mRNA
chr3	196051221	196051305	17	-	90	TFRC	transferrin receptor	mRNA
chr19	53874967	53875059	17	+	93	MYADM	myeloid associated differentiation marker	mRNA
chr15	73560811	73560913	17	-	103	NPTN	neuroplastin	mRNA
chr18	672976	673078	17	+	103	TYMS	thymidylate synthetase	mRNA
chr11	102398003	102398111	17	-	110	TMEM123	transmembrane protein 123	mRNA
chr5	151268044	151268154	17	+	111	GM2A	GM2 ganglioside activator	mRNA
chr8	56960045	56960160	17	-	116	IMPAD1	inositol monophosphatase domain containing 1	mRNA
chr3	105575799	105575890	17	+	117	ALCAM	activated leukocyte cell adhesion molecule	mRNA
chr3	13316252	13316358	17	-	119	NUP210	nucleoporin 210	mRNA
chr11	62888644	62888762	17	+	119	SLC3A2	solute carrier family 3 member 2	mRNA
chr15	98964366	98964466	17	+	127	IGF1R	insulin like growth factor 1 receptor	mRNA
chr11	111294744	111294793	17	-	138	COLCA1	colorectal cancer associated 1	mRNA
chr11	33708900	33709038	17	-	139	CD59	CD59 molecule	mRNA
chr1	53246343	53246478	17	-	142	LRP8	LDL receptor related protein 8	mRNA
chr3	47852093	47852236	17	-	144	MAP4	microtubule associated protein 4	mRNA
chr6	115939044	115939120	17	-	160	FRK	fyn-related Src family tyrosine kinase	mRNA
chr2	177222334	177222427	17	+	160	HNRNPA3	heterogeneous nuclear ribonucleoprotein A3	mRNA
chr5	97036288	97036411	17	+	161	LNPEP	leucyl and cystinyl aminopeptidase	mRNA
chr6	160105775	160105852	17	+	165	IGF2R	insulin like growth factor 2 receptor	mRNA
chr6	42103077	42103240	17	-	166	C6orf132	chromosome 6 open reading frame 132	mRNA
chr9	83969217	83969396	17	-	180	HNRNPK	heterogeneous nuclear ribonucleoprotein K	mRNA
chr19	38730173	38730323	17	+	185	ACTN4	actinin alpha 4	mRNA
chr6	43181267	43181298	17	+	190	SRF	serum response factor (c-fos serum response element-binding transcription fact	mRNA
chr6	34245810	34245957	17	+	208	HMGA1	high mobility group AT-hook 1	mRNA
chr1	28147534	28147669	17	-	213	PTAFR	platelet activating factor receptor	mRNA
chr22	41663803	41664038	17	+	242	XRCC6	X-ray repair cross complementing 6	mRNA
chr6	31817748	31817837	17	+	242	HSPA1A	heat shock 70kDa protein 1A	mRNA
chr9	105385421	105385452	16	+	82	SLC44A1	solute carrier family 44 member 1	mRNA
chr12	53297849	53297924	16	+	83	PFDN5	prefoldin subunit 5	mRNA
chr11	34076559	34076642	16	+	84	CAPRIN1	cell cycle associated protein 1	mRNA
chr1	183118084	183118146	16	+	97	LAMC1	laminin, gamma 1 (formerly LAMB2)	mRNA
chr8	124543086	124543194	16	+	109	NDUFB9	NADH:ubiquinone oxidoreductase subunit B9	mRNA
chr2	74369423	74369491	16	-	109	DCTN1	dynactin subunit 1	mRNA
chr3	41224525	41224650	16	+	126	CTNNB1	catenin beta 1	mRNA
chr3	25633924	25633994	16	-	134	TOP2B	DNA topoisomerase II beta	mRNA
chr1	23694659	23694796	16	+	138	RPL11	ribosomal protein L11	mRNA
chr6	158783540	158783589	16	-	139	EZR	ezrin	mRNA

chr5	136049490	136049580	16	+	151	TGFBI	transforming growth factor beta induced	mRNA
chr1	81907083	81907214	16	+	153	ADGRL2	adhesion G protein-coupled receptor L2	mRNA
chr3	49122162	49122346	16	-	231	LAMB2	laminin subunit beta 2	mRNA
chr14	21263058	21263128	16	-	83	HNRNPC	heterogeneous nuclear ribonucleoprotein C	mRNA
chr20	5588878	5588925	16	-	84	GPCPD1	glycerophosphocholine phosphodiesterase 1	mRNA
chr3	42627549	42627601	16	+	86	NKTR	natural killer cell triggering receptor	mRNA
chr17	50751847	50751913	16	+	93	LUC7L3	LUC7 like 3 pre-mRNA splicing factor	mRNA
chr7	23530197	23530264	16	-	99	TRA2A	transformer 2 alpha homolog	mRNA
chr20	34072860	34072924	16	+	127	RALY	RALY heterogeneous nuclear ribonucleoprotein	mRNA
chr19	49102838	49102965	16	+	128	SNRNP70	small nuclear ribonucleoprotein U1 subunit 70	mRNA
chr17	17820892	17820943	16	-	165	SREBF1	sterol regulatory element binding transcription factor 1	mRNA
chr8	127480294	127480339	16	+	126	CASC8		lncRNA
chr19	12703597	12703669	16	-	73	SNORD135		snoRNA
chr20	2656639	2656695	16	+	73	SNORD56		snoRNA
chr1	155925957	155926023	16	-	67	SCARNA4		scaRNA
chr21	8392665	8392834	16	+	170	RNA45SN3		rRNA
chr4	14473600	14473687	16	+	95	LINC00504		lncRNA
chr14	55684098	55684150	16	+	53	KTN1	kinectin 1	mRNA
chr11	75405603	75405667	16	+	65	RPS3	ribosomal protein S3	mRNA
chr5	1461612	1461681	16	-	70	LPCAT1	lysophosphatidylcholine acyltransferase 1	mRNA
chr3	196049296	196049366	16	-	76	TFRC	transferrin receptor	mRNA
chr5	95888068	95888151	16	-	86	ELL2	elongation factor for RNA polymerase II 2	mRNA
chr7	73832659	73832690	16	+	87	CLDN4	claudin 4	mRNA
chr14	49583589	49583675	16	-	87	RPS29	ribosomal protein S29	mRNA
chr1	119914385	119914460	16	-	88	NOTCH2	notch receptor 2	mRNA
chr1	46677044	46677132	16	-	95	EFCAB14	EF-hand calcium binding domain 14	mRNA
chr1	202015800	202015845	16	+	98	ELF3	E74 like ETS transcription factor 3	mRNA
chr16	10530199	10530293	16	-	98	EMP2	epithelial membrane protein 2	mRNA
chr20	32850065	32850140	16	+	101	MAPRE1	microtubule-associated protein, RP/EB family, member 1	mRNA
chr11	47468271	47468333	16	-	101	CELF1	CUGBP Elav-like family member 1	mRNA
chr5	95795777	95795874	16	+	102	RHOBTB3	Rho related BTB domain containing 3	mRNA
chr5	159157601	159157648	16	-	103	RNF145	ring finger protein 145	mRNA
chr14	70370791	70370876	16	-	104	SYNJ2BP-COX	SYNJ2BP-COX16 readthrough	mRNA
chr2	212999914	212999997	16	-	107	IKZF2	IKAROS family zinc finger 2	mRNA
chr6	137197855	137197934	16	-	109	IFNGR1	interferon gamma receptor 1	mRNA
chr3	64002437	64002549	16	+	113	ATXN7	ataxin 7	mRNA
chr20	64275257	64275343	16	+	115	PCMTD2	protein-L-isoaspartate (D-aspartate) O-methyltransferase domain containing 2	mRNA
chr16	84975548	84975648	16	-	117	ZDHHC7	zinc finger DHHC-type containing 7	mRNA
chr8	37754977	37755093	16	+	117	ERLIN2	ER lipid raft associated 2	mRNA
chr14	95190864	95190908	16	-	118	CLMN	calmin	mRNA
chr17	29256974	29257050	16	-	120	NUFIP2	nuclear FMR1 interacting protein 2	mRNA
chr19	54193531	54193599	16	+	120	TSEN34	tRNA splicing endonuclease subunit 34	mRNA
chr9	113261807	113261894	16	+	122	SLC31A1	solute carrier family 31 (copper transporter), member 1	mRNA
chr3	141925657	141925778	16	+	122	ATP1B3	ATPase Na+/K+ transporting subunit beta 3	mRNA
chr14	63685849	63685954	16	-	124	SGPP1	sphingosine-1-phosphate phosphatase 1	mRNA
chr3	160501844	160501963	16	-	126	KPNA4	karyopherin subunit alpha 4	mRNA
chr1	156465525	156465650	16	-	126	MEF2D	myocyte enhancer factor 2D	mRNA
chr12	909853	909979	16	+	127	WNK1	WNK lysine deficient protein kinase 1	mRNA
chr2	222943113	222943188	16	+	130	ACSL3	acyl-CoA synthetase long chain family member 3	mRNA
chr2	230820520	230820620	16	+	135	CAB39	calcium binding protein 39	mRNA
chr1	162529161	162529232	16	+	149	UHMK1	U2AF homology motif kinase 1	mRNA
chr1	154582094	154582153	16	-	151	ADAR	adenosine deaminase RNA specific	mRNA
chr1	92390038	92390126	16	-	158	RPAP2	RNA polymerase II associated protein 2	mRNA
chr17	57680437	57680528	16	+	194	MSI2	musashi RNA binding protein 2	mRNA
chr1	207359712	207359930	16	+	219	CD55	CD55 molecule	mRNA
chrX	153696005	153696160	16	+	224	SLC6A8	solute carrier family 6 member 8	mRNA
chr21	25880867	25880908	16	-	231	APP	amyloid beta precursor protein	mRNA
chr8	99887539	99887618	15	-	121	COX6C	cytochrome c oxidase subunit 6C	mRNA
chr10	96527212	96527267	15	-	56	TM9SF3	transmembrane 9 superfamily member 3	mRNA
chr10	96552927	96552991	15	-	65	TM9SF3	transmembrane 9 superfamily member 3	mRNA
chr14	55637276	55637348	15	+	73	KTN1	kinectin 1	mRNA
chr14	55648033	55648115	15	+	83	KTN1	kinectin 1	mRNA
chr3	121717032	121717099	15	-	99	GOLGB1	golgin B1	mRNA
chr12	6236191	6236275	15	+	102	CD9	CD9 molecule	mRNA

chr1	119966375	119966482	15	-	108	NOTCH2	notch receptor 2	mRNA
chr18	46091708	46091816	15	-	109	ATP5F1A	ATP synthase F1 subunit alpha	mRNA
chr22	50280905	50280973	15	-	109	PLXNB2	plexin B2	mRNA
chr5	141579116	141579196	15	-	110	DIAPH1	diaphanous related formin 1	mRNA
chr2	241256288	241256335	15	-	115	HDLBP	high density lipoprotein binding protein	mRNA
chr1	1053841	1053894	15	+	146	AGRN	agrin	mRNA
chr17	63766027	63766045	15	-	149	CCDC47	coiled-coil domain containing 47	mRNA
chr19	38721537	38721688	15	+	152	ACTN4	actinin alpha 4	mRNA
chr3	49968148	49968224	15	+	187	RBM6	RNA binding motif protein 6	mRNA
chr12	6943829	6943878	15	+	63	C12orf57		lncRNA
chr8	124999328	124999361	15	+	119	SQLE	squalene epoxidase	mRNA
chr12	102311632	102311715	15	+	87			no annotation
chr22	17026779	17026824	15	-	89			no annotation
chr16	90221565	90221698	15	+	134			no annotation
chr1	77745527	77745577	15	+	56	USP33	ubiquitin specific peptidase 33	mRNA
chr4	158987520	158987569	15	-	59	C4orf45	chromosome 4 open reading frame 45	mRNA
chr1	146074581	146074636	15	-	59	NBPF10	NBPF member 10	mRNA
chr8	30414271	30414315	15	+	60	RBPMS	RNA binding protein, mRNA processing factor	mRNA
chr17	59839295	59839388	15	+	110	VMP1	vacuole membrane protein 1	mRNA
chr1	234407990	234408035	15	-	111	TARBP1	TAR (HIV-1) RNA binding protein 1	mRNA
chr18	37075114	37075177	15	-	123	KIAA1328	KIAA1328	mRNA
chr5	171406571	171406641	15	+	148	NPM1	nucleophosmin 1	mRNA
chr8	51839122	51839170	15	-	152	PCMTD1	protein-L-isoaspartate (D-aspartate) O-methyltransferase domain containing 1	mRNA
chr19	36214134	36214146	15	-	181	ZNF565	zinc finger protein 565	mRNA
chr19	36214283	36214314	15	-	181	ZNF565	zinc finger protein 565	mRNA
chr8	70646339	70646410	15	-	99	LACTB2-AS1		lncRNA
chr3	52688892	52688951	15	+	63	SNORD136		snoRNA
chr2	202278115	202278191	15	+	78	SNORD70B		snoRNA
chr17	7576885	7576962	15	+	103	SNORD10		snoRNA
chr11	46762375	46762420	15	-	123	SNORD67		snoRNA
chr11	811688	811814	15	+	149	SNORA52		snoRNA
chr3	845583	845620	15	-	61	LINC01266		lncRNA
chr1	93338779	93338843	15	-	134	CCDC18-AS1		lncRNA
chr8	125022234	125022280	15	+	51	SQLE	squalene epoxidase	mRNA
chr20	51598218	51598266	15	-	56	ATP9A	ATPase phospholipid transporting 9A	mRNA
chr1	154979119	154979174	15	+	60	CKS1B	CDC28 protein kinase regulatory subunit 1B	mRNA
chr12	6238009	6238057	15	+	61	CD9	CD9 molecule	mRNA
chr12	123597683	123597731	15	+	61	TMED2	transmembrane p24 trafficking protein 2	mRNA
chr12	13216629	13216689	15	+	61	EMP1	epithelial membrane protein 1	mRNA
chr1	154584489	154584544	15	-	64	ADAR	adenosine deaminase RNA specific	mRNA
chr2	85321521	85321587	15	-	73	TGOLN2	trans-golgi network protein 2	mRNA
chr8	61624730	61624802	15	-	73	ASPH	aspartate beta-hydroxylase	mRNA
chr2	8858183	8858256	15	-	74	MBOAT2	membrane bound O-acyltransferase domain containing 2	mRNA
chr1	171593292	171593371	15	+	80	PRRC2C	proline rich coiled-coil 2C	mRNA
chr1	169131611	169131679	15	+	82	ATP1B1	ATPase Na+/K+ transporting subunit beta 1	mRNA
chr9	86027611	86027653	15	-	84	GOLM1	golgi membrane protein 1	mRNA
chr3	49533574	49533641	15	+	86	DAG1	dystroglycan 1	mRNA
chr12	8941137	8941213	15	-	90	M6PR	mannose-6-phosphate receptor	mRNA
chr1	42926291	42926380	15	-	91	SLC2A1	solute carrier family 2 member 1	mRNA
chr6	109367810	109367898	15	-	93	CD164	CD164 molecule	mRNA
chr8	100919778	100919871	15	-	94	YWHAZ	tyrosine 3-monooxygenase/tryptophan 5-monooxygenase activation protein zet	mRNA
chr11	95131275	95131372	15	+	99	ENDOD1	endonuclease domain containing 1	mRNA
chr12	95658318	95658419	15	-	102	NTN4	netrin 4	mRNA
chr20	47655646	47655753	15	+	108	NCOA3	nuclear receptor coactivator 3	mRNA
chr16	15413126	15413227	15	-	113	MPV17L	MPV17 mitochondrial membrane protein-like	mRNA
chr15	40872540	40872666	15	-	127	RHOV	ras homolog family member V	mRNA
chr6	158632372	158632489	15	+	130	TMEM181	transmembrane protein 181	mRNA
chr15	89197333	89197460	15	+	131	ABHD2	abhydrolase domain containing 2	mRNA
chr3	196348478	196348595	15	-	131	UBXN7	UBX domain protein 7	mRNA
chr3	107807884	107807937	15	+	136	BBX	BBX high mobility group box domain containing	mRNA
chr2	227559186	227559225	15	+	140	AGFG1	ArfGAP with FG repeats 1	mRNA
chr9	105390049	105390189	15	+	141	SLC44A1	solute carrier family 44 member 1	mRNA
chr12	53040477	53040628	15	+	152	EIF4B	eukaryotic translation initiation factor 4B	mRNA
chr18	9400393	9400561	15	+	169	TWSG1	twisted gastrulation BMP signaling modulator 1	mRNA

chr7	90414389	90414573	15	+	187	CLDN12	claudin 12	mRNA
chr2	208236513	208236704	15	-	201	IDH1	isocitrate dehydrogenase (NADP(+)) 1	mRNA
chr16	71928745	71928803	15	+	221	IST1	IST1 factor associated with ESCRT-III	mRNA
chr16	56362420	56362506	15	-	239	AMFR	autocrine motility factor receptor	mRNA
chr16	85801204	85801278	14	+	75	COX4I1	cytochrome c oxidase subunit 4I1	mRNA
chr17	50750502	50750574	14	+	193	LUC7L3	LUC7 like 3 pre-mRNA splicing factor	mRNA
chr17	38850123	38850214	14	-	92	RPL23	ribosomal protein L23	mRNA
chr10	96551371	96551411	14	-	41	TM9SF3	transmembrane 9 superfamily member 3	mRNA
chr3	194430272	194430315	14	-	44	ATP13A3	ATPase 13A3	mRNA
chr2	218238792	218238850	14	+	59	ARPC2	actin related protein 2/3 complex subunit 2	mRNA
chr12	52518102	52518161	14	-	60	KRT5	keratin 5	mRNA
chr2	222930749	222930805	14	+	63	ACSL3	acyl-CoA synthetase long chain family member 3	mRNA
chr9	98015363	98015426	14	+	64	ANP32B	acidic (leucine-rich) nuclear phosphoprotein 32 family, member B	mRNA
chr8	125007423	125007487	14	+	65	SQLE	squalene epoxidase	mRNA
chr2	182736248	182736320	14	+	73	DNAJC10	DnaJ heat shock protein family (Hsp40) member C10	mRNA
chr5	135346009	135346057	14	-	76	MACROH2A1	macroH2A.1 histone	mRNA
chrX	53562142	53562192	14	-	83	HUWE1	HECT, UBA and WWE domain containing E3 ubiquitin protein ligase 1	mRNA
chr3	31625992	31626074	14	+	83	STT3B	STT3 oligosaccharyltransferase complex catalytic subunit B	mRNA
chr6	31626771	31626862	14	+	92	PRRC2A	proline rich coiled-coil 2A	mRNA
chr15	98942922	98943021	14	+	100	IGF1R	insulin like growth factor 1 receptor	mRNA
chr3	53229006	53229107	14	-	102	TKT	transketolase	mRNA
chr1	119969578	119969687	14	-	110	NOTCH2	notch receptor 2	mRNA
chr10	93369649	93369763	14	-	115	MYOF	myoferlin	mRNA
chr5	141576755	141576817	14	-	117	DIAPH1	diaphanous related formin 1	mRNA
chr11	88309202	88309276	14	-	119	CTSC	cathepsin C	mRNA
chr12	21637070	21637194	14	-	125	LDHB	lactate dehydrogenase B	mRNA
chr11	1761323	1761468	14	-	146	CTSD	cathepsin D	mRNA
chr18	743259	743285	14	-	157	YES1	YES proto-oncogene 1	mRNA
chr1	156320864	156321025	14	-	162	CCT3	chaperonin containing TCP1 subunit 3	mRNA
chr3	9249370	9249449	14	-	144	SRGAP3	SLIT-ROBO Rho GTPase activating protein 3	mRNA
chr8	124999365	124999482	14	+	118	SQLE	squalene epoxidase	mRNA
chr4	25200598	25200715	14	-	118			no annotation
chr15	38851833	38851908	14	+	122			no annotation
chr8	11807158	11807199	14	+	45	FDFT1	farnesyl-diphosphate farnesyltransferase 1	mRNA
chr15	100912467	100912519	14	+	53	ALDH1A3	aldehyde dehydrogenase 1 family member A3	mRNA
chr1	86350440	86350485	14	+	68	ODF2L	outer dense fiber of sperm tails 2 like	mRNA
chr5	17222728	17222802	14	+	75	BASP1	brain abundant, membrane attached signal protein 1	mRNA
chr17	46938853	46938923	14	+	79	GOSR2	golgi SNAP receptor complex member 2	mRNA
chr2	121479747	121479785	14	-	81	CLASP1	cytoplasmic linker associated protein 1	mRNA
chr1	207769520	207769610	14	+	91	CD46		mRNA
chr1	70223407	70223501	14	+	96	SRSF11	serine and arginine rich splicing factor 11	mRNA
chr8	42534637	42534670	14	-	97	SLC20A2	solute carrier family 20 member 2	mRNA
chr16	81626446	81626548	14	+	103	CMIP	c-Maf inducing protein	mRNA
chr2	197419895	197419927	14	-	105	SF3B1	splicing factor 3b subunit 1	mRNA
chr13	110899432	110899486	14	-	106	ANKRD10	ankyrin repeat domain 10	mRNA
chr16	87738382	87738462	14	-	116	KLHDC4	kelch domain containing 4	mRNA
chr19	36213986	36214110	14	-	125	ZNF565	zinc finger protein 565	mRNA
chr6	142934904	142935012	14	-	126	HIVEP2	HIVEP zinc finger 2	mRNA
chr5	32441433	32441499	14	-	140	ZFR	zinc finger RNA binding protein	mRNA
chr10	68755171	68755239	14	+	69	SNORD98		snoRNA
chr16	71758401	71758482	14	-	82	SNORD71		snoRNA
chr14	103337844	103337937	14	+	132	SNORA28		snoRNA
chr17	7574776	7574847	14	+	136	SNORA48		snoRNA
chr21	45602624	45602696	14	+	74	LINC01694		lncRNA
chr3	128620228	128620289	14	-	62	RPN1	ribophorin I	mRNA
chr1	159918550	159918616	14	-	67	TAGLN2	transgelin 2	mRNA
chr12	13216525	13216594	14	+	70	EMP1	epithelial membrane protein 1	mRNA
chr11	110231533	110231580	14	-	71	RDX	radixin, transcript variant 4	mRNA
chr1	32365343	32365411	14	-	71	BSDC1	BSD domain containing 1	mRNA
chr2	112117306	112117366	14	+	76	TMEM87B	transmembrane protein 87B	mRNA
chr10	119168246	119168312	14	-	77	PRDX3	peroxiredoxin 3	mRNA
chr20	33854167	33854216	14	+	78	CHMP4B	charged multivesicular body protein 4B	mRNA
chr10	72011415	72011492	14	+	78	CHST3	carbohydrate (chondroitin 6) sulfotransferase 3	mRNA
chr12	909981	910059	14	+	79	WNK1	WNK lysine deficient protein kinase 1	mRNA

chr5	6670993	6671071	14	+	79	SRD5A1	steroid 5 alpha-reductase 1	mRNA
chr19	42377408	42377490	14	+	83	MEGF8	multiple EGF like domains 8	mRNA
chr3	128070778	128070862	14	+	85	SEC61A1	SEC61 translocon alpha 1 subunit	mRNA
chr20	51599551	51599617	14	-	86	ATP9A	ATPase phospholipid transporting 9A	mRNA
chr9	112221333	112221408	14	-	92	PTBP3	polypyrimidine tract binding protein 3	mRNA
chr17	50090385	50090479	14	+	97	ITGA3	integrin subunit alpha 3	mRNA
chr5	10435408	10435473	14	+	100	MARCHF6	membrane associated ring-CH-type finger 6	mRNA
chr18	63598498	63598551	14	+	100	SERPINB13	serpin family B member 13	mRNA
chr3	33144209	33144308	14	+	100	CRTAP	cartilage associated protein	mRNA
chr3	182942706	182942775	14	-	102	DCUN1D1	defective in cullin neddylation 1 domain containing 1	mRNA
chr2	175073457	175073559	14	-	103	SF3B1	splicing factor 3b subunit 1	mRNA
chrX	103358303	103358366	14	+	106	TCEAL9	transcription elongation factor A like 9	mRNA
chr19	45609519	45609569	14	-	107	EML2	EMAP like 2	mRNA
chr1	171593138	171593201	14	+	107	PRRC2C	proline rich coiled-coil 2C	mRNA
chr14	24308094	24308182	14	+	114	NOP9	NOP9 nucleolar protein	mRNA
chr6	42101268	42101373	14	-	118	C6orf132	chromosome 6 open reading frame 132	mRNA
chr5	10264741	10264853	14	+	118	CCT5	chaperonin containing TCP1 subunit 5	mRNA
chr2	197503263	197503342	14	+	120	HSPE1	heat shock protein family E	mRNA
chr1	116987617	116987665	14	+	122	PTGFRN	prostaglandin F2 receptor inhibitor	mRNA
chr6	31829874	31829979	14	+	122	HSPA1B	heat shock 70kDa protein 1B	mRNA
chr2	168770330	168770394	14	+	136	CERS6	ceramide synthase 6	mRNA
chr3	172820990	172821050	14	+	141	ECT2	epithelial cell transforming 2	mRNA
chr3	49533460	49533573	14	+	142	DAG1	dystroglycan 1	mRNA
chr17	35266116	35266162	14	+	144	SLFN5	schlafen family member 5	mRNA
chr3	49359959	49360096	14	-	144	RHOA	ras homolog family member A	mRNA
chr10	70879428	70879539	14	+	153	SGPL1	sphingosine-1-phosphate lyase 1	mRNA
chr14	105470511	105470541	14	+	160	MTA1	metastasis associated 1	mRNA
chr12	110721031	110721088	13	-	145	PPP1CC	protein phosphatase 1 catalytic subunit gamma	mRNA
chr18	743065	743097	13	-	37	YES1	YES proto-oncogene 1	mRNA
chr19	41292244	41292286	13	+	61	HNRNPUL1	heterogeneous nuclear ribonucleoprotein U like 1	mRNA
chr5	96896796	96896860	13	+	68	ERAP2	endoplasmic reticulum aminopeptidase 2	mRNA
chr10	32911885	32911933	13	-	80	ITGB1	integrin subunit beta 1	mRNA
chr17	58005825	58005909	13	-	92	SRSF1	serine and arginine rich splicing factor 1	mRNA
chr12	21646916	21647016	13	-	101	LDHB	lactate dehydrogenase B	mRNA
chr7	2379133	2379242	13	+	110	EIF3B	eukaryotic translation initiation factor 3 subunit B	mRNA
chr6	43159870	43159957	13	+	155	PTK7	protein tyrosine kinase 7 (inactive)	mRNA
chr14	20455913	20456101	13	+	189	APEX1	apurinic/apyrimidinic endodeoxyribonuclease 1	mRNA
chr19	40374518	40374620	13	+	198	PLD3	phospholipase D family member 3	mRNA
chr3	9249263	9249347	13	-	85	SRGAP3	SLIT-ROBO Rho GTPase activating protein 3	mRNA
chr3	112640729	112640814	13	-	86	CCDC80	coiled-coil domain containing 80	mRNA
chr4	11477326	11477375	13	-	63			no annotation
chr13	109270414	109270445	13	-	110			no annotation
chr11	118921049	118921108	13	-	124			no annotation
chr11	35194842	35194883	13	+	49	CD44	CD44 molecule	mRNA
chr8	115570800	115570864	13	-	67	TRPS1	transcriptional repressor GATA binding 1	mRNA
chr15	80444536	80444591	13	+	80	ARNT2	aryl hydrocarbon receptor nuclear translocator 2	mRNA
chr9	76175248	76175284	13	+	82	PCSK5	proprotein convertase subtilisin/kexin type 5	mRNA
chr16	85706687	85706764	13	-	103	C16orf74	chromosome 16 open reading frame 74	mRNA
chr3	122014951	122014986	13	-	129	ILDR1	immunoglobulin like domain containing receptor 1	mRNA
chr9	33347717	33347807	13	-	133	NFX1	nuclear transcription factor, X-box binding 1	mRNA
chr7	23529570	23529613	13	-	134	TRA2A	transformer 2 alpha homolog	mRNA
chrX	71547730	71547789	13	+	166	OGT	O-linked N-acetylglucosamine (GlcNAc) transferase	mRNA
chrX	74122491	74122543	13	+	54	FTX		lncRNA
chr3	186787376	186787433	13	+	58	SNORA63		snoRNA
chr3	52691394	52691445	13	+	68	SNORD19C		snoRNA
chr2	202276437	202276513	13	+	84	SNORD70		snoRNA
chr9	33952769	33952843	13	-	104	SNORD121A		snoRNA
chr11	8685437	8685565	13	+	135	SNORA3B		snoRNA
chr14	53949961	53950002	13	+	49	BMP4	bone morphogenetic protein 4	mRNA
chr11	128460108	128460173	13	-	74	ETS1	ETS proto-oncogene 1	mRNA
chr11	66003930	66004005	13	+	76	BANF1	barrier to autointegration factor 1	mRNA
chr20	44907568	44907645	13	+	78	YWHAB	tyrosine 3-monooxygenase/tryptophan 5-monooxygenase activation protein bet	mRNA
chr20	44908209	44908286	13	+	82	YWHAB	tyrosine 3-monooxygenase/tryptophan 5-monooxygenase activation protein be	mRNA
chr7	106090638	106090675	13	-	82	SYPL1	synaptophysin like 1	mRNA

chr9	94460683	94460716	13	+	82	MFSD14B	major facilitator superfamily domain containing 14B	mRNA
chr1	211573575	211573636	13	-	83	SLC30A1	solute carrier family 30 (zinc transporter), member 1	mRNA
chr14	49894637	49894720	13	+	84	ARF6	ADP-ribosylation factor 6	mRNA
chr20	51599806	51599882	13	-	85	ATP9A	ATPase phospholipid transporting 9A	mRNA
chr7	116559524	116559584	13	+	86	CAV1	caveolin 1	mRNA
chr9	127940947	127941010	13	-	89	FAM102A	family with sequence similarity 102 member A	mRNA
chr2	3515579	3515619	13	-	90	ADI1	acireductone dioxygenase 1	mRNA
chr15	42210686	42210761	13	-	94	TMEM87A	transmembrane protein 87A	mRNA
chr3	152463715	152463809	13	+	95	MBNL1	muscleblind like splicing regulator 1	mRNA
chr14	63684982	63685058	13	-	96	SGPP1	sphingosine-1-phosphate phosphatase 1	mRNA
chr1	154583289	154583356	13	-	96	ADAR	adenosine deaminase RNA specific	mRNA
chr1	225496319	225496418	13	-	100	ENAH	ENAH actin regulator	mRNA
chr2	241352098	241352127	13	+	104	SEPTIN2	septin 2	mRNA
chr19	48615343	48615430	13	-	105	RPL18	ribosomal protein L18	mRNA
chr1	144440487	144440563	13	-	108	NBPF15	NBPF member 15	mRNA
chr19	44663347	44663381	13	+	109	PVR	PVR cell adhesion molecule	mRNA
chr4	183639944	183640023	13	-	110	RWDD4	RWD domain containing 4	mRNA
chr11	95130993	95131097	13	+	111	ENDOD1	endonuclease domain containing 1	mRNA
chrX	20152066	20152177	13	-	112	RPS6KA3	ribosomal protein S6 kinase A3	mRNA
chr22	36226701	36226744	13	-	114	APOL2	apolipoprotein L2	mRNA
chrX	153700728	153700844	13	-	117	BCAP31	B cell receptor associated protein 31	mRNA
chr12	50475976	50476023	13	+	119	LARP4	La ribonucleoprotein 4	mRNA
chr15	65888516	65888587	13	+	122	RAB11A	RAB11A, member RAS oncogene family	mRNA
chr5	256542	256662	13	+	122	SDHA	succinate dehydrogenase complex flavoprotein subunit A	mRNA
chr12	48938717	48938787	13	-	122	ARF3	ADP ribosylation factor 3	mRNA
chr11	130877273	130877313	13	-	127	SNX19	sorting nexin 19	mRNA
chr1	29326258	29326328	13	+	128	PTPRU	protein tyrosine phosphatase receptor type U	mRNA
chr19	47209809	47209918	13	+	137	SAE1	SUMO1 activating enzyme subunit 1	mRNA
chr3	48693787	48693823	13	-	137	IP6K2	inositol hexakisphosphate kinase 2	mRNA
chr17	42314563	42314571	13	-	153	STAT3	signal transducer and activator of transcription 3	mRNA
chr5	175959608	175959772	13	-	165	THOC3	THO complex 3	mRNA
chr15	65887805	65887934	12	+	244	RAB11A	RAB11A, member RAS oncogene family	mRNA
chr1	39568914	39568939	12	-	39	PABPC4	poly(A) binding protein cytoplasmic 4	mRNA
chr8	61643385	61643433	12	-	49	ASPH	aspartate beta-hydroxylase	mRNA
chr3	105552143	105552182	12	+	57	ALCAM	activated leukocyte cell adhesion molecule	mRNA
chr8	123209390	123209442	12	+	59	FAM83A	family with sequence similarity 83 member A	mRNA
chr12	103941467	103941527	12	+	61	HSP90B1	heat shock protein 90 beta family member 1	mRNA
chr14	61740512	61740575	12	+	64	HIF1A	hypoxia inducible factor 1 subunit alpha inhibitor	mRNA
chr4	186600298	186600360	12	-	77	FAT1	FAT atypical cadherin 1	mRNA
chr5	141578224	141578300	12	-	77	DIAPH1	diaphanous related formin 1	mRNA
chr8	39082991	39083073	12	+	83	ADAM9	ADAM metallopeptidase domain 9	mRNA
chr3	53228055	53228149	12	-	95	TKT	transketolase	mRNA
chr2	43963612	43963655	12	-	95	LRPPRC	leucine rich pentatricopeptide repeat containing	mRNA
chr1	156333546	156333638	12	-	98	CCT3	chaperonin containing TCP1 subunit 3	mRNA
chr3	105547192	105547253	12	+	106	ALCAM	activated leukocyte cell adhesion molecule	mRNA
chr5	133096097	133096204	12	+	108	HSPA4	heat shock protein family A (Hsp70) member 4	mRNA
chr22	45535244	45535285	12	+	109	FBLN1	fibulin 1	mRNA
chr17	42999932	43000102	12	+	171	RPL27	ribosomal protein L27	mRNA
chr11	1759015	1759087	12	-	73	CSTD	cathepsin D	mRNA
chr21	17707167	17707213	12	-	60			no annotation
chr5	80651419	80651460	12	-	42	DHFR	dihydrofolate reductase	mRNA
chr2	177261848	177261909	12	-	64	NFE2L2	nuclear factor, erythroid 2 like 2	mRNA
chr1	70226461	70226509	12	+	65	SRSF11	serine and arginine rich splicing factor 11	mRNA
chr11	71477302	71477338	12	+	69	NADSYN1	NAD synthetase 1	mRNA
chr14	21263136	21263206	12	-	71	HNRNPC	heterogeneous nuclear ribonucleoprotein C	mRNA
chr1	151213417	151213455	12	+	73	PIP5K1A	phosphatidylinositol-4-phosphate 5-kinase type 1 alpha	mRNA
chr22	49573086	49573136	12	+	78	C22orf34	chromosome 22 open reading frame 34	mRNA
chr14	21262277	21262335	12	-	84	HNRNPC	heterogeneous nuclear ribonucleoprotein C	mRNA
chr12	110049571	110049636	12	+	84	C12orf76	chromosome 12 open reading frame 76	mRNA
chr6	151948765	151948842	12	+	85	ESR1	estrogen receptor 1	mRNA
chr2	233702907	233702953	12	+	86	UGT1A8	UDP glucuronosyltransferase 1 family, polypeptide A8	mRNA
chr7	6700343	6700390	12	-	95	ZNF12	zinc finger protein 12	mRNA
chr19	47719983	47720037	12	+	114	EHD2	EH domain containing 2	mRNA
chr20	34073179	34073234	12	+	120	RALY	RALY heterogeneous nuclear ribonucleoprotein	mRNA

chr2	241507	241559	12	-	121	SH3YL1	SH3 and SYLF domain containing 1	mRNA
chr19	36213945	36213984	12	-	122	ZNF565	zinc finger protein 565	mRNA
chr22	16980486	16980533	12	+	127	GAB4	GRB2 associated binding protein family member 4	mRNA
chr21	33530392	33530431	12	-	145	GART	phosphoribosylglycinamide formyltransferase, phosphoribosylglycinamide syn	mRNA
chr15	101966530	101966573	12	+	132	WASH3P		pseudo
chrX	73802053	73802123	12	+	73	TSIX		lncRNA
chr2	241351991	241352033	12	+	44	SEPTIN2	septin 2	mRNA
chr6	122443503	122443550	12	-	48	SERINC1	serine incorporator 1	mRNA
chr3	41239941	41239986	12	+	48	CTNNB1	catenin beta 1	mRNA
chr5	314621	314669	12	+	49	PDCD6	programmed cell death 6	mRNA
chr16	19310069	19310105	12	-	55	CLEC19A	C-type lectin domain containing 19A	mRNA
chr9	98015556	98015615	12	+	60	ANP32B	acidic (leucine-rich) nuclear phosphoprotein 32 family, member B	mRNA
chr12	53479596	53479655	12	+	60	PCBP2	poly(rC) binding protein 2	mRNA
chr1	119912491	119912556	12	-	66	NOTCH2	notch receptor 2	mRNA
chr11	34100070	34100141	12	+	72	CAPRIN1	cell cycle associated protein 1	mRNA
chr6	113861101	113861164	12	+	73	MARCKS	myristoylated alanine-rich protein kinase C substrate	mRNA
chr15	100916292	100916364	12	+	74	ALDH1A3	aldehyde dehydrogenase 1 family member A3	mRNA
chr15	73560284	73560343	12	-	79	NPTN	neuroplastin	mRNA
chr12	84860988	84861057	12	-	82	SLC6A15	solute carrier family 6 member 15	mRNA
chr9	128694654	128694723	12	+	86	SET	SET nuclear proto-oncogene	mRNA
chr10	96519760	96519839	12	-	87	TM9SF3	transmembrane 9 superfamily member 3	mRNA
chr12	125030942	125031025	12	+	90	BRI3BP	BRI3 binding protein	mRNA
chr18	30992356	30992431	12	-	91	DSC3	desmocollin 3	mRNA
chr20	37241527	37241619	12	+	93	RPN2	ribophorin II	mRNA
chr8	142784896	142784990	12	-	95	LY6D	lymphocyte antigen 6 complex, locus D	mRNA
chr14	77508393	77508478	12	-	97	SPTLC2	serine palmitoyltransferase long chain base subunit 2	mRNA
chr2	170993414	170993442	12	-	99	TLK1	tousled like kinase 1	mRNA
chr12	6238060	6238087	12	+	100	CD9	CD9 molecule	mRNA
chr21	17569286	17569357	12	+	101	CXADR	CXADR Ig-like cell adhesion molecule	mRNA
chr12	26973746	26973844	12	-	104	TM7SF3	transmembrane 7 superfamily member 3	mRNA
chr20	44908022	44908124	12	+	108	YWHAB	tyrosine 3-monooxygenase/tryptophan 5-monooxygenase activation protein bet	mRNA
chr1	116987216	116987315	12	+	113	PTGFRN	prostaglandin F2 receptor inhibitor	mRNA
chr11	88293905	88293990	12	-	115	CTSC	cathepsin C	mRNA
chr9	136007318	136007413	12	-	118	NACC2	NACC family member 2	mRNA
chr8	6757613	6757716	12	+	119	AGPAT5	1-acylglycerol-3-phosphate O-acyltransferase 5	mRNA
chr6	2785265	2785390	12	+	126	WRNIP1	WRN helicase interacting protein 1	mRNA
chr14	103136919	103137049	12	+	131	TNFAIP2	TNF alpha induced protein 2	mRNA
chr2	177538566	177538664	12	+	139	AGPS	alkylglycerone phosphate synthase	mRNA
chr15	40857287	40857422	12	+	163	SPINT1	serine peptidase inhibitor	mRNA
chr4	108621950	108621997	11	+	55	RPL34	ribosomal protein L34	mRNA
chr11	62572583	62572612	11	-	30	EEF1G	eukaryotic translation elongation factor 1 gamma	mRNA
chr11	130141497	130141536	11	+	40	APLP2	amyloid beta precursor like protein 2	mRNA
chr3	196065442	196065486	11	-	45	TFRC	transferrin receptor	mRNA
chr11	33339349	33339396	11	+	48	HIPK3	homeodomain interacting protein kinase 3	mRNA
chr2	96300997	96301053	11	-	57	SNRNP200	small nuclear ribonucleoprotein U5 subunit 200	mRNA
chr6	85533739	85533780	11	-	62	SNX14	sorting nexin 14	mRNA
chr3	180962882	180962951	11	+	70	FXR1	FMR1 autosomal homolog 1	mRNA
chr3	37327702	37327779	11	+	78	GOLGA4	golgin A4	mRNA
chr2	27383995	27384073	11	-	79	PPM1G	protein phosphatase, Mg2+/Mn2+ dependent, 1G	mRNA
chr5	34938891	34938969	11	+	79	DNAJC21	DnaJ heat shock protein family (Hsp40) member C21	mRNA
chr3	149177889	149177964	11	-	80	CP	ceruloplasmin	mRNA
chr1	156135900	156135984	11	+	85	LMNA	lamin A/C	mRNA
chr12	110326389	110326475	11	+	87	ATP2A2	ATPase sarcoplasmic/endoplasmic reticulum Ca2+ transporting 2	mRNA
chr2	55871050	55871123	11	-	104	EFEMP1	EGF containing fibulin extracellular matrix protein 1	mRNA
chr21	31667257	31667375	11	+	119	SOD1	superoxide dismutase 1	mRNA
chr3	184334726	184334845	11	+	120	EIF4G1	eukaryotic translation initiation factor 4 gamma 1	mRNA
chr5	139321933	139322029	11	+	133	MATR3	matrin 3	mRNA
chr16	16379141	16379200	11	+	80	NPIPA7	nuclear pore complex interacting protein family, member A7	mRNA
chr15	98649436	98649510	11	+	85	IGF1R	insulin like growth factor 1 receptor	mRNA
chr22	15304326	15304385	11	+	71			no annotation
chr22	15283751	15283785	11	-	35			no annotation
chr2	172509110	172509146	11	-	40			no annotation
chr15	32185995	32186046	11	-	52			no annotation
chr6	2881296	2881331	11	-	76			no annotation

chr19	53158879	53158914	11	-	36	ZNF347	zinc finger protein 347	mRNA
chr6	47002365	47002404	11	-	40	ADGRF1	adhesion G protein-coupled receptor F1	mRNA
chr18	80162316	80162359	11	-	46	PARD6G	par-6 family cell polarity regulator gamma	mRNA
chr9	21892790	21892842	11	-	63	MTAP	methylthioadenosine phosphorylase	mRNA
chr2	233700887	233700949	11	+	66	UGT1A8	UDP glucuronosyltransferase 1 family, polypeptide A8	mRNA
chr1	205748089	205748145	11	-	67	NUCKS1	nuclear casein kinase and cyclin-dependent kinase substrate 1	mRNA
chr6	79201778	79201820	11	-	73	HMGN3	high mobility group nucleosomal binding domain 3	mRNA
chr14	96533445	96533521	11	+	77	PAPOLA	poly(A) polymerase alpha	mRNA
chr14	55283842	55283911	11	+	79	FBXO34	F-box protein 34	mRNA
chr22	45929771	45929843	11	-	88	WNT7B	Wnt family member 7B	mRNA
chr7	44005215	44005301	11	-	99	SPDYE1	speedy/RINGO cell cycle regulator family member E1	mRNA
chr2	227528930	227529021	11	+	104	AGFG1	ArfGAP with FG repeats 1	mRNA
chr1	1737226	1737315	11	-	109	SLC35E2A	solute carrier family 35 member E2A	mRNA
chr17	82146886	82146962	11	-	118	CCDC57	coiled-coil domain containing 57	mRNA
chr20	36137386	36137423	11	+	121	EPB41L1	erythrocyte membrane protein band 4.1 like 1	mRNA
chr14	19372669	19372714	11	+	55	LINC01297		lncRNA
chr15	25256607	25256646	11	-	78	SNHG14		lncRNA
chr5	139276212	139276324	11	+	149	SNORA74D		snoRNA
chr1	145594485	145594576	11	-	117	NBPF25P		pseudo
chr1	64106647	64106701	11	+	65	ROR1-AS1		lncRNA
chr2	8561720	8561763	11	+	88	LINC01814		lncRNA
chr1	134789	134831	11	-	103	LOC729737		lncRNA
chr2	66392055	66392149	11	-	180	LINC01873		lncRNA
chr9	88475726	88475761	11	-	36	SPIN1	spindlin 1	mRNA
chr22	46362665	46362704	11	-	41	CELSR1	cadherin EGF LAG seven-pass G-type receptor 1	mRNA
chr19	5206283	5206329	11	-	47	PTPRS	protein tyrosine phosphatase receptor type S	mRNA
chr5	177331689	177331736	11	-	48	LMAN2	lectin, mannose binding 2	mRNA
chr1	161071920	161071965	11	-	49	NECTIN4	nectin cell adhesion molecule 4	mRNA
chr3	196051058	196051108	11	-	51	TFRC	transferrin receptor	mRNA
chr5	157786562	157786610	11	-	52	CLINT1	clathrin interactor 1	mRNA
chr14	77510270	77510321	11	-	52	SPTLC2	serine palmitoyltransferase long chain base subunit 2	mRNA
chr1	235167390	235167433	11	-	53	ARID4B	AT-rich interaction domain 4B	mRNA
chr17	38727157	38727205	11	+	56	MLLT6	MLLT6, PHD finger containing	mRNA
chr19	35267898	35267944	11	+	56	LSR	lipolysis stimulated lipoprotein receptor	mRNA
chr18	673388	673443	11	+	56	TYMS	thymidylate synthetase	mRNA
chr17	78357136	78357184	11	-	57	SOCS3	suppressor of cytokine signaling 3	mRNA
chr11	118750996	118751053	11	-	59	DDX6	DEAD-box helicase 6	mRNA
chr8	41624003	41624039	11	+	61	GPAT4	glycerol-3-phosphate acyltransferase 4	mRNA
chr14	21210683	21210743	11	-	61	HNRNPC	heterogeneous nuclear ribonucleoprotein C	mRNA
chr20	32850149	32850209	11	+	62	MAPRE1	microtubule-associated protein, RP/EB family, member 1	mRNA
chr2	65088201	65088260	11	-	63	RAB1A	RAB1A, member RAS oncogene family	mRNA
chr3	122575035	122575098	11	+	65	DTX3L	deltex E3 ubiquitin ligase 3L	mRNA
chr5	97034651	97034713	11	+	65	LNPEP	leucyl and cystinyl aminopeptidase	mRNA
chr4	76160969	76161033	11	-	65	SCARB2	scavenger receptor class B member 2	mRNA
chr2	232857667	232857716	11	+	66	GIGYF2	GRB10 interacting GYF protein 2	mRNA
chr19	57863571	57863631	11	+	67	ZNF587	zinc finger protein 587	mRNA
chr13	48261714	48261772	11	+	67	ITM2B	integral membrane protein 2B	mRNA
chr17	61945216	61945275	11	-	67	MED13	mediator complex subunit 13	mRNA
chr2	200823017	200823084	11	+	68	BZW1	basic leucine zipper and W2 domains 1	mRNA
chr21	37202518	37202569	11	+	71	TTC3	tetratricopeptide repeat domain 3	mRNA
chr3	42646929	42646987	11	+	71	NKTR	natural killer cell triggering receptor	mRNA
chr7	75410828	75410876	11	+	71	TRIM73	tripartite motif containing 73	mRNA
chr3	191267690	191267732	11	+	72	UTS2B	Urotensin 2B	mRNA
chr8	97852431	97852494	11	+	74	LAPTM4B	lysosomal protein transmembrane 4 beta	mRNA
chr10	123165193	123165246	11	+	75	BUB3	BUB3 mitotic checkpoint protein	mRNA
chr12	56238757	56238801	11	-	76	ANKRD52	ankyrin repeat domain 52	mRNA
chr6	31830106	31830144	11	+	76	HSPA1B	heat shock 70kDa protein 1B	mRNA
chr17	50751514	50751525	11	+	76	LUC7L3	LUC7 like 3 pre-mRNA splicing factor	mRNA
chr1	30932178	30932254	11	-	77	PUM1	pumilio RNA binding family member 1	mRNA
chr16	23067892	23067957	11	-	77	USP31	ubiquitin specific peptidase 31	mRNA
chr16	1349381	1349458	11	-	78	TSR3	TSR3, 20S rRNA accumulation, homolog (S. cerevisiae)	mRNA
chr18	26016762	26016831	11	-	80	SS18	SS18 subunit of BAF chromatin remodeling complex	mRNA
chr3	194402828	194402896	11	-	82	ATP13A3	ATPase 13A3	mRNA
chr1	162523336	162523380	11	+	84	UHMK1	U2AF homology motif kinase 1	mRNA

chr6	79201513	79201599	11	-	87	HMGN3	high mobility group nucleosomal binding domain 3	mRNA
chr22	19037615	19037655	11	-	87	DGCR2	DiGeorge syndrome critical region gene 2	mRNA
chr3	58171753	58171840	11	+	88	FLNB	filamin B	mRNA
chr11	111293731	111293818	11	-	88	COLCA1	colorectal cancer associated 1	mRNA
chr12	92143942	92143988	11	-	95	BTG1	B-cell translocation gene 1, anti-proliferative	mRNA
chrX	154436247	154436342	11	+	96	ATP6AP1	ATPase H+ transporting accessory protein 1	mRNA
chr5	83054654	83054690	11	-	98	TMEM167A	transmembrane protein 167A	mRNA
chr4	168927458	168927508	11	+	98	PALLD	palladin	mRNA
chr9	113261343	113261413	11	+	101	SLC31A1	solute carrier family 31 (copper transporter), member 1	mRNA
chr11	125620892	125620988	11	+	101	STT3A	STT3 oligosaccharyltransferase complex catalytic subunit A	mRNA
chr15	98962557	98962568	11	+	104	IGF1R	insulin like growth factor 1 receptor	mRNA
chr10	74119630	74119708	11	+	106	VCL	vinculin	mRNA
chr3	195568836	195568941	11	-	107	APOD	apolipoprotein D	mRNA
chr10	103595294	103595395	11	-	108	SH3PXD2A	SH3 and PX domains 2A	mRNA
chr20	45816804	45816852	11	+	121	UBE2C	ubiquitin conjugating enzyme E2 C	mRNA
chr5	176375182	176375218	11	-	134	ARL10	ADP-ribosylation factor-like 10	mRNA
chr5	176375235	176375299	11	-	134	ARL10	ADP-ribosylation factor-like 10	mRNA
chr15	77046212	77046243	11	-	137	TSPAN3	tetraspanin 3	mRNA
chr15	77046283	77046348	11	-	137	TSPAN3	tetraspanin 3	mRNA
chr19	35658593	35658673	11	+	147	COX6B1	cytochrome c oxidase subunit 6B1	mRNA
chr11	62714889	62715003	11	-	159	HNRNPUL2	heterogeneous nuclear ribonucleoprotein U like 2	mRNA
chr19	48197135	48197224	11	+	105	ZSWIM9	zinc finger SWIM-type containing 9	mRNA
chr1	203869869	203869983	10	+	122	SNRPE	small nuclear ribonucleoprotein polypeptide E	mRNA
chr6	7585371	7585407	10	+	37	DSP	desmoplakin	mRNA
chr15	76935599	76935641	10	+	43	RCN2	reticulocalbin 2	mRNA
chr2	27383951	27383994	10	-	44	PPM1G	protein phosphatase, Mg2+/Mn2+ dependent, 1G	mRNA
chr2	232549134	232549165	10	+	46	TIGD1	tigger transposable element derived 1	mRNA
chr19	41281223	41281275	10	+	54	HNRNPUL1	heterogeneous nuclear ribonucleoprotein U like 1	mRNA
chr6	73782613	73782671	10	+	59	CD109	CD109 molecule	mRNA
chrX	53584214	53584268	10	-	66	HUWE1	HECT, UBA and WWE domain containing E3 ubiquitin protein ligase 1	mRNA
chr1	119968076	119968126	10	-	70	NOTCH2	notch receptor 2	mRNA
chr6	18258330	18258405	10	-	76	DEK	DEK proto-oncogene	mRNA
chr11	65624470	65624523	10	+	84	PCNX3	pecanex 3	mRNA
chr1	10403070	10403122	10	+	90	PGD	phosphogluconate dehydrogenase	mRNA
chr3	31622202	31622283	10	+	92	STT3B	STT3 oligosaccharyltransferase complex catalytic subunit B	mRNA
chr2	61877392	61877496	10	-	105	CCT4	chaperonin containing TCP1 subunit 4	mRNA
chr20	45670212	45670256	10	-	73	WFDC11	WAP four-disulfide core domain 11	mRNA
chr5	163235674	163235724	10	+	61			no annotation
chr16	67205324	67205360	10	+	49			no annotation
chr4	65858797	65858842	10	-	57			no annotation
chr15	38140499	38140553	10	+	68			no annotation
chr13	109270055	109270107	10	-	74			no annotation
chr15	91035528	91035587	10	-	79			no annotation
chr22	17028401	17028444	10	-	44			no annotation
chr11	70397293	70397348	10	-	65			no annotation
chr1	110939937	110940041	10	-	105			no annotation
chr3	185293013	185293046	10	+	37	MAP3K13	mitogen-activated protein kinase kinase kinase 13	mRNA
chr2	177249593	177249630	10	-	38	NFE2L2	nuclear factor, erythroid 2 like 2	mRNA
chr9	116612382	116612416	10	-	41	ASTN2	astrotactin 2	mRNA
chr11	32587104	32587144	10	+	43	EIF3M	eukaryotic translation initiation factor 3 subunit M	mRNA
chr3	54638639	54638694	10	+	56	CACNA2D3	calcium voltage-gated channel auxiliary subunit alpha2delta 3	mRNA
chr22	16977670	16977726	10	-	60	GAB4	GRB2 associated binding protein family member 4	mRNA
chr14	55581091	55581145	10	+	64	KTN1	kinectin 1	mRNA
chr1	223770200	223770254	10	+	71	CAPN2	calpain 2	mRNA
chr19	47721197	47721266	10	+	82	EHD2	EH domain containing 2	mRNA
chr7	74883112	74883166	10	-	87	STAG3L2	stromal antigen 3-like 2	mRNA
chr12	22668388	22668435	10	+	118	ETNK1	ethanolamine kinase 1	mRNA
chr14	76281481	76281515	10	-	54	RP11-516J2.1		lncRNA
chr10	100381881	100381952	10	-	72	OLMALINC	oligodendrocyte maturation-associated long intergenic non-coding RNA	lncRNA
chr12	50456571	50456641	10	+	74	SNORD133		snoRNA
chr15	84201552	84201605	10	-	110	GOLGA2P7		pseudo
chr10	42752103	42752139	10	-	38	LOC283028		lncRNA
chr8	116875635	116875683	10	-	59	RAD21-AS1		lncRNA
chr11	67603487	67603521	10	+	100	C11orf72		lncRNA

chr11	67603544	67603581	10	+	100	C11orf72		lncRNA
chr18	46669886	46669909	10	-	36	ST8SIA5	ST8 alpha-N-acetyl-neuraminide alpha-2,8-sialyltransferase 5	mRNA
chr2	241352048	241352083	10	+	36	SEPTIN2	septin 2	mRNA
chr11	101998391	101998422	10	-	36	CEP126	centrosomal protein 126	mRNA
chr19	11146895	11146931	10	-	38	SPC24	SPC24 component of NDC80 kinetochore complex	mRNA
chr11	111295163	111295201	10	-	41	COLCA1	colorectal cancer associated 1	mRNA
chr17	82239443	82239483	10	+	44	SLC16A3	solute carrier family 16 member 3	mRNA
chr16	68833852	68833897	10	+	47	CDH1	cadherin 1	mRNA
chr11	65637385	65637417	10	+	51	PCNX3	pecanex 3	mRNA
chr6	158632512	158632563	10	+	52	TMEM181	transmembrane protein 181	mRNA
chr1	111440983	111441032	10	-	52	WDR77	WD repeat domain 77	mRNA
chr7	16783676	16783727	10	+	54	TSPAN13	tetraspanin 13	mRNA
chr14	50905263	50905299	10	-	54	PYGL	glycogen phosphorylase L	mRNA
chr11	102396443	102396491	10	-	55	TMEM123	transmembrane protein 123	mRNA
chr14	52640795	52640849	10	-	55	ERO1A	endoplasmic reticulum oxidoreductase 1 alpha	mRNA
chr13	42320338	42320388	10	+	57	AKAP11	A kinase (PRKA) anchor protein 11	mRNA
chr16	89914005	89914063	10	+	59	MC1R	Multi-pass membrane protein (By similarity)	mRNA
chr5	141515877	141515910	10	-	60	DIAPH1	diaphanous related formin 1	mRNA
chr16	13239624	13239661	10	+	61	SHISA9	shisa family member 9	mRNA
chrX	118792292	118792341	10	+	63	IL13RA1	interleukin 13 receptor subunit alpha 1	mRNA
chr1	225790028	225790090	10	+	63	SRP9	signal recognition particle 9	mRNA
chr17	15539083	15539135	10	-	63	TVP23C	trans-golgi network vesicle protein 23 homolog C	mRNA
chr10	71302762	71302818	10	+	65	UNC5B	unc-5 netrin receptor B	mRNA
chr17	39997846	39997907	10	+	66	PSMD3	proteasome 26S subunit	mRNA
chr8	127417532	127417595	10	+	66	POU5F1B	POU class 5 homeobox 1B	mRNA
chr13	79553688	79553739	10	+	67	NDFIP2	Nedd4 family interacting protein 2	mRNA
chr8	53968046	53968112	10	-	67	TCEA1	transcription elongation factor A1	mRNA
chr15	98963268	98963314	10	+	68	IGF1R	insulin like growth factor 1 receptor	mRNA
chr15	98963319	98963335	10	+	68	IGF1R	insulin like growth factor 1 receptor	mRNA
chr1	203741174	203741216	10	+	69	ATP2B4	ATPase plasma membrane Ca2+ transporting 4	mRNA
chr19	36238579	36238623	10	+	70	ZNF146	zinc finger protein 146	mRNA
chr11	35809782	35809827	10	+	71	TRIM44	tripartite motif containing 44	mRNA
chr7	135928799	135928869	10	-	71	MTPN	myotrophin	mRNA
chr12	79774028	79774059	10	-	74	PPP1R12A	protein phosphatase 1 regulatory subunit 12A	mRNA
chr9	112221257	112221296	10	-	74	PTBP3	polypyrimidine tract binding protein 3	mRNA
chr9	112221297	112221330	10	-	74	PTBP3	polypyrimidine tract binding protein 3	mRNA
chr5	139683824	139683862	10	+	74	CXXC5	CXXC finger protein 5	mRNA
chr10	103595477	103595545	10	-	75	SH3PXD2A	SH3 and PX domains 2A	mRNA
chr7	44578998	44579045	10	-	76	TMED4	transmembrane p24 trafficking protein 4	mRNA
chr2	219183045	219183120	10	+	76	RETREG2	reticulophagy regulator family member 2	mRNA
chr2	28800356	28800416	10	+	77	PPP1CB	protein phosphatase 1 catalytic subunit beta	mRNA
chr3	23920020	23920096	10	+	77	RPL15	ribosomal protein L15	mRNA
chr3	197782640	197782716	10	+	77	FYTTD1	forty-two-three domain containing 1,	mRNA
chr1	26854728	26854793	10	+	78	ZDHHC18	zinc finger DHHC-type containing 18	mRNA
chr6	121449220	121449242	10	+	79	GJA1	gap junction protein, alpha 1, 43kDa	mRNA
chr8	18056471	18056500	10	-	79	ASAH1	N-acylsphingosine amidohydrolase 1	mRNA
chr11	61802091	61802162	10	-	80	FADS1	fatty acid desaturase 1	mRNA
chr14	103137285	103137337	10	+	81	TNFAIP2	TNF alpha induced protein 2	mRNA
chr3	10150804	10150845	10	+	84	VHL	von Hippel-Lindau tumor suppressor, E3 ubiquitin protein ligase	mRNA
chr5	72911366	72911447	10	+	85	TNPO1	transportin 1	mRNA
chr16	57664938	57664983	10	+	85	ADGRG1	adhesion G protein-coupled receptor G1	mRNA
chr3	98796216	98796256	10	-	86	DCBLD2	discoidin	mRNA
chr4	105969765	105969828	10	+	86	NPNT	nephronectin	mRNA
chr2	178505871	178505906	10	+	88	PLEKHA3	pleckstrin homology domain containing A3	mRNA
chr1	234606473	234606561	10	-	89	IRF2BP2	interferon regulatory factor 2 binding protein 2	mRNA
chr6	47624749	47624798	10	+	89	CD2AP	CD2-associated protein	mRNA
chr7	140453975	140454054	10	-	92	MKRN1	makorin ring finger protein 1	mRNA
chr4	127721473	127721545	10	+	93	INTU	inturned planar cell polarity protein	mRNA
chr1	24673052	24673097	10	+	98	SRRM1	serine and arginine repetitive matrix 1,	mRNA
chr19	18279779	18279850	10	-	99	JUND	jun D proto-oncogene	mRNA
chr11	118751384	118751438	10	-	100	DDX6	DEAD-box helicase 6	mRNA
chr12	53479446	53479547	10	+	102	PCBP2	poly(rC) binding protein 2	mRNA
chr14	74904234	74904268	10	+	108	RPS6KL1	ribosomal protein S6 kinase like 1	mRNA
chr2	214932104	214932195	10	-	113	ABCA12	ATP binding cassette subfamily A member 12	mRNA

chr12	1787976	1788099	10	+	124	ADIPOR2	adiponectin receptor 2	mRNA
chr1	156465700	156465776	10	-	131	MEF2D	myocyte enhancer factor 2D	mRNA
chr5	79325840	79325902	10	+	172	JMY	junction mediating and regulatory protein, p53 cofactor	mRNA
chr2	46378708	46378732	9	+	41	EPAS1	endothelial PAS domain protein 1	mRNA
chr6	137204331	137204372	9	-	42	IFNGR1	interferon gamma receptor 1	mRNA
chr12	110339669	110339708	9	+	45	ATP2A2	ATPase sarcoplasmic/endoplasmic reticulum Ca2+ transporting 2	mRNA
chr10	93401517	93401544	9	-	45	MYOF	myoferlin	mRNA
chr6	143502066	143502122	9	-	57	FUCA2	alpha-L-fucosidase 2	mRNA
chr6	143502007	143502063	9	-	60	FUCA2	alpha-L-fucosidase 2	mRNA
chr15	76947458	76947517	9	+	60	RCN2	reticulocalbin 2	mRNA
chr13	25096985	25097032	9	+	62	PABPC3	poly(A) binding protein, cytoplasmic 3	mRNA
chr12	22062371	22062434	9	+	64	CMAS	cytidine monophosphate N-acetylneuraminic acid synthetase	mRNA
chr10	74090097	74090121	9	+	100	VCL	vinculin	mRNA
chr2	61876943	61877052	9	-	110	CCT4	chaperonin containing TCP1 subunit 4	mRNA
chr4	108620579	108620600	9	+	25	RPL34	ribosomal protein L34	mRNA
chr4	25200554	25200577	9	-	41			no annotation
chr4	25200582	25200594	9	-	41			no annotation
chr14	75271735	75271763	9	-	48			no annotation
chr5	37795349	37795393	9	-	51			no annotation
chr15	38140074	38140137	9	+	86			no annotation
chr22	17070507	17070538	9	+	34			no annotation
chr18	54269102	54269146	9	-	49			no annotation
chr22	17027272	17027319	9	-	50			no annotation
chr12	102237900	102237937	9	+	53			no annotation
chr12	102296247	102296277	9	+	69			no annotation
chr15	29760020	29760052	9	+	33	TJP1	tight junction protein 1	mRNA
chr1	20775334	20775374	9	-	41	HP1BP3	heterochromatin protein 1 binding protein 3	mRNA
chr6	63658799	63658842	9	+	48	PHF3	PHD finger protein 3	mRNA
chr16	61725898	61725930	9	+	50	CDH8	cadherin 8	mRNA
chr14	55136212	55136262	9	+	51	LGALS3	galectin 3	mRNA
chr2	44229614	44229652	9	+	51	PPM1B	protein phosphatase, Mg2+/Mn2+ dependent 1B	mRNA
chr3	185933815	185933863	9	-	51	TRA2B	Transformer-2 protein homolog beta	mRNA
chr4	15358478	15358528	9	+	53	C1QTNF7	C1q and TNF related 7	mRNA
chr14	51644309	51644366	9	-	70	FRMD6	FERM domain containing 6	mRNA
chr5	180809779	180809834	9	-	73	MGAT1	alpha-1,3-mannosyl-glycoprotein 2-beta-N-acetylglucosaminyltransferase	mRNA
chr13	106558199	106558231	9	-	81	ARGLU1	arginine and glutamate rich 1	mRNA
chr11	82914102	82914154	9	+	84	DDIAS	DNA damage induced apoptosis suppressor	mRNA
chr14	67938739	67938821	9	+	86	RAD51B	RAD51 paralog B	mRNA
chr8	115552248	115552285	9	-	89	TRPS1	transcriptional repressor GATA binding 1	mRNA
chr2	169623034	169623091	9	+	93	PPIG	peptidylprolyl isomerase G	mRNA
chr17	47974808	47974861	9	+	94	CDK5RAP3	CDK5 regulatory subunit associated protein 3	mRNA
chr1	110344017	110344093	9	+	103	RBM15	RNA binding motif protein 15	mRNA
chr15	79925284	79925331	9	+	56	ST20-AS1	ST20 antisense RNA 1	lncRNA
chr2	80811844	80811893	9	-	57	AC084193.1		lncRNA
chr8	119246699	119246751	9	+	70	MAL2-AS1		lncRNA
chr2	101272936	101272974	9	-	48	SNORD89		snoRNA
chr9	19063655	19063695	9	-	133	SCARNA8		scaRNA
chr19	52389107	52389144	9	+	38	ZNF528-AS1		lncRNA
chr4	7776352	7776390	9	+	63	AFAP1-AS1		lncRNA
chrX	73802704	73802761	9	+	80	TSIX		lncRNA
chr11	108368220	108368251	9	+	34	ATM	ATM serine/threonine kinase,	mRNA
chr1	150996217	150996252	9	-	36	DHCR24	24-dehydrocholesterol reductase	mRNA
chr8	143655073	143655108	9	+	37	ZNF623	zinc finger protein 623	mRNA
chr3	49533725	49533761	9	+	38	DAG1	dystroglycan 1	mRNA
chr3	46669200	46669234	9	+	38	ALS2CL	ALS2 C-terminal like	mRNA
chrX	24076799	24076839	9	+	41	EIF2S3	eukaryotic translation initiation factor 2 subunit gamma	mRNA
chr5	1050407	1050448	9	-	42	SLC12A7	solute carrier family 12 member 7	mRNA
chr10	811232	811265	9	-	43	LARP4B	La ribonucleoprotein 4B	mRNA
chr1	179918715	179918759	9	+	45	TOR1AIP1	torsin 1A interacting protein 1	mRNA
chr19	11783066	11783103	9	-	45	ZNF441	zinc finger protein 441	mRNA
chr1	39004450	39004495	9	+	47	AKIRIN1	akirin 1	mRNA
chr19	42227864	42227909	9	-	47	ZNF526	zinc finger protein 526	mRNA
chr1	156465657	156465698	9	-	48	MEF2D	myocyte enhancer factor 2D	mRNA
chr15	98963173	98963220	9	+	48	IGF1R	insulin like growth factor 1 receptor	mRNA

chr11	119118428	119118471	9	+	49	C2CD2L	C2CD2 like	mRNA
chr6	73827914	73827960	9	+	49	CD109	CD109 molecule	mRNA
chr7	6402532	6402580	9	+	49	RAC1	Rac family small GTPase 1	mRNA
chr12	133019474	133019517	9	-	49	ZNF26	zinc finger protein 26	mRNA
chr8	61625891	61625937	9	-	50	ASPH	aspartate beta-hydroxylase	mRNA
chr11	33354647	33354685	9	+	52	HIPK3	homeodomain interacting protein kinase 3	mRNA
chr7	94668141	94668192	9	+	52	PEG10	paternally expressed 10	mRNA
chr16	2520097	2520145	9	+	53	ATP6V0C	ATPase H+ transporting V0 subunit c	mRNA
chr5	175959867	175959921	9	-	55	THOC3	THO complex 3	mRNA
chr2	26135155	26135193	9	+	56	RAB10	RAB10, member RAS oncogene family	mRNA
chr5	172952382	172952438	9	+	57	ERGIC1	endoplasmic reticulum-golgi intermediate compartment 1	mRNA
chr1	43623527	43623584	9	+	58	PTPRF	protein tyrosine phosphatase receptor type F	mRNA
chr1	153959176	153959233	9	-	58	SLC39A1	solute carrier family 39 member 1	mRNA
chr1	202891706	202891763	9	-	58	KLHL12	kelch like family member 12	mRNA
chr12	93506423	93506467	9	+	63	MRPL42	mitochondrial ribosomal protein L42	mRNA
chr19	44821367	44821414	9	+	64	BCAM	basal cell adhesion molecule (Lutheran blood group)	mRNA
chr12	122769834	122769895	9	+	68	DENR	density regulated re-initiation and release factor	mRNA
chr14	62109143	62109199	9	+	68	SYT16	synaptotagmin 16	mRNA
chr16	15409930	15409986	9	-	69	MPV17L	MPV17 mitochondrial membrane protein-like	mRNA
chr8	61502978	61503047	9	-	70	ASPH	aspartate beta-hydroxylase	mRNA
chr3	196829964	196830033	9	+	70	PAK2	p21 (RAC1) activated kinase 2	mRNA
chr1	16396082	16396129	9	+	70	SZRD1	SUZ RNA binding domain containing 1	mRNA
chr13	29509490	29509561	9	-	72	SLC7A1	solute carrier family 7 member 1	mRNA
chr17	68531254	68531325	9	+	74	PRKAR1A	protein kinase cAMP-dependent type I regulatory subunit alpha	mRNA
chr2	36548683	36548755	9	+	74	CRIM1	cysteine rich transmembrane BMP regulator 1	mRNA
chr16	81386420	81386472	9	+	75	GAN	gigaxonin	mRNA
chr20	45326979	45327053	9	-	75	SDC4	syndecan 4	mRNA
chr19	13141054	13141121	9	+	76	NACC1	nucleus accumbens associated 1, BEN and BTB (POZ) domain containing	mRNA
chr13	42321747	42321822	9	+	76	AKAP11	A kinase (PRKA) anchor protein 11	mRNA
chr1	150265889	150265942	9	-	77	APH1A	aph-1 homolog A, gamma-secretase subunit	mRNA
chr6	116495759	116495800	9	+	77	TRAPPC3L	trafficking protein particle complex 3 like	mRNA
chr15	65577432	65577473	9	-	79	HACD3	3-hydroxyacyl-CoA dehydratase 3	mRNA
chr4	98872828	98872910	9	-	83	EIF4E	eukaryotic translation initiation factor 4E	mRNA
chr5	138507332	138507413	9	-	83	ETF1	eukaryotic translation termination factor 1	mRNA
chr19	34227820	34227893	9	+	86	LSM14A	LSM14A mRNA processing body assembly factor	mRNA
chr9	136440858	136440946	9	-	90	SEC16A	SEC16 homolog A, endoplasmic reticulum export factor	mRNA
chr17	56938428	56938473	9	-	90	COIL	coilin	mRNA
chr6	34417458	34417547	9	-	90	RPS10	ribosomal protein S10	mRNA
chr9	6532735	6532801	9	-	93	GLDC	glycine dehydrogenase (decarboxylating)	mRNA
chr16	15411436	15411524	9	-	96	MPV17L	MPV17 mitochondrial membrane protein-like	mRNA
chr3	42646832	42646928	9	+	97	NKTR	natural killer cell triggering receptor	mRNA
chr21	31668576	31668638	9	+	104	SOD1	superoxide dismutase 1	mRNA
chr2	182781772	182781845	9	+	107	DNAJC10	DnaJ heat shock protein family (Hsp40) member C10	mRNA
chr11	10307021	10307189	9	+	169	ADM	adrenomedullin	mRNA
chr12	53446279	53446357	9	+	217	PRR13	proline rich 13	mRNA
chr18	31524702	31524727	8	+	26	DSG2	desmoglein 2	mRNA
chr14	102968328	102968358	8	-	32	CDC42BPB	CDC42 binding protein kinase beta	mRNA
chr7	128084811	128084847	8	+	37	SND1	staphylococcal nuclease and tudor domain containing 1	mRNA
chr7	128085710	128085746	8	+	39	SND1	staphylococcal nuclease and tudor domain containing 1	mRNA
chr1	88807737	88807775	8	+	45	PKN2	protein kinase N2	mRNA
chr10	93402231	93402275	8	-	45	MYOF	myoferlin	mRNA
chr3	128064876	128064885	8	+	60	SEC61A1	SEC61 translocon alpha 1 subunit	mRNA
chr2	44209283	44209325	8	+	61	PPM1B	protein phosphatase, Mg2+/Mn2+ dependent 1B	mRNA
chr19	36145805	36145859	8	+	71	CAPNS1	calpain small subunit 1	mRNA
chr15	58611031	58611107	8	-	77	ADAM10	ADAM metallopeptidase domain 10	mRNA
chr2	54631368	54631410	8	+	88	SPTBN1	spectrin beta, non-erythrocytic 1	mRNA
chr7	105110550	105110585	8	-	95	KMT2E	lysine methyltransferase 2E	mRNA
chr8	73293598	73293629	8	-	35	RPL7	ribosomal protein L7	mRNA
chr16	69977075	69977109	8	-	167	PDXDC2P-NPIPB14P		pseudo
chr7	54201561	54201593	8	-	33			no annotation
chr12	131292420	131292448	8	+	36			no annotation
chr9	66721325	66721371	8	-	53			no annotation
chr14	61657824	61657854	8	-	65			no annotation
chr12	102381991	102382052	8	-	68			no annotation

chr15	30345250	30345333	8	+	93			no annotation
chr12	102244690	102244724	8	+	61			no annotation
chr12	102237954	102237991	8	+	40			no annotation
chr12	102309648	102309690	8	+	43			no annotation
chr17	16786587	16786632	8	+	53			no annotation
chr12	102288493	102288548	8	+	63			no annotation
chr22	15303150	15303207	8	-	70			no annotation
chr1	179544488	179544524	8	-	38	AXDND1	axonemal dynein light chain domain containing 1	mRNA
chr22	46525532	46525569	8	-	40	CELSR1	cadherin EGF LAG seven-pass G-type receptor 1	mRNA
chr9	123079550	123079587	8	+	41	RABGAP1	RAB GTPase activating protein 1	mRNA
chr14	51639142	51639177	8	-	41	FRMD6	FERM domain containing 6	mRNA
chr1	27670207	27670249	8	+	43	IFI6	interferon alpha inducible protein 6	mRNA
chr3	131001366	131001412	8	+	47	ATP2C1	ATPase secretory pathway Ca2+ transporting 1	mRNA
chr15	89591694	89591733	8	-	47	TICRR	TOPBP1 interacting checkpoint and replication regulator	mRNA
chr16	67007435	67007468	8	+	49	CES4A	carboxylesterase 4A	mRNA
chr22	25086411	25086460	8	+	51	KIAA1671	KIAA1671	mRNA
chr16	10448627	10448678	8	+	52	ATF7IP2	activating transcription factor 7 interacting protein 2	mRNA
chr21	15794857	15794909	8	+	54	USP25	ubiquitin specific peptidase 25	mRNA
chr2	200890885	200890950	8	+	66	NIF3L1	NGG1 interacting factor 3 like 1	mRNA
chr7	158640350	158640384	8	-	67	NCAPG2	non-SMC condensin II complex subunit G2	mRNA
chr6	16263090	16263149	8	+	76	GMPR	guanosine monophosphate reductase	mRNA
chr7	98304469	98304529	8	-	78	BAIAP2L1	BAR/IMD domain containing adaptor protein 2 like 1	mRNA
chr18	3600204	3600249	8	+	91	DLGAP1	DLG associated protein 1	mRNA
chrX	85969516	85969564	8	-	148	CHM	CHM Rab escort protein	mRNA
chr17	35989759	35989793	8	+	35	CCL15-CCL14		lncRNA
chr15	84199325	84199371	8	-	47	LOC440300		lncRNA
chr3	163231300	163231351	8	-	56	LINC01192		lncRNA
chr22	35167660	35167706	8	-	58	LINC01399	long intergenic non-protein coding RNA 1399	lncRNA
chr2	104506633	104506667	8	+	63	LINC01102	long intergenic non-protein coding RNA 1102	lncRNA
chr20	25677237	25677298	8	-	77	ZNF337-AS1		lncRNA
chr22	35168130	35168193	8	-	80	LINC01399		lncRNA
chr4	39593577	39593627	8	-	81	UGDH-AS1		lncRNA
chrX	155487001	155487038	8	-	43	TMLHE-AS1		lncRNA
chr15	84169504	84169556	8	+	56	DNM1P41		pseudo
chr10	87845395	87845440	8	+	47	CFL1P1		pseudo
chr7	76997565	76997611	8	+	87	DTX2P1		pseudo
chr4	143973216	143973251	8	+	36	LOC101927636		lncRNA
chr11	12059338	12059369	8	+	102	LINC02547		lncRNA
chr13	27256537	27256568	8	+	32	RPL21	ribosomal protein L21	mRNA
chr6	122443563	122443592	8	-	32	SERINC1	serine incorporator 1	mRNA
chr17	37611993	37612022	8	-	34	DDX52	DExD-box helicase 52	mRNA
chr2	60797730	60797762	8	+	34	PAPOLG	poly(A) polymerase gamma	mRNA
chr12	121185904	121185932	8	+	34	P2RX7	purinergic receptor P2X 7	mRNA
chr1	182383683	182383713	8	-	36	GLUL	glutamate-ammonia ligase	mRNA
chr1	248918944	248918975	8	+	36	PGBD2	piggyBac transposable element derived 2	mRNA
chr20	3805940	3805975	8	+	36	CDC25B	cell division cycle 25B	mRNA
chr20	49096726	49096762	8	+	37	CSE1L	chromosome segregation 1 like	mRNA
chr1	65231288	65231323	8	+	40	AK4	adenylate kinase 4	mRNA
chr9	136207332	136207371	8	-	40	QSOX2	quiescin sulfhydryl oxidase 2	mRNA
chr2	11181801	11181832	8	+	43	ROCK2	Rho associated coiled-coil containing protein kinase 2	mRNA
chr20	13820509	13820545	8	-	44	NDUFAF5	NADH:ubiquinone oxidoreductase complex assembly factor 5	mRNA
chr1	32042769	32042813	8	+	45	KHDRBS1	KH RNA binding domain containing, signal transduction associated 1	mRNA
chr17	1706573	1706617	8	-	45	TLCD2	TLC domain containing 2	mRNA
chr5	177065869	177065913	8	+	45	ZNF346	zinc finger protein 346	mRNA
chr6	106569242	106569284	8	+	45	CRYBG1	crystallin beta-gamma domain containing 1	mRNA
chr6	115939663	115939708	8	-	46	FRK	fyn-related Src family tyrosine kinase	mRNA
chr2	230819798	230819836	8	+	47	CAB39	calcium binding protein 39	mRNA
chr19	55230266	55230312	8	-	47	PPP6R1	protein phosphatase 6 regulatory subunit 1	mRNA
chr11	34100012	34100060	8	+	49	CAPRIN1	cell cycle associated protein 1	mRNA
chr15	52548119	52548157	8	-	50	ARPP19	cAMP regulated phosphoprotein 19	mRNA
chr1	246767365	246767410	8	+	51	SCCPDH	saccharopine dehydrogenase (putative)	mRNA
chr22	45843865	45843918	8	+	54	ATXN10	ataxin 10	mRNA
chr6	36602729	36602782	8	+	54	SRSF3	serine and arginine rich splicing factor 3	mRNA
chr3	101822293	101822335	8	+	55	NXPE3	neurexophilin and PC-esterase domain family member 3	mRNA

chr6	116495813	116495854	8	+	55	TRAPPC3L	trafficking protein particle complex 3 like	mRNA
chr2	168773287	168773342	8	+	56	CERS6	ceramide synthase 6	mRNA
chr12	113390839	113390888	8	-	57	PLBD2	phospholipase B domain containing 2	mRNA
chr6	115938997	115939033	8	-	58	FRK	fyn-related Src family tyrosine kinase	mRNA
chr3	23980017	23980074	8	+	60	NR1D2	nuclear receptor subfamily 1 group D member 2	mRNA
chr17	4898001	4898032	8	+	60	MINK1	misshapen like kinase 1	mRNA
chr19	56527272	56527305	8	-	61	ZNF471	zinc finger protein 471	mRNA
chr10	87753780	87753820	8	-	63	ATAD1	ATPase family AAA domain containing 1	mRNA
chr11	126202430	126202493	8	-	64	RPUSD4	RNA pseudouridine synthase D4	mRNA
chr1	24471665	24471728	8	+	64	NIPAL3	NIPA like domain containing 3	mRNA
chr5	43706851	43706910	8	-	64	NNT	nicotinamide nucleotide transhydrogenase	mRNA
chr2	131147030	131147093	8	+	65	PLEKHB2	pleckstrin homology domain containing B2	mRNA
chr3	123492064	123492135	8	-	72	HACD2	3-hydroxyacyl-CoA dehydratase 2	mRNA
chr11	77666058	77666097	8	-	72	RSF1	remodeling and spacing factor 1	mRNA
chr17	44034749	44034788	8	-	73	LSM12	LSM12 homolog	mRNA
chr5	160252037	160252094	8	-	73	CCNJL	cyclin J like	mRNA
chr7	103099558	103099596	8	+	75	ARMC10	armadillo repeat containing 10	mRNA
chr3	189895006	189895068	8	+	76	TP63	tumor protein p63	mRNA
chr7	152121967	152122033	8	+	76	GALNT11	polypeptide N-acetylgalactosaminyltransferase 11	mRNA
chr14	70325381	70325427	8	-	77	COX16	cytochrome c oxidase assembly factor COX16	mRNA
chr1	149943729	149943794	8	-	77	OTUD7B	OTU deubiquitinase 7B	mRNA
chr18	216361	216397	8	+	79	THOC1	THO complex 1	mRNA
chr3	122571919	122571972	8	+	80	DTX3L	deltex E3 ubiquitin ligase 3L	mRNA
chr15	97971300	97971368	8	+	81	ARRDC4	arrestin domain containing 4	mRNA
chr16	67229427	67229500	8	-	84	FHOD1	formin homology 2 domain containing 1	mRNA
chr1	89181876	89181913	8	-	89	GBP4	guanylate binding protein 4	mRNA
chr3	123491987	123492060	8	-	89	HACD2	3-hydroxyacyl-CoA dehydratase 2	mRNA
chr4	138164912	138165011	8	-	100	SLC7A11	solute carrier family 7 member 11	mRNA
chr22	19036392	19036417	8	-	100	DGCR2	DiGeorge syndrome critical region gene 2	mRNA
chrX	54444959	54445002	8	+	103	TSR2	TSR2, 20S rRNA accumulation, homolog (S. cerevisiae)	mRNA
chr19	42377300	42377353	8	+	107	MEGF8	multiple EGF like domains 8	mRNA
chr2	203427263	203427323	8	+	67	ABI2	abl interactor 2	mRNA
chr11	65624341	65624366	7	+	31	PCNX3	pecanex 3	mRNA
chr10	42592910	42592948	7	-	40	ZNF33B	zinc finger protein 33B	mRNA
chr4	77048275	77048319	7	-	45	CCNI	cyclin I	mRNA
chr14	31144212	31144257	7	-	46	HECTD1	HECT domain E3 ubiquitin protein ligase 1	mRNA
chr4	39113019	39113059	7	+	55	KLHL5	kelch like family member 5	mRNA
chr7	112784576	112784620	7	-	62	TMEM168	transmembrane protein 168	mRNA
chr5	128112880	128112933	7	+	85	SLC12A2	solute carrier family 12 member 2	mRNA
chr16	16379064	16379118	7	+	62	NPIPA7	nuclear pore complex interacting protein family, member A7	mRNA
chr14	19300749	19300790	7	-	44	DUXAP10		pseudo
chr12	102216103	102216145	7	-	54			no annotation
chr15	39419507	39419544	7	+	46			no annotation
chr22	31053459	31053503	7	-	50			no annotation
chr12	102254889	102254944	7	-	56			no annotation
chr4	104036786	104036820	7	-	85			no annotation
chr8	17811798	17811831	7	+	43			no annotation
chr4	40166741	40166767	7	+	27			no annotation
chr12	16695045	16695088	7	+	45			no annotation
chr22	21954186	21954215	7	+	48			no annotation
chr12	102254561	102254608	7	+	50			no annotation
chr11	83422633	83422664	7	+	53			no annotation
chr15	93720219	93720268	7	-	58			no annotation
chr2	210022925	210022994	7	+	75			no annotation
chr22	15304326	15304390	7	-	97			no annotation
chr6	3303281	3303312	7	-	32	SLC22A23	solute carrier family 22 member 23	mRNA
chr2	219555449	219555482	7	+	34	OBSL1	obscurin like cytoskeletal adaptor 1	mRNA
chr3	50101291	50101324	7	+	36	RBM5	RNA binding motif protein 5	mRNA
chr12	4676051	4676086	7	-	36	NDUFA9	NADH:ubiquinone oxidoreductase subunit A9	mRNA
chr15	45598094	45598129	7	-	36	BLOC1S6	biogenesis of lysosomal organelles complex 1 subunit 6	mRNA
chr2	61887742	61887777	7	-	37	CCT4	chaperonin containing TCP1 subunit 4	mRNA
chr16	67615430	67615460	7	-	38	CTCF	CCCTC-binding factor	mRNA
chr21	15063795	15063830	7	-	38	NRIP1	nuclear receptor interacting protein 1	mRNA
chr15	72370508	72370541	7	-	43	HEXA	hexosaminidase subunit alpha	mRNA

chr13	39044284	39044329	7	+	46	NHLRC3	NHL repeat containing 3	mRNA
chrX	41649589	41649624	7	+	46	CASK	calcium/calmodulin dependent serine protein kinase	mRNA
chr8	43063251	43063294	7	+	47	FNTA	farnesyltransferase	mRNA
chr9	124528327	124528362	7	-	47	NR6A1	nuclear receptor subfamily 6 group A member 1	mRNA
chr4	37952410	37952451	7	-	47	TBC1D1	TBC1 domain family member 1	mRNA
chr8	11319047	11319080	7	-	50	MTMR9	myotubularin related protein 9	mRNA
chr22	16978267	16978296	7	-	50	GAB4	GRB2 associated binding protein family member 4	mRNA
chr17	81834199	81834240	7	+	51	PPP1R27	protein phosphatase 1, regulatory subunit 27	mRNA
chr10	96134614	96134670	7	+	57	ZNF518A	zinc finger protein 518A	mRNA
chr14	104704663	104704698	7	-	58	INF2	inverted formin, FH2 and WH2 domain containing	mRNA
chr6	36503925	36503974	7	-	66	STK38	serine/threonine kinase 38	mRNA
chr1	120432693	120432759	7	+	67	NBPF8	NBPF member 8	mRNA
chr1	186412028	186412093	7	+	70	ODR4	odr-4 GPCR localization factor homolog	mRNA
chr17	5431929	5431997	7	-	76	RPAIN	RPA interacting protein	mRNA
chr12	50190182	50190232	7	+	80	LIMA1	LIM domain and actin binding 1	mRNA
chr1	65630324	65630354	7	-	83	LEPR	leptin receptor	mRNA
chr14	19372541	19372573	7	+	33	LINC01297		lncRNA
chr3	18128406	18128442	7	+	38	LOC339862		lncRNA
chr3	54639500	54639541	7	+	42	ESRG		lncRNA
chr20	50318430	50318478	7	-	54	LINC01271	long intergenic non-protein coding RNA 1271	lncRNA
chr14	21397291	21397323	7	-	51	SNORD8		snoRNA
chr17	18538299	18538337	7	+	60	CCDC144B		pseudo
chr20	26209239	26209289	7	-	51	MIR663AHG		lncRNA
chr8	143745916	143745961	7	+	46	FAM83H-AS1		lncRNA
chr20	12951902	12951945	7	+	56	LINC01722		lncRNA
chr11	2635629	2635670	7	+	66	KCNQ1OT1		lncRNA
chr7	148989284	148989331	7	-	78	GHET1		lncRNA
chr13	29512648	29512679	7	-	32	SLC7A1	solute carrier family 7 member 1	mRNA
chr7	121350032	121350065	7	-	34	FAM3C	family with sequence similarity 3 member C	mRNA
chr5	14696868	14696902	7	+	35	OTULIN	OTU deubiquitinase with linear linkage specificity	mRNA
chr6	100858643	100858673	7	-	37	ASCC3	activating signal cointegrator 1 complex subunit 3	mRNA
chr16	75474445	75474479	7	+	38	CHST6	carbohydrate sulfotransferase 6	mRNA
chr19	8509850	8509887	7	+	38	ZNF414	zinc finger protein 414	mRNA
chr20	37983142	37983179	7	+	38	TTI1	TELO2 interacting protein 1	mRNA
chr6	89163800	89163837	7	+	39	PM20D2	peptidase M20 domain containing 2	mRNA
chr3	100180318	100180353	7	-	39	CMSS1	cms1 ribosomal small subunit homolog	mRNA
chr5	137752856	137752893	7	-	40	HNRNPA0	heterogeneous nuclear ribonucleoprotein A0	mRNA
chr6	142444751	142444790	7	+	40	ADGRG6	adhesion G protein-coupled receptor G6	mRNA
chr10	119167785	119167821	7	-	41	PRDX3	peroxiredoxin 3	mRNA
chr18	23531789	23531823	7	-	42	RMC1	regulator of MON1-CCZ1	mRNA
chr7	40127229	40127264	7	-	42	MPLKIP	M-phase specific PLK1 interacting protein	mRNA
chr7	93101045	93101079	7	-	42	SAMD9	sterile alpha motif domain containing 9	mRNA
chr6	34279776	34279818	7	-	43	NUDT3	nudix (nucleoside diphosphate linked moiety X)-type motif 3	mRNA
chr6	89162542	89162584	7	+	43	PM20D2	peptidase M20 domain containing 2	mRNA
chr9	19372740	19372783	7	-	44	DENND4C	DENN domain containing 4C	mRNA
chr1	15568691	15568733	7	+	45	DNAJC16	DnaJ heat shock protein family (Hsp40) member C16	mRNA
chr8	22005656	22005701	7	+	46	XPO7	exportin 7	mRNA
chr17	4143341	4143377	7	-	46	CYB5D2	cytochrome b5 domain containing 2	mRNA
chr3	100364704	100364751	7	-	48	TOMM70A	translocase of outer mitochondrial membrane 70 homolog A (S. cerevisiae)	mRNA
chr3	188879816	188879858	7	+	48	LPP	LIM domain containing preferred translocation partner in lipoma	mRNA
chr7	116797014	116797063	7	+	50	MET	MET proto-oncogene, receptor tyrosine kinase	mRNA
chr12	112508815	112508866	7	+	52	PTPN11	protein tyrosine phosphatase non-receptor type 11	mRNA
chr14	74736482	74736516	7	+	53	FCF1	FCF1 rRNA-processing protein	mRNA
chr4	105969829	105969882	7	+	54	NPNT	nephronectin	mRNA
chr1	32196831	32196871	7	+	55	TXLNA	taxilin alpha	mRNA
chr14	105470559	105470612	7	+	55	MTA1	metastasis associated 1	mRNA
chr11	63758621	63758673	7	+	55	RTN3	reticulon 3	mRNA
chr6	158634269	158634310	7	+	57	TMEM181	transmembrane protein 181	mRNA
chr17	59110374	59110431	7	-	58	SKA2	spindle and kinetochore associated complex subunit 2	mRNA
chr20	49096669	49096724	7	+	58	CSE1L	chromosome segregation 1 like	mRNA
chr1	11919679	11919734	7	-	59	KIAA2013	KIAA2013	mRNA
chr1	112671452	112671511	7	+	60	CAPZA1	capping actin protein of muscle Z-line subunit alpha 1	mRNA
chr17	38323042	38323086	7	+	61	MRPL45	mitochondrial ribosomal protein L45	mRNA
chr12	92144041	92144098	7	-	61	BTG1	B-cell translocation gene 1, anti-proliferative	mRNA

chr9	131577725	131577756	7	-	62	RAPGEF1	Rap guanine nucleotide exchange factor 1	mRNA
chr15	24683006	24683048	7	-	63	NPAP1	nuclear pore associated protein 1	mRNA
chr12	53188391	53188430	7	+	64	ZNF740	zinc finger protein 740	mRNA
chr5	42721080	42721125	7	+	65	GHR	growth hormone receptor	mRNA
chr5	102236582	102236647	7	+	66	SLCO4C1	solute carrier organic anion transporter family, member 4C1	mRNA
chr11	63624888	63624947	7	-	67	ATL3	atlastin GTPase 3	mRNA
chr15	65887994	65888026	7	+	67	RAB11A	RAB11A, member RAS oncogene family	mRNA
chr1	23795484	23795513	7	+	68	LYPLA2	lysophospholipase 2	mRNA
chr6	57008625	57008662	7	-	68	BEND6	BEN domain containing 6	mRNA
chr5	97093596	97093627	7	-	70	LIX1	limb and CNS expressed 1	mRNA
chr2	182919665	182919715	7	+	73	NCKAP1	NCK associated protein 1	mRNA
chr12	93399922	93399969	7	+	75	NUDT4	nudix hydrolase 4	mRNA
chr14	105479840	105479914	7	+	75	CRIP2	cysteine rich protein 2	mRNA
chr16	13239078	13239126	7	+	78	SHISA9	shisa family member 9	mRNA
chr14	75651177	75651218	7	-	80	ERG28	ergosterol biosynthesis 28 homolog	mRNA
chr12	6975857	6975938	7	+	86	EMG1	EMG1 N1-specific pseudouridine methyltransferase	mRNA
chr16	4383271	4383336	7	+	88	VASN	vasorin	mRNA
chr17	39671905	39671976	7	-	91	PGAP3	post-GPI attachment to proteins phospholipase 3	mRNA
chr1	92392077	92392153	7	-	97	RPAP2	RNA polymerase II associated protein 2	mRNA
chr19	10513307	10513360	7	-	105	S1PR5	sphingosine-1-phosphate receptor 5	mRNA
chr19	5691599	5691644	6	+	65	RPL36	ribosomal protein L36	mRNA
chr8	73292688	73292797	6	-	110	RPL7	ribosomal protein L7	mRNA
chr6	34425079	34425221	6	-	143	RPS10	ribosomal protein S10	mRNA
chr6	34426031	34426069	6	-	39	RPS10	ribosomal protein S10	mRNA
chr22	16614559	16614615	6	-	69	TPTEP1		pseudo
chr16	31196746	31196772	6	-	29			no annotation
chr4	25201072	25201110	6	-	40			no annotation
chr8	26423724	26423756	6	-	46			no annotation
chr21	44244752	44244795	6	-	48			no annotation
chr3	858038	858085	6	-	48			no annotation
chr17	41519354	41519394	6	+	49			no annotation
chr12	102255959	102255983	6	-	57			no annotation
chr11	86436637	86436664	6	+	28			no annotation
chr8	36279422	36279451	6	+	38			no annotation
chr2	38083263	38083302	6	+	40			no annotation
chr4	121871988	121872029	6	+	42			no annotation
chr12	102255245	102255290	6	+	47			no annotation
chr17	45558630	45558671	6	-	61			no annotation
chr22	15302844	15302891	6	-	75			no annotation
chr19	48704195	48704208	6	+	69	FUT2	fucosyltransferase 2 (secretor status included)	mRNA
chr14	50727603	50727628	6	+	31	NIN	ninein	mRNA
chr16	89740613	89740646	6	-	34	ZNF276	zinc finger protein 276	mRNA
chr18	79967425	79967458	6	+	36	HSBP1L1	heat shock factor binding protein 1-like 1	mRNA
chr22	17003836	17003870	6	-	36	GAB4	GRB2 associated binding protein family member 4	mRNA
chr9	122874692	122874722	6	+	37	RC3H2	ring finger and CCCH-type domains 2	mRNA
chr17	63746922	63746961	6	-	40	CCDC47	coiled-coil domain containing 47	mRNA
chr3	167692439	167692469	6	-	41	PDCD10	programmed cell death 10	mRNA
chr19	44095039	44095082	6	+	44	ZNF224	zinc finger protein 224	mRNA
chr12	20860261	20860303	6	+	45	SLCO1B3	solute carrier organic anion transporter family member 1B3	mRNA
chr2	207531487	207531522	6	+	46	CREB1	cAMP responsive element binding protein 1	mRNA
chr12	131712611	131712658	6	+	51	SFSWAP	splicing factor SWAP	mRNA
chr2	138568445	138568490	6	+	52	SPOPL	speckle type BTB/POZ protein like	mRNA
chr9	87918949	87918993	6	+	52	SPATA31C1	SPATA31 subfamily C member 1	mRNA
chr2	229180778	229180825	6	-	54	PID1	phosphotyrosine interaction domain containing 1	mRNA
chr6	111311973	111312007	6	+	55	REV3L	REV3 like, DNA directed polymerase zeta catalytic subunit	mRNA
chr8	11782698	11782739	6	+	67	NEIL2	nei like DNA glycosylase 2	mRNA
chr2	3515579	3515629	6	+	67	ADI1	acireductone dioxygenase 1	mRNA
chr5	45575681	45575744	6	-	68	HCN1	hyperpolarization activated cyclic nucleotide gated potassium channel 1	mRNA
chr2	210026515	210026563	6	+	69	KANSL1L	KAT8 regulatory NSL complex subunit 1 like	mRNA
chr14	64523597	64523673	6	+	77	ZBTB1	zinc finger and BTB domain containing 1	mRNA
chr8	79956616	79956675	6	+	79	MRPS28	mitochondrial ribosomal protein S28	mRNA
chr2	111237180	111237211	6	+	35	MIR4435-2HG		lncRNA
chr10	43940445	43940479	6	+	39	LINC00841		lncRNA
chrX	3821717	3821763	6	+	47	LOC389906		lncRNA

chr2	14230625	14230661	6	+	37	LINC00276		lncRNA
chr17	12636408	12636441	6	+	44	LINC00670		lncRNA
chr3	54638648	54638693	6	-	46	ESRG		lncRNA
chr4	14473558	14473593	6	+	49	LINC00504		lncRNA
chr2	25039155	25039202	6	-	50	DNAJC27-AS1		lncRNA
chr8	65527040	65527089	6	+	50	LINC01299		lncRNA
chr5	23980449	23980481	6	-	57	C5orf17		lncRNA
chr2	66391967	66392005	6	-	61	LINC01873		lncRNA
chr2	66392061	66392149	6	+	127	LINC01873		lncRNA
chr3	98981334	98981356	6	+	24	LINC00973		lncRNA
chr12	49293965	49294006	6	+	42	LOC101927267		lncRNA
chr3	179398456	179398465	6	+	10	GNB4	G protein subunit beta 4	mRNA
chr3	179397901	179397922	6	+	22	GNB4	G protein subunit beta 4	mRNA
chr4	40157068	40157092	6	-	26	N4BP2	NEDD4 binding protein 2	mRNA
chr21	37612709	37612736	6	-	28	KCNJ6	potassium channel, inwardly rectifying subfamily J, member 6	mRNA
chrX	55181518	55181547	6	-	30	MTRNR2L10	MT-RNR2-like 10	mRNA
chr13	51763565	51763596	6	+	34	WDFY2	WD repeat and FYVE domain containing 2	mRNA
chr19	6663293	6663321	6	+	34	TNFSF14	TNF superfamily member 14	mRNA
chr11	74978533	74978567	6	-	35	SPCS2	signal peptidase complex subunit 2	mRNA
chr11	74978583	74978617	6	-	36	SPCS2	signal peptidase complex subunit 2	mRNA
chr19	5891423	5891455	6	+	37	NDUFA11	NADH:ubiquinone oxidoreductase subunit A11	mRNA
chr14	102498851	102498881	6	-	38	TECPR2	tectonin beta-propeller repeat containing 2	mRNA
chr1	92398298	92398332	6	-	40	RPAP2	RNA polymerase II associated protein 2	mRNA
chr5	116446245	116446283	6	+	41	SEMA6A	semaphorin 6A	mRNA
chr10	73498442	73498478	6	+	44	USP54	ubiquitin specific peptidase 54	mRNA
chr1	25861979	25862022	6	-	45	PAQR7	progestin and adipoQ receptor family member VII	mRNA
chr3	119669577	119669621	6	-	45	COX17	cytochrome c oxidase copper chaperone COX17	mRNA
chrX	18239825	18239865	6	-	45	SCML2	Scm polycomb group protein like 2	mRNA
chr11	74977220	74977267	6	+	48	SPCS2	signal peptidase complex subunit 2	mRNA
chr11	118354501	118354549	6	+	50	CD3G	CD3G molecule	mRNA
chr7	16783844	16783895	6	+	52	TSPAN13	tetraspanin 13	mRNA
chr7	66955634	66955677	6	+	52	TMEM248	transmembrane protein 248	mRNA
chr3	48443876	48443927	6	+	52	TMA7	translation machinery associated 7 homolog (S. cerevisiae)	mRNA
chr1	156525789	156525818	6	-	53	IQGAP3	IQ motif containing GTPase activating protein 3	mRNA
chr16	19268116	19268171	6	+	56	SYT17	synaptotagmin 17	mRNA
chrX	68042594	68042640	6	-	56	OPHN1	oligophrenin 1	mRNA
chr11	791193	791248	6	-	56	SLC25A22	solute carrier family 25 member 22	mRNA
chr6	122781910	122781946	6	+	56	FABP7	fatty acid binding protein 7	mRNA
chr3	9806475	9806516	6	+	59	ARPC4	actin related protein 2/3 complex subunit 4	mRNA
chr7	2442107	2442162	6	+	59	CHST12	carbohydrate (chondroitin 4) sulfotransferase 12	mRNA
chr12	32645444	32645489	6	-	59	FGD4	FYVE, RhoGEF and PH domain containing 4	mRNA
chr19	5152862	5152922	6	-	63	KDM4B	lysine demethylase 4B	mRNA
chr4	106043726	106043777	6	+	63	TBCK	TBC1 domain containing kinase	mRNA
chr1	113975978	113976015	6	+	64	HIPK1	homeodomain interacting protein kinase 1	mRNA
chr17	74593815	74593868	6	-	64	C17orf77	chromosome 17 open reading frame 77	mRNA
chr2	231400978	231401028	6	+	67	B3GNT7	UDP-GlcNAc:betaGal beta-1,3-N-acetylglucosaminyltransferase 7	mRNA
chr2	70296704	70296738	6	-	70	FAM136A	family with sequence similarity 136 member A	mRNA
chr3	100364805	100364874	6	-	70	TOMM70A	translocase of outer mitochondrial membrane 70 homolog A (S. cerevisiae)	mRNA
chr6	89643415	89643490	6	-	77	MDN1	midasin AAA ATPase 1	mRNA
chr13	36224964	36224980	6	-	50	CCDC169	coiled-coil domain containing 169	mRNA
chr20	326541	326564	5	-	25	SOX12	SRY-box transcription factor 12	mRNA
chr15	44533935	44533958	5	+	27			no annotation
chr10	89667554	89667580	5	+	28			no annotation
chr22	36072459	36072491	5	-	33			no annotation
chr3	120136487	120136522	5	-	36			no annotation
chr5	21616500	21616534	5	-	36			no annotation
chr10	116583182	116583221	5	-	40			no annotation
chr16	35227634	35227675	5	-	42			no annotation
chr18	27336954	27336994	5	+	43			no annotation
chr15	46804716	46804755	5	-	48			no annotation
chr2	63642694	63642740	5	+	48			no annotation
chr6	36158467	36158517	5	+	52			no annotation
chr2	46778993	46779027	5	-	53			no annotation
chr6	94447121	94447147	5	-	27			no annotation

chr1	92189833	92189852	5	+	32			no annotation
chr12	132913451	132913486	5	+	36			no annotation
chr16	24472951	24472987	5	-	38			no annotation
chr5	100053503	100053539	5	-	53			no annotation
chr20	3243347	3243382	5	+	61			no annotation
chr5	112164624	112164647	5	+	27	EPB41L4A	erythrocyte membrane protein band 4.1 like 4A	mRNA
chr20	53398567	53398594	5	-	28	TSHZ2	teashirt zinc finger homeobox 2	mRNA
chr11	88322110	88322139	5	+	33	CTSC	cathepsin C	mRNA
chr12	122020317	122020339	5	-	34	BCL7A	B-cell CLL/lymphoma 7 protein family member A	mRNA
chr11	117907881	117907907	5	+	35	TMPRSS13	transmembrane serine protease 13	mRNA
chr17	75263693	75263722	5	-	35	MRPS7	mitochondrial ribosomal protein S7	mRNA
chr12	65954995	65955027	5	-	36	HMGA2	high mobility group AT-hook 2	mRNA
chr7	102352533	102352569	5	+	39	SPDYE6	speedy/RINGO cell cycle regulator family member E6	mRNA
chr11	13394774	13394813	5	+	41	BTBD10	BTB domain containing 10	mRNA
chr18	37075575	37075611	5	-	42	KIAA1328	KIAA1328	mRNA
chr9	33347912	33347951	5	-	48	NFX1	nuclear transcription factor, X-box binding 1	mRNA
chr11	31574770	31574792	5	-	50	ELP4	elongator acetyltransferase complex subunit 4	mRNA
chr2	30606984	30607016	5	-	51	LCLAT1	lysocardiolipin acyltransferase 1	mRNA
chr19	9813350	9813399	5	+	52	FBXL12	F-box and leucine rich repeat protein 12	mRNA
chr16	3051190	3051236	5	+	53	MMP25	matrix metallopeptidase 25	mRNA
chr8	30176792	30176824	5	-	54	DCTN6	dynactin subunit 6	mRNA
chr14	105370527	105370557	5	-	61	PACS2	phosphofurin acidic cluster sorting protein 2	mRNA
chr15	45598164	45598221	5	-	65	BLOC1S6	biogenesis of lysosomal organelles complex 1 subunit 6	mRNA
chr18	80012968	80013039	5	+	72	TXNL4A	thioredoxin like 4A	mRNA
chr1	64106265	64106302	5	+	58	ROR1-AS1		lncRNA
chr1	211433837	211433894	5	-	58	LINC00467		lncRNA
chr3	49551111	49551154	5	+	63	BSN-AS2	BSN antisense RNA 2 (head to head)	lncRNA
chr17	18538293	18538337	5	-	69	CCDC144B		pseudo
chrX	73803732	73803772	5	+	42	TSIX		lncRNA
chr11	67603487	67603521	5	-	43	C11orf72		lncRNA
chr7	23102490	23102532	5	-	44	KLHL7-DT		lncRNA
chr17	76805633	76805676	5	+	45	LINC02080		lncRNA
chrX	73804503	73804542	5	+	45	TSIX		lncRNA
chr17	12636774	12636810	5	-	83	LINC00670		lncRNA
chr7	116276599	116276634	5	+	41	LOC102724434		lncRNA
chr19	48705316	48705336	5	+	21	FUT2	fucosyltransferase 2 (secretor status included)	mRNA
chr1	114705771	114705791	5	+	21	NRAS	neuroblastoma RAS viral (v-ras) oncogene homolog	mRNA
chr9	6506525	6506550	5	-	26	UHRF2	ubiquitin like with PHD and ring finger domains 2	mRNA
chr5	1222584	1222611	5	+	28	SLC6A19	solute carrier family 6 member 19	mRNA
chr11	8687216	8687244	5	-	32	RPL27A	ribosomal protein L27a	mRNA
chr8	55524598	55524629	5	-	32	XKR4	XK, Kell blood group complex subunit-related family, member 4	mRNA
chr9	34368301	34368330	5	+	32	MYORG	myogenesis regulating glycosidase	mRNA
chr18	32070211	32070238	5	+	34	RNF125	ring finger protein 125	mRNA
chr11	2131835	2131859	5	+	34	IGF2	insulin like growth factor 2	mRNA
chr12	49273904	49273938	5	-	35	TUBA1C	tubulin alpha 1c	mRNA
chr14	90526103	90526134	5	+	35	TTC7B	tetratricopeptide repeat domain 7B	mRNA
chr1	160216297	160216332	5	+	36	DCAF8	DDB1 and CUL4 associated factor 8	mRNA
chr1	226226432	226226463	5	+	37	MIXL1	Mix paired-like homeobox	mRNA
chr4	69723810	69723846	5	-	37	SULT1B1	sulfotransferase family 1B member 1	mRNA
chrX	107064741	107064770	5	+	37	RBM41	RNA binding motif protein 41	mRNA
chr5	144478901	144478938	5	+	38	KCTD16	potassium channel tetramerization domain containing 16	mRNA
chr19	48600823	48600853	5	+	39	FAM83E	family with sequence similarity 83, member E	mRNA
chr3	49415495	49415520	5	-	41	TCTA	T-cell leukemia translocation altered	mRNA
chr7	152647040	152647080	5	+	42	XRCC2	X-ray repair complementing defective repair in Chinese hamster cells 2	mRNA
chr21	38301939	38301970	5	-	43	KCNJ15	potassium inwardly rectifying channel subfamily J member 15	mRNA
chr5	77431335	77431374	5	+	43	WDR41	WD repeat domain 41	mRNA
chr7	152647457	152647499	5	+	43	XRCC2	X-ray repair complementing defective repair in Chinese hamster cells 2	mRNA
chr7	13892750	13892794	5	+	45	ETV1	ETS variant transcription factor 1	mRNA
chr19	52293131	52293169	5	+	46	ZNF766	zinc finger protein 766	mRNA
chr3	101225399	101225435	5	-	47	IMPG2	interphotoreceptor matrix proteoglycan 2	mRNA
chrX	16763105	16763151	5	-	47	SYAP1	synapse associated protein 1	mRNA
chr12	113390317	113390364	5	-	48	PLBD2	phospholipase B domain containing 2	mRNA
chr16	4383339	4383388	5	+	50	VASN	vasorin	mRNA
chr9	21330118	21330162	5	+	50	KLHL9	kelch-like family member 9	mRNA

chr13	30205606	30205661	5	+	56	KATNAL1	katanin catalytic subunit A1 like 1	mRNA
chr17	28327136	28327188	5	+	58	TMEM97	transmembrane protein 97	mRNA
chr7	2437712	2437767	5	-	64	CHST12	carbohydrate (chondroitin 4) sulfotransferase 12	mRNA
chr9	88575483	88575547	5	-	65	NXNL2	nucleoredoxin like 2	mRNA
chr5	161289773	161289836	5	+	66	GABRB2	gamma-aminobutyric acid type A receptor beta2 subunit	mRNA
chr1	92389543	92389602	5	-	68	RPAP2	RNA polymerase II associated protein 2	mRNA
chrX	70063837	70063867	5	-	72	OTUD6A	OTU deubiquitinase 6A	mRNA
chr4	69724204	69724248	5	-	78	SULT1B1	sulfotransferase family 1B member 1	mRNA
chr1	53084875	53084907	5	-	33	PODN	podocan	mRNA
chr1	161037684	161037733	5	-	73	TSTD1	thiosulfate sulfurtransferase like domain containing 1	mRNA
chr5	96729712	96729725	4	-	15	CAST	calpastatin	mRNA
chr5	96730779	96730790	4	-	15	CAST	calpastatin	mRNA
chr19	50637895	50637920	4	-	26	SYT3	synaptotagmin 3	mRNA
chr8	36279419	36279444	4	-	34	MTND6P19		pseudo
chr2	2838279	2838301	4	+	23			no annotation
chr1	44150659	44150686	4	+	29			no annotation
chr2	63642547	63642578	4	+	32			no annotation
chr2	190537934	190537965	4	-	35			no annotation
chr1	630717	630754	4	+	101			no annotation
chr5	99997839	99997859	4	+	21			no annotation
chr22	37892360	37892385	4	+	30			no annotation
chr3	147941746	147941773	4	+	30			no annotation
chr7	130791545	130791577	4	+	33			no annotation
chr12	12649500	12649537	4	+	38			no annotation
chr16	21863789	21863831	4	-	43			no annotation
chr17	47049059	47049107	4	+	50			no annotation
chr22	36080492	36080535	4	+	72			no annotation
chr4	67731600	67731645	4	-	75			no annotation
chr16	4839267	4839292	4	+	27	GLYR1	glyoxylate reductase 1 homolog	mRNA
chr19	6717633	6717659	4	+	30	C3	complement C3	mRNA
chr19	6717661	6717690	4	+	30	C3	complement C3	mRNA
chr1	18858431	18858462	4	-	32	TAS1R2	taste receptor, type 1, member 2	mRNA
chr17	14328815	14328848	4	-	34	HS3ST3B1	heparan sulfate-glucosamine 3-sulfotransferase 3B1	mRNA
chr5	61114482	61114513	4	-	36	NDUFAF2	NADH:ubiquinone oxidoreductase complex assembly factor 2	mRNA
chr12	26978783	26978815	4	+	39	TM7SF3	transmembrane 7 superfamily member 3	mRNA
chr7	100077596	100077619	4	+	39	ZNF3	zinc finger protein 3	mRNA
chr9	33348453	33348492	4	-	40	NFX1	nuclear transcription factor, X-box binding 1	mRNA
chr9	83054708	83054736	4	+	42	RASEF	RAS and EF-hand domain containing	mRNA
chr17	51250075	51250104	4	+	44	MBTD1	mbt domain containing 1	mRNA
chr7	99485420	99485441	4	-	44	ZNF789	zinc finger protein 789	mRNA
chr3	142472210	142472250	4	+	49	ATR	ATR serine/threonine kinase	mRNA
chr17	4110880	4110915	4	+	52	ZZEF1	zinc finger ZZ-type and EF-hand domain containing 1	mRNA
chr19	58000396	58000427	4	+	52	ZNF606	zinc finger protein 606	mRNA
chr8	140902739	140902792	4	+	78	PTK2	protein tyrosine kinase 2	mRNA
chr15	45598265	45598363	4	-	102	BLOC1S6	biogenesis of lysosomal organelles complex 1 subunit 6	mRNA
chr2	32563882	32563917	4	-	36	BIRC6-AS2		lncRNA
chr5	142394456	142394494	4	-	46	SPRY4-AS1		lncRNA
chr2	165975488	165975532	4	-	52	LOC102724058		lncRNA
chr8	70646358	70646413	4	+	56	LACTB2-AS1		lncRNA
chr6	22135001	22135030	4	-	31	NBAT1		lncRNA
chr2	213276950	213276978	4	-	32	SPAG16-DT		lncRNA
chr2	37826262	37826288	4	+	32	LINC00211		lncRNA
chr3	128501793	128501819	4	-	33	GATA2-AS1		lncRNA
chr20	12951930	12951963	4	-	37	LINC01772		lncRNA
chr7	56534423	56534462	4	-	40	LOC101928401		lncRNA
chr11	127225	127268	4	-	44	LINC01001		lncRNA
chr10	17642941	17642986	4	+	58	STAM-AS1		lncRNA
chr16	29455126	29455181	4	+	60	SLX1B-SULT1A4		lncRNA
chr4	113761273	113761292	4	+	21	CAMK2D	calcium/calmodulin dependent protein kinase II delta	mRNA
chr2	174337814	174337833	4	-	22	SP9	Sp9 transcription factor	mRNA
chr21	45515629	45515652	4	+	24	SLC19A1	solute carrier family 19 member 1	mRNA
chr6	55755246	55755266	4	+	24	BMP5	bone morphogenetic protein 5	mRNA
chr2	96330068	96330092	4	-	25	ITPRIPL1	ITPRIP like 1	mRNA
chr16	28536527	28536553	4	+	27	NUPR1	nuclear protein, transcriptional regulator, 1	mRNA

chr4	127723789	127723814	4	-	29	INTU	inturned planar cell polarity protein	mRNA
chr6	131894448	131894478	4	+	31	ENPP1	ectonucleotide pyrophosphatase/phosphodiesterase 1	mRNA
chr1	166913934	166913966	4	-	34	ILDR2	immunoglobulin like domain containing receptor 2	mRNA
chr12	121651133	121651164	4	+	34	MORN3	MORN repeat containing 3	mRNA
chr17	82489810	82489833	4	-	35	NARF	nuclear prelamin A recognition factor	mRNA
chr12	121020302	121020334	4	+	36	OASL	2'-5'-oligoadenylate synthetase like	mRNA
chr5	176379334	176379370	4	+	37	ARL10	ADP-ribosylation factor-like 10	mRNA
chr2	70279584	70279616	4	-	37	PCYOX1	prenylcysteine oxidase 1	mRNA
chr12	54546173	54546197	4	-	41	NCKAP1L	NCK associated protein 1 like	mRNA
chr3	12734086	12734127	4	+	42	TMEM40	transmembrane protein 40	mRNA
chr5	65669615	65669655	4	-	43	SGTB	small glutamine rich tetratricopeptide repeat containing beta	mRNA
chr12	10159287	10159326	4	+	43	OLR1	oxidized low density lipoprotein receptor 1	mRNA
chr17	28908490	28908528	4	+	43	PHF12	PHD finger protein 12	mRNA
chr11	82729962	82729999	4	+	44	FAM181B	family with sequence similarity 181, member B	mRNA
chr19	34507186	34507229	4	-	45	WTIP	WT1 interacting protein	mRNA
chr9	83938920	83938963	4	+	47	C9orf64	chromosome 9 open reading frame 64	mRNA
chr10	38122607	38122638	4	-	49	ZNF37A	zinc finger protein 37A	mRNA
chr12	120460970	120461008	4	-	53	GATC	glutamyl-tRNA amidotransferase subunit C	mRNA
chr9	126697884	126697918	4	+	55	LMX1B	LIM homeobox transcription factor 1 beta	mRNA
chrX	46604601	46604632	4	+	56	SLC9A7	solute carrier family 9 member A7	mRNA
chr8	38988354	38988410	4	-	57	HTRA4	HtrA serine peptidase 4	mRNA
chr1	54638981	54639034	4	-	58	ACOT11	acyl-CoA thioesterase 11	mRNA
chr5	179035019	179035045	4	-	59	ZNF879	zinc finger protein 879	mRNA
chr3	182942706	182942727	4	+	59	DCUN1D1	defective in cullin neddylation 1 domain containing 1	mRNA
chr12	66121421	66121481	4	+	61	LLPH	LLP homolog, long-term synaptic facilitation factor	mRNA
chr19	1778785	1778815	4	-	61	ONECUT3	one cut homeobox 3	mRNA
chr5	6671000	6671060	4	-	61	SRD5A1	steroid 5 alpha-reductase 1	mRNA
chr10	122438857	122438903	4	-	66	PLEKHA1	pleckstrin homology domain containing A1	mRNA
chr2	173965297	173965331	4	+	89	SP3	Sp3 transcription factor	mRNA
chr19	44384808	44384834	4	-	27	ZNF285	zinc finger protein 285	mRNA
chr17	79081897	79081907	3	-	11	ENGASE	endo-beta-N-acetylglucosaminidase	mRNA
chr12	131986513	131986525	3	-	13	EP400	E1A binding protein p400	mRNA
chr17	79081061	79081073	3	-	13	ENGASE	endo-beta-N-acetylglucosaminidase	mRNA
chr2	219162104	219162118	3	-	15	SLC23A3	solute carrier family 23 member 3	mRNA
chr4	1819384	1819402	3	+	20	LETM1	leucine zipper and EF-hand containing transmembrane protein 1	mRNA
chr2	241869787	241869808	3	-	22	RTP5	receptor (chemosensory) transporter protein 5 (putative)	mRNA
chr7	128846334	128846355	3	-	22	FLNC	filamin C	mRNA
chr19	15372230	15372250	3	+	22	AKAP8	A-kinase anchoring protein 8	mRNA
chr9	127523756	127523778	3	+	23	NIBAN2	niban apoptosis regulator 2	mRNA
chr6	57913270	57913309	3	+	42	GUSBP4		pseudo
chr7	1464748	1464775	3	+	28			no annotation
chr11	115646681	115646705	3	-	32			no annotation
chrX	42448082	42448121	3	-	59			no annotation
chr20	52051694	52051712	3	+	19			no annotation
chr9	79036988	79037007	3	+	20			no annotation
chr1	629931	629956	3	+	29			no annotation
chr20	62633848	62633869	3	-	29			no annotation
chr4	141405802	141405828	3	+	31			no annotation
chr9	66721325	66721353	3	+	32			no annotation
chr7	55579014	55579050	3	+	37			no annotation
chr5	127550617	127550634	3	-	18	PRRC1	proline rich coiled-coil 1	mRNA
chr12	63623807	63623824	3	+	18	DPY19L2	dpy-19 like 2	mRNA
chr17	44720241	44720260	3	-	20	DBF4B	DBF4 zinc finger B	mRNA
chr15	68081526	68081549	3	-	24	PIAS1	protein inhibitor of activated STAT 1	mRNA
chr3	127966070	127966093	3	+	25	KBTBD12	kelch repeat and BTB domain containing 12	mRNA
chr1	108573525	108573549	3	-	25	FAM102B	family with sequence similarity 102 member B	mRNA
chr4	76480679	76480704	3	-	26	SHROOM3	shroom family member 3	mRNA
chr13	33849056	33849082	3	-	27	RFC3	replication factor C subunit 3	mRNA
chr19	15383382	15383408	3	+	28	AKAP8L	A-kinase anchoring protein 8 like	mRNA
chr10	72917431	72917459	3	-	30	OIT3	oncoprotein induced transcript 3	mRNA
chr17	56984915	56984941	3	-	32	SCPEP1	serine carboxypeptidase 1	mRNA
chr19	35353298	35353323	3	-	33	FFAR1	free fatty acid receptor 1	mRNA
chr12	95934858	95934883	3	+	36	CCDC38	coiled-coil domain containing 38	mRNA
chr19	50258509	50258545	3	-	39	MYH14	myosin heavy chain 14	mRNA

chr3	142472075	142472113	3	+	39	ATR	ATR serine/threonine kinase	mRNA
chr19	19932087	19932124	3	-	44	ZNF93	zinc finger protein 93	mRNA
chr19	43496028	43496063	3	+	46	PHLDB3	pleckstrin homology like domain family B member 3	mRNA
chr9	127184127	127184175	3	-	49	RALGPS1	Ral GEF with PH domain and SH3 binding motif 1	mRNA
chr1	174799960	174799992	3	-	50	RABGAP1L	RAB GTPase activating protein 1 like	mRNA
chr7	151501981	151502018	3	+	53	RHEB	Ras homolog, mTORC1 binding	mRNA
chr12	51202336	51202375	3	+	53	POU6F1	POU class 6 homeobox 1	mRNA
chr16	50074800	50074858	3	-	64	HEATR3	HEAT repeat containing 3	mRNA
chr21	37059189	37059232	3	+	44	PIGP	phosphatidylinositol glycan anchor biosynthesis class P	mRNA
chr11	19517870	19517892	3	-	24	NAV2-AS4		lncRNA
chr5	173710988	173711011	3	+	24	LINC01484		lncRNA
chrX	102826121	102826147	3	-	27	LINC00630		lncRNA
chrX	74237518	74237549	3	+	34	FTX		lncRNA
chr5	44775919	44775962	3	+	44	MRPS30-DT		lncRNA
chr12	12356761	12356779	3	+	20	LOH12CR2		lncRNA
chr1	77881719	77881747	3	-	46	NEXN-AS1		lncRNA
chr9	132594181	132594198	3	+	18	DDX31	DEAD-box helicase 31	mRNA
chr1	205303126	205303147	3	+	22	NUAK2	NUAK family kinase 2	mRNA
chr11	61581070	61581090	3	+	22	SYT7	synaptotagmin 7	mRNA
chr19	863413	863435	3	-	23	CFD	complement factor D	mRNA
chr1	42420316	42420339	3	+	24	RIMKLA	ribosomal modification protein rimK like family member A	mRNA
chr13	48710751	48710773	3	+	24	CYSLTR2	cysteinyl leukotriene receptor 2	mRNA
chr1	65638167	65638191	3	-	25	LEPR	leptin receptor	mRNA
chr10	118304470	118304491	3	+	25	FAM204A	family with sequence similarity 204 member A	mRNA
chr3	196049311	196049335	3	+	25	TFRC	transferrin receptor	mRNA
chr4	39477348	39477373	3	-	26	LIAS	lipoic acid synthetase	mRNA
chr10	112548435	112548461	3	-	27	VTI1A	vesicle transport through interaction with t-SNAREs 1A	mRNA
chr13	25163577	25163597	3	-	28	AMER2	APC membrane recruitment protein 2	mRNA
chrX	3608054	3608081	3	+	28	PRKX	protein kinase, X-linked	mRNA
chr16	4804949	4804977	3	+	29	GLYR1	glyoxylate reductase 1 homolog	mRNA
chr13	49492965	49492994	3	-	30	SETDB2	SET domain bifurcated histone lysine methyltransferase 2	mRNA
chr3	124905602	124905631	3	+	30	MUC13	mucin 13, cell surface associated	mRNA
chr9	33253853	33253881	3	+	30	BAG1	BCL2 associated athanogene 1	mRNA
chr12	55995423	55995452	3	-	32	RAB5B	RAB5B, member RAS oncogene family	mRNA
chr7	38724748	38724779	3	+	32	VPS41	VPS41 subunit of HOPS complex	mRNA
chr3	191267693	191267726	3	-	34	UTS2B	Urotensin 2B	mRNA
chr1	160029538	160029571	3	+	34	PIGM	phosphatidylinositol glycan anchor biosynthesis, class M	mRNA
chr13	27256536	27256568	3	-	35	RPL21	ribosomal protein L21	mRNA
chr7	40096640	40096659	3	-	37	CDK13	cyclin dependent kinase 13	mRNA
chr19	46467325	46467361	3	-	38	PNMA8A	PNMA family member 8A	mRNA
chr2	88110878	88110910	3	+	40	SMYD1	SET and MYND domain containing 1	mRNA
chr15	40662759	40662801	3	-	43	KNL1	kinetochore scaffold 1	mRNA
chr20	3926915	3926946	3	-	43	PANK2	pantothenate kinase 2	mRNA
chr1	220058686	220058727	3	+	45	BPNT1	3'(2'), 5'-bisphosphate nucleotidase 1	mRNA
chr2	27155709	27155745	3	-	46	TCF23	transcription factor 23	mRNA
chr18	74585809	74585840	3	-	47	CNDP1	carnosine dipeptidase 1	mRNA
chr1	114584776	114584808	3	+	48	DENND2C	DENN domain containing 2C	mRNA
chr14	70371424	70371476	3	+	53	SYNJ2BP	synaptojanin 2 binding protein	mRNA
chr9	109018914	109018961	3	+	53	TMEM245	transmembrane protein 245	mRNA
chr8	18029111	18029161	3	-	53	PCM1	pericentriolar material 1	mRNA
chr19	15057691	15057734	3	-	56	CASP14	caspase 14, apoptosis-related cysteine peptidase	mRNA
chr5	83054654	83054690	3	+	57	TMEM167A	transmembrane protein 167A	mRNA
chr15	94483494	94483525	3	-	64	MCTP2	multiple C2 and transmembrane domain containing 2	mRNA
chr1	26877881	26877905	3	+	25	GPN2	GPN-loop GTPase 2	mRNA
chr11	68894570	68894589	2	+	20	MRPL21	mitochondrial ribosomal protein L21	mRNA
chr1	53963299	53963319	2	-	22	LRRC42	leucine rich repeat containing 42	mRNA
chr11	94175863	94175889	2	-	28	PANX1	pannexin 1	mRNA
chr17	6854376	6854407	2	-	42	ALOX12P2		pseudo
chr1	9730463	9730483	2	+	21	CLSTN1	calsyntenin 1	mRNA
chrX	155260403	155260424	2	-	23	RAB39B	RAB39B, member RAS oncogene family	mRNA
chr15	20532500	20532526	2	-	27	GOLGA6L6	golgin A6 family-like 6	mRNA
chr13	110892942	110892959	2	+	18	ANKRD10	ankyrin repeat domain 10	mRNA

Supplementary Table S4_Specific Peaks bound to PURa in CLIP-seq_PURa vs IgG

Specific mRNA bound to PURα in CLIP-seq

Chr	Start	End	regionInfo	Gene	Description	Genetype
chr20	326541	326564	CDS	SOX12	SRY-box transcription factor 12	mRNA
chr11	62880946	62881234	CDS	SLC3A2	solute carrier family 3 member 2	mRNA
chr4	108621950	108621997	CDS	RPL34	ribosomal protein L34	mRNA
chr17	41518466	41518867	CDS	KRT15	keratin 15	mRNA
chr5	179705678	179705852	CDS	CANX	calnexin	mRNA
chr16	85801204	85801278	CDS	COX4I1	cytochrome c oxidase subunit 4I1	mRNA
chr3	194405993	194406094	CDS	ATP13A3	ATPase 13A3	mRNA
chr17	78971428	78971488	CDS	LGALS3BP	galectin 3 binding protein	mRNA
chr17	78971489	78971727	CDS	LGALS3BP	galectin 3 binding protein	mRNA
chr19	48966561	48966878	CDS	FTL	ferritin light chain	mRNA
chr6	113860605	113860679	CDS	MARCKS	myristoylated alanine-rich protein kinase C substrate	mRNA
chr11	319740	319870	CDS	IFITM3	interferon induced transmembrane protein 3	mRNA
chr8	11838558	11838778	CDS	FDFT1	farnesyl-diphosphate farnesyltransferase 1	mRNA
chr15	65887805	65887934	CDS	RAB11A	RAB11A, member RAS oncogene family	mRNA
chr1	203869869	203869983	CDS	SNRPE	small nuclear ribonucleoprotein polypeptide E	mRNA
chr4	6696899	6697013	CDS	S100P	S100 calcium binding protein P	mRNA
chr4	6697016	6697061	CDS	S100P	S100 calcium binding protein P	mRNA
chr8	11845060	11845214	CDS	CTSB	cathepsin B	mRNA
chr12	110721031	110721088	CDS	PPP1CC	protein phosphatase 1 catalytic subunit gamma	mRNA
chr19	5691599	5691644	CDS	RPL36	ribosomal protein L36	mRNA
chr11	35229128	35229407	CDS	CD44	CD44 molecule	mRNA
chr8	144789768	144789829	CDS	RPL8	ribosomal protein L8	mRNA
chr16	85806737	85806878	CDS	COX4I1	cytochrome c oxidase subunit 4I1	mRNA
chr5	179614797	179614959	CDS	HNRNPH1	heterogeneous nuclear ribonucleoprotein H1	mRNA
chr22	39522500	39522662	CDS	ATF4	activating transcription factor 4	mRNA
chr17	58005356	58005410	CDS	SRSF1	serine and arginine rich splicing factor 1	mRNA
chr4	55425375	55425469	CDS	TMEM165	transmembrane protein 165	mRNA
chr17	50750502	50750574	CDS	LUC7L3	LUC7 like 3 pre-mRNA splicing factor	mRNA
chr3	49360176	49360382	CDS	RHOA	ras homolog family member A	mRNA
chr1	183142618	183142763	CDS	LAMC1	laminin, gamma 1 (formerly LAMB2)	mRNA
chr8	119243416	119243661	CDS	MAL2	mal, T cell differentiation protein 2	mRNA
chr17	38850123	38850214	CDS	RPL23	ribosomal protein L23	mRNA
chr8	99887539	99887618	CDS	COX6C	cytochrome c oxidase subunit 6C	mRNA
chr17	79081897	79081907	CDS	ENGASE	endo-beta-N-acetylglucosaminidase	mRNA
chr12	131986513	131986525	CDS	EP400	E1A binding protein p400	mRNA
chr17	79081061	79081073	CDS	ENGASE	endo-beta-N-acetylglucosaminidase	mRNA
chr2	219162104	219162118	CDS	SLC23A3	solute carrier family 23 member 3	mRNA
chr5	96729712	96729725	CDS	CAST	calpastatin	mRNA
chr5	96730779	96730790	CDS	CAST	calpastatin	mRNA
chr4	1819384	1819402	CDS	LETM1	leucine zipper and EF-hand containing transmembrane protein 1	mRNA
chr2	241869787	241869808	CDS	RTP5	receptor (chemosensory) transporter protein 5 (putative)	mRNA
chr7	128846334	128846355	CDS	FLNC	filamin C	mRNA
chr19	15372230	15372250	CDS	AKAP8	A-kinase anchoring protein 8	mRNA
chr9	127523756	127523778	CDS	NIBAN2	niban apoptosis regulator 2	mRNA
chr18	31524702	31524727	CDS	DSG2	desmoglein 2	mRNA
chr11	62572583	62572612	CDS	EEF1G	eukaryotic translation elongation factor 1 gamma	mRNA
chr11	65624341	65624366	CDS	PCNX3	pecanex 3	mRNA
chr14	102968328	102968358	CDS	CDC42BPB	CDC42 binding protein kinase beta	mRNA
chr8	100718245	100718280	CDS	PABPC1	poly(A) binding protein cytoplasmic 1	mRNA
chr6	7585371	7585407	CDS	DSP	desmoplakin	mRNA
chr7	128084811	128084847	CDS	SND1	staphylococcal nuclease and tudor domain containing 1	mRNA
chr18	743065	743097	CDS	YES1	YES proto-oncogene 1	mRNA
chr7	128085710	128085746	CDS	SND1	staphylococcal nuclease and tudor domain containing 1	mRNA
chr1	39568914	39568939	CDS	PABPC4	poly(A) binding protein cytoplasmic 4	mRNA
chr11	130141497	130141536	CDS	APLP2	amyloid beta precursor like protein 2	mRNA
chr10	42592910	42592948	CDS	ZNF33B	zinc finger protein 33B	mRNA
chr10	96551371	96551411	CDS	TM9SF3	transmembrane 9 superfamily member 3	mRNA
chr2	46378708	46378732	CDS	EPAS1	endothelial PAS domain protein 1	mRNA
chr6	137204331	137204372	CDS	IFNGR1	interferon gamma receptor 1	mRNA

chr15	76935599	76935641	CDS	RCN2	reticulocalbin 2	mRNA
chr2	27383951	27383994	CDS	PPM1G	protein phosphatase, Mg2+/Mn2+ dependent, 1G	mRNA
chr3	194430272	194430315	CDS	ATP13A3	ATPase 13A3	mRNA
chr3	196065442	196065486	CDS	TFRC	transferrin receptor	mRNA
chr12	110339669	110339708	CDS	ATP2A2	ATPase sarcoplasmic/endoplasmic reticulum Ca2+ transporting 2	mRNA
chr1	88807737	88807775	CDS	PKN2	protein kinase N2	mRNA
chr10	93402231	93402275	CDS	MYOF	myoferlin	mRNA
chr4	77048275	77048319	CDS	CCNI	cyclin I	mRNA
chr8	39054480	39054524	CDS	ADAM9	ADAM metallopeptidase domain 9	mRNA
chr10	93401517	93401544	CDS	MYOF	myoferlin	mRNA
chr14	31144212	31144257	CDS	HECTD1	HECT domain E3 ubiquitin protein ligase 1	mRNA
chr2	232549134	232549165	CDS	TIGD1	tigger transposable element derived 1	mRNA
chr11	33339349	33339396	CDS	HIPK3	homeodomain interacting protein kinase 3	mRNA
chr8	61643385	61643433	CDS	ASPH	aspartate beta-hydroxylase	mRNA
chr19	41281223	41281275	CDS	HNRNPUL1	heterogeneous nuclear ribonucleoprotein U like 1	mRNA
chr4	39113019	39113059	CDS	KLHL5	kelch like family member 5	mRNA
chr10	96527212	96527267	CDS	TM9SF3	transmembrane 9 superfamily member 3	mRNA
chr2	96300997	96301053	CDS	SNRNP200	small nuclear ribonucleoprotein U5 subunit 200	mRNA
chr6	143502066	143502122	CDS	FUCA2	alpha-L-fucosidase 2	mRNA
chr3	105552143	105552182	CDS	ALCAM	activated leukocyte cell adhesion molecule	mRNA
chr2	218238792	218238850	CDS	ARPC2	actin related protein 2/3 complex subunit 2	mRNA
chr6	73782613	73782671	CDS	CD109	CD109 molecule	mRNA
chr8	123209390	123209442	CDS	FAM83A	family with sequence similarity 83 member A	mRNA
chr12	52518102	52518161	CDS	KRT5	keratin 5	mRNA
chr6	143502007	143502063	CDS	FUCA2	alpha-L-fucosidase 2	mRNA
chr15	76947458	76947517	CDS	RCN2	reticulocalbin 2	mRNA
chr3	128064876	128064885	CDS	SEC61A1	SEC61 translocon alpha 1 subunit	mRNA
chr12	103941467	103941527	CDS	HSP90B1	heat shock protein 90 beta family member 1	mRNA
chr19	41292244	41292286	CDS	HNRNPUL1	heterogeneous nuclear ribonucleoprotein U like 1	mRNA
chr2	44209283	44209325	CDS	PPM1B	protein phosphatase, Mg2+/Mn2+ dependent 1B	mRNA
chr13	25096985	25097032	CDS	PABPC3	poly(A) binding protein, cytoplasmic 3	mRNA
chr7	112784576	112784620	CDS	TMEM168	transmembrane protein 168	mRNA
chr6	85533739	85533780	CDS	SNX14	sorting nexin 14	mRNA
chr2	222930749	222930805	CDS	ACSL3	acyl-CoA synthetase long chain family member 3	mRNA
chr1	39569862	39569924	CDS	PABPC4	poly(A) binding protein cytoplasmic 4	mRNA
chr14	55671565	55671627	CDS	KTN1	kinectin 1	mRNA
chr14	61740512	61740575	CDS	HIF1A	hypoxia inducible factor 1 subunit alpha inhibitor	mRNA
chr9	98015363	98015426	CDS	ANP32B	acidic (leucine-rich) nuclear phosphoprotein 32 family, member B	mRNA
chr12	22062371	22062434	CDS	CMAS	cytidine monophosphate N-acetylneuraminic acid synthetase	mRNA
chr11	62571837	62571901	CDS	EEF1G	eukaryotic translation elongation factor 1 gamma	mRNA
chr10	96552927	96552991	CDS	TM9SF3	transmembrane 9 superfamily member 3	mRNA
chr8	125007423	125007487	CDS	SQLE	squalene epoxidase	mRNA
chrX	53584214	53584268	CDS	HUWE1	HECT, UBA and WWE domain containing E3 ubiquitin protein ligase 1	mRNA
chr15	100905522	100905589	CDS	ALDH1A3	aldehyde dehydrogenase 1 family member A3	mRNA
chr5	96896796	96896860	CDS	ERAP2	endoplasmic reticulum aminopeptidase 2	mRNA
chr11	62884456	62884525	CDS	SLC3A2	solute carrier family 3 member 2	mRNA
chr1	119968076	119968126	CDS	NOTCH2	notch receptor 2	mRNA
chr14	55661537	55661606	CDS	KTN1	kinectin 1	mRNA
chr5	10394081	10394143	CDS	MARCHF6	membrane associated ring-CH-type finger 6	mRNA
chr3	180962882	180962951	CDS	FXR1	FMR1 autosomal homolog 1	mRNA
chr19	36145805	36145859	CDS	CAPNS1	calpain small subunit 1	mRNA
chr14	55672629	55672701	CDS	KTN1	kinectin 1	mRNA
chr14	55637276	55637348	CDS	KTN1	kinectin 1	mRNA
chr2	182736248	182736320	CDS	DNAJC10	DnaJ heat shock protein family (Hsp40) member C10	mRNA
chr5	135346009	135346057	CDS	MACROH2A1	macroH2A.1 histone	mRNA
chr6	18258330	18258405	CDS	DEK	DEK proto-oncogene	mRNA
chrX	53563745	53563821	CDS	HUWE1	HECT, UBA and WWE domain containing E3 ubiquitin protein ligase 1	mRNA
chr12	49761703	49761779	CDS	TMBIM6	transmembrane BAX inhibitor motif containing 6	mRNA
chr4	186600298	186600360	CDS	FAT1	FAT atypical cadherin 1	mRNA
chr5	141578224	141578300	CDS	DIAPH1	diaphanous related formin 1	mRNA
chr15	58611031	58611107	CDS	ADAM10	ADAM metallopeptidase domain 10	mRNA

chr3	37327702	37327779	CDS	GOLGA4	golgin A4	mRNA
chr2	27383995	27384073	CDS	PPM1G	protein phosphatase, Mg2+/Mn2+ dependent, 1G	mRNA
chr5	34938891	34938969	CDS	DNAJC21	DnaJ heat shock protein family (Hsp40) member C21	mRNA
chr1	244858728	244858807	CDS	HNRNPU	heterogeneous nuclear ribonucleoprotein U	mRNA
chr3	149177889	149177964	CDS	CP	ceruloplasmin	mRNA
chr10	32911885	32911933	CDS	ITGB1	integrin subunit beta 1	mRNA
chr5	179726679	179726759	CDS	CANX	calnexin	mRNA
chr3	112638173	112638250	CDS	CCDC80	coiled-coil domain containing 80	mRNA
chr9	105385421	105385452	CDS	SLC44A1	solute carrier family 44 member 1	mRNA
chrX	53562142	53562192	CDS	HUWE1	HECT, UBA and WWE domain containing E3 ubiquitin protein ligase 1	mRNA
chr3	31625992	31626074	CDS	STT3B	STT3 oligosaccharyltransferase complex catalytic subunit B	mRNA
chr8	39025802	39025884	CDS	ADAM9	ADAM metallopeptidase domain 9	mRNA
chr14	55648033	55648115	CDS	KTN1	kinectin 1	mRNA
chr8	39082991	39083073	CDS	ADAM9	ADAM metallopeptidase domain 9	mRNA
chr12	53297849	53297924	CDS	PFDN5	prefoldin subunit 5	mRNA
chr11	34076559	34076642	CDS	CAPRIN1	cell cycle associated protein 1	mRNA
chr11	65624470	65624523	CDS	PCNX3	pecanex 3	mRNA
chr1	156135900	156135984	CDS	LMNA	lamin A/C	mRNA
chr14	55672928	55673012	CDS	KTN1	kinectin 1	mRNA
chr14	55675834	55675918	CDS	KTN1	kinectin 1	mRNA
chr5	128112880	128112933	CDS	SLC12A2	solute carrier family 12 member 2	mRNA
chr1	183134723	183134809	CDS	LAMC1	laminin, gamma 1 (formerly LAMB2)	mRNA
chr12	110326389	110326475	CDS	ATP2A2	ATPase sarcoplasmic/endoplasmic reticulum Ca2+ transporting 2	mRNA
chr2	54631368	54631410	CDS	SPTBN1	spectrin beta, non-erythrocytic 1	mRNA
chr14	55633234	55633321	CDS	KTN1	kinectin 1	mRNA
chr14	55636448	55636536	CDS	KTN1	kinectin 1	mRNA
chr1	10403070	10403122	CDS	PGD	phosphogluconate dehydrogenase	mRNA
chr6	31626771	31626862	CDS	PRRC2A	proline rich coiled-coil 2A	mRNA
chr3	31622202	31622283	CDS	STT3B	STT3 oligosaccharyltransferase complex catalytic subunit B	mRNA
chr17	58005825	58005909	CDS	SRSF1	serine and arginine rich splicing factor 1	mRNA
chr3	53228055	53228149	CDS	TKT	transketolase	mRNA
chr2	43963612	43963655	CDS	LRPPRC	leucine rich pentatricopeptide repeat containing	mRNA
chr7	105110550	105110585	CDS	KMT2E	lysine methyltransferase 2E	mRNA
chr10	71820239	71820335	CDS	PSAP	prosaposin	mRNA
chr12	52517901	52517992	CDS	KRT5	keratin 5	mRNA
chr1	183118084	183118146	CDS	LAMC1	laminin, gamma 1 (formerly LAMB2)	mRNA
chr1	156333546	156333638	CDS	CCT3	chaperonin containing TCP1 subunit 3	mRNA
chr3	121717032	121717099	CDS	GOLGB1	golgin B1	mRNA
chr15	98942922	98943021	CDS	IGF1R	insulin like growth factor 1 receptor	mRNA
chr10	74090097	74090121	CDS	VCL	vinculin	mRNA
chr5	10426389	10426489	CDS	MARCHF6	membrane associated ring-CH-type finger 6	mRNA
chr12	21646916	21647016	CDS	LDHB	lactate dehydrogenase B	mRNA
chr3	53229006	53229107	CDS	TKT	transketolase	mRNA
chr12	6236191	6236275	CDS	CD9	CD9 molecule	mRNA
chr12	103931520	103931623	CDS	HSP90B1	heat shock protein 90 beta family member 1	mRNA
chr17	75729287	75729390	CDS	ITGB4	integrin subunit beta 4	mRNA
chr2	55871050	55871123	CDS	EFEMP1	EGF containing fibulin extracellular matrix protein 1	mRNA
chr7	45916566	45916667	CDS	IGFBP3	insulin like growth factor binding protein 3	mRNA
chr1	230250456	230250560	CDS	GALNT2	polypeptide N-acetylgalactosaminyltransferase 2	mRNA
chr2	61877392	61877496	CDS	CCT4	chaperonin containing TCP1 subunit 4	mRNA
chr4	118308437	118308542	CDS	PRSS12	protease, serine, 12 (neurotrypsin, motopsin)	mRNA
chr3	105547192	105547253	CDS	ALCAM	activated leukocyte cell adhesion molecule	mRNA
chr1	23693806	23693913	CDS	RPL11	ribosomal protein L11	mRNA
chr8	116858351	116858458	CDS	RAD21	RAD21 cohesin complex component	mRNA
chr1	119966375	119966482	CDS	NOTCH2	notch receptor 2	mRNA
chr5	133096097	133096204	CDS	HSPA4	heat shock protein family A (Hsp70) member 4	mRNA
chr1	114726982	114727090	CDS	CSDE1	cold shock domain containing E1	mRNA
chr19	38283717	38283797	CDS	SPINT2	serine peptidase inhibitor, Kunitz type 2	mRNA
chr5	141574066	141574139	CDS	DIAPH1	diaphanous related formin 1	mRNA
chr8	124543086	124543194	CDS	NDUFB9	NADH:ubiquinone oxidoreductase subunit B9	mRNA
chr18	46091708	46091816	CDS	ATP5F1A	ATP synthase F1 subunit alpha	mRNA

chr22	50280905	50280973	CDS	PLXNB2	plexin B2	mRNA
chr15	90476695	90476803	CDS	IQGAP1	IQ motif containing GTPase activating protein 1	mRNA
chr2	74369423	74369491	CDS	DCTN1	dynactin subunit 1	mRNA
chr22	45535244	45535285	CDS	FBLN1	fibulin 1	mRNA
chr1	119969578	119969687	CDS	NOTCH2	notch receptor 2	mRNA
chr5	141579116	141579196	CDS	DIAPH1	diaphanous related formin 1	mRNA
chr7	2379133	2379242	CDS	EIF3B	eukaryotic translation initiation factor 3 subunit B	mRNA
chr2	61876943	61877052	CDS	CCT4	chaperonin containing TCP1 subunit 4	mRNA
chr8	73292688	73292797	CDS	RPL7	ribosomal protein L7	mRNA
chr1	186326103	186326163	CDS	TPR	translocated promoter region	mRNA
chr1	186326165	186326213	CDS	TPR	translocated promoter region	mRNA
chr1	116384013	116384059	CDS	ATP1A1	ATPase Na+/K+ transporting subunit alpha 1	mRNA
chr1	116384065	116384124	CDS	ATP1A1	ATPase Na+/K+ transporting subunit alpha 1	mRNA
chr10	32920273	32920321	CDS	ITGB1	integrin subunit beta 1	mRNA
chr8	116857379	116857473	CDS	RAD21	RAD21 cohesin complex component	mRNA
chr19	3981338	3981452	CDS	EEF2	eukaryotic translation elongation factor 2	mRNA
chr5	179719667	179719781	CDS	CANX	calnexin	mRNA
chr10	93369649	93369763	CDS	MYOF	myoferlin	mRNA
chr2	241256288	241256335	CDS	HDLBP	high density lipoprotein binding protein	mRNA
chr8	100717772	100717888	CDS	PABPC1	poly(A) binding protein cytoplasmic 1	mRNA
chr5	141576755	141576817	CDS	DIAPH1	diaphanous related formin 1	mRNA
chr14	55671784	55671877	CDS	KTN1	kinectin 1	mRNA
chr8	11847051	11847168	CDS	CTSB	cathepsin B	mRNA
chr12	21654542	21654659	CDS	LDHB	lactate dehydrogenase B	mRNA
chr14	55627911	55628028	CDS	KTN1	kinectin 1	mRNA
chr18	35689202	35689290	CDS	GALNT1	polypeptide N-acetylgalactosaminyltransferase 1	mRNA
chr11	88309202	88309276	CDS	CTSC	cathepsin C	mRNA
chr21	31667257	31667375	CDS	SOD1	superoxide dismutase 1	mRNA
chr1	43620873	43620992	CDS	PTPRF	protein tyrosine phosphatase receptor type F	mRNA
chr3	184334726	184334845	CDS	EIF4G1	eukaryotic translation initiation factor 4 gamma 1	mRNA
chr5	133097166	133097286	CDS	HSPA4	heat shock protein family A (Hsp70) member 4	mRNA
chr16	87841049	87841155	CDS	SLC7A5	solute carrier family 7 member 5	mRNA
chr12	21637070	21637194	CDS	LDHB	lactate dehydrogenase B	mRNA
chr1	30995064	30995178	CDS	PUM1	pumilio RNA binding family member 1	mRNA
chr2	10802498	10802623	CDS	PDIA6	protein disulfide isomerase family A member 6	mRNA
chr8	125021777	125021900	CDS	SQLE	squalene epoxidase	mRNA
chr3	41224525	41224650	CDS	CTNNB1	catenin beta 1	mRNA
chr9	87730373	87730498	CDS	CTSL	cathepsin L	mRNA
chr3	128626732	128626832	CDS	RPN1	ribophorin I	mRNA
chr18	36109618	36109683	CDS	SLC39A6	solute carrier family 39 member 6	mRNA
chr11	10802293	10802421	CDS	EIF4G2	eukaryotic translation initiation factor 4 gamma 2	mRNA
chr2	10791794	10791925	CDS	PDIA6	protein disulfide isomerase family A member 6	mRNA
chr10	71821875	71822007	CDS	PSAP	prosaposin	mRNA
chr1	55146997	55147034	CDS	USP24	ubiquitin specific peptidase 24	mRNA
chr12	110327563	110327674	CDS	ATP2A2	ATPase sarcoplasmic/endoplasmic reticulum Ca2+ transporting 2	mRNA
chr5	139321933	139322029	CDS	MATR3	matrin 3	mRNA
chr3	112638102	112638149	CDS	CCDC80	coiled-coil domain containing 80	mRNA
chr3	25633924	25633994	CDS	TOP2B	DNA topoisomerase II beta	mRNA
chr14	55634525	55634658	CDS	KTN1	kinectin 1	mRNA
chr17	41515884	41516010	CDS	KRT15	keratin 15	mRNA
chr1	23694659	23694796	CDS	RPL11	ribosomal protein L11	mRNA
chr6	158783540	158783589	CDS	EZR	ezrin	mRNA
chr6	31626070	31626162	CDS	PRRC2A	proline rich coiled-coil 2A	mRNA
chr1	114730507	114730648	CDS	CSDE1	cold shock domain containing E1	mRNA
chr14	55629956	55630097	CDS	KTN1	kinectin 1	mRNA
chr6	34425079	34425221	CDS	RPS10	ribosomal protein S10	mRNA
chr6	7558115	7558249	CDS	DSP	desmoplakin	mRNA
chr2	218234352	218234454	CDS	ARPC2	actin related protein 2/3 complex subunit 2	mRNA
chr15	76948409	76948471	CDS	RCN2	reticulocalbin 2	mRNA
chr11	62570964	62571108	CDS	EEF1G	eukaryotic translation elongation factor 1 gamma	mRNA
chr1	1053841	1053894	CDS	AGRN	agrin	mRNA

chr11	1761323	1761468	CDS	CTSD	cathepsin D	mRNA
chr3	184324200	184324347	CDS	EIF4G1	eukaryotic translation initiation factor 4 gamma 1	mRNA
chr8	116726061	116726162	CDS	EIF3H	eukaryotic translation initiation factor 3 subunit H	mRNA
chr11	111296108	111296207	CDS	COLCA1	colorectal cancer associated 1	mRNA
chr17	63766027	63766045	CDS	CCDC47	coiled-coil domain containing 47	mRNA
chr5	136049490	136049580	CDS	TGFBI	transforming growth factor beta induced	mRNA
chr1	23692608	23692759	CDS	RPL11	ribosomal protein L11	mRNA
chr19	38721537	38721688	CDS	ACTN4	actinin alpha 4	mRNA
chr8	39026676	39026810	CDS	ADAM9	ADAM metallopeptidase domain 9	mRNA
chr1	81907083	81907214	CDS	ADGRL2	adhesion G protein-coupled receptor L2	mRNA
chr1	116398929	116399016	CDS	ATP1A1		mRNA
chr11	102401538	102401692	CDS	TMEM123	transmembrane protein 123	mRNA
chr1	209616470	209616624	CDS	LAMB3	laminin subunit beta 3	mRNA
chr6	43159870	43159957	CDS	PTK7	protein tyrosine kinase 7 (inactive)	mRNA
chr8	39023170	39023277	CDS	ADAM9	ADAM metallopeptidase domain 9	mRNA
chr8	39023279	39023325	CDS	ADAM9	ADAM metallopeptidase domain 9	mRNA
chr8	119240164	119240320	CDS	MAL2	mal, T cell differentiation protein 2	mRNA
chr14	64186473	64186579	CDS	SYNE2	spectrin repeat containing nuclear envelope protein 2	mRNA
chr18	743259	743285	CDS	YES1	YES proto-oncogene 1	mRNA
chr16	85805709	85805864	CDS	COX4I1	cytochrome c oxidase subunit 4I1	mRNA
chr10	32911966	32912124	CDS	ITGB1	integrin subunit beta 1	mRNA
chr11	71437795	71437943	CDS	DHCR7	7-dehydrocholesterol reductase	mRNA
chr1	156320864	156321025	CDS	CCT3	chaperonin containing TCP1 subunit 3	mRNA
chr7	45917212	45917374	CDS	IGFBP3	insulin like growth factor binding protein 3	mRNA
chr16	85804936	85805104	CDS	COX4I1	cytochrome c oxidase subunit 4I1	mRNA
chrX	53561780	53561924	CDS	HUWE1	HECT, UBA and WWE domain containing E3 ubiquitin protein ligase 1	mRNA
chr1	116398620	116398735	CDS	ATP1A1	ATPase Na+/K+ transporting subunit alpha 1	mRNA
chr1	116398749	116398789	CDS	ATP1A1	ATPase Na+/K+ transporting subunit alpha 1	mRNA
chr17	42999932	43000102	CDS	RPL27	ribosomal protein L27	mRNA
chr17	7890570	7890683	CDS	CHD3	chromodomain helicase DNA binding protein 3	mRNA
chr8	39042070	39042117	CDS	ADAM9	ADAM metallopeptidase domain 9	mRNA
chr1	54853453	54853609	CDS	DHCR24	24-dehydrocholesterol reductase	mRNA
chr2	172469188	172469302	CDS	ITGA6	integrin subunit alpha 6	mRNA
chr10	32919884	32919984	CDS	ITGB1	integrin subunit beta 1	mRNA
chr6	137203546	137203675	CDS	IFNGR1	interferon gamma receptor 1	mRNA
chr8	39077227	39077297	CDS	ADAM9	ADAM metallopeptidase domain 9	mRNA
chr8	39077304	39077411	CDS	ADAM9	ADAM metallopeptidase domain 9	mRNA
chr12	56643402	56643587	CDS	ATP5F1B	ATP synthase F1 subunit beta	mRNA
chr10	119049804	119049985	CDS	EIF3A	eukaryotic translation initiation factor 3 subunit A	mRNA
chr19	48913053	48913128	CDS	NUCB1	nucleobindin 1	mRNA
chr1	156744162	156744348	CDS	HDGF	heparin binding growth factor	mRNA
chr3	49968148	49968224	CDS	RBM6	RNA binding motif protein 6	mRNA
chr10	71819713	71819900	CDS	PSAP	prosaposin	mRNA
chr14	20455913	20456101	CDS	APEX1	apurinic/apyrimidinic endodeoxyribonuclease 1	mRNA
chr1	183110492	183110606	CDS	LAMC1	laminin, gamma 1 (formerly LAMB2)	mRNA
chr8	100721390	100721472	CDS	PABPC1	poly(A) binding protein cytoplasmic 1	mRNA
chr8	39017218	39017277	CDS	ADAM9	ADAM metallopeptidase domain 9	mRNA
chr8	39017313	39017396	CDS	ADAM9	ADAM metallopeptidase domain 9	mRNA
chr19	40374518	40374620	CDS	PLD3	phospholipase D family member 3	mRNA
chr1	43619321	43619408	CDS	PTPRF	protein tyrosine phosphatase receptor type F	mRNA
chr8	47807136	47807299	CDS	PRKDC	protein kinase, DNA-activated, catalytic subunit	mRNA
chr3	196075240	196075360	CDS	TFRC	transferrin receptor	mRNA
chr11	71438941	71439083	CDS	DHCR7	7-dehydrocholesterol reductase	mRNA
chr1	183130343	183130549	CDS	LAMC1	laminin, gamma 1 (formerly LAMB2)	mRNA
chr10	119042006	119042212	CDS	EIF3A	eukaryotic translation initiation factor 3 subunit A	mRNA
chr1	39568801	39568900	CDS	PABPC4	poly(A) binding protein cytoplasmic 4	mRNA
chr11	130122369	130122425	CDS	APLP2	amyloid beta precursor like protein 2	mRNA
chr12	110343358	110343418	CDS	ATP2A2	ATPase sarcoplasmic/endoplasmic reticulum Ca2+ transporting 2	mRNA
chr12	57718145	57718359	CDS	OS9	OS9 endoplasmic reticulum lectin	mRNA
chr9	19378707	19378918	CDS	RPS6	ribosomal protein S6	mRNA
chr3	49122162	49122346	CDS	LAMB2	laminin subunit beta 2	mRNA

chr3	105524417	105524491	CDS	ALCAM	activated leukocyte cell adhesion molecule	mRNA
chr3	184324877	184325114	CDS	EIF4G1	eukaryotic translation initiation factor 4 gamma 1	mRNA
chr5	181242215	181242345	CDS	RACK1	receptor for activated C kinase 1	mRNA
chr11	34076250	34076474	CDS	CAPRIN1	cell cycle associated protein 1	mRNA
chr10	119042359	119042512	CDS	EIF3A	eukaryotic translation initiation factor 3 subunit A	mRNA
chr6	7580672	7580921	CDS	DSP	desmoplakin	mRNA
chr1	43588772	43589000	CDS	PTPRF	protein tyrosine phosphatase receptor type F	mRNA
chr14	55612303	55612389	CDS	KTN1	kinectin 1	mRNA
chr4	186706562	186706835	CDS	FAT1	FAT atypical cadherin 1	mRNA
chr8	118110051	118110141	CDS	EXT1	exostosin glycosyltransferase 1	mRNA
chr1	214645916	214646132	CDS	CENPF	centromere protein F	mRNA
chr4	186707201	186707366	CDS	FAT1	FAT atypical cadherin 1	mRNA
chr17	41515487	41515692	CDS	KRT15	keratin 15	mRNA
chr3	121695488	121695667	CDS	GOLGB1	golgin B1	mRNA
chr4	186618197	186618463	CDS	FAT1	FAT atypical cadherin 1	mRNA
chr6	7584730	7584928	CDS	DSP	desmoplakin	mRNA
chr6	7579501	7579830	CDS	DSP	desmoplakin	mRNA
chr3	49531186	49531256	CDS	DAG1	dystroglycan 1	mRNA
chr14	39180764	39180962	CDS	PNN	pinin, desmosome associated protein	mRNA
chr11	70420336	70420615	CDS	CTTN	cortactin	mRNA
chr6	7583435	7583518	CDS	DSP	desmoplakin	mRNA
chr11	1759015	1759087	CDS	CSTD	cathepsin D	mRNA
chr3	9249370	9249449	five_prime_U	SRGAP3	SLIT-ROBO Rho GTPase activating protein 3	mRNA
chr8	124999365	124999482	five_prime_U	SQLE	squalene epoxidase	mRNA
chr6	34426031	34426069	five_prime_U	RPS10	ribosomal protein S10	mRNA
chr4	108620579	108620600	five_prime_U	RPL34	ribosomal protein L34	mRNA
chr8	73293598	73293629	five_prime_U	RPL7	ribosomal protein L7	mRNA
chr20	45670212	45670256	five_prime_U	WFDC11	WAP four-disulfide core domain 11	mRNA
chr18	3451868	3451953	five_prime_U	TGIF1	TGFB induced factor homeobox 1	mRNA
chr19	50637895	50637920	five_prime_U	SYT3	synaptotagmin 3	mRNA
chr16	16379064	16379118	five_prime_U	NPIPA7	nuclear pore complex interacting protein family, member A7	mRNA
chr16	16379141	16379200	five_prime_U	NPIPA7	nuclear pore complex interacting protein family, member A7	mRNA
chr15	98649436	98649510	five_prime_U	IGF1R	insulin like growth factor 1 receptor	mRNA
chr3	9249263	9249347	five_prime_U	SRGAP3	SLIT-ROBO Rho GTPase activating protein 3	mRNA
chr3	112640729	112640814	five_prime_U	CCDC80	coiled-coil domain containing 80	mRNA
chr8	124999328	124999361	five_prime_U	SQLE	squalene epoxidase	mRNA
chr2	61537639	61537809	five_prime_U	XPO1	exportin 1	mRNA
chr6	30616608	30616642	five_prime_U	PPP1R10	protein phosphatase 1 regulatory subunit 10	mRNA
chr19	48704195	48704208	intron	FUT2	fucosyltransferase 2 (secretor status included)	mRNA
chr13	45337197	45337320	intron	TPT1	tumor protein, translationally-controlled 1	mRNA
chr8	11844018	11844167	intron	CTSB	cathepsin B	mRNA
chr21	39342497	39342570	intron	HMGN1	high mobility group nucleosome binding domain 1	mRNA
chr21	39342574	39342692	intron	HMGN1	high mobility group nucleosome binding domain 1	mRNA
chr5	127550617	127550634	intron	PRRC1	proline rich coiled-coil 1	mRNA
chr12	63623807	63623824	intron	DPY19L2	dpy-19 like 2	mRNA
chr17	44720241	44720260	intron	DBF4B	DBF4 zinc finger B	mRNA
chr11	68894570	68894589	intron	MRPL21	mitochondrial ribosomal protein L21	mRNA
chr1	53963299	53963319	intron	LRRC42	leucine rich repeat containing 42	mRNA
chr15	68081526	68081549	intron	PIAS1	protein inhibitor of activated STAT 1	mRNA
chr3	127966070	127966093	intron	KBTBD12	kelch repeat and BTB domain containing 12	mRNA
chr1	108573525	108573549	intron	FAM102B	family with sequence similarity 102 member B	mRNA
chr4	76480679	76480704	intron	SHROOM3	shroom family member 3	mRNA
chr5	112164624	112164647	intron	EPB41L4A	erythrocyte membrane protein band 4.1 like 4A	mRNA
chr16	4839267	4839292	intron	GLYR1	glyoxylate reductase 1 homolog	mRNA
chr13	33849056	33849082	intron	RFC3	replication factor C subunit 3	mRNA
chr20	53398567	53398594	intron	TSHZ2	teashirt zinc finger homeobox 2	mRNA
chr11	94175863	94175889	intron	PANX1	pannexin 1	mRNA
chr19	15383382	15383408	intron	AKAP8L	A-kinase anchoring protein 8 like	mRNA
chr10	72917431	72917459	intron	OIT3	oncoprotein induced transcript 3	mRNA
chr19	6717633	6717659	intron	C3	complement C3	mRNA
chr19	6717661	6717690	intron	C3	complement C3	mRNA

chr14	50727603	50727628	intron	NIN	ninein	mRNA
chr1	18858431	18858462	intron	TAS1R2	taste receptor, type 1, member 2	mRNA
chr6	3303281	3303312	intron	SLC22A23	solute carrier family 22 member 23	mRNA
chr17	56984915	56984941	intron	SCPEP1	serine carboxypeptidase 1	mRNA
chr15	29760020	29760052	intron	TJP1	tight junction protein 1	mRNA
chr11	88322110	88322139	intron	CTSC	cathepsin C	mRNA
chr19	35353298	35353323	intron	FFAR1	free fatty acid receptor 1	mRNA
chr16	89740613	89740646	intron	ZNF276	zinc finger protein 276	mRNA
chr2	219555449	219555482	intron	OBSL1	obscurin like cytoskeletal adaptor 1	mRNA
chr12	122020317	122020339	intron	BCL7A	B-cell CLL/lymphoma 7 protein family member A	mRNA
chr17	14328815	14328848	intron	HS3ST3B1	heparan sulfate-glucosamine 3-sulfotransferase 3B1	mRNA
chr11	117907881	117907907	intron	TMPRSS13	transmembrane serine protease 13	mRNA
chr17	75263693	75263722	intron	MRPS7	mitochondrial ribosomal protein S7	mRNA
chr5	61114482	61114513	intron	NDUFAF2	NADH:ubiquinone oxidoreductase complex assembly factor 2	mRNA
chr19	53158879	53158914	intron	ZNF347	zinc finger protein 347	mRNA
chr3	50101291	50101324	intron	RBM5	RNA binding motif protein 5	mRNA
chr18	79967425	79967458	intron	HSBP1L1	heat shock factor binding protein 1-like 1	mRNA
chr22	17003836	17003870	intron	GAB4	GRB2 associated binding protein family member 4	mRNA
chr12	4676051	4676086	intron	NDUFA9	NADH:ubiquinone oxidoreductase subunit A9	mRNA
chr12	65954995	65955027	intron	HMGA2	high mobility group AT-hook 2	mRNA
chr12	95934858	95934883	intron	CCDC38	coiled-coil domain containing 38	mRNA
chr15	45598094	45598129	intron	BLOC1S6	biogenesis of lysosomal organelles complex 1 subunit 6	mRNA
chr3	185293013	185293046	intron	MAP3K13	mitogen-activated protein kinase kinase kinase 13	mRNA
chr2	61887742	61887777	intron	CCT4	chaperonin containing TCP1 subunit 4	mRNA
chr9	122874692	122874722	intron	RC3H2	ring finger and CCCH-type domains 2	mRNA
chr16	67615430	67615460	intron	CTCF	CCCTC-binding factor	mRNA
chr2	177249593	177249630	intron	NFE2L2	nuclear factor, erythroid 2 like 2	mRNA
chr21	15063795	15063830	intron	NRIP1	nuclear receptor interacting protein 1	mRNA
chr1	179544488	179544524	intron	AXDND1	axonemal dynein light chain domain containing 1	mRNA
chr12	26978783	26978815	intron	TM7SF3	transmembrane 7 superfamily member 3	mRNA
chr7	102352533	102352569	intron	SPDYE6	speedy/RINGO cell cycle regulator family member E6	mRNA
chr7	100077596	100077619	intron	ZNF3	zinc finger protein 3	mRNA
chr19	50258509	50258545	intron	MYH14	myosin heavy chain 14	mRNA
chr3	142472075	142472113	intron	ATR	ATR serine/threonine kinase	mRNA
chr6	47002365	47002404	intron	ADGRF1	adhesion G protein-coupled receptor F1	mRNA
chr22	46525532	46525569	intron	CELSR1	cadherin EGF LAG seven-pass G-type receptor 1	mRNA
chr17	63746922	63746961	intron	CCDC47	coiled-coil domain containing 47	mRNA
chr9	33348453	33348492	intron	NFX1	nuclear transcription factor, X-box binding 1	mRNA
chr9	116612382	116612416	intron	ASTN2	astrotactin 2	mRNA
chr1	20775334	20775374	intron	HP1BP3	heterochromatin protein 1 binding protein 3	mRNA
chr9	123079550	123079587	intron	RABGAP1	RAB GTPase activating protein 1	mRNA
chr3	167692439	167692469	intron	PDCD10	programmed cell death 10	mRNA
chr14	51639142	51639177	intron	FRMD6	FERM domain containing 6	mRNA
chr11	13394774	13394813	intron	BTBD10	BTB domain containing 10	mRNA
chr5	80651419	80651460	intron	DHFR	dihydrofolate reductase	mRNA
chr9	83054708	83054736	intron	RASEF	RAS and EF-hand domain containing	mRNA
chr18	37075575	37075611	intron	KIAA1328	KIAA1328	mRNA
chr1	27670207	27670249	intron	IFI6	interferon alpha inducible protein 6	mRNA
chr11	32587104	32587144	intron	EIF3M	eukaryotic translation initiation factor 3 subunit M	mRNA
chr15	72370508	72370541	intron	HEXA	hexosaminidase subunit alpha	mRNA
chr19	44095039	44095082	intron	ZNF224	zinc finger protein 224	mRNA
chr17	51250075	51250104	intron	MBTD1	mbt domain containing 1	mRNA
chr7	99485420	99485441	intron	ZNF789	zinc finger protein 789	mRNA
chr19	19932087	19932124	intron	ZNF93	zinc finger protein 93	mRNA
chr8	11807158	11807199	intron	FDFT1	farnesyl-diphosphate farnesyltransferase 1	mRNA
chr12	20860261	20860303	intron	SLCO1B3	solute carrier organic anion transporter family member 1B3	mRNA
chr18	80162316	80162359	intron	PARD6G	par-6 family cell polarity regulator gamma	mRNA
chr13	39044284	39044329	intron	NHLRC3	NHL repeat containing 3	mRNA
chr2	207531487	207531522	intron	CREB1	cAMP responsive element binding protein 1	mRNA
chrX	41649589	41649624	intron	CASK	calcium/calmodulin dependent serine protein kinase	mRNA
chr19	43496028	43496063	intron	PHLDB3	pleckstrin homology like domain family B member 3	mRNA

chr3	131001366	131001412	intron	ATP2C1	ATPase secretory pathway Ca2+ transporting 1	mRNA
chr8	43063251	43063294	intron	FNTA	farnesyltransferase	mRNA
chr9	124528327	124528362	intron	NR6A1	nuclear receptor subfamily 6 group A member 1	mRNA
chr15	89591694	89591733	intron	TICRR	TOPBP1 interacting checkpoint and replication regulator	mRNA
chr4	37952410	37952451	intron	TBC1D1	TBC1 domain family member 1	mRNA
chr6	63658799	63658842	intron	PHF3	PHD finger protein 3	mRNA
chr9	33347912	33347951	intron	NFX1	nuclear transcription factor, X-box binding 1	mRNA
chr11	35194842	35194883	intron	CD44	CD44 molecule	mRNA
chr16	67007435	67007468	intron	CES4A	carboxylesterase 4A	mRNA
chr3	142472210	142472250	intron	ATR	ATR serine/threonine kinase	mRNA
chr9	127184127	127184175	intron	RALGPS1	Ral GEF with PH domain and SH3 binding motif 1	mRNA
chr8	11319047	11319080	intron	MTMR9	myotubularin related protein 9	mRNA
chr22	16978267	16978296	intron	GAB4	GRB2 associated binding protein family member 4	mRNA
chr16	61725898	61725930	intron	CDH8	cadherin 8	mRNA
chr11	31574770	31574792	intron	ELP4	elongator acetyltransferase complex subunit 4	mRNA
chr1	174799960	174799992	intron	RABGAP1L	RAB GTPase activating protein 1 like	mRNA
chr14	55136212	55136262	intron	LGALS3	galectin 3	mRNA
chr2	44229614	44229652	intron	PPM1B	protein phosphatase, Mg2+/Mn2+ dependent 1B	mRNA
chr3	185933815	185933863	intron	TRA2B	Transformer-2 protein homolog beta	mRNA
chr22	25086411	25086460	intron	KIAA1671	KIAA1671	mRNA
chr12	131712611	131712658	intron	SFSWAP	splicing factor SWAP	mRNA
chr17	81834199	81834240	intron	PPP1R27	protein phosphatase 1, regulatory subunit 27	mRNA
chr2	30606984	30607016	intron	LCLAT1	lysocardiolipin acyltransferase 1	mRNA
chr2	138568445	138568490	intron	SPOPL	speckle type BTB/POZ protein like	mRNA
chr9	87918949	87918993	intron	SPATA31C1	SPATA31 subfamily C member 1	mRNA
chr16	10448627	10448678	intron	ATF7IP2	activating transcription factor 7 interacting protein 2	mRNA
chr19	9813350	9813399	intron	FBXL12	F-box and leucine rich repeat protein 12	mRNA
chr17	4110880	4110915	intron	ZZEF1	zinc finger ZZ-type and EF-hand domain containing 1	mRNA
chr19	58000396	58000427	intron	ZNF606	zinc finger protein 606	mRNA
chr7	151501981	151502018	intron	RHEB	Ras homolog, mTORC1 binding	mRNA
chr15	100912467	100912519	intron	ALDH1A3	aldehyde dehydrogenase 1 family member A3	mRNA
chr4	15358478	15358528	intron	C1QTNF7	C1q and TNF related 7	mRNA
chr16	3051190	3051236	intron	MMP25	matrix metallopeptidase 25	mRNA
chr12	51202336	51202375	intron	POU6F1	POU class 6 homeobox 1	mRNA
chr8	30176792	30176824	intron	DCTN6	dynactin subunit 6	mRNA
chr21	15794857	15794909	intron	USP25	ubiquitin specific peptidase 25	mRNA
chr2	229180778	229180825	intron	PID1	phosphotyrosine interaction domain containing 1	mRNA
chr6	111311973	111312007	intron	REV3L	REV3 like, DNA directed polymerase zeta catalytic subunit	mRNA
chr3	54638639	54638694	intron	CACNA2D3	calcium voltage-gated channel auxiliary subunit alpha2delta 3	mRNA
chr1	77745527	77745577	intron	USP33	ubiquitin specific peptidase 33	mRNA
chr10	96134614	96134670	intron	ZNF518A	zinc finger protein 518A	mRNA
chr14	104704663	104704698	intron	INF2	inverted formin, FH2 and WH2 domain containing	mRNA
chr4	158987520	158987569	intron	C4orf45	chromosome 4 open reading frame 45	mRNA
chr1	146074581	146074636	intron	NBPF10	NBPF member 10	mRNA
chr8	30414271	30414315	intron	RBPMS	RNA binding protein, mRNA processing factor	mRNA
chr22	16977670	16977726	intron	GAB4	GRB2 associated binding protein family member 4	mRNA
chr14	105370527	105370557	intron	PACS2	phosphofurin acidic cluster sorting protein 2	mRNA
chr19	51355315	51355371	intron	ETFB	electron transfer flavoprotein subunit beta	mRNA
chr9	21892790	21892842	intron	MTAP	methylthioadenosine phosphorylase	mRNA
chr2	177261848	177261909	intron	NFE2L2	nuclear factor, erythroid 2 like 2	mRNA
chr14	55581091	55581145	intron	KTN1	kinectin 1	mRNA
chr16	50074800	50074858	intron	HEATR3	HEAT repeat containing 3	mRNA
chr1	70226461	70226509	intron	SRSF11	serine and arginine rich splicing factor 11	mRNA
chr15	45598164	45598221	intron	BLOC1S6	biogenesis of lysosomal organelles complex 1 subunit 6	mRNA
chr2	233700887	233700949	intron	UGT1A8	UDP glucuronosyltransferase 1 family, polypeptide A8	mRNA
chr2	200890885	200890950	intron	NIF3L1	NGG1 interacting factor 3 like 1	mRNA
chr6	36503925	36503974	intron	STK38	serine/threonine kinase 38	mRNA
chr1	205748089	205748145	intron	NUCKS1	nuclear casein kinase and cyclin-dependent kinase substrate 1	mRNA
chr8	115570800	115570864	intron	TRPS1	transcriptional repressor GATA binding 1	mRNA
chr7	158640350	158640384	intron	NCAPG2	non-SMC condensin II complex subunit G2	mRNA
chr8	11782698	11782739	intron	NEIL2	nei like DNA glycosylase 2	mRNA

chr2	3515579	3515629	intron	ADI1	acireductone dioxygenase 1	mRNA
chr1	120432693	120432759	intron	NBPF8	NBPF member 8	mRNA
chr5	45575681	45575744	intron	HCN1	hyperpolarization activated cyclic nucleotide gated potassium channel 1	mRNA
chr1	86350440	86350485	intron	ODF2L	outer dense fiber of sperm tails 2 like	mRNA
chr11	71477302	71477338	intron	NADSYN1	NAD synthetase 1	mRNA
chr2	210026515	210026563	intron	KANSL1L	KAT8 regulatory NSL complex subunit 1 like	mRNA
chr1	186412028	186412093	intron	ODR4	odr-4 GPCR localization factor homolog	mRNA
chr14	51644309	51644366	intron	FRMD6	FERM domain containing 6	mRNA
chr14	21263136	21263206	intron	HNRNPC	heterogeneous nuclear ribonucleoprotein C	mRNA
chr1	223770200	223770254	intron	CAPN2	calpain 2	mRNA
chr18	80012968	80013039	intron	TXNL4A	thioredoxin like 4A	mRNA
chr1	151213417	151213455	intron	PIP5K1A	phosphatidylinositol-4-phosphate 5-kinase type 1 alpha	mRNA
chr6	79201778	79201820	intron	HMGN3	high mobility group nucleosomal binding domain 3	mRNA
chr5	180809779	180809834	intron	MGAT1	alpha-1,3-mannosyl-glycoprotein 2-beta-N-acetylglucosaminyltransferase	mRNA
chr1	207767464	207767534	intron	CD46	CD46 molecule	mRNA
chr5	17222728	17222802	intron	BASP1	brain abundant, membrane attached signal protein 1	mRNA
chr6	16263090	16263149	intron	GMPR	guanosine monophosphate reductase	mRNA
chr17	5431929	5431997	intron	RPAIN	RPA interacting protein	mRNA
chr14	96533445	96533521	intron	PAPOLA	poly(A) polymerase alpha	mRNA
chr14	64523597	64523673	intron	ZBTB1	zinc finger and BTB domain containing 1	mRNA
chr7	98304469	98304529	intron	BAIAP2L1	BAR/IMD domain containing adaptor protein 2 like 1	mRNA
chr8	140902739	140902792	intron	PTK2	protein tyrosine kinase 2	mRNA
chr22	49573086	49573136	intron	C22orf34	chromosome 22 open reading frame 34	mRNA
chr14	55283842	55283911	intron	FBXO34	F-box protein 34	mRNA
chr17	46938853	46938923	intron	GOSR2	golgi SNAP receptor complex member 2	mRNA
chr8	79956616	79956675	intron	MRPS28	mitochondrial ribosomal protein S28	mRNA
chr15	80444536	80444591	intron	ARNT2	aryl hydrocarbon receptor nuclear translocator 2	mRNA
chr12	50190182	50190232	intron	LIMA1	LIM domain and actin binding 1	mRNA
chr2	121479747	121479785	intron	CLASP1	cytoplasmic linker associated protein 1	mRNA
chr13	106558199	106558231	intron	ARGLU1	arginine and glutamate rich 1	mRNA
chr19	47721197	47721266	intron	EHD2	EH domain containing 2	mRNA
chr9	76175248	76175284	intron	PCSK5	proprotein convertase subtilisin/kexin type 5	mRNA
chrX	136879191	136879273	intron	RBMX	RNA binding motif protein X-linked	mRNA
chr14	21263058	21263128	intron	HNRNPC	heterogeneous nuclear ribonucleoprotein C	mRNA
chr1	65630324	65630354	intron	LEPR	leptin receptor	mRNA
chr20	5588878	5588925	intron	GPCPD1	glycerophosphocholine phosphodiesterase 1	mRNA
chr14	21262277	21262335	intron	HNRNPC	heterogeneous nuclear ribonucleoprotein C	mRNA
chr11	82914102	82914154	intron	DDIAS	DNA damage induced apoptosis suppressor	mRNA
chr12	110049571	110049636	intron	C12orf76	chromosome 12 open reading frame 76	mRNA
chr6	151948765	151948842	intron	ESR1	estrogen receptor 1	mRNA
chr2	233702907	233702953	intron	UGT1A8	UDP glucuronosyltransferase 1 family, polypeptide A8	mRNA
chr3	42627549	42627601	intron	NKTR	natural killer cell triggering receptor	mRNA
chr14	67938739	67938821	intron	RAD51B	RAD51 paralog B	mRNA
chr19	6717539	6717624	intron	C3	complement C3	mRNA
chr7	74883112	74883166	intron	STAG3L2	stromal antigen 3-like 2	mRNA
chr22	45929771	45929843	intron	WNT7B	Wnt family member 7B	mRNA
chr8	115552248	115552285	intron	TRPS1	transcriptional repressor GATA binding 1	mRNA
chr1	207769520	207769610	intron	CD46		mRNA
chr18	3600204	3600249	intron	DLGAP1	DLG associated protein 1	mRNA
chr17	50751847	50751913	intron	LUC7L3	LUC7 like 3 pre-mRNA splicing factor	mRNA
chr2	169623034	169623091	intron	PPIG	peptidylprolyl isomerase G	mRNA
chr1	103554374	103554446	intron	RNPC3	RNA binding region (RNP1, RRM) containing 3	mRNA
chr17	47974808	47974861	intron	CDK5RAP3	CDK5 regulatory subunit associated protein 3	mRNA
chr7	6700343	6700390	intron	ZNF12	zinc finger protein 12	mRNA
chr1	70223407	70223501	intron	SRSF11	serine and arginine rich splicing factor 11	mRNA
chr8	42534637	42534670	intron	SLC20A2	solute carrier family 20 member 2	mRNA
chr7	23530197	23530264	intron	TRA2A	transformer 2 alpha homolog	mRNA
chr7	44005215	44005301	intron	SPDYE1	speedy/RINGO cell cycle regulator family member E1	mRNA
chr8	118285059	118285108	intron	SAMD12	sterile alpha motif domain containing 12	mRNA
chr8	118247959	118248058	intron	SAMD12	sterile alpha motif domain containing 12	mRNA
chr15	45598265	45598363	intron	BLOC1S6	biogenesis of lysosomal organelles complex 1 subunit 6	mRNA

chr16	85706687	85706764	intron	C16orf74	chromosome 16 open reading frame 74	mRNA
chr16	81626446	81626548	intron	CMIP	c-Maf inducing protein	mRNA
chr1	110344017	110344093	intron	RBM15	RNA binding motif protein 15	mRNA
chr2	227528930	227529021	intron	AGFG1	ArfGAP with FG repeats 1	mRNA
chr22	46382095	46382198	intron	CELSR1	cadherin EGF LAG seven-pass G-type receptor 1	mRNA
chr2	197419895	197419927	intron	SF3B1	splicing factor 3b subunit 1	mRNA
chr13	110899432	110899486	intron	ANKRD10	ankyrin repeat domain 10	mRNA
chr1	1737226	1737315	intron	SLC35E2A	solute carrier family 35 member E2A	mRNA
chr17	59839295	59839388	intron	VMP1	vacuole membrane protein 1	mRNA
chr11	62530347	62530410	intron	AHNAK	AHNAK nucleoprotein	mRNA
chr1	234407990	234408035	intron	TARBP1	TAR (HIV-1) RNA binding protein 1	mRNA
chr19	47719983	47720037	intron	EHD2	EH domain containing 2	mRNA
chr8	118292322	118292436	intron	SAMD12	sterile alpha motif domain containing 12	mRNA
chr8	118279150	118279214	intron	SAMD12	sterile alpha motif domain containing 12	mRNA
chr16	87738382	87738462	intron	KLHDC4	kelch domain containing 4	mRNA
chr17	59839571	59839672	intron	VMP1	vacuole membrane protein 1	mRNA
chr17	82146886	82146962	intron	CCDC57	coiled-coil domain containing 57	mRNA
chr12	22668388	22668435	intron	ETNK1	ethanolamine kinase 1	mRNA
chr2	61536942	61537039	intron	XPO1	exportin 1	mRNA
chr20	34073179	34073234	intron	RALY	RALY heterogeneous nuclear ribonucleoprotein	mRNA
chr8	118038993	118039113	intron	EXT1	exostosin glycosyltransferase 1	mRNA
chr20	36137386	36137423	intron	EPB41L1	erythrocyte membrane protein band 4.1 like 1	mRNA
chr2	241507	241559	intron	SH3YL1	SH3 and SYLF domain containing 1	mRNA
chr11	65085812	65085919	intron	ZFPL1	zinc finger protein like 1	mRNA
chr11	62529113	62529234	intron	AHNAK	AHNAK nucleoprotein	mRNA
chr1	1052808	1052914	intron	AGRN	agrin	mRNA
chr19	36213945	36213984	intron	ZNF565	zinc finger protein 565	mRNA
chr18	37075114	37075177	intron	KIAA1328	KIAA1328	mRNA
chr20	49694844	49694935	intron	B4GALT5	UDP-Gal:betaGlcNAc beta 1,4- galactosyltransferase, polypeptide 5	mRNA
chr19	36213986	36214110	intron	ZNF565	zinc finger protein 565	mRNA
chr6	142934904	142935012	intron	HIVEP2	HIVEP zinc finger 2	mRNA
chr20	34072860	34072924	intron	RALY	RALY heterogeneous nuclear ribonucleoprotein	mRNA
chr22	16980486	16980533	intron	GAB4	GRB2 associated binding protein family member 4	mRNA
chr19	49102838	49102965	intron	SNRNP70	small nuclear ribonucleoprotein U1 subunit 70	mRNA
chr17	17821094	17821161	intron	SREBF1	sterol regulatory element binding transcription factor 1	mRNA
chr3	122014951	122014986	intron	ILDR1	immunoglobulin like domain containing receptor 1	mRNA
chr11	35194673	35194718	intron	CD44	CD44 molecule	mRNA
chr12	52237862	52237915	intron	KRT7	keratin 7	mRNA
chr12	76068630	76068700	intron	NAP1L1	nucleosome assembly protein 1 like 1	mRNA
chr9	33347717	33347807	intron	NFX1	nuclear transcription factor, X-box binding 1	mRNA
chr19	15331024	15331091	intron	BRD4	bromodomain containing 4	mRNA
chr7	23529570	23529613	intron	TRA2A	transformer 2 alpha homolog	mRNA
chr20	44209672	44209756	intron	OSER1	oxidative stress responsive serine rich 1	mRNA
chrX	120438720	120438854	intron	LAMP2	lysosomal associated membrane protein 2	mRNA
chr19	40687906	40688041	intron	NUMBL	NUMB like endocytic adaptor protein	mRNA
chr19	47721068	47721130	intron	EHD2	EH domain containing 2	mRNA
chr22	38488801	38488918	intron	DDX17	DEAD-box helicase 17	mRNA
chr22	29291001	29291079	intron	EWSR1	EWS RNA binding protein 1	mRNA
chr5	32441433	32441499	intron	ZFR	zinc finger RNA binding protein	mRNA
chr1	43596749	43596889	intron	PTPRF	protein tyrosine phosphatase receptor type F	mRNA
chr20	44498607	44498748	intron	SERINC3	serine incorporator 3	mRNA
chr11	33140342	33140436	intron	CSTF3	cleavage stimulation factor subunit 3	mRNA
chr17	59837965	59838108	intron	VMP1	vacuole membrane protein 1	mRNA
chr1	20775523	20775667	intron	HP1BP3		mRNA
chr21	38808461	38808601	intron	ETS2	ETS proto-oncogene 2	mRNA
chr21	33530392	33530431	intron	GART	phosphoribosylglycinamide formyltransferase, phosphoribosylglycinamide synthe	mRNA
chr19	40688178	40688245	intron	NUMBL	NUMB like endocytic adaptor protein	mRNA
chr1	20774287	20774433	intron	HP1BP3	heterochromatin protein 1 binding protein 3	mRNA
chrX	85969516	85969564	intron	CHM	CHM Rab escort protein	mRNA
chr5	171406571	171406641	intron	NPM1	nucleophosmin 1	mRNA
chr11	62531254	62531364	intron	AHNAK	AHNAK nucleoprotein	mRNA

chr1	42850072	42850211	intron	ZNF691	zinc finger protein 691	mRNA
chr18	2686661	2686767	intron	SMCHD1	structural maintenance of chromosomes flexible hinge domain containing 1	mRNA
chr8	118039936	118040045	intron	EXT1	exostosin glycosyltransferase 1	mRNA
chr8	51839122	51839170	intron	PCMTD1	protein-L-isoaspartate (D-aspartate) O-methyltransferase domain containing 1	mRNA
chr15	100883033	100883102	intron	ALDH1A3	aldehyde dehydrogenase 1 family member A3	mRNA
chr1	1391160	1391243	intron	CCNL2	cyclin L2	mRNA
chr8	117938884	117939033	intron	EXT1	exostosin glycosyltransferase 1	mRNA
chr1	20774611	20774699	intron	HP1BP3	heterochromatin protein 1 binding protein 3	mRNA
chr8	118288852	118288965	intron	SAMD12	sterile alpha motif domain containing 12	mRNA
chr9	28533	28679	intron	WASHC1	WASH complex subunit 1	mRNA
chr2	85543489	85543545	intron	MAT2A	methionine adenosyltransferase 2A	mRNA
chr17	17820892	17820943	intron	SREBF1	sterol regulatory element binding transcription factor 1	mRNA
chrX	71547730	71547789	intron	OGT	O-linked N-acetylglucosamine (GlcNAc) transferase	mRNA
chr20	36093175	36093263	intron	EPB41L1	erythrocyte membrane protein band 4.1 like 1	mRNA
chr8	23568147	23568285	intron	SLC25A37	solute carrier family 25 member 37	mRNA
chr11	64306472	64306576	intron	ESRRA	estrogen related receptor alpha	mRNA
chr18	3455220	3455281	intron	TGIF1	TGFB induced factor homeobox 1	mRNA
chr8	117957785	117957885	intron	EXT1	exostosin glycosyltransferase 1	mRNA
chr6	169649755	169649886	intron	WDR27	WD repeat domain 27	mRNA
chr15	85384870	85385042	intron	AKAP13	A-kinase anchoring protein 13	mRNA
chr19	36214134	36214146	intron	ZNF565	zinc finger protein 565	mRNA
chr19	36214283	36214314	intron	ZNF565	zinc finger protein 565	mRNA
chr8	118012788	118012887	intron	EXT1	exostosin glycosyltransferase 1	mRNA
chr11	62528909	62529093	intron	AHNAK	AHNAK nucleoprotein	mRNA
chr19	47720444	47720632	intron	EHD2	EH domain containing 2	mRNA
chr6	47005508	47005697	intron	ADGRF1	adhesion G protein-coupled receptor F1	mRNA
chr19	40688692	40688774	intron	NUMBL	NUMB like endocytic adaptor protein	mRNA
chr19	40688775	40688882	intron	NUMBL	NUMB like endocytic adaptor protein	mRNA
chr11	70371325	70371521	intron	PPFIA1	PTPRF interacting protein alpha 1	mRNA
chr8	118078847	118078956	intron	EXT1	exostosin glycosyltransferase 1	mRNA
chr7	47352237	47352366	intron	TNS3	tensin 3	mRNA
chr11	62517725	62517927	intron	AHNAK	AHNAK nucleoprotein	mRNA
chr11	1745744	1745847	intron	IFITM10	interferon induced transmembrane protein 10	mRNA
chr5	882176	882268	intron	BRD9	bromodomain containing 9	mRNA
chr12	54285911	54285967	intron	HNRNPA1	heterogeneous nuclear ribonucleoprotein A1	mRNA
chr19	36215292	36215475	intron	ZNF146	zinc finger protein 146	mRNA
chr7	44005005	44005167	intron	SPDYE1	speedy/RINGO cell cycle regulator family member E1	mRNA
chr16	87738579	87738663	intron	KLHDC4	kelch domain containing 4	mRNA
chrX	151973532	151973751	intron	GABRE	gamma-aminobutyric acid type A receptor epsilon subunit	mRNA
chr20	44498755	44498824	intron	SERINC3	serine incorporator 3	mRNA
chr20	44498825	44498884	intron	SERINC3	serine incorporator 3	mRNA
chr20	44498886	44498989	intron	SERINC3	serine incorporator 3	mRNA
chr8	118296799	118296876	intron	SAMD12	sterile alpha motif domain containing 12	mRNA
chr13	106559090	106559203	intron	ARGLU1	arginine and glutamate rich 1	mRNA
chr8	117979481	117979527	intron	EXT1	exostosin glycosyltransferase 1	mRNA
chr11	62529507	62529650	intron	AHNAK	AHNAK nucleoprotein	mRNA
chr7	55067991	55068033	intron	EGFR	epidermal growth factor receptor	mRNA
chr7	47351882	47351972	intron	TNS3	tensin 3	mRNA
chr20	45479013	45479225	intron	WFDC2	WAP four-disulfide core domain 2	mRNA
chr18	3594772	3594912	intron	DLGAP1	DLG associated protein 1	mRNA
chr11	62517929	62518198	intron	AHNAK	AHNAK nucleoprotein	mRNA
chr16	87741814	87742004	intron	KLHDC4	kelch domain containing 4	mRNA
chr5	139284162	139284241	intron	MATR3	matrin 3	mRNA
chr18	35246782	35246835	intron	ZNF397	zinc finger protein 397	mRNA
chr13	113215029	113215120	intron	CUL4A	cullin 4A	mRNA
chr8	117989580	117989853	intron	EXT1	exostosin glycosyltransferase 1	mRNA
chr3	48425838	48425958	intron	PLXNB1	plexin B1	mRNA
chr11	71484619	71484925	intron	NADSYN1	NAD synthetase 1	mRNA
chr17	59838854	59839031	intron	VMP1	vacuole membrane protein 1	mRNA
chr20	44499259	44499317	intron	SERINC3	serine incorporator 3	mRNA
chr20	44498996	44499119	intron	SERINC3	serine incorporator 3	mRNA

chr11	1746050	1746144	intron	IFITM10	interferon induced transmembrane protein 10	mRNA
chr11	62532524	62532729	intron	AHNAK	AHNAK nucleoprotein	mRNA
chr20	36094149	36094211	intron	EPB41L1	erythrocyte membrane protein band 4.1 like 1	mRNA
chr20	36094302	36094449	intron	EPB41L1	erythrocyte membrane protein band 4.1 like 1	mRNA
chr16	31187071	31187250	intron	FUS	FUS RNA binding protein	mRNA
chr16	31187252	31187415	intron	FUS	FUS RNA binding protein	mRNA
chr11	62516716	62516755	intron	AHNAK	AHNAK nucleoprotein	mRNA
chr11	62516767	62517112	intron	AHNAK	AHNAK nucleoprotein	mRNA
chr19	47720642	47720770	intron	EHD2	EH domain containing 2	mRNA
chr1	12582607	12582844	intron	DHRS3	dehydrogenase/reductase 3	mRNA
chr11	70408269	70408361	intron	CTTN	cortactin	mRNA
chr11	62516343	62516494	intron	AHNAK	AHNAK nucleoprotein	mRNA
chr8	118042615	118042857	intron	EXT1	exostosin glycosyltransferase 1	mRNA
chr8	118356966	118357075	intron	SAMD12	sterile alpha motif domain containing 12	mRNA
chr9	134339817	134340099	intron	RXRA	retinoid X receptor, alpha	mRNA
chr1	43596959	43597330	intron	PTPRF	protein tyrosine phosphatase receptor type F	mRNA
chr11	70421914	70422157	intron	CTTN	cortactin	mRNA
chr11	70422158	70422290	intron	CTTN	cortactin	mRNA
chr11	70422291	70422577	intron	CTTN	cortactin	mRNA
chr5	882730	883050	intron	BRD9	bromodomain containing 9	mRNA
chr1	70231445	70231545	intron	SRSF11	serine and arginine rich splicing factor 1	mRNA
chr21	37059189	37059232	intron	PIGP	phosphatidylinositol glycan anchor biosynthesis class P	mRNA
chr17	59840952	59841166	three_prime_	VMP1	vacuole membrane protein 1	mRNA
chr2	202206714	202206802	three_prime_	SUMO1	small ubiquitin like modifier 1	mRNA
chr13	48261307	48261482	three_prime_	ITM2B	integral membrane protein 2B	mRNA
chr13	48261528	48261637	three_prime_	ITM2B	integral membrane protein 2B	mRNA
chr3	179398456	179398465	three_prime_	GNB4	G protein subunit beta 4	mRNA
chr9	132594181	132594198	three_prime_	DDX31	DEAD-box helicase 31	mRNA
chr19	48705316	48705336	three_prime_	FUT2	fucosyltransferase 2 (secretor status included)	mRNA
chr1	114705771	114705791	three_prime_	NRAS	neuroblastoma RAS viral (v-ras) oncogene homolog	mRNA
chr4	113761273	113761292	three_prime_	CAMK2D	calcium/calmodulin dependent protein kinase II delta	mRNA
chr1	9730463	9730483	three_prime_	CLSTN1	calsyntenin 1	mRNA
chr2	174337814	174337833	three_prime_	SP9	Sp9 transcription factor	mRNA
chr1	205303126	205303147	three_prime_	NUAK2	NUAK family kinase 2	mRNA
chr11	61581070	61581090	three_prime_	SYT7	synaptotagmin 7	mRNA
chr3	179397901	179397922	three_prime_	GNB4	G protein subunit beta 4	mRNA
chrX	155260403	155260424	three_prime_	RAB39B	RAB39B, member RAS oncogene family	mRNA
chr19	863413	863435	three_prime_	CFD	complement factor D	mRNA
chr1	42420316	42420339	three_prime_	RIMKLA	ribosomal modification protein rimK like family member A	mRNA
chr13	48710751	48710773	three_prime_	CYSLTR2	cysteinyl leukotriene receptor 2	mRNA
chr21	45515629	45515652	three_prime_	SLC19A1	solute carrier family 19 member 1	mRNA
chr6	55755246	55755266	three_prime_	BMP5	bone morphogenetic protein 5	mRNA
chr2	96330068	96330092	three_prime_	ITPRIPL1	ITPRIP like 1	mRNA
chr1	65638167	65638191	three_prime_	LEPR	leptin receptor	mRNA
chr10	118304470	118304491	three_prime_	FAM204A	family with sequence similarity 204 member A	mRNA
chr3	196049311	196049335	three_prime_	TFRC	transferrin receptor	mRNA
chr4	40157068	40157092	three_prime_	N4BP2	NEDD4 binding protein 2	mRNA
chr9	6506525	6506550	three_prime_	UHRF2	ubiquitin like with PHD and ring finger domains 2	mRNA
chr4	39477348	39477373	three_prime_	LIAS	lipoic acid synthetase	mRNA
chr15	20532500	20532526	three_prime_	GOLGA6L6	golgin A6 family-like 6	mRNA
chr16	28536527	28536553	three_prime_	NUPR1	nuclear protein, transcriptional regulator, 1	mRNA
chr10	112548435	112548461	three_prime_	VTI1A	vesicle transport through interaction with t-SNAREs 1A	mRNA
chr21	37612709	37612736	three_prime_	KCNJ6	potassium channel, inwardly rectifying subfamily J, member 6	mRNA
chr5	1222584	1222611	three_prime_	SLC6A19	solute carrier family 6 member 19	mRNA
chr13	25163577	25163597	three_prime_	AMER2	APC membrane recruitment protein 2	mRNA
chrX	3608054	3608081	three_prime_	PRKX	protein kinase, X-linked	mRNA
chr4	127723789	127723814	three_prime_	INTU	inturned planar cell polarity protein	mRNA
chr16	4804949	4804977	three_prime_	GLYR1	glyoxylate reductase 1 homolog	mRNA
chrX	55181518	55181547	three_prime_	MTRNR2L10	MT-RNR2-like 10	mRNA
chr13	49492965	49492994	three_prime_	SETDB2	SET domain bifurcated histone lysine methyltransferase 2	mRNA
chr3	124905602	124905631	three_prime_	MUC13	mucin 13, cell surface associated	mRNA

chr9	33253853	33253881	three_prime_	BAG1	BCL2 associated athanogene 1	mRNA
chr6	131894448	131894478	three_prime_	ENPP1	ectonucleotide pyrophosphatase/phosphodiesterase 1	mRNA
chr13	27256537	27256568	three_prime_	RPL21	ribosomal protein L21	mRNA
chr6	122443563	122443592	three_prime_	SERINC1	serine incorporator 1	mRNA
chr13	29512648	29512679	three_prime_	SLC7A1	solute carrier family 7 member 1	mRNA
chr11	8687216	8687244	three_prime_	RPL27A	ribosomal protein L27a	mRNA
chr8	55524598	55524629	three_prime_	XKR4	XK, Kell blood group complex subunit-related family, member 4	mRNA
chr9	34368301	34368330	three_prime_	MYORG	myogenesis regulating glycosidase	mRNA
chr12	55995423	55995452	three_prime_	RAB5B	RAB5B, member RAS oncogene family	mRNA
chr7	38724748	38724779	three_prime_	VPS41	VPS41 subunit of HOPS complex	mRNA
chr11	108368220	108368251	three_prime_	ATM	ATM serine/threonine kinase,	mRNA
chr17	37611993	37612022	three_prime_	DDX52	DExD-box helicase 52	mRNA
chr2	60797730	60797762	three_prime_	PAPOLG	poly(A) polymerase gamma	mRNA
chr7	121350032	121350065	three_prime_	FAM3C	family with sequence similarity 3 member C	mRNA
chr13	51763565	51763596	three_prime_	WDFY2	WD repeat and FYVE domain containing 2	mRNA
chr18	32070211	32070238	three_prime_	RNF125	ring finger protein 125	mRNA
chr1	166913934	166913966	three_prime_	ILDR2	immunoglobulin like domain containing receptor 2	mRNA
chr3	191267693	191267726	three_prime_	UTS2B	Urotensin 2B	mRNA
chr19	6663293	6663321	three_prime_	TNFSF14	TNF superfamily member 14	mRNA
chr11	2131835	2131859	three_prime_	IGF2	insulin like growth factor 2	mRNA
chr12	121651133	121651164	three_prime_	MORN3	MORN repeat containing 3	mRNA
chr1	160029538	160029571	three_prime_	PIGM	phosphatidylinositol glycan anchor biosynthesis, class M	mRNA
chr12	121185904	121185932	three_prime_	P2RX7	purinergic receptor P2X 7	mRNA
chr5	14696868	14696902	three_prime_	OTULIN	OTU deubiquitinase with linear linkage specificity	mRNA
chr11	74978533	74978567	three_prime_	SPCS2	signal peptidase complex subunit 2	mRNA
chr12	49273904	49273938	three_prime_	TUBA1C	tubulin alpha 1c	mRNA
chr14	90526103	90526134	three_prime_	TTC7B	tetratricopeptide repeat domain 7B	mRNA
chr17	82489810	82489833	three_prime_	NARF	nuclear prelamin A recognition factor	mRNA
chr13	27256536	27256568	three_prime_	RPL21	ribosomal protein L21	mRNA
chr18	46669886	46669909	three_prime_	ST8SIA5	ST8 alpha-N-acetyl-neuraminide alpha-2,8-sialyltransferase 5	mRNA
chr2	241352048	241352083	three_prime_	SEPTIN2	septin 2	mRNA
chr1	150996217	150996252	three_prime_	DHCR24	24-dehydrocholesterol reductase	mRNA
chr1	182383683	182383713	three_prime_	GLUL	glutamate-ammonia ligase	mRNA
chr1	248918944	248918975	three_prime_	PGBD2	piggyBac transposable element derived 2	mRNA
chr9	88475726	88475761	three_prime_	SPIN1	spindlin 1	mRNA
chr11	101998391	101998422	three_prime_	CEP126	centrosomal protein 126	mRNA
chr11	74978583	74978617	three_prime_	SPCS2	signal peptidase complex subunit 2	mRNA
chr12	121020302	121020334	three_prime_	OASL	2'-5'-oligoadenylate synthetase like	mRNA
chr20	3805940	3805975	three_prime_	CDC25B	cell division cycle 25B	mRNA
chr1	160216297	160216332	three_prime_	DCAF8	DDB1 and CUL4 associated factor 8	mRNA
chr8	143655073	143655108	three_prime_	ZNF623	zinc finger protein 623	mRNA
chr20	49096726	49096762	three_prime_	CSE1L	chromosome segregation 1 like	mRNA
chr6	100858643	100858673	three_prime_	ASCC3	activating signal cointegrator 1 complex subunit 3	mRNA
chr1	226226432	226226463	three_prime_	MIXL1	Mix paired-like homeobox	mRNA
chr4	69723810	69723846	three_prime_	SULT1B1	sulfotransferase family 1B member 1	mRNA
chr5	176379334	176379370	three_prime_	ARL10	ADP-ribosylation factor-like 10	mRNA
chr19	5891423	5891455	three_prime_	NDUFA11	NADH:ubiquinone oxidoreductase subunit A11	mRNA
chr2	70279584	70279616	three_prime_	PCYOX1	prenylcysteine oxidase 1	mRNA
chr7	40096640	40096659	three_prime_	CDK13	cyclin dependent kinase 13	mRNA
chrX	107064741	107064770	three_prime_	RBM41	RNA binding motif protein 41	mRNA
chr19	11146895	11146931	three_prime_	SPC24	SPC24 component of NDC80 kinetochore complex	mRNA
chr3	49533725	49533761	three_prime_	DAG1	dystroglycan 1	mRNA
chr5	144478901	144478938	three_prime_	KCTD16	potassium channel tetramerization domain containing 16	mRNA
chr19	46467325	46467361	three_prime_	PNMA8A	PNMA family member 8A	mRNA
chr3	46669200	46669234	three_prime_	ALS2CL	ALS2 C-terminal like	mRNA
chr16	75474445	75474479	three_prime_	CHST6	carbohydrate sulfotransferase 6	mRNA
chr19	8509850	8509887	three_prime_	ZNF414	zinc finger protein 414	mRNA
chr20	37983142	37983179	three_prime_	TTI1	TELO2 interacting protein 1	mRNA
chr14	102498851	102498881	three_prime_	TECPR2	tectonin beta-propeller repeat containing 2	mRNA
chr6	89163800	89163837	three_prime_	PM20D2	peptidase M20 domain containing 2	mRNA
chr19	48600823	48600853	three_prime_	FAM83E	family with sequence similarity 83, member E	mRNA

chr3	100180318	100180353	three_prime_	CMSS1	cms1 ribosomal small subunit homolog	mRNA
chr1	65231288	65231323	three_prime_	AK4	adenylate kinase 4	mRNA
chr9	136207332	136207371	three_prime_	QSOX2	quiescin sulfhydryl oxidase 2	mRNA
chr5	137752856	137752893	three_prime_	HNRNPA0	heterogeneous nuclear ribonucleoprotein A0	mRNA
chr6	142444751	142444790	three_prime_	ADGRG6	adhesion G protein-coupled receptor G6	mRNA
chr2	88110878	88110910	three_prime_	SMYD1	SET and MYND domain containing 1	mRNA
chr1	92398298	92398332	three_prime_	RPAP2	RNA polymerase II associated protein 2	mRNA
chr22	46362665	46362704	three_prime_	CELSR1	cadherin EGF LAG seven-pass G-type receptor 1	mRNA
chrX	24076799	24076839	three_prime_	EIF2S3	eukaryotic translation initiation factor 2 subunit gamma	mRNA
chr5	116446245	116446283	three_prime_	SEMA6A	semaphorin 6A	mRNA
chr3	49415495	49415520	three_prime_	TCTA	T-cell leukemia translocation altered	mRNA
chr12	54546173	54546197	three_prime_	NCKAP1L	NCK associated protein 1 like	mRNA
chr10	119167785	119167821	three_prime_	PRDX3	peroxiredoxin 3	mRNA
chr11	111295163	111295201	three_prime_	COLCA1	colorectal cancer associated 1	mRNA
chr5	1050407	1050448	three_prime_	SLC12A7	solute carrier family 12 member 7	mRNA
chr18	23531789	23531823	three_prime_	RMC1	regulator of MON1-CCZ1	mRNA
chr7	40127229	40127264	three_prime_	MPLKIP	M-phase specific PLK1 interacting protein	mRNA
chr7	93101045	93101079	three_prime_	SAMD9	sterile alpha motif domain containing 9	mRNA
chr7	152647040	152647080	three_prime_	XRCC2	X-ray repair complementing defective repair in Chinese hamster cells 2	mRNA
chr3	12734086	12734127	three_prime_	TMEM40	transmembrane protein 40	mRNA
chr10	811232	811265	three_prime_	LARP4B	La ribonucleoprotein 4B	mRNA
chr6	34279776	34279818	three_prime_	NUDT3	nudix (nucleoside diphosphate linked moiety X)-type motif 3	mRNA
chr6	89162542	89162584	three_prime_	PM20D2	peptidase M20 domain containing 2	mRNA
chr5	65669615	65669655	three_prime_	SGTB	small glutamine rich tetratricopeptide repeat containing beta	mRNA
chr2	11181801	11181832	three_prime_	ROCK2	Rho associated coiled-coil containing protein kinase 2	mRNA
chr21	38301939	38301970	three_prime_	KCNJ15	potassium inwardly rectifying channel subfamily J member 15	mRNA
chr5	77431335	77431374	three_prime_	WDR41	WD repeat domain 41	mRNA
chr7	152647457	152647499	three_prime_	XRCC2	X-ray repair complementing defective repair in Chinese hamster cells 2	mRNA
chr12	10159287	10159326	three_prime_	OLR1	oxidized low density lipoprotein receptor 1	mRNA
chr17	28908490	28908528	three_prime_	PHF12	PHD finger protein 12	mRNA
chr15	40662759	40662801	three_prime_	KNL1	kinetochore scaffold 1	mRNA
chr20	3926915	3926946	three_prime_	PANK2	pantothenate kinase 2	mRNA
chr2	241351991	241352033	three_prime_	SEPTIN2	septin 2	mRNA
chr17	82239443	82239483	three_prime_	SLC16A3	solute carrier family 16 member 3	mRNA
chr11	82729962	82729999	three_prime_	FAM181B	family with sequence similarity 181, member B	mRNA
chr10	73498442	73498478	three_prime_	USP54	ubiquitin specific peptidase 54	mRNA
chr20	13820509	13820545	three_prime_	NDUFAF5	NADH:ubiquinone oxidoreductase complex assembly factor 5	mRNA
chr9	19372740	19372783	three_prime_	DENND4C	DENN domain containing 4C	mRNA
chr1	179918715	179918759	three_prime_	TOR1AIP1	torsin 1A interacting protein 1	mRNA
chr1	32042769	32042813	three_prime_	KHDRBS1	KH RNA binding domain containing, signal transduction associated 1	mRNA
chr17	1706573	1706617	three_prime_	TLCD2	TLC domain containing 2	mRNA
chr5	177065869	177065913	three_prime_	ZNF346	zinc finger protein 346	mRNA
chr6	106569242	106569284	three_prime_	CRYBG1	crystallin beta-gamma domain containing 1	mRNA
chr1	25861979	25862022	three_prime_	PAQR7	progestin and adipoQ receptor family member VII	mRNA
chr3	119669577	119669621	three_prime_	COX17	cytochrome c oxidase copper chaperone COX17	mRNA
chrX	18239825	18239865	three_prime_	SCML2	Scm polycomb group protein like 2	mRNA
chr19	11783066	11783103	three_prime_	ZNF441	zinc finger protein 441	mRNA
chr19	34507186	34507229	three_prime_	WTIP	WT1 interacting protein	mRNA
chr1	220058686	220058727	three_prime_	BPNT1	3'(2'), 5'-bisphosphate nucleotidase 1	mRNA
chr1	15568691	15568733	three_prime_	DNAJC16	DnaJ heat shock protein family (Hsp40) member C16	mRNA
chr7	13892750	13892794	three_prime_	ETV1	ETS variant transcription factor 1	mRNA
chr6	115939663	115939708	three_prime_	FRK	fyn-related Src family tyrosine kinase	mRNA
chr8	22005656	22005701	three_prime_	XPO7	exportin 7	mRNA
chr19	52293131	52293169	three_prime_	ZNF766	zinc finger protein 766	mRNA
chr17	4143341	4143377	three_prime_	CYB5D2	cytochrome b5 domain containing 2	mRNA
chr2	27155709	27155745	three_prime_	TCF23	transcription factor 23	mRNA
chr19	5206283	5206329	three_prime_	PTPRS	protein tyrosine phosphatase receptor type S	mRNA
chr16	68833852	68833897	three_prime_	CDH1	cadherin 1	mRNA
chr1	39004450	39004495	three_prime_	AKIRIN1	akirin 1	mRNA
chr2	230819798	230819836	three_prime_	CAB39	calcium binding protein 39	mRNA
chr3	101225399	101225435	three_prime_	IMPG2	interphotoreceptor matrix proteoglycan 2	mRNA

chr19	42227864	42227909	three_prime_	ZNF526	zinc finger protein 526	mRNA
chrX	16763105	16763151	three_prime_	SYAP1	synapse associated protein 1	mRNA
chr9	83938920	83938963	three_prime_	C9orf64	chromosome 9 open reading frame 64	mRNA
chr18	74585809	74585840	three_prime_	CNDP1	carnosine dipeptidase 1	mRNA
chr19	55230266	55230312	three_prime_	PPP6R1	protein phosphatase 6 regulatory subunit 1	mRNA
chr6	122443503	122443550	three_prime_	SERINC1	serine incorporator 1	mRNA
chr3	41239941	41239986	three_prime_	CTNNB1	catenin beta 1	mRNA
chr5	177331689	177331736	three_prime_	LMAN2	lectin, mannose binding 2	mRNA
chr1	156465657	156465698	three_prime_	MEF2D	myocyte enhancer factor 2D	mRNA
chr15	98963173	98963220	three_prime_	IGF1R	insulin like growth factor 1 receptor	mRNA
chr3	100364704	100364751	three_prime_	TOMM70A	translocase of outer mitochondrial membrane 70 homolog A (S. cerevisiae)	mRNA
chr3	188879816	188879858	three_prime_	LPP	LIM domain containing preferred translocation partner in lipoma	mRNA
chr11	74977220	74977267	three_prime_	SPCS2	signal peptidase complex subunit 2	mRNA
chr12	113390317	113390364	three_prime_	PLBD2	phospholipase B domain containing 2	mRNA
chr1	114584776	114584808	three_prime_	DENND2C	DENN domain containing 2C	mRNA
chr5	314621	314669	three_prime_	PDCD6	programmed cell death 6	mRNA
chr1	161071920	161071965	three_prime_	NECTIN4	nectin cell adhesion molecule 4	mRNA
chr11	119118428	119118471	three_prime_	C2CD2L	C2CD2 like	mRNA
chr6	73827914	73827960	three_prime_	CD109	CD109 molecule	mRNA
chr7	6402532	6402580	three_prime_	RAC1	Rac family small GTPase 1	mRNA
chr11	34100012	34100060	three_prime_	CAPRIN1	cell cycle associated protein 1	mRNA
chr14	53949961	53950002	three_prime_	BMP4	bone morphogenetic protein 4	mRNA
chr12	133019474	133019517	three_prime_	ZNF26	zinc finger protein 26	mRNA
chr10	38122607	38122638	three_prime_	ZNF37A	zinc finger protein 37A	mRNA
chr8	61625891	61625937	three_prime_	ASPH	aspartate beta-hydroxylase	mRNA
chr15	52548119	52548157	three_prime_	ARPP19	cAMP regulated phosphoprotein 19	mRNA
chr7	116797014	116797063	three_prime_	MET	MET proto-oncogene, receptor tyrosine kinase	mRNA
chr11	118354501	118354549	three_prime_	CD3G	CD3G molecule	mRNA
chr16	4383339	4383388	three_prime_	VASN	vasorin	mRNA
chr9	21330118	21330162	three_prime_	KLHL9	kelch-like family member 9	mRNA
chr8	125022234	125022280	three_prime_	SQLE	squalene epoxidase	mRNA
chr3	196051058	196051108	three_prime_	TFRC	transferrin receptor	mRNA
chr1	246767365	246767410	three_prime_	SCCPDH	saccharopine dehydrogenase (putative)	mRNA
chr11	65637385	65637417	three_prime_	PCNX3	pecanex 3	mRNA
chr5	157786562	157786610	three_prime_	CLINT1	clathrin interactor 1	mRNA
chr6	158632512	158632563	three_prime_	TMEM181	transmembrane protein 181	mRNA
chr14	77510270	77510321	three_prime_	SPTLC2	serine palmitoyltransferase long chain base subunit 2	mRNA
chr11	33354647	33354685	three_prime_	HIPK3	homeodomain interacting protein kinase 3	mRNA
chr7	94668141	94668192	three_prime_	PEG10	paternally expressed 10	mRNA
chr12	112508815	112508866	three_prime_	PTPN11	protein tyrosine phosphatase non-receptor type 11	mRNA
chr7	16783844	16783895	three_prime_	TSPAN13	tetraspanin 13	mRNA
chr7	66955634	66955677	three_prime_	TMEM248	transmembrane protein 248	mRNA
chr3	48443876	48443927	three_prime_	TMA7	translation machinery associated 7 homolog (S. cerevisiae)	mRNA
chr1	111440983	111441032	three_prime_	WDR77	WD repeat domain 77	mRNA
chr1	235167390	235167433	three_prime_	ARID4B	AT-rich interaction domain 4B	mRNA
chr16	2520097	2520145	three_prime_	ATP6V0C	ATPase H+ transporting V0 subunit c	mRNA
chr14	74736482	74736516	three_prime_	FCF1	FCF1 rRNA-processing protein	mRNA
chr1	156525789	156525818	three_prime_	IQGAP3	IQ motif containing GTPase activating protein 3	mRNA
chr14	70371424	70371476	three_prime_	SYNJ2BP	synaptojanin 2 binding protein	mRNA
chr9	109018914	109018961	three_prime_	TMEM245	transmembrane protein 245	mRNA
chr8	18029111	18029161	three_prime_	PCM1	pericentriolar material 1	mRNA
chr12	120460970	120461008	three_prime_	GATC	glutamyl-tRNA amidotransferase subunit C	mRNA
chr14	55684098	55684150	three_prime_	KTN1	kinectin 1	mRNA
chr7	16783676	16783727	three_prime_	TSPAN13	tetraspanin 13	mRNA
chr14	50905263	50905299	three_prime_	PYGL	glycogen phosphorylase L	mRNA
chr22	45843865	45843918	three_prime_	ATXN10	ataxin 10	mRNA
chr6	36602729	36602782	three_prime_	SRSF3	serine and arginine rich splicing factor 3	mRNA
chr4	105969829	105969882	three_prime_	NPNT	nephronectin	mRNA
chr11	102396443	102396491	three_prime_	TMEM123	transmembrane protein 123	mRNA
chr5	175959867	175959921	three_prime_	THOC3	THO complex 3	mRNA
chr14	52640795	52640849	three_prime_	ERO1A	endoplasmic reticulum oxidoreductase 1 alpha	mRNA

chr3	101822293	101822335	three_prime_	NXPE3	neurexophilin and PC-esterase domain family member 3	mRNA
chr1	32196831	32196871	three_prime_	TXLNA	taxilin alpha	mRNA
chr14	105470559	105470612	three_prime_	MTA1	metastasis associated 1	mRNA
chr9	126697884	126697918	three_prime_	LMX1B	LIM homeobox transcription factor 1 beta	mRNA
chr6	116495813	116495854	three_prime_	TRAPPC3L	trafficking protein particle complex 3 like	mRNA
chr16	19310069	19310105	three_prime_	CLEC19A	C-type lectin domain containing 19A	mRNA
chr11	63758621	63758673	three_prime_	RTN3	reticulon 3	mRNA
chr20	51598218	51598266	three_prime_	ATP9A	ATPase phospholipid transporting 9A	mRNA
chr17	38727157	38727205	three_prime_	MLLT6	MLLT6, PHD finger containing	mRNA
chr19	35267898	35267944	three_prime_	LSR	lipolysis stimulated lipoprotein receptor	mRNA
chr18	673388	673443	three_prime_	TYMS	thymidylate synthetase	mRNA
chr2	26135155	26135193	three_prime_	RAB10	RAB10, member RAS oncogene family	mRNA
chr2	168773287	168773342	three_prime_	CERS6	ceramide synthase 6	mRNA
chr16	19268116	19268171	three_prime_	SYT17	synaptotagmin 17	mRNA
chrX	68042594	68042640	three_prime_	OPHN1	oligophrenin 1	mRNA
chr11	791193	791248	three_prime_	SLC25A22	solute carrier family 25 member 22	mRNA
chr6	122781910	122781946	three_prime_	FABP7	fatty acid binding protein 7	mRNA
chr13	30205606	30205661	three_prime_	KATNAL1	katanin catalytic subunit A1 like 1	mRNA
chrX	46604601	46604632	three_prime_	SLC9A7	solute carrier family 9 member A7	mRNA
chr19	15057691	15057734	three_prime_	CASP14	caspase 14, apoptosis-related cysteine peptidase	mRNA
chr13	42320338	42320388	three_prime_	AKAP11	A kinase (PRKA) anchor protein 11	mRNA
chr5	172952382	172952438	three_prime_	ERGIC1	endoplasmic reticulum-golgi intermediate compartment 1	mRNA
chr17	78357136	78357184	three_prime_	SOCS3	suppressor of cytokine signaling 3	mRNA
chr6	158634269	158634310	three_prime_	TMEM181	transmembrane protein 181	mRNA
chr12	113390839	113390888	three_prime_	PLBD2	phospholipase B domain containing 2	mRNA
chr8	38988354	38988410	three_prime_	HTRA4	HtrA serine peptidase 4	mRNA
chr5	83054654	83054690	three_prime_	TMEM167A	transmembrane protein 167A	mRNA
chr1	43623527	43623584	three_prime_	PTPRF	protein tyrosine phosphatase receptor type F	mRNA
chr1	153959176	153959233	three_prime_	SLC39A1	solute carrier family 39 member 1	mRNA
chr1	202891706	202891763	three_prime_	KLHL12	kelch like family member 12	mRNA
chr6	115938997	115939033	three_prime_	FRK	fyn-related Src family tyrosine kinase	mRNA
chr17	59110374	59110431	three_prime_	SKA2	spindle and kinetochore associated complex subunit 2	mRNA
chr20	49096669	49096724	three_prime_	CSE1L	chromosome segregation 1 like	mRNA
chr17	28327136	28327188	three_prime_	TMEM97	transmembrane protein 97	mRNA
chr1	54638981	54639034	three_prime_	ACOT11	acyl-CoA thioesterase 11	mRNA
chr20	62388441	62388498	three_prime_	RPS21	ribosomal protein S21	mRNA
chr11	118750996	118751053	three_prime_	DDX6	DEAD-box helicase 6	mRNA
chr1	11919679	11919734	three_prime_	KIAA2013	KIAA2013	mRNA
chr3	9806475	9806516	three_prime_	ARPC4	actin related protein 2/3 complex subunit 4	mRNA
chr7	2442107	2442162	three_prime_	CHST12	carbohydrate (chondroitin 4) sulfotransferase 12	mRNA
chr12	32645444	32645489	three_prime_	FGD4	FYVE, RhoGEF and PH domain containing 4	mRNA
chr5	179035019	179035045	three_prime_	ZNF879	zinc finger protein 879	mRNA
chr3	182942706	182942727	three_prime_	DCUN1D1	defective in cullin neddylation 1 domain containing 1	mRNA
chr16	89914005	89914063	three_prime_	MC1R	Multi-pass membrane protein (By similarity)	mRNA
chr1	154979119	154979174	three_prime_	CKS1B	CDC28 protein kinase regulatory subunit 1B	mRNA
chr9	98015556	98015615	three_prime_	ANP32B	acidic (leucine-rich) nuclear phosphoprotein 32 family, member B	mRNA
chr12	53479596	53479655	three_prime_	PCBP2	poly(rC) binding protein 2	mRNA
chr5	141515877	141515910	three_prime_	DIAPH1	diaphanous related formin 1	mRNA
chr3	23980017	23980074	three_prime_	NR1D2	nuclear receptor subfamily 1 group D member 2	mRNA
chr1	112671452	112671511	three_prime_	CAPZA1	capping actin protein of muscle Z-line subunit alpha 1	mRNA
chr17	4898001	4898032	three_prime_	MINK1	misshapen like kinase 1	mRNA
chr12	6238009	6238057	three_prime_	CD9	CD9 molecule	mRNA
chr12	123597683	123597731	three_prime_	TMED2	transmembrane p24 trafficking protein 2	mRNA
chr12	13216629	13216689	three_prime_	EMP1	epithelial membrane protein 1	mRNA
chr8	41624003	41624039	three_prime_	GPAT4	glycerol-3-phosphate acyltransferase 4	mRNA
chr14	21210683	21210743	three_prime_	HNRNPC	heterogeneous nuclear ribonucleoprotein C	mRNA
chr16	13239624	13239661	three_prime_	SHISA9	shisa family member 9	mRNA
chr17	38323042	38323086	three_prime_	MRPL45	mitochondrial ribosomal protein L45	mRNA
chr19	56527272	56527305	three_prime_	ZNF471	zinc finger protein 471	mRNA
chr12	66121421	66121481	three_prime_	LLPH	LLP homolog, long-term synaptic facilitation factor	mRNA
chr19	1778785	1778815	three_prime_	ONECUT3	one cut homeobox 3	mRNA

chr5	6671000	6671060	three_prime_	SRD5A1	steroid 5 alpha-reductase 1	mRNA
chr12	92144041	92144098	three_prime_	BTG1	B-cell translocation gene 1, anti-proliferative	mRNA
chr3	128620228	128620289	three_prime_	RPN1	ribophorin I	mRNA
chr20	32850149	32850209	three_prime_	MAPRE1	microtubule-associated protein, RP/EB family, member 1	mRNA
chr9	131577725	131577756	three_prime_	RAPGEF1	Rap guanine nucleotide exchange factor 1	mRNA
chrX	118792292	118792341	three_prime_	IL13RA1	interleukin 13 receptor subunit alpha 1	mRNA
chr12	93506423	93506467	three_prime_	MRPL42	mitochondrial ribosomal protein L42	mRNA
chr2	65088201	65088260	three_prime_	RAB1A	RAB1A, member RAS oncogene family	mRNA
chr1	225790028	225790090	three_prime_	SRP9	signal recognition particle 9	mRNA
chr17	15539083	15539135	three_prime_	TVP23C	trans-golgi network vesicle protein 23 homolog C	mRNA
chr15	24683006	24683048	three_prime_	NPAP1	nuclear pore associated protein 1	mRNA
chr19	5152862	5152922	three_prime_	KDM4B	lysine demethylase 4B	mRNA
chr4	106043726	106043777	three_prime_	TBCK	TBC1 domain containing kinase	mRNA
chr10	87753780	87753820	three_prime_	ATAD1	ATPase family AAA domain containing 1	mRNA
chr1	154584489	154584544	three_prime_	ADAR	adenosine deaminase RNA specific	mRNA
chr19	44821367	44821414	three_prime_	BCAM	basal cell adhesion molecule (Lutheran blood group)	mRNA
chr11	126202430	126202493	three_prime_	RPUSD4	RNA pseudouridine synthase D4	mRNA
chr1	24471665	24471728	three_prime_	NIPAL3	NIPA like domain containing 3	mRNA
chr12	53188391	53188430	three_prime_	ZNF740	zinc finger protein 740	mRNA
chr1	113975978	113976015	three_prime_	HIPK1	homeodomain interacting protein kinase 1	mRNA
chr5	43706851	43706910	three_prime_	NNT	nicotinamide nucleotide transhydrogenase	mRNA
chr17	74593815	74593868	three_prime_	C17orf77	chromosome 17 open reading frame 77	mRNA
chr7	2437712	2437767	three_prime_	CHST12	carbohydrate (chondroitin 4) sulfotransferase 12	mRNA
chr15	94483494	94483525	three_prime_	MCTP2	multiple C2 and transmembrane domain containing 2	mRNA
chr19	11450904	11450967	three_prime_	PRKCSH	protein kinase C substrate 80K-H	mRNA
chr11	75405603	75405667	three_prime_	RPS3	ribosomal protein S3	mRNA
chr3	122575035	122575098	three_prime_	DTX3L	deltex E3 ubiquitin ligase 3L	mRNA
chr5	97034651	97034713	three_prime_	LNPEP	leucyl and cystinyl aminopeptidase	mRNA
chr4	76160969	76161033	three_prime_	SCARB2	scavenger receptor class B member 2	mRNA
chr10	71302762	71302818	three_prime_	UNC5B	unc-5 netrin receptor B	mRNA
chr5	42721080	42721125	three_prime_	GHR	growth hormone receptor	mRNA
chr2	131147030	131147093	three_prime_	PLEKHB2	pleckstrin homology domain containing B2	mRNA
chr9	88575483	88575547	three_prime_	NXNL2	nucleoredoxin like 2	mRNA
chr17	39997846	39997907	three_prime_	PSMD3	proteasome 26S subunit	mRNA
chr8	127417532	127417595	three_prime_	POU5F1B	POU class 5 homeobox 1B	mRNA
chr1	119912491	119912556	three_prime_	NOTCH2	notch receptor 2	mRNA
chr5	102236582	102236647	three_prime_	SLCO4C1	solute carrier organic anion transporter family, member 4C1	mRNA
chr5	161289773	161289836	three_prime_	GABRB2	gamma-aminobutyric acid type A receptor beta2 subunit	mRNA
chr10	122438857	122438903	three_prime_	PLEKHA1	pleckstrin homology domain containing A1	mRNA
chr2	232857667	232857716	three_prime_	GIGYF2	GRB10 interacting GYF protein 2	mRNA
chr1	159918550	159918616	three_prime_	TAGLN2	transgelin 2	mRNA
chr19	57863571	57863631	three_prime_	ZNF587	zinc finger protein 587	mRNA
chr13	48261714	48261772	three_prime_	ITM2B	integral membrane protein 2B	mRNA
chr13	79553688	79553739	three_prime_	NDFIP2	Nedd4 family interacting protein 2	mRNA
chr17	61945216	61945275	three_prime_	MED13	mediator complex subunit 13	mRNA
chr11	63624888	63624947	three_prime_	ATL3	atlastin GTPase 3	mRNA
chr15	65887994	65888026	three_prime_	RAB11A	RAB11A, member RAS oncogene family	mRNA
chr2	231400978	231401028	three_prime_	B3GNT7	UDP-GlcNAc:betaGal beta-1,3-N-acetylglucosaminyltransferase 7	mRNA
chr8	53968046	53968112	three_prime_	TCEA1	transcription elongation factor A1	mRNA
chr2	200823017	200823084	three_prime_	BZW1	basic leucine zipper and W2 domains 1	mRNA
chr15	98963268	98963314	three_prime_	IGF1R	insulin like growth factor 1 receptor	mRNA
chr15	98963319	98963335	three_prime_	IGF1R	insulin like growth factor 1 receptor	mRNA
chr12	122769834	122769895	three_prime_	DENR	density regulated re-initiation and release factor	mRNA
chr14	62109143	62109199	three_prime_	SYT16	synaptotagmin 16	mRNA
chr1	23795484	23795513	three_prime_	LYPLA2	lysophospholipase 2	mRNA
chr6	57008625	57008662	three_prime_	BEND6	BEN domain containing 6	mRNA
chr1	92389543	92389602	three_prime_	RPAP2	RNA polymerase II associated protein 2	mRNA
chr4	112193795	112193855	three_prime_	FAM241A	family with sequence similarity 241 member A	mRNA
chr1	203741174	203741216	three_prime_	ATP2B4	ATPase plasma membrane Ca2+ transporting 4	mRNA
chr16	15409930	15409986	three_prime_	MPV17L	MPV17 mitochondrial membrane protein-like	mRNA
chr5	1461612	1461681	three_prime_	LPCAT1	lysophosphatidylcholine acyltransferase 1	mRNA

chr7	121349777	121349846	three_prime_	FAM3C	family with sequence similarity 3 member C	mRNA
chr12	13216525	13216594	three_prime_	EMP1	epithelial membrane protein 1	mRNA
chr19	36238579	36238623	three_prime_	ZNF146	zinc finger protein 146	mRNA
chr8	61502978	61503047	three_prime_	ASPH	aspartate beta-hydroxylase	mRNA
chr3	196829964	196830033	three_prime_	PAK2	p21 (RAC1) activated kinase 2	mRNA
chr5	97093596	97093627	three_prime_	LIX1	limb and CNS expressed 1	mRNA
chr2	70296704	70296738	three_prime_	FAM136A	family with sequence similarity 136 member A	mRNA
chr3	100364805	100364874	three_prime_	TOMM70A	translocase of outer mitochondrial membrane 70 homolog A (S. cerevisiae)	mRNA
chr1	16396082	16396129	three_prime_	SZRD1	SUZ RNA binding domain containing 1	mRNA
chr11	110231533	110231580	three_prime_	RDX	radixin, transcript variant 4	mRNA
chr21	37202518	37202569	three_prime_	TTC3	tetratricopeptide repeat domain 3	mRNA
chr11	35809782	35809827	three_prime_	TRIM44	tripartite motif containing 44	mRNA
chr7	135928799	135928869	three_prime_	MTPN	myotrophin	mRNA
chr3	42646929	42646987	three_prime_	NKTR	natural killer cell triggering receptor	mRNA
chr7	75410828	75410876	three_prime_	TRIM73	tripartite motif containing 73	mRNA
chr1	32365343	32365411	three_prime_	BSDC1	BSD domain containing 1	mRNA
chr11	34100070	34100141	three_prime_	CAPRIN1	cell cycle associated protein 1	mRNA
chr3	123492064	123492135	three_prime_	HACD2	3-hydroxyacyl-CoA dehydratase 2	mRNA
chr13	29509490	29509561	three_prime_	SLC7A1	solute carrier family 7 member 1	mRNA
chr11	77666058	77666097	three_prime_	RSF1	remodeling and spacing factor 1	mRNA
chr3	191267690	191267732	three_prime_	UTS2B	Urotensin 2B	mRNA
chrX	70063837	70063867	three_prime_	OTUD6A	OTU deubiquitinase 6A	mRNA
chr1	43622436	43622508	three_prime_	PTPRF	protein tyrosine phosphatase receptor type F	mRNA
chr2	85321521	85321587	three_prime_	TGOLN2	trans-golgi network protein 2	mRNA
chr8	61624730	61624802	three_prime_	ASPH	aspartate beta-hydroxylase	mRNA
chr6	113861101	113861164	three_prime_	MARCKS	myristoylated alanine-rich protein kinase C substrate	mRNA
chr17	44034749	44034788	three_prime_	LSM12	LSM12 homolog	mRNA
chr2	182919665	182919715	three_prime_	NCKAP1	NCK associated protein 1	mRNA
chr5	160252037	160252094	three_prime_	CCNJL	cyclin J like	mRNA
chr10	78040614	78040687	three_prime_	RPS24	ribosomal protein S24	mRNA
chr15	100916292	100916364	three_prime_	ALDH1A3	aldehyde dehydrogenase 1 family member A3	mRNA
chr8	97852431	97852494	three_prime_	LAPTM4B	lysosomal protein transmembrane 4 beta	mRNA
chr11	128460108	128460173	three_prime_	ETS1	ETS proto-oncogene 1	mRNA
chr12	79774028	79774059	three_prime_	PPP1R12A	protein phosphatase 1 regulatory subunit 12A	mRNA
chr9	112221257	112221296	three_prime_	PTBP3	polypyrimidine tract binding protein 3	mRNA
chr9	112221297	112221330	three_prime_	PTBP3	polypyrimidine tract binding protein 3	mRNA
chr17	68531254	68531325	three_prime_	PRKAR1A	protein kinase cAMP-dependent type I regulatory subunit alpha	mRNA
chr2	8858183	8858256	three_prime_	MBOAT2	membrane bound O-acyltransferase domain containing 2	mRNA
chr5	139683824	139683862	three_prime_	CXXC5	CXXC finger protein 5	mRNA
chr2	36548683	36548755	three_prime_	CRIM1	cysteine rich transmembrane BMP regulator 1	mRNA
chr22	39312897	39312970	three_prime_	RPL3	ribosomal protein L3	mRNA
chr10	123165193	123165246	three_prime_	BUB3	BUB3 mitotic checkpoint protein	mRNA
chr16	81386420	81386472	three_prime_	GAN	gigaxonin	mRNA
chr20	45326979	45327053	three_prime_	SDC4	syndecan 4	mRNA
chr10	103595477	103595545	three_prime_	SH3PXD2A	SH3 and PX domains 2A	mRNA
chr12	93399922	93399969	three_prime_	NUDT4	nudix hydrolase 4	mRNA
chr7	103099558	103099596	three_prime_	ARMC10	armadillo repeat containing 10	mRNA
chr2	177219638	177219690	three_prime_	HNRNPA3	heterogeneous nuclear ribonucleoprotein A3	mRNA
chr14	105479840	105479914	three_prime_	CRIP2	cysteine rich protein 2	mRNA
chr4	173322285	173322360	three_prime_	GALNT7	polypeptide N-acetylgalactosaminyltransferase 7	mRNA
chr5	179836931	179837005	three_prime_	SQSTM1	sequestosome 1	mRNA
chr3	196049296	196049366	three_prime_	TFRC	transferrin receptor	mRNA
chr11	66003930	66004005	three_prime_	BANF1	barrier to autointegration factor 1	mRNA
chr12	56238757	56238801	three_prime_	ANKRD52	ankyrin repeat domain 52	mRNA
chr2	112117306	112117366	three_prime_	TMEM87B	transmembrane protein 87B	mRNA
chr6	31830106	31830144	three_prime_	HSPA1B	heat shock 70kDa protein 1B	mRNA
chr7	44578998	44579045	three_prime_	TMED4	transmembrane p24 trafficking protein 4	mRNA
chr19	13141054	13141121	three_prime_	NACC1	nucleus accumbens associated 1, BEN and BTB (POZ) domain containing	mRNA
chr3	189895006	189895068	three_prime_	TP63	tumor protein p63	mRNA
chr13	42321747	42321822	three_prime_	AKAP11	A kinase (PRKA) anchor protein 11	mRNA
chr2	219183045	219183120	three_prime_	RETREG2	reticulophagy regulator family member 2	mRNA

chr7	152121967	152122033	three_prime_	GALNT11	polypeptide N-acetylgalactosaminyltransferase 11	mRNA
chr17	50751514	50751525	three_prime_	LUC7L3	LUC7 like 3 pre-mRNA splicing factor	mRNA
chr10	119168246	119168312	three_prime_	PRDX3	peroxiredoxin 3	mRNA
chr1	30932178	30932254	three_prime_	PUM1	pumilio RNA binding family member 1	mRNA
chr16	23067892	23067957	three_prime_	USP31	ubiquitin specific peptidase 31	mRNA
chr1	150265889	150265942	three_prime_	APH1A	aph-1 homolog A, gamma-secretase subunit	mRNA
chr2	28800356	28800416	three_prime_	PPP1CB	protein phosphatase 1 catalytic subunit beta	mRNA
chr3	23920020	23920096	three_prime_	RPL15	ribosomal protein L15	mRNA
chr3	197782640	197782716	three_prime_	FYTTD1	forty-two-three domain containing 1,	mRNA
chr14	70325381	70325427	three_prime_	COX16	cytochrome c oxidase assembly factor COX16	mRNA
chr1	149943729	149943794	three_prime_	OTUD7B	OTU deubiquitinase 7B	mRNA
chr6	89643415	89643490	three_prime_	MDN1	midasin AAA ATPase 1	mRNA
chr6	116495759	116495800	three_prime_	TRAPPC3L	trafficking protein particle complex 3 like	mRNA
chr20	33854167	33854216	three_prime_	CHMP4B	charged multivesicular body protein 4B	mRNA
chr10	72011415	72011492	three_prime_	CHST3	carbohydrate (chondroitin 6) sulfotransferase 3	mRNA
chr20	44907568	44907645	three_prime_	YWHAB	tyrosine 3-monooxygenase/tryptophan 5-monooxygenase activation protein beta	mRNA
chr1	26854728	26854793	three_prime_	ZDHHC18	zinc finger DHHC-type containing 18	mRNA
chr16	13239078	13239126	three_prime_	SHISA9	shisa family member 9	mRNA
chr4	69724204	69724248	three_prime_	SULT1B1	sulfotransferase family 1B member 1	mRNA
chr16	1349381	1349458	three_prime_	TSR3	TSR3, 20S rRNA accumulation, homolog (S. cerevisiae)	mRNA
chr12	909981	910059	three_prime_	WNK1	WNK lysine deficient protein kinase 1	mRNA
chr15	73560284	73560343	three_prime_	NPTN	neuroplastin	mRNA
chr6	121449220	121449242	three_prime_	GJA1	gap junction protein, alpha 1, 43kDa	mRNA
chr8	18056471	18056500	three_prime_	ASAH1	N-acylsphingosine amidohydrolase 1	mRNA
chr18	216361	216397	three_prime_	THOC1	THO complex 1	mRNA
chr15	65577432	65577473	three_prime_	HACD3	3-hydroxyacyl-CoA dehydratase 3	mRNA
chr5	6670993	6671071	three_prime_	SRD5A1	steroid 5 alpha-reductase 1	mRNA
chr20	51597494	51597573	three_prime_	ATP9A	ATPase phospholipid transporting 9A	mRNA
chr1	171593292	171593371	three_prime_	PRRC2C	proline rich coiled-coil 2C	mRNA
chr11	61802091	61802162	three_prime_	FADS1	fatty acid desaturase 1	mRNA
chr3	122571919	122571972	three_prime_	DTX3L	deltex E3 ubiquitin ligase 3L	mRNA
chr18	26016762	26016831	three_prime_	SS18	SS18 subunit of BAF chromatin remodeling complex	mRNA
chr14	75651177	75651218	three_prime_	ERG28	ergosterol biosynthesis 28 homolog	mRNA
chr11	102398815	102398891	three_prime_	TMEM123	transmembrane protein 123	mRNA
chr18	724125	724203	three_prime_	YES1	YES proto-oncogene 1	mRNA
chr14	103137285	103137337	three_prime_	TNFAIP2	TNF alpha induced protein 2	mRNA
chr15	97971300	97971368	three_prime_	ARRDC4	arrestin domain containing 4	mRNA
chr1	169131611	169131679	three_prime_	ATP1B1	ATPase Na+/K+ transporting subunit beta 1	mRNA
chr20	44908209	44908286	three_prime_	YWHAB	tyrosine 3-monooxygenase/tryptophan 5-monooxygenase activation protein beta	mRNA
chr7	106090638	106090675	three_prime_	SYPL1	synaptophysin like 1	mRNA
chr9	94460683	94460716	three_prime_	MFSD14B	major facilitator superfamily domain containing 14B	mRNA
chr3	194402828	194402896	three_prime_	ATP13A3	ATPase 13A3	mRNA
chr12	84860988	84861057	three_prime_	SLC6A15	solute carrier family 6 member 15	mRNA
chr1	211573575	211573636	three_prime_	SLC30A1	solute carrier family 30 (zinc transporter), member 1	mRNA
chr4	98872828	98872910	three_prime_	EIF4E	eukaryotic translation initiation factor 4E	mRNA
chr19	42377408	42377490	three_prime_	MEGF8	multiple EGF like domains 8	mRNA
chr5	138507332	138507413	three_prime_	ETF1	eukaryotic translation termination factor 1	mRNA
chr15	98959955	98960038	three_prime_	IGF1R	insulin like growth factor 1 receptor	mRNA
chr3	133589805	133589853	three_prime_	CDV3	CDV3 homolog	mRNA
chr3	133589854	133589888	three_prime_	CDV3	CDV3 homolog	mRNA
chr14	49894637	49894720	three_prime_	ARF6	ADP-ribosylation factor 6	mRNA
chr1	162523336	162523380	three_prime_	UHMK1	U2AF homology motif kinase 1	mRNA
chr9	86027611	86027653	three_prime_	GOLM1	golgi membrane protein 1	mRNA
chr3	10150804	10150845	three_prime_	VHL	von Hippel-Lindau tumor suppressor, E3 ubiquitin protein ligase	mRNA
chr16	67229427	67229500	three_prime_	FHOD1	formin homology 2 domain containing 1	mRNA
chr3	128070778	128070862	three_prime_	SEC61A1	SEC61 translocon alpha 1 subunit	mRNA
chr5	72911366	72911447	three_prime_	TNPO1	transportin 1	mRNA
chr20	51599806	51599882	three_prime_	ATP9A	ATPase phospholipid transporting 9A	mRNA
chr16	57664938	57664983	three_prime_	ADGRG1	adhesion G protein-coupled receptor G1	mRNA
chr3	49533574	49533641	three_prime_	DAG1	dystroglycan 1	mRNA
chr20	51599551	51599617	three_prime_	ATP9A	ATPase phospholipid transporting 9A	mRNA

chr5	95888068	95888151	three_prime_	ELL2	elongation factor for RNA polymerase II 2	mRNA
chr7	116559524	116559584	three_prime_	CAV1	caveolin 1	mRNA
chr12	6975857	6975938	three_prime_	EMG1	EMG1 N1-specific pseudouridine methyltransferase	mRNA
chr3	98796216	98796256	three_prime_	DCBLD2	discoidin	mRNA
chr19	34227820	34227893	three_prime_	LSM14A	LSM14A mRNA processing body assembly factor	mRNA
chr4	105969765	105969828	three_prime_	NPNT	nephronectin	mRNA
chr9	128694654	128694723	three_prime_	SET	SET nuclear proto-oncogene	mRNA
chr10	96519760	96519839	three_prime_	TM9SF3	transmembrane 9 superfamily member 3	mRNA
chr6	79201513	79201599	three_prime_	HMGN3	high mobility group nucleosomal binding domain 3	mRNA
chr22	19037615	19037655	three_prime_	DGCR2	DiGeorge syndrome critical region gene 2	mRNA
chr7	73832659	73832690	three_prime_	CLDN4	claudin 4	mRNA
chr14	49583589	49583675	three_prime_	RPS29	ribosomal protein S29	mRNA
chr1	119914385	119914460	three_prime_	NOTCH2	notch receptor 2	mRNA
chr3	58171753	58171840	three_prime_	FLNB	filamin B	mRNA
chr2	178505871	178505906	three_prime_	PLEKHA3	pleckstrin homology domain containing A3	mRNA
chr16	4383271	4383336	three_prime_	VASN	vasorin	mRNA
chr17	44206436	44206523	three_prime_	UBTF	upstream binding transcription factor	mRNA
chr11	111293731	111293818	three_prime_	COLCA1	colorectal cancer associated 1	mRNA
chr9	127940947	127941010	three_prime_	FAM102A	family with sequence similarity 102 member A	mRNA
chr1	234606473	234606561	three_prime_	IRF2BP2	interferon regulatory factor 2 binding protein 2	mRNA
chr6	47624749	47624798	three_prime_	CD2AP	CD2-associated protein	mRNA
chr1	89181876	89181913	three_prime_	GBP4	guanylate binding protein 4	mRNA
chr3	123491987	123492060	three_prime_	HACD2	3-hydroxyacyl-CoA dehydratase 2	mRNA
chr2	173965297	173965331	three_prime_	SP3	Sp3 transcription factor	mRNA
chr18	21867869	21867924	three_prime_	MIB1	mindbomb E3 ubiquitin protein ligase 1	mRNA
chr6	75253308	75253394	three_prime_	TMEM30A	transmembrane protein 30A	mRNA
chr19	5206129	5206164	three_prime_	PTPRS	protein tyrosine phosphatase receptor type S	mRNA
chr3	196051221	196051305	three_prime_	TFRC	transferrin receptor	mRNA
chr5	80141115	80141191	three_prime_	SERINC5	serine incorporator 5	mRNA
chr12	8941137	8941213	three_prime_	M6PR	mannose-6-phosphate receptor	mRNA
chr12	125030942	125031025	three_prime_	BRI3BP	BRI3 binding protein	mRNA
chr9	136440858	136440946	three_prime_	SEC16A	SEC16 homolog A, endoplasmic reticulum export factor	mRNA
chr17	56938428	56938473	three_prime_	COIL	coilin	mRNA
chr6	159680357	159680446	three_prime_	SOD2	superoxide dismutase 2	mRNA
chr2	3515579	3515619	three_prime_	ADI1	acireductone dioxygenase 1	mRNA
chr6	34417458	34417547	three_prime_	RPS10	ribosomal protein S10	mRNA
chr1	42926291	42926380	three_prime_	SLC2A1	solute carrier family 2 member 1	mRNA
chr11	33709235	33709325	three_prime_	CD59	CD59 molecule	mRNA
chr18	30992356	30992431	three_prime_	DSC3	desmocollin 3	mRNA
chr14	75133322	75133412	three_prime_	TMED10	transmembrane p24 trafficking protein 10	mRNA
chr17	39671905	39671976	three_prime_	PGAP3	post-GPI attachment to proteins phospholipase 3	mRNA
chr9	112221333	112221408	three_prime_	PTBP3	polypyrimidine tract binding protein 3	mRNA
chr7	140453975	140454054	three_prime_	MKRN1	makorin ring finger protein 1	mRNA
chr8	56960180	56960257	three_prime_	IMPAD1	inositol monophosphatase domain containing 1	mRNA
chr19	53874967	53875059	three_prime_	MYADM	myeloid associated differentiation marker	mRNA
chr6	109367810	109367898	three_prime_	CD164	CD164 molecule	mRNA
chr20	37241527	37241619	three_prime_	RPN2	ribophorin II	mRNA
chr4	127721473	127721545	three_prime_	INTU	inturned planar cell polarity protein	mRNA
chr9	6532735	6532801	three_prime_	GLDC	glycine dehydrogenase (decarboxylating)	mRNA
chr8	100919778	100919871	three_prime_	YWHAZ	tyrosine 3-monooxygenase/tryptophan 5-monooxygenase activation protein zeta	mRNA
chr15	42210686	42210761	three_prime_	TMEM87A	transmembrane protein 87A	mRNA
chr1	46677044	46677132	three_prime_	EFCAB14	EF-hand calcium binding domain 14	mRNA
chr3	152463715	152463809	three_prime_	MBNL1	muscleblind like splicing regulator 1	mRNA
chr8	142784896	142784990	three_prime_	LY6D	lymphocyte antigen 6 complex, locus D	mRNA
chr12	92143942	92143988	three_prime_	BTG1	B-cell translocation gene 1, anti-proliferative	mRNA
chr16	19116681	19116776	three_prime_	ITPRIPL2	inositol 1,4,5-trisphosphate receptor interacting protein-like 2	mRNA
chr3	149173144	149173239	three_prime_	HPS3	HPS3 biogenesis of lysosomal organelles complex 2 subunit 1	mRNA
chr1	154582305	154582357	three_prime_	ADAR	adenosine deaminase RNA specific	mRNA
chr12	46358290	46358385	three_prime_	SLC38A2	solute carrier family 38 member 2	mRNA
chr14	63684982	63685058	three_prime_	SGPP1	sphingosine-1-phosphate phosphatase 1	mRNA
chr1	154583289	154583356	three_prime_	ADAR	adenosine deaminase RNA specific	mRNA

chrX	154436247	154436342	three_prime_	ATP6AP1	ATPase H+ transporting accessory protein 1	mRNA
chr16	15411436	15411524	three_prime_	MPV17L	MPV17 mitochondrial membrane protein-like	mRNA
chr9	129738333	129738429	three_prime_	PTGES	prostaglandin E synthase	mRNA
chr14	21210586	21210680	three_prime_	HNRNPC	heterogeneous nuclear ribonucleoprotein C	mRNA
chr16	19121087	19121174	three_prime_	ITPRIPL2	inositol 1,4,5-trisphosphate receptor interacting protein-like 2	mRNA
chr17	50090385	50090479	three_prime_	ITGA3	integrin subunit alpha 3	mRNA
chr14	77508393	77508478	three_prime_	SPTLC2	serine palmitoyltransferase long chain base subunit 2	mRNA
chr19	48382957	48383050	three_prime_	KDELR1	KDEL endoplasmic reticulum protein retention receptor 1	mRNA
chr3	42646832	42646928	three_prime_	NKTR	natural killer cell triggering receptor	mRNA
chr1	92392077	92392153	three_prime_	RPAP2	RNA polymerase II associated protein 2	mRNA
chr12	12917244	12917341	three_prime_	GPRC5A	G protein-coupled receptor, class C, group 5, member A	mRNA
chr1	202015800	202015845	three_prime_	ELF3	E74 like ETS transcription factor 3	mRNA
chr16	10530199	10530293	three_prime_	EMP2	epithelial membrane protein 2	mRNA
chr5	83054654	83054690	three_prime_	TMEM167A	transmembrane protein 167A	mRNA
chr1	24673052	24673097	three_prime_	SRRM1	serine and arginine repetitive matrix 1,	mRNA
chr4	168927458	168927508	three_prime_	PALLD	palladin	mRNA
chr11	65854946	65855040	three_prime_	CFL1	cofilin 1	mRNA
chr19	53876312	53876406	three_prime_	MYADM	myeloid associated differentiation marker	mRNA
chr11	95131275	95131372	three_prime_	ENDOD1	endonuclease domain containing 1	mRNA
chr19	18279779	18279850	three_prime_	JUND	jun D proto-oncogene	mRNA
chr2	170993414	170993442	three_prime_	TLK1	tousled like kinase 1	mRNA
chr2	38068867	38068966	three_prime_	CYP1B1	cytochrome P450 family 1 subfamily B member 1	mRNA
chr5	10435408	10435473	three_prime_	MARCHF6	membrane associated ring-CH-type finger 6	mRNA
chr18	63598498	63598551	three_prime_	SERPINB13	serpin family B member 13	mRNA
chr3	33144209	33144308	three_prime_	CRTAP	cartilage associated protein	mRNA
chr5	97034494	97034584	three_prime_	LNPEP	leucyl and cystinyl aminopeptidase	mRNA
chr12	6238060	6238087	three_prime_	CD9	CD9 molecule	mRNA
chr11	118751384	118751438	three_prime_	DDX6	DEAD-box helicase 6	mRNA
chr1	225496319	225496418	three_prime_	ENAH	ENAH actin regulator	mRNA
chr4	138164912	138165011	three_prime_	SLC7A11	solute carrier family 7 member 11	mRNA
chr22	19036392	19036417	three_prime_	DGCR2	DiGeorge syndrome critical region gene 2	mRNA
chr20	32850065	32850140	three_prime_	MAPRE1	microtubule-associated protein, RP/EB family, member 1	mRNA
chr21	17569286	17569357	three_prime_	CXADR	CXADR Ig-like cell adhesion molecule	mRNA
chr9	113261343	113261413	three_prime_	SLC31A1	solute carrier family 31 (copper transporter), member 1	mRNA
chr11	125620892	125620988	three_prime_	STT3A	STT3 oligosaccharyltransferase complex catalytic subunit A	mRNA
chr10	87754296	87754390	three_prime_	ATAD1	ATPase family AAA domain containing 1	mRNA
chr11	47468271	47468333	three_prime_	CELF1	CUGBP Elav-like family member 1	mRNA
chr1	26854797	26854897	three_prime_	ZDHHC18	zinc finger DHHC-type containing 18	mRNA
chr1	9729917	9730018	three_prime_	CLSTN1	calsyntenin 1	mRNA
chr5	95795777	95795874	three_prime_	RHOBTB3	Rho related BTB domain containing 3	mRNA
chr12	95658318	95658419	three_prime_	NTN4	netrin 4	mRNA
chr3	182942706	182942775	three_prime_	DCUN1D1	defective in cullin neddylation 1 domain containing 1	mRNA
chr12	53479446	53479547	three_prime_	PCBP2	poly(rC) binding protein 2	mRNA
chr9	112218970	112219069	three_prime_	PTBP3	polypyrimidine tract binding protein 3	mRNA
chr15	73560811	73560913	three_prime_	NPTN	neuroplastin	mRNA
chr5	159157601	159157648	three_prime_	RNF145	ring finger protein 145	mRNA
chr2	175073457	175073559	three_prime_	SF3B1	splicing factor 3b subunit 1	mRNA
chrX	54444959	54445002	three_prime_	TSR2	TSR2, 20S rRNA accumulation, homolog (S. cerevisiae)	mRNA
chr18	672976	673078	three_prime_	TYMS	thymidylate synthetase	mRNA
chr12	112508317	112508409	three_prime_	PTPN11	protein tyrosine phosphatase non-receptor type 11	mRNA
chr2	241352098	241352127	three_prime_	SEPTIN2	septin 2	mRNA
chr12	26973746	26973844	three_prime_	TM7SF3	transmembrane 7 superfamily member 3	mRNA
chr15	98962557	98962568	three_prime_	IGF1R	insulin like growth factor 1 receptor	mRNA
chr14	70370791	70370876	three_prime_	SYNJ2BP-COX16	SYNJ2BP-COX16 readthrough	mRNA
chr21	31668576	31668638	three_prime_	SOD1	superoxide dismutase 1	mRNA
chr19	10513307	10513360	three_prime_	S1PR5	sphingosine-1-phosphate receptor 5	mRNA
chr11	70435266	70435365	three_prime_	CTTN	cortactin	mRNA
chr3	194402947	194403050	three_prime_	ATP13A3	ATPase 13A3	mRNA
chr1	209786515	209786619	three_prime_	IRF6	interferon regulatory factor 6	mRNA
chr1	20743271	20743353	three_prime_	HP1BP3	heterochromatin protein 1 binding protein 3	mRNA
chr2	54972193	54972284	three_prime_	RTN4	reticulon 4	mRNA

chr19	48615343	48615430	three_prime_	RPL18	ribosomal protein L18	mRNA
chr1	22091815	22091913	three_prime_	CDC42	Cell Division Cycle 42	mRNA
chr6	106970836	106970914	three_prime_	CD24	CD24 molecule	mRNA
chr6	106970924	106970939	three_prime_	CD24	CD24 molecule	mRNA
chr14	69462001	69462106	three_prime_	SLC39A9	solute carrier family 39 member 9	mRNA
chr1	43622376	43622434	three_prime_	PTPRF	protein tyrosine phosphatase receptor type F	mRNA
chr10	74119630	74119708	three_prime_	VCL	vinculin	mRNA
chrX	103358303	103358366	three_prime_	TCEAL9	transcription elongation factor A like 9	mRNA
chr19	45609519	45609569	three_prime_	EML2	EMAP like 2	mRNA
chr1	171593138	171593201	three_prime_	PRRC2C	proline rich coiled-coil 2C	mRNA
chr19	42377300	42377353	three_prime_	MEGF8	multiple EGF like domains 8	mRNA
chr2	212999914	212999997	three_prime_	IKZF2	IKAROS family zinc finger 2	mRNA
chr2	182781772	182781845	three_prime_	DNAJC10	DnaJ heat shock protein family (Hsp40) member C10	mRNA
chr3	195568836	195568941	three_prime_	APOD	apolipoprotein D	mRNA
chr20	44908022	44908124	three_prime_	YWHAB	tyrosine 3-monooxygenase/tryptophan 5-monooxygenase activation protein beta	mRNA
chr10	103595294	103595395	three_prime_	SH3PXD2A	SH3 and PX domains 2A	mRNA
chr1	144440487	144440563	three_prime_	NBPF15	NBPF member 15	mRNA
chr14	74904234	74904268	three_prime_	RPS6KL1	ribosomal protein S6 kinase like 1	mRNA
chr20	47655646	47655753	three_prime_	NCOA3	nuclear receptor coactivator 3	mRNA
chr6	137197855	137197934	three_prime_	IFNGR1	interferon gamma receptor 1	mRNA
chr19	44663347	44663381	three_prime_	PVR	PVR cell adhesion molecule	mRNA
chr5	168549666	168549775	three_prime_	PANK3	pantothenate kinase 3	mRNA
chr3	49534524	49534622	three_prime_	DAG1	dystroglycan 1	mRNA
chr3	49534623	49534631	three_prime_	DAG1	dystroglycan 1	mRNA
chr11	102398003	102398111	three_prime_	TMEM123	transmembrane protein 123	mRNA
chr4	183639944	183640023	three_prime_	RWDD4	RWD domain containing 4	mRNA
chr16	19118499	19118543	three_prime_	ITPRIPL2	inositol 1,4,5-trisphosphate receptor interacting protein-like 2	mRNA
chr3	128620028	128620101	three_prime_	RPN1	ribophorin I	mRNA
chr5	151268044	151268154	three_prime_	GM2A	GM2 ganglioside activator	mRNA
chr11	95130993	95131097	three_prime_	ENDOD1	endonuclease domain containing 1	mRNA
chr1	234606162	234606231	three_prime_	IRF2BP2	interferon regulatory factor 2 binding protein 2	mRNA
chrX	20152066	20152177	three_prime_	RPS6KA3	ribosomal protein S6 kinase A3	mRNA
chr19	15160331	15160442	three_prime_	NOTCH3	notch receptor 3	mRNA
chr1	116987216	116987315	three_prime_	PTGFRN	prostaglandin F2 receptor inhibitor	mRNA
chr3	64002437	64002549	three_prime_	ATXN7	ataxin 7	mRNA
chr16	15413126	15413227	three_prime_	MPV17L	MPV17 mitochondrial membrane protein-like	mRNA
chr2	214932104	214932195	three_prime_	ABCA12	ATP binding cassette subfamily A member 12	mRNA
chr1	233384645	233384756	three_prime_	MAP3K21	mitogen-activated protein kinase kinase kinase 21	mRNA
chr14	69771536	69771644	three_prime_	SRSF5	serine and arginine rich splicing factor 5	mRNA
chr11	57552125	57552233	three_prime_	UBE2L6	ubiquitin conjugating enzyme E2 L6	mRNA
chr14	24308094	24308182	three_prime_	NOP9	NOP9 nucleolar protein	mRNA
chr22	36226701	36226744	three_prime_	APOL2	apolipoprotein L2	mRNA
chr16	70249596	70249673	three_prime_	EXOSC6	exosome component 6	mRNA
chr4	55425484	55425595	three_prime_	TMEM165	transmembrane protein 165	mRNA
chr3	105575926	105576040	three_prime_	ALCAM	activated leukocyte cell adhesion molecule	mRNA
chr20	64275257	64275343	three_prime_	PCMTD2	protein-L-isoaspartate (D-aspartate) O-methyltransferase domain containing 2	mRNA
chr11	88293905	88293990	three_prime_	CTSC	cathepsin C	mRNA
chr10	100549793	100549881	three_prime_	HIF1AN	hypoxia inducible factor 1 subunit alpha inhibitor	mRNA
chr2	96185069	96185126	three_prime_	STARD7	StAR related lipid transfer domain containing 7	mRNA
chr5	151806330	151806420	three_prime_	G3BP1	G3BP stress granule assembly factor 1	mRNA
chr8	56960045	56960160	three_prime_	IMPAD1	inositol monophosphatase domain containing 1	mRNA
chr7	135928959	135929034	three_prime_	MTPN	myotrophin	mRNA
chr3	105575799	105575890	three_prime_	ALCAM	activated leukocyte cell adhesion molecule	mRNA
chr16	84975548	84975648	three_prime_	ZDHHC7	zinc finger DHHC-type containing 7	mRNA
chr8	37754977	37755093	three_prime_	ERLIN2	ER lipid raft associated 2	mRNA
chrX	153700728	153700844	three_prime_	BCAP31	B cell receptor associated protein 31	mRNA
chr10	5457879	5457996	three_prime_	NET1	neuroepithelial cell transforming 1	mRNA
chr17	4959282	4959399	three_prime_	SPAG7	sperm associated antigen 7	mRNA
chr2	9584359	9584440	three_prime_	YWHAQ	tyrosine 3-monooxygenase/tryptophan 5-monooxygenase activation protein theta	mRNA
chr14	95190864	95190908	three_prime_	CLMN	calmin	mRNA
chr6	42101268	42101373	three_prime_	C6orf132	chromosome 6 open reading frame 132	mRNA

chr9	136007318	136007413	three_prime_	NACC2	NACC family member 2	mRNA
chr5	10264741	10264853	three_prime_	CCT5	chaperonin containing TCP1 subunit 5	mRNA
chr8	6757613	6757716	three_prime_	AGPAT5	1-acylglycerol-3-phosphate O-acyltransferase 5	mRNA
chr3	13316252	13316358	three_prime_	NUP210	nucleoporin 210	mRNA
chr12	50475976	50476023	three_prime_	LARP4	La ribonucleoprotein 4	mRNA
chr7	140453252	140453292	three_prime_	MKRN1	makorin ring finger protein 1	mRNA
chr7	140453335	140453370	three_prime_	MKRN1	makorin ring finger protein 1	mRNA
chr11	62888644	62888762	three_prime_	SLC3A2	solute carrier family 3 member 2	mRNA
chr1	1056001	1056113	three_prime_	AGRN	agrin	mRNA
chr14	75134282	75134351	three_prime_	TMED10	transmembrane p24 trafficking protein 10	mRNA
chr12	111809692	111809760	three_prime_	ALDH2	aldehyde dehydrogenase 2 family member	mRNA
chr17	29256974	29257050	three_prime_	NUFIP2	nuclear FMR1 interacting protein 2	mRNA
chr19	54193531	54193599	three_prime_	TSEN34	tRNA splicing endonuclease subunit 34	mRNA
chr2	197503263	197503342	three_prime_	HSPE1	heat shock protein family E	mRNA
chrX	68533030	68533094	three_prime_	YIPF6	Yip1 domain family member 6	mRNA
chr20	45816804	45816852	three_prime_	UBE2C	ubiquitin conjugating enzyme E2 C	mRNA
chr9	113261807	113261894	three_prime_	SLC31A1	solute carrier family 31 (copper transporter), member 1	mRNA
chr15	65888516	65888587	three_prime_	RAB11A	RAB11A, member RAS oncogene family	mRNA
chr1	116987617	116987665	three_prime_	PTGFRN	prostaglandin F2 receptor inhibitor	mRNA
chr5	256542	256662	three_prime_	SDHA	succinate dehydrogenase complex flavoprotein subunit A	mRNA
chr12	48938717	48938787	three_prime_	ARF3	ADP ribosylation factor 3	mRNA
chr6	31829874	31829979	three_prime_	HSPA1B	heat shock 70kDa protein 1B	mRNA
chr6	79201635	79201726	three_prime_	HMGN3	high mobility group nucleosomal binding domain 3	mRNA
chr3	141925657	141925778	three_prime_	ATP1B3	ATPase Na+/K+ transporting subunit beta 3	mRNA
chr16	10529028	10529092	three_prime_	EMP2	epithelial membrane protein 2	mRNA
chr7	23505078	23505163	three_prime_	TRA2A	transformer 2 alpha homolog	mRNA
chr16	68835369	68835470	three_prime_	CDH1	cadherin 1	mRNA
chr14	63685849	63685954	three_prime_	SGPP1	sphingosine-1-phosphate phosphatase 1	mRNA
chr12	1787976	1788099	three_prime_	ADIPOR2	adiponectin receptor 2	mRNA
chr1	235111003	235111078	three_prime_	TOMM20	translocase of outer mitochondrial membrane 20	mRNA
chr2	135783853	135783977	three_prime_	UBXN4	UBX domain protein 4	mRNA
chr3	149173560	149173678	three_prime_	HPS3	HPS3 biogenesis of lysosomal organelles complex 2 subunit 1	mRNA
chr3	160501844	160501963	three_prime_	KPNA4	karyopherin subunit alpha 4	mRNA
chr2	20201983	20202097	three_prime_	SDC1	syndecan 1	mRNA
chr12	64695436	64695528	three_prime_	RASSF3	Ras association domain family member 3	mRNA
chr1	156465525	156465650	three_prime_	MEF2D	myocyte enhancer factor 2D	mRNA
chr4	39780234	39780346	three_prime_	UBE2K	ubiquitin conjugating enzyme E2 K	mRNA
chr6	2785265	2785390	three_prime_	WRNIP1	WRN helicase interacting protein 1	mRNA
chr16	81380504	81380612	three_prime_	GAN	gigaxonin	mRNA
chr14	75131804	75131930	three_prime_	TMED10	transmembrane p24 trafficking protein 10	mRNA
chr20	45325971	45326097	three_prime_	SDC4	syndecan 4	mRNA
chr1	169132215	169132330	three_prime_	ATP1B1	ATPase Na+/K+ transporting subunit beta 1	mRNA
chr15	98964366	98964466	three_prime_	IGF1R	insulin like growth factor 1 receptor	mRNA
chr12	909853	909979	three_prime_	WNK1	WNK lysine deficient protein kinase 1	mRNA
chr15	40872540	40872666	three_prime_	RHOV	ras homolog family member V	mRNA
chr11	130877273	130877313	three_prime_	SNX19	sorting nexin 19	mRNA
chr16	16142142	16142253	three_prime_	ABCC1	ATP binding cassette subfamily C member 1	mRNA
chr1	29326258	29326328	three_prime_	PTPRU	protein tyrosine phosphatase receptor type U	mRNA
chr19	11133617	11133743	three_prime_	LDLR	low density lipoprotein receptor	mRNA
chr3	128070882	128071010	three_prime_	SEC61A1	SEC61 translocon alpha 1 subunit	mRNA
chr11	57816697	57816773	three_prime_	CTNND1	catenin delta 1	mRNA
chr16	18791691	18791735	three_prime_	ARL6IP1	ADP ribosylation factor like GTPase 6 interacting protein 1	mRNA
chr16	18791749	18791815	three_prime_	ARL6IP1	ADP ribosylation factor like GTPase 6 interacting protein 1	mRNA
chr1	203852437	203852565	three_prime_	ZC3H11A	zinc finger CCCH-type containing 11A	mRNA
chr14	77506036	77506162	three_prime_	SPTLC2	serine palmitoyltransferase long chain base subunit 2	mRNA
chr6	158632372	158632489	three_prime_	TMEM181	transmembrane protein 181	mRNA
chr2	222943113	222943188	three_prime_	ACSL3	acyl-CoA synthetase long chain family member 3	mRNA
chr6	17615586	17615716	three_prime_	NUP153	nucleoporin 153	mRNA
chr14	103136919	103137049	three_prime_	TNFAIP2	TNF alpha induced protein 2	mRNA
chr1	156465700	156465776	three_prime_	MEF2D	myocyte enhancer factor 2D	mRNA
chr15	89197333	89197460	three_prime_	ABHD2	abhydrolase domain containing 2	mRNA

chr3	196348478	196348595	three_prime_	UBXN7	UBX domain protein 7	mRNA
chr3	195568706	195568828	three_prime_	APOD	apolipoprotein D	mRNA
chr17	40194279	40194395	three_prime_	RAPGEFL1	Rap guanine nucleotide exchange factor like 1	mRNA
chr19	15159633	15159740	three_prime_	NOTCH3	notch receptor 3	mRNA
chr6	7289522	7289652	three_prime_	SSR1	signal sequence receptor subunit 1	mRNA
chr6	31268915	31269047	three_prime_	HLA-C	major histocompatibility complex	mRNA
chr22	36266918	36267041	three_prime_	APOL1	apolipoprotein L1	mRNA
chr1	207795031	207795147	three_prime_	CD46	CD46 molecule	mRNA
chr5	176375182	176375218	three_prime_	ARL10	ADP-ribosylation factor-like 10	mRNA
chr5	176375235	176375299	three_prime_	ARL10	ADP-ribosylation factor-like 10	mRNA
chr18	723426	723558	three_prime_	YES1	YES proto-oncogene 1	mRNA
chr2	230820520	230820620	three_prime_	CAB39	calcium binding protein 39	mRNA
chr10	96522117	96522251	three_prime_	TM9SF3	transmembrane 9 superfamily member 3	mRNA
chr6	47232710	47232789	three_prime_	TNFRSF21	tumor necrosis factor receptor superfamily, member 21	mRNA
chr11	10307205	10307340	three_prime_	ADM	adrenomedullin	mRNA
chr6	34588848	34588959	three_prime_	ILRUN	inflammation and lipid regulator with UBA-like and NBR1-like domains	mRNA
chr7	155309609	155309740	three_prime_	INSIG1	insulin induced gene 1	mRNA
chr2	168770330	168770394	three_prime_	CERS6	ceramide synthase 6	mRNA
chr3	107807884	107807937	three_prime_	BBX	BBX high mobility group box domain containing	mRNA
chr14	54427774	54427841	three_prime_	CNIH1	cornichon family AMPA receptor auxiliary protein 1	mRNA
chr6	47232051	47232158	three_prime_	TNFRSF21	tumor necrosis factor receptor superfamily, member 21	mRNA
chr3	128813685	128813808	three_prime_	RAB7A	RAB7A, member RAS oncogene family	mRNA
chr15	77046212	77046243	three_prime_	TSPAN3	tetraspanin 3	mRNA
chr15	77046283	77046348	three_prime_	TSPAN3	tetraspanin 3	mRNA
chr19	47209809	47209918	three_prime_	SAE1	SUMO1 activating enzyme subunit 1	mRNA
chr3	48693787	48693823	three_prime_	IP6K2	inositol hexakisphosphate kinase 2	mRNA
chr1	152032537	152032673	three_prime_	S100A11	S100 calcium binding protein A11	mRNA
chr14	49895558	49895675	three_prime_	ARF6	ADP-ribosylation factor 6	mRNA
chr16	68698884	68699012	three_prime_	CDH3	cadherin 3	mRNA
chr19	53874630	53874762	three_prime_	MYADM	myeloid associated differentiation marker	mRNA
chr6	34587732	34587773	three_prime_	ILRUN	inflammation and lipid regulator with UBA-like and NBR1-like domains	mRNA
chr6	34587776	34587845	three_prime_	ILRUN	inflammation and lipid regulator with UBA-like and NBR1-like domains	mRNA
chr2	37105709	37105763	three_prime_	EIF2AK2	eukaryotic translation initiation factor 2 alpha kinase 2	mRNA
chr2	37105769	37105833	three_prime_	EIF2AK2	eukaryotic translation initiation factor 2 alpha kinase 2	mRNA
chr11	111294744	111294793	three_prime_	COLCA1	colorectal cancer associated 1	mRNA
chr8	100920401	100920539	three_prime_	YWHAZ	tyrosine 3-monooxygenase/tryptophan 5-monooxygenase activation protein zeta	mRNA
chr11	33708900	33709038	three_prime_	CD59	CD59 molecule	mRNA
chr2	177538566	177538664	three_prime_	AGPS	alkylglycerone phosphate synthase	mRNA
chr10	73917074	73917158	three_prime_	PLAU	plasminogen activator	mRNA
chr2	227559186	227559225	three_prime_	AGFG1	ArfGAP with FG repeats 1	mRNA
chr1	235111208	235111348	three_prime_	TOMM20	translocase of outer mitochondrial membrane 20	mRNA
chr9	105390049	105390189	three_prime_	SLC44A1	solute carrier family 44 member 1	mRNA
chr3	172820990	172821050	three_prime_	ECT2	epithelial cell transforming 2	mRNA
chr3	49533460	49533573	three_prime_	DAG1	dystroglycan 1	mRNA
chr1	53246343	53246478	three_prime_	LRP8	LDL receptor related protein 8	mRNA
chr19	50310219	50310360	three_prime_	MYH14	myosin heavy chain 14	mRNA
chr9	69715234	69715374	three_prime_	PTAR1	protein prenyltransferase alpha subunit repeat containing 1	mRNA
chr1	162523153	162523239	three_prime_	UHMK1	U2AF homology motif kinase 1	mRNA
chr18	723272	723413	three_prime_	YES1	YES proto-oncogene 1	mRNA
chr6	7287549	7287691	three_prime_	SSR1	signal sequence receptor subunit 1	mRNA
chr1	183144964	183145107	three_prime_	LAMC1	laminin, gamma 1 (formerly LAMB2)	mRNA
chr12	120699143	120699270	three_prime_	MLEC	malectin	mRNA
chr9	133362844	133362987	three_prime_	SURF4	surfeit 4	mRNA
chr17	37086315	37086420	three_prime_	ACACA	acetyl-CoA carboxylase alpha	mRNA
chr3	47852093	47852236	three_prime_	MAP4	microtubule associated protein 4	mRNA
chr17	759832	759926	three_prime_	GLOD4	glyoxalase domain containing 4	mRNA
chr17	35266116	35266162	three_prime_	SLFN5	schlafen family member 5	mRNA
chr3	49359959	49360096	three_prime_	RHOA	ras homolog family member A	mRNA
chr9	34087834	34087939	three_prime_	DCAF12	DDB1 and CUL4 associated factor 12	mRNA
chr22	45600839	45600983	three_prime_	FBLN1	fibulin 1	mRNA
chr19	53874496	53874627	three_prime_	MYADM	myeloid associated differentiation marker	mRNA

chr3	14486915	14486976	three_prime_	SLC6A6	solute carrier family 6 member 6	mRNA
chr19	35658593	35658673	three_prime_	COX6B1	cytochrome c oxidase subunit 6B1	mRNA
chr6	75255641	75255788	three_prime_	TMEM30A	transmembrane protein 30A	mRNA
chr11	121633103	121633249	three_prime_	SORL1	sortilin related receptor 1	mRNA
chr1	207793646	207793793	three_prime_	CD46	CD46 molecule	mRNA
chr3	177025061	177025138	three_prime_	TBL1XR1	transducin beta like 1 X-linked receptor 1	mRNA
chr3	177025164	177025205	three_prime_	TBL1XR1	transducin beta like 1 X-linked receptor 1	mRNA
chr17	47682459	47682550	three_prime_	KPNB1	karyopherin subunit beta 1	mRNA
chr16	18792568	18792621	three_prime_	ARL6IP1	ADP ribosylation factor like GTPase 6 interacting protein 1	mRNA
chr14	102050627	102050775	three_prime_	DYNC1H1	dynein cytoplasmic 1 heavy chain 1	mRNA
chr9	5785630	5785767	three_prime_	ERMP1	endoplasmic reticulum metallopeptidase 1	mRNA
chr19	48923153	48923268	three_prime_	NUCB1	nucleobindin 1	mRNA
chr2	9488618	9488708	three_prime_	IAH1	isoamyl acetate hydrolyzing esterase 1 (putative)	mRNA
chr1	162529161	162529232	three_prime_	UHMK1	U2AF homology motif kinase 1	mRNA
chr20	3805532	3805616	three_prime_	CDC25B	cell division cycle 25B	mRNA
chr10	5458030	5458166	three_prime_	NET1	neuroepithelial cell transforming 1	mRNA
chr17	5024230	5024354	three_prime_	KIF1C	kinesin family member 1C	mRNA
chr17	47682892	47683041	three_prime_	KPNB1	karyopherin subunit beta 1	mRNA
chr6	109367577	109367726	three_prime_	CD164	CD164 molecule	mRNA
chr8	142682527	142682615	three_prime_	PSCA	prostate stem cell antigen	mRNA
chr8	43122941	43122982	three_prime_	POMK	protein O-mannose kinase	mRNA
chr4	56933986	56934025	three_prime_	REST	RE1 silencing transcription factor	mRNA
chr9	92032289	92032412	three_prime_	SPTLC1	serine palmitoyltransferase long chain base subunit 1	mRNA
chr9	130139331	130139450	three_prime_	GPR107	G protein-coupled receptor 107	mRNA
chr1	154582094	154582153	three_prime_	ADAR	adenosine deaminase RNA specific	mRNA
chr17	50751354	50751504	three_prime_	LUC7L3	LUC7 like 3 pre-mRNA splicing factor	mRNA
chr12	57095800	57095943	three_prime_	STAT6	signal transducer and activator of transcription 6	mRNA
chr7	88279212	88279316	three_prime_	STEAP4	STEAP4 metalloreductase	mRNA
chr12	53040477	53040628	three_prime_	EIF4B	eukaryotic translation initiation factor 4B	mRNA
chr5	138934330	138934482	three_prime_	CTNNA1	catenin alpha 1	mRNA
chr17	42314563	42314571	three_prime_	STAT3	signal transducer and activator of transcription 3	mRNA
chr10	70879428	70879539	three_prime_	SGPL1	sphingosine-1-phosphate lyase 1	mRNA
chr4	99062667	99062783	three_prime_	METAP1	methionyl aminopeptidase 1	mRNA
chr16	18792911	18793017	three_prime_	ARL6IP1	ADP ribosylation factor like GTPase 6 interacting protein 1	mRNA
chr16	18793026	18793063	three_prime_	ARL6IP1	ADP ribosylation factor like GTPase 6 interacting protein 1	mRNA
chr10	87753188	87753308	three_prime_	ATAD1	ATPase family AAA domain containing 1	mRNA
chr17	35266313	35266466	three_prime_	SLFN5	schlafen family member 5	mRNA
chr4	105970119	105970173	three_prime_	NPNT	nephronectin	mRNA
chr4	105970186	105970235	three_prime_	NPNT	nephronectin	mRNA
chr9	121340174	121340235	three_prime_	STOM	stomatin	mRNA
chr3	197043326	197043381	three_prime_	DLG1	discs large MAGUK scaffold protein 1	mRNA
chr5	151504125	151504233	three_prime_	FAT2	FAT atypical cadherin 2	mRNA
chr1	1785995	1786044	three_prime_	GNB1	G protein subunit beta 1	mRNA
chr9	33112008	33112100	three_prime_	B4GALT1	beta-1,4-galactosyltransferase 1	mRNA
chr1	20652215	20652372	three_prime_	DDOST	dolichyl-diphosphooligosaccharide--protein glycosyltransferase non-catalytic subu	mRNA
chr1	31908756	31908913	three_prime_	PTP4A2	protein tyrosine phosphatase 4A2	mRNA
chr1	92390038	92390126	three_prime_	RPAP2	RNA polymerase II associated protein 2	mRNA
chr2	47386571	47386728	three_prime_	EPCAM	epithelial cell adhesion molecule	mRNA
chr11	62714889	62715003	three_prime_	HNRNPUL2	heterogeneous nuclear ribonucleoprotein U like 2	mRNA
chr12	130876191	130876259	three_prime_	RAN	RAN, member RAS oncogene family	mRNA
chr6	115939044	115939120	three_prime_	FRK	fyn-related Src family tyrosine kinase	mRNA
chr5	97032728	97032785	three_prime_	LNPEP	leucyl and cystinyl aminopeptidase	mRNA
chr14	105470511	105470541	three_prime_	MTA1	metastasis associated 1	mRNA
chr2	177222334	177222427	three_prime_	HNRNPA3	heterogeneous nuclear ribonucleoprotein A3	mRNA
chr6	122444756	122444916	three_prime_	SERINC1	serine incorporator 1	mRNA
chr5	97036288	97036411	three_prime_	LNPEP	leucyl and cystinyl aminopeptidase	mRNA
chr1	32043358	32043489	three_prime_	KHDRBS1	KH RNA binding domain containing, signal transduction associated 1	mRNA
chr15	40857287	40857422	three_prime_	SPINT1	serine peptidase inhibitor	mRNA
chr15	68207990	68208077	three_prime_	CLN6	CLN6 transmembrane ER protein	mRNA
chr1	183145344	183145475	three_prime_	LAMC1	laminin, gamma 1 (formerly LAMB2)	mRNA
chr6	99400161	99400240	three_prime_	PNISR	PNN interacting serine and arginine rich protein	mRNA

chr19	42378159	42378240	three_prime_	MEGF8	multiple EGF like domains 8	mRNA
chr8	27598022	27598077	three_prime_	CLU	clusterin	mRNA
chr6	160105775	160105852	three_prime_	IGF2R	insulin like growth factor 2 receptor	mRNA
chr5	175959608	175959772	three_prime_	THOC3	THO complex 3	mRNA
chr12	48936802	48936921	three_prime_	ARF3	ADP ribosylation factor 3	mRNA
chr8	97852048	97852201	three_prime_	LAPTM4B	lysosomal protein transmembrane 4 beta	mRNA
chr9	123103881	123103926	three_prime_	RABGAP1	RAB GTPase activating protein 1	mRNA
chr6	42103077	42103240	three_prime_	C6orf132	chromosome 6 open reading frame 132	mRNA
chr17	29261371	29261468	three_prime_	NUFIP2	nuclear FMR1 interacting protein 2	mRNA
chr3	49534264	49534430	three_prime_	DAG1	dystroglycan 1	mRNA
chr9	121340812	121340976	three_prime_	STOM	stomatin	mRNA
chr16	56362014	56362098	three_prime_	AMFR	autocrine motility factor receptor	mRNA
chr11	10307021	10307189	three_prime_	ADM	adrenomedullin	mRNA
chr18	9400393	9400561	three_prime_	TWSG1	twisted gastrulation BMP signaling modulator 1	mRNA
chr12	48937017	48937127	three_prime_	ARF3	ADP ribosylation factor 3	mRNA
chr1	183144273	183144443	three_prime_	LAMC1	laminin, gamma 1 (formerly LAMB2)	mRNA
chr10	114434303	114434377	three_prime_	ABLIM1	actin binding LIM protein 1	mRNA
chr1	167788236	167788302	three_prime_	MPZL1	myelin protein zero like 1	mRNA
chr1	167788311	167788377	three_prime_	MPZL1	myelin protein zero like 1	mRNA
chr2	20250342	20250390	three_prime_	PUM2	pumilio RNA binding family member 2	mRNA
chr5	79325840	79325902	three_prime_	JMY	junction mediating and regulatory protein, p53 cofactor	mRNA
chr7	44834271	44834360	three_prime_	H2AZ2	H2A.Z variant histone 2	mRNA
chr2	128190780	128190951	three_prime_	UGGT1	UDP-glucose glycoprotein glucosyltransferase 1	mRNA
chr1	207794861	207795029	three_prime_	CD46	CD46 molecule	mRNA
chr12	6237762	6237920	three_prime_	CD9	CD9 molecule	mRNA
chr12	1787780	1787953	three_prime_	ADIPOR2	adiponectin receptor 2	mRNA
chr2	86777291	86777440	three_prime_	RMND5A	required for meiotic nuclear division 5 homolog A	mRNA
chr3	108047127	108047292	three_prime_	CD47	CD47 molecule	mRNA
chr8	30678701	30678801	three_prime_	GSR	glutathione-disulfide reductase	mRNA
chr17	50751097	50751173	three_prime_	LUC7L3	LUC7 like 3 pre-mRNA splicing factor	mRNA
chr3	108046054	108046099	three_prime_	CD47	CD47 molecule	mRNA
chr3	49534191	49534262	three_prime_	DAG1	dystroglycan 1	mRNA
chr5	140706172	140706311	three_prime_	ZMAT2	zinc finger matrin-type 2	mRNA
chr6	30493572	30493689	three_prime_	HLA-E	major histocompatibility complex, class I, E	mRNA
chr1	6186293	6186470	three_prime_	RPL22	ribosomal protein L22	mRNA
chr2	218254115	218254155	three_prime_	ARPC2	actin related protein 2/3 complex subunit 2	mRNA
chr15	77046057	77046206	three_prime_	TSPAN3	tetraspanin 3	mRNA
chr5	151663420	151663572	three_prime_	SPARC	secreted protein acidic and cysteine rich	mRNA
chr9	83969217	83969396	three_prime_	HNRNPK	heterogeneous nuclear ribonucleoprotein K	mRNA
chr1	53246893	53247056	three_prime_	LRP8	LDL receptor related protein 8	mRNA
chr8	22005431	22005611	three_prime_	XPO7	exportin 7	mRNA
chr17	4733751	4733872	three_prime_	CXCL16	C-X-C motif chemokine ligand 16	mRNA
chr12	49763501	49763681	three_prime_	TMBIM6	transmembrane BAX inhibitor motif containing 7	mRNA
chr8	116846005	116846069	three_prime_	RAD21	RAD21 cohesin complex component	mRNA
chr8	116846078	116846144	three_prime_	RAD21	RAD21 cohesin complex component	mRNA
chr11	119664486	119664564	three_prime_	NECTIN1	nectin cell adhesion molecule 1	mRNA
chr3	31637420	31637462	three_prime_	STT3B	STT3 oligosaccharyltransferase complex catalytic subunit B	mRNA
chr11	130144427	130144584	three_prime_	APLP2	amyloid beta precursor like protein 2	mRNA
chr1	205715444	205715611	three_prime_	NUCKS1	nuclear casein kinase and cyclin-dependent kinase substrate 1	mRNA
chr5	151662611	151662795	three_prime_	SPARC	secreted protein acidic and cysteine rich	mRNA
chr1	169131742	169131879	three_prime_	ATP1B1	ATPase Na+/K+ transporting subunit beta 1	mRNA
chr19	38730173	38730323	three_prime_	ACTN4	actinin alpha 4	mRNA
chr5	179614538	179614723	three_prime_	HNRNPH1	heterogeneous nuclear ribonucleoprotein H1	mRNA
chr2	65269345	65269395	three_prime_	ACTR2	actin related protein 2	mRNA
chr1	40073334	40073439	three_prime_	PPT1	palmitoyl-protein thioesterase 1	mRNA
chr1	1785392	1785454	three_prime_	GNB1	G protein subunit beta 1	mRNA
chr1	35715234	35715359	three_prime_	C1orf216	chromosome 1 open reading frame 216	mRNA
chr16	87830558	87830574	three_prime_	SLC7A5	solute carrier family 7 member 5	mRNA
chr2	20202187	20202295	three_prime_	SDC1	syndecan 1	mRNA
chr7	90414389	90414573	three_prime_	CLDN12	claudin 12	mRNA
chr8	125021916	125022085	three_prime_	SQLE	squalene epoxidase	mRNA

chr6	33203908	33204095	three_prime_	SLC39A7	solute carrier family 39 member 7	mRNA
chr6	30626108	30626271	three_prime_	MRPS18B	mitochondrial ribosomal protein S18B	mRNA
chr8	97851933	97851967	three_prime_	LAPTM4B	lysosomal protein transmembrane 4 beta	mRNA
chr8	97851968	97852032	three_prime_	LAPTM4B	lysosomal protein transmembrane 4 beta	mRNA
chr2	138571924	138572109	three_prime_	SPOPL	speckle type BTB/POZ protein like	mRNA
chr6	43181267	43181298	three_prime_	SRF	serum response factor (c-fos serum response element-binding transcription factor)	mRNA
chr2	233772975	233773165	three_prime_	UGT1A8	UDP glucuronosyltransferase 1 family, polypeptide A8	mRNA
chr3	194403688	194403866	three_prime_	ATP13A3	ATPase 13A3	mRNA
chr20	49634157	49634347	three_prime_	B4GALT5	UDP-Gal:betaGlcNAc beta 1,4- galactosyltransferase, polypeptide 5	mRNA
chr17	48059835	48060026	three_prime_	NFE2L1	nuclear factor, erythroid 2 like 1	mRNA
chr6	82366370	82366478	three_prime_	TPBG	trophoblast glycoprotein	mRNA
chr20	45326114	45326297	three_prime_	SDC4	syndecan 4	mRNA
chr18	31548623	31548716	three_prime_	DSG2	desmoglein 2	mRNA
chr17	57680437	57680528	three_prime_	MSI2	musashi RNA binding protein 2	mRNA
chr10	96521193	96521321	three_prime_	TM9SF3	transmembrane 9 superfamily member 3	mRNA
chr11	102398424	102398619	three_prime_	TMEM123	transmembrane protein 123	mRNA
chr13	29512318	29512460	three_prime_	SLC7A1	solute carrier family 7 member 1	mRNA
chr1	119913625	119913722	three_prime_	NOTCH2	notch receptor 2	mRNA
chr9	111933600	111933648	three_prime_	UGCG	UDP-glucose ceramide glucosyltransferase	mRNA
chr16	18793070	18793124	three_prime_	ARL6IP1	ADP ribosylation factor like GTPase 6 interacting protein 1	mRNA
chr16	18793126	18793248	three_prime_	ARL6IP1	ADP ribosylation factor like GTPase 6 interacting protein 1	mRNA
chr5	6671320	6671417	three_prime_	SRD5A1	steroid 5 alpha-reductase 1	mRNA
chr2	26395387	26395482	three_prime_	SELENOI	selenoprotein I	mRNA
chr2	200823394	200823541	three_prime_	BZW1	basic leucine zipper and W2 domains 1	mRNA
chr4	105970432	105970560	three_prime_	NPNT	nephronectin	mRNA
chr4	105970562	105970631	three_prime_	NPNT	nephronectin	mRNA
chr20	32332651	32332754	three_prime_	KIF3B	kinesin family member 3B	mRNA
chr19	54461820	54461992	three_prime_	LENG8	leukocyte receptor cluster member 8	mRNA
chr1	159918349	159918510	three_prime_	TAGLN2	transgelin 2	mRNA
chr2	208236513	208236704	three_prime_	IDH1	isocitrate dehydrogenase (NADP(+)) 1	mRNA
chr6	106971336	106971533	three_prime_	CD24	CD24 molecule	mRNA
chr17	1345298	1345499	three_prime_	YWHAE	tyrosine 3-monooxygenase/tryptophan 5-monooxygenase activation protein epsilo	mRNA
chr18	3458116	3458275	three_prime_	TGIF1	TGFB induced factor homeobox 1	mRNA
chr22	36266405	36266606	three_prime_	APOL1	apolipoprotein L1	mRNA
chr12	88198204	88198258	three_prime_	TMTC3	transmembrane O-mannosyltransferase targeting cadherins 3	mRNA
chr12	88198262	88198356	three_prime_	TMTC3	transmembrane O-mannosyltransferase targeting cadherins 3	mRNA
chr2	85544933	85545018	three_prime_	MAT2A	methionine adenosyltransferase 2A	mRNA
chr15	98959655	98959767	three_prime_	IGF1R	insulin like growth factor 1 receptor	mRNA
chr17	40457482	40457687	three_prime_	IGFBP4	insulin-like growth factor binding protein 4	mRNA
chr9	129739076	129739256	three_prime_	PTGES	prostaglandin E synthase	mRNA
chr17	57006172	57006376	three_prime_	SCPEP1	serine carboxypeptidase 1	mRNA
chr1	225497342	225497425	three_prime_	ENAH	ENAH actin regulator	mRNA
chr6	34245810	34245957	three_prime_	HMGA1	high mobility group AT-hook 1	mRNA
chr2	201380401	201380516	three_prime_	TRAK2	trafficking kinesin protein 2	mRNA
chr9	93565865	93566073	three_prime_	FAM120A	family with sequence similarity 120A	mRNA
chr17	40457259	40457449	three_prime_	IGFBP4	insulin-like growth factor binding protein 4	mRNA
chr17	40457450	40457468	three_prime_	IGFBP4	insulin-like growth factor binding protein 4	mRNA
chr1	205715018	205715096	three_prime_	NUCKS1	nuclear casein kinase and cyclin-dependent kinase substrate 1	mRNA
chr17	5024627	5024716	three_prime_	KIF1C	kinesin family member 1C	mRNA
chr1	209786167	209786325	three_prime_	IRF6	interferon regulatory factor 6	mRNA
chr11	64823848	64824016	three_prime_	CDC42BPG	CDC42 binding protein kinase gamma (DMPK-like)	mRNA
chr5	10434263	10434357	three_prime_	MARCHF6	membrane associated ring-CH-type finger 6	mRNA
chr12	56597506	56597601	three_prime_	BAZ2A	bromodomain adjacent to zinc finger domain 2A	mRNA
chr12	56597603	56597718	three_prime_	BAZ2A	bromodomain adjacent to zinc finger domain 2A	mRNA
chr1	28147534	28147669	three_prime_	PTAFR	platelet activating factor receptor	mRNA
chr12	48938464	48938568	three_prime_	ARF3	ADP ribosylation factor 3	mRNA
chr12	48938570	48938676	three_prime_	ARF3	ADP ribosylation factor 3	mRNA
chr1	227919700	227919888	three_prime_	WNT9A	wingless-type MMTV integration site family, member 9A	mRNA
chr1	116988839	116988900	three_prime_	PTGFRN	prostaglandin F2 receptor inhibitor	mRNA
chr15	60347338	60347492	three_prime_	ANXA2	annexin A2	mRNA
chr15	60347496	60347552	three_prime_	ANXA2	annexin A2	mRNA

chr8	97852241	97852337	three_prime_	LAPTM4B	lysosomal protein transmembrane 4 beta	mRNA
chr8	97852361	97852410	three_prime_	LAPTM4B	lysosomal protein transmembrane 4 beta	mRNA
chr12	113390224	113390366	three_prime_	PLBD2	phospholipase B domain containing 2	mRNA
chrX	147949571	147949618	three_prime_	FMR1	FMRP translational regulator 1	mRNA
chr9	111933295	111933510	three_prime_	UGCG	UDP-glucose ceramide glucosyltransferase	mRNA
chr9	5785890	5786106	three_prime_	ERMP1	endoplasmic reticulum metallopeptidase 1	mRNA
chr12	53446279	53446357	three_prime_	PRR13	proline rich 13	mRNA
chr22	41907087	41907280	three_prime_	SREBF2	sterol regulatory element binding transcription factor 2	mRNA
chr1	207359712	207359930	three_prime_	CD55	CD55 molecule	mRNA
chr14	21210938	21211157	three_prime_	HNRNPC	heterogeneous nuclear ribonucleoprotein C	mRNA
chr17	29262813	29262899	three_prime_	NUFIP2	nuclear FMR1 interacting protein 2	mRNA
chr17	29262903	29262968	three_prime_	NUFIP2	nuclear FMR1 interacting protein 2	mRNA
chr19	582774	582993	three_prime_	BSG	basigin (Ok blood group)	mRNA
chr16	71928745	71928803	three_prime_	IST1	IST1 factor associated with ESCRT-III	mRNA
chr5	1462139	1462202	three_prime_	LPCAT1	lysophosphatidylcholine acyltransferase 1	mRNA
chr17	38919803	38919943	three_prime_	LASP1	LIM and SH3 protein 1	mRNA
chr15	98961403	98961440	three_prime_	IGF1R	insulin like growth factor 1 receptor	mRNA
chr14	69714553	69714661	three_prime_	SUSD6	sushi domain containing 6	mRNA
chrX	153696005	153696160	three_prime_	SLC6A8	solute carrier family 6 member 8	mRNA
chr2	47160379	47160539	three_prime_	CALM2	calmodulin 2	mRNA
chr2	131051855	131051947	three_prime_	FAM168B	family with sequence similarity 168 member B	mRNA
chr14	64744153	64744206	three_prime_	PLEKHG3	pleckstrin homology and RhoGEF domain containing G3	mRNA
chr1	171592262	171592362	three_prime_	PRRC2C	proline rich coiled-coil 2C	mRNA
chr21	25880955	25881059	three_prime_	APP	amyloid beta precursor protein	mRNA
chr21	25881060	25881160	three_prime_	APP	amyloid beta precursor protein	mRNA
chr14	70371170	70371366	three_prime_	SYNJ2BP-COX16	SYNJ2BP-COX16 readthrough	mRNA
chr6	106970159	106970308	three_prime_	CD24	CD24 molecule	mRNA
chr21	25880867	25880908	three_prime_	APP	amyloid beta precursor protein	mRNA
chr3	160501478	160501708	three_prime_	KPNA4	karyopherin subunit alpha 4	mRNA
chr17	59840463	59840561	three_prime_	VMP1	vacuole membrane protein 1	mRNA
chr19	48197350	48197481	three_prime_	ZSWIM9	zinc finger SWIM-type containing 9	mRNA
chr16	66723203	66723370	three_prime_	DYNC1LI2	dynein cytoplasmic 1 light intermediate chain 2	mRNA
chr11	110230506	110230688	three_prime_	RDX	radixin, transcript variant 4	mRNA
chr1	209785855	209785909	three_prime_	IRF6	interferon regulatory factor 6	mRNA
chr1	119913318	119913491	three_prime_	NOTCH2	notch receptor 2	mRNA
chrX	41348613	41348762	three_prime_	DDX3X	DEAD-box helicase 3 X-linked	mRNA
chr12	53041270	53041420	three_prime_	EIF4B	eukaryotic translation initiation factor 4B	mRNA
chr16	56362420	56362506	three_prime_	AMFR	autocrine motility factor receptor	mRNA
chr16	18791926	18792143	three_prime_	ARL6IP1	ADP ribosylation factor like GTPase 6 interacting protein 1	mRNA
chr6	30899889	30900129	three_prime_	DDR1	discoidin domain receptor tyrosine kinase 1	mRNA
chr11	58618503	58618636	three_prime_	ZFP91	ZFP91 zinc finger protein	mRNA
chr22	41663803	41664038	three_prime_	XRCC6	X-ray repair cross complementing 6	mRNA
chr6	31817748	31817837	three_prime_	HSPA1A	heat shock 70kDa protein 1A	mRNA
chr10	100362423	100362627	three_prime_	SCD	stearoyl-CoA desaturase (delta-9-desaturase)	mRNA
chr6	107871577	107871717	three_prime_	SEC63	SEC63 homolog, protein translocation regulator	mRNA
chr17	35725561	35725665	three_prime_	AP2B1	adaptor related protein complex 2 subunit beta 1	mRNA
chr13	113264490	113264627	three_prime_	CUL4A	cullin 4A	mRNA
chr5	141516341	141516565	three_prime_	DIAPH1	diaphanous related formin 1	mRNA
chr1	42178162	42178266	three_prime_	FOXJ3	forkhead box J3	mRNA
chr5	171410621	171410683	three_prime_	NPM1	nucleophosmin 1	mRNA
chr5	171410761	171410869	three_prime_	NPM1	nucleophosmin 1	mRNA
chr1	112913654	112913820	three_prime_	SLC16A1	solute carrier family 16 member 1	mRNA
chr6	109368075	109368308	three_prime_	CD164	CD164 molecule	mRNA
chr10	71299955	71300183	three_prime_	UNC5B	unc-5 netrin receptor B	mRNA
chr5	159158106	159158176	three_prime_	RNF145	ring finger protein 145	mRNA
chr5	159158195	159158356	three_prime_	RNF145	ring finger protein 145	mRNA
chr14	61278932	61278997	three_prime_	TMEM30B	transmembrane protein 30B	mRNA
chr14	61279107	61279180	three_prime_	TMEM30B	transmembrane protein 30B	mRNA
chr7	106091017	106091220	three_prime_	SYPL1	synaptophysin like 1	mRNA
chr15	72199028	72199099	three_prime_	PKM	pyruvate kinase M1/2	mRNA
chr1	42177638	42177842	three_prime_	FOXJ3	forkhead box J3	mRNA

chr19	38292205	38292366	three_prime_	SPINT2	serine peptidase inhibitor, Kunitz type 2	mRNA
chr19	38292371	38292414	three_prime_	SPINT2	serine peptidase inhibitor, Kunitz type 2	mRNA
chr14	75134354	75134607	three_prime_	TMED10	transmembrane p24 trafficking protein 10	mRNA
chr3	196829487	196829741	three_prime_	PAK2	p21 (RAC1) activated kinase 2	mRNA
chr1	10419689	10419847	three_prime_	PGD	phosphogluconate dehydrogenase	mRNA
chr20	49634972	49635147	three_prime_	B4GALT5	UDP-Gal:betaGlcNAc beta 1,4- galactosyltransferase, polypeptide 5	mRNA
chr20	49635150	49635199	three_prime_	B4GALT5	UDP-Gal:betaGlcNAc beta 1,4- galactosyltransferase, polypeptide 5	mRNA
chr2	230818841	230818915	three_prime_	CAB39	calcium binding protein 39	mRNA
chr2	230818921	230819098	three_prime_	CAB39	calcium binding protein 39	mRNA
chr11	71434873	71435133	three_prime_	DHCR7	7-dehydrocholesterol reductase	mRNA
chr16	1701024	1701184	three_prime_	JPT2	Jupiter microtubule associated homolog 2	mRNA
chr1	207794623	207794753	three_prime_	CD46	CD46 molecule	mRNA
chr19	42377801	42377980	three_prime_	MEGF8	multiple EGF like domains 8	mRNA
chr16	19119112	19119375	three_prime_	ITPRIPL2	inositol 1,4,5-trisphosphate receptor interacting protein-like 2	mRNA
chr3	152464729	152464862	three_prime_	MBNL1	muscleblind like splicing regulator 1	mRNA
chr19	1272581	1272754	three_prime_	CIRBP	cold inducible RNA binding protein	mRNA
chr1	203740129	203740222	three_prime_	ATP2B4	ATPase plasma membrane Ca2+ transporting 4	mRNA
chr1	203740227	203740328	three_prime_	ATP2B4	ATPase plasma membrane Ca2+ transporting 4	mRNA
chr1	167789312	167789404	three_prime_	MPZL1	myelin protein zero like 1	mRNA
chr1	167789461	167789556	three_prime_	MPZL1	myelin protein zero like 1	mRNA
chr2	10783908	10784118	three_prime_	PDIA6	protein disulfide isomerase family A member 6	mRNA
chr20	3805709	3805936	three_prime_	CDC25B	cell division cycle 25B	mRNA
chr1	205714570	205714734	three_prime_	NUCKS1	nuclear casein kinase and cyclin-dependent kinase substrate 1	mRNA
chr10	87754440	87754687	three_prime_	ATAD1	ATPase family AAA domain containing 1	mRNA
chr19	46774908	46775179	three_prime_	SLC1A5	solute carrier family 1 member 5	mRNA
chr12	12916963	12917236	three_prime_	GPRC5A	G protein-coupled receptor, class C, group 5, member A	mRNA
chr1	1055393	1055646	three_prime_	AGRN	agrin	mRNA
chr10	71300346	71300474	three_prime_	UNC5B	unc-5 netrin receptor B	mRNA
chr8	119244794	119245048	three_prime_	MAL2	mal, T cell differentiation protein 2	mRNA
chr11	69651751	69651925	three_prime_	CCND1	cyclin D1	mRNA
chr9	112221641	112221716	three_prime_	PTBP3	polypyrimidine tract binding protein 3	mRNA
chr9	112221719	112221869	three_prime_	PTBP3	polypyrimidine tract binding protein 3	mRNA
chr12	125029781	125029937	three_prime_	BRI3BP	BRI3 binding protein	mRNA
chr11	1752754	1753035	three_prime_	CTSD	cathepsin D	mRNA
chr20	51600691	51600740	three_prime_	ATP9A	ATPase phospholipid transporting 9A	mRNA
chr20	51600755	51600879	three_prime_	ATP9A	ATPase phospholipid transporting 9A	mRNA
chr1	154584452	154584486	three_prime_	ADAR	adenosine deaminase RNA specific	mRNA
chr8	123014521	123014755	three_prime_	DERL1	derlin 1	mRNA
chr6	117707146	117707224	three_prime_	NUS1	nuclear undecaprenyl pyrophosphate synthase 1 homolog (S. cerevisiae)	mRNA
chr6	117707227	117707265	three_prime_	NUS1	nuclear undecaprenyl pyrophosphate synthase 1 homolog (S. cerevisiae)	mRNA
chr3	41239653	41239736	three_prime_	CTNNB1	catenin beta 1	mRNA
chr3	41239794	41239916	three_prime_	CTNNB1	catenin beta 1	mRNA
chr11	62624831	62624903	three_prime_	GANAB	glucosidase II alpha subunit	mRNA
chr12	12914239	12914465	three_prime_	GPRC5A	G protein-coupled receptor, class C, group 5, member A	mRNA
chr17	58089788	58089934	three_prime_	DYNLL2	dynein, light chain, LC8-type 2	mRNA
chr17	58089935	58089996	three_prime_	DYNLL2	dynein, light chain, LC8-type 2	mRNA
chr15	98963364	98963523	three_prime_	IGF1R	insulin like growth factor 1 receptor	mRNA
chr9	129739261	129739554	three_prime_	PTGES	prostaglandin E synthase	mRNA
chr9	128695190	128695305	three_prime_	SET	SET nuclear proto-oncogene	mRNA
chr11	1753177	1753449	three_prime_	CTSD	cathepsin D	mRNA
chr17	1344791	1345028	three_prime_	YWHAE	tyrosine 3-monooxygenase/tryptophan 5-monooxygenase activation protein epsilo	mRNA
chr1	207360131	207360299	three_prime_	CD55	CD55 molecule	mRNA
chr15	64156047	64156145	three_prime_	PPIB	peptidylprolyl isomerase B (cyclophilin B)	mRNA
chr1	26864158	26864456	three_prime_	SFN	stratifin	mRNA
chr11	2396940	2397067	three_prime_	CD81	CD81 molecule	mRNA
chr14	103136378	103136501	three_prime_	TNFAIP2	TNF alpha induced protein 2	mRNA
chr2	85320914	85321223	three_prime_	TGOLN2	trans-golgi network protein 2	mRNA
chr17	38921477	38921744	three_prime_	LASP1	LIM and SH3 protein 1	mRNA
chr6	36601961	36602272	three_prime_	SRSF3	serine and arginine rich splicing factor 3	mRNA
chr12	49764431	49764610	three_prime_	TMBIM6	transmembrane BAX inhibitor motif containing 6	mRNA
chr12	49764618	49764721	three_prime_	TMBIM6	transmembrane BAX inhibitor motif containing 6	mRNA

chrX	16844835	16845087	three_prime_	RBBP7	RB binding protein 7, chromatin remodeling factor	mRNA
chr12	108645265	108645317	three_prime_	CORO1C	coronin 1C	mRNA
chr12	108645329	108645424	three_prime_	CORO1C	coronin 1C	mRNA
chr12	108645554	108645572	three_prime_	CORO1C	coronin 1C	mRNA
chr9	128694822	128695139	three_prime_	SET	SET nuclear proto-oncogene	mRNA
chr3	49534632	49534730	three_prime_	DAG1	dystroglycan 1	mRNA
chr19	35123440	35123619	three_prime_	FXYD3	FXYD domain containing ion transport regulator 3	mRNA
chr14	75132584	75132655	three_prime_	TMED10	transmembrane p24 trafficking protein 10	mRNA
chr14	75132658	75132831	three_prime_	TMED10	transmembrane p24 trafficking protein 10	mRNA
chr1	154206946	154207114	three_prime_	C1orf43	chromosome 1 open reading frame 43	mRNA
chr10	72275138	72275466	three_prime_	DDIT4	DNA-damage-inducible transcript 4	mRNA
chr14	67677026	67677116	three_prime_	RDH11	retinol dehydrogenase 11	mRNA
chr3	48467886	48467994	three_prime_	SHISA5	shisa family member 5	mRNA
chr3	48468079	48468217	three_prime_	SHISA5	shisa family member 5	mRNA
chr19	38291927	38292059	three_prime_	SPINT2	serine peptidase inhibitor, Kunitz type 2	mRNA
chr19	38292062	38292156	three_prime_	SPINT2	serine peptidase inhibitor, Kunitz type 2	mRNA
chr3	105574715	105574836	three_prime_	ALCAM	activated leukocyte cell adhesion molecule	mRNA
chr1	160997625	160997963	three_prime_	F11R	F11 receptor	mRNA
chr8	100918956	100919246	three_prime_	YWHAZ	tyrosine 3-monooxygenase/tryptophan 5-monooxygenase activation protein zeta	mRNA
chr15	78897048	78897220	three_prime_	MORF4L1	mortality factor 4 like 1	mRNA
chr15	78897267	78897328	three_prime_	MORF4L1	mortality factor 4 like 1	mRNA
chr6	122444259	122444570	three_prime_	SERINC1	serine incorporator 1	mRNA
chr5	179728590	179728928	three_prime_	CANX	calnexin	mRNA
chr2	20032707	20032756	three_prime_	LAPTM4A	lysosomal protein transmembrane 4 alpha	mRNA
chr2	20032760	20033024	three_prime_	LAPTM4A	lysosomal protein transmembrane 4 alpha	mRNA
chr16	87830024	87830195	three_prime_	SLC7A5	solute carrier family 7 member 5	mRNA
chr16	87830218	87830374	three_prime_	SLC7A5	solute carrier family 7 member 5	mRNA
chr1	160996232	160996523	three_prime_	F11R	F11 receptor	mRNA
chr1	116404440	116404749	three_prime_	ATP1A1	ATPase Na+/K+ transporting subunit alpha 1	mRNA
chr3	194405089	194405214	three_prime_	ATP13A3	ATPase 13A3	mRNA
chr2	231454951	231455004	three_prime_	NCL	nucleolin	mRNA
chr7	129832390	129832634	three_prime_	UBE2H	ubiquitin conjugating enzyme E2 H	mRNA
chr16	2771149	2771306	three_prime_	SRRM2	serine/arginine repetitive matrix 2	mRNA
chr13	21147645	21147717	three_prime_	SAP18	Sin3A associated protein 18	mRNA
chr13	21147722	21147789	three_prime_	SAP18	Sin3A associated protein 18	mRNA
chr5	43290826	43291089	three_prime_	HMGCS1	3-hydroxy-3-methylglutaryl-CoA synthase 1	mRNA
chr12	12916594	12916793	three_prime_	GPRC5A	G protein-coupled receptor, class C, group 5, member A	mRNA
chr12	12916794	12916939	three_prime_	GPRC5A	G protein-coupled receptor, class C, group 5, member A	mRNA
chr8	119245265	119245390	three_prime_	MAL2	mal, T cell differentiation protein 2	mRNA
chr8	119245393	119245652	three_prime_	MAL2	mal, T cell differentiation protein 2	mRNA
chr7	45912383	45912576	three_prime_	IGFBP3	insulin like growth factor binding protein 3	mRNA
chr7	45912608	45912771	three_prime_	IGFBP3	insulin like growth factor binding protein 3	mRNA
chr1	205715664	205715958	three_prime_	NUCKS1	nuclear casein kinase and cyclin-dependent kinase substrate 1	mRNA
chr6	44253488	44253843	three_prime_	HSP90AB1	heat shock protein 90 alpha family class B member 1	mRNA
chr17	41513764	41514125	three_prime_	KRT15	keratin 15	mRNA
chr2	85544544	85544725	three_prime_	MAT2A	methionine adenosyltransferase 2A	mRNA
chr11	71434470	71434714	three_prime_	DHCR7	7-dehydrocholesterol reductase	mRNA
chr11	71434817	71434872	three_prime_	DHCR7	7-dehydrocholesterol reductase	mRNA
chr15	98958600	98958979	three_prime_	IGF1R	insulin like growth factor 1 receptor	mRNA
chr1	153981820	153981938	three_prime_	RAB13	RAB13, member RAS oncogene family	mRNA
chr1	153981939	153982078	three_prime_	RAB13	RAB13, member RAS oncogene family	mRNA
chr1	209787225	209787488	three_prime_	IRF6	interferon regulatory factor 6	mRNA
chr1	45511036	45511167	three_prime_	PRDX1	peroxiredoxin 1	mRNA
chr1	45511173	45511414	three_prime_	PRDX1	peroxiredoxin 1	mRNA
chr12	49762872	49763049	three_prime_	TMBIM6	transmembrane BAX inhibitor motif containing 6	mRNA
chr12	49763057	49763174	three_prime_	TMBIM6	transmembrane BAX inhibitor motif containing 6	mRNA
chr2	241352202	241352330	three_prime_	SEPTIN2	septin 2	mRNA
chr15	98961229	98961402	three_prime_	IGF1R	insulin like growth factor 1 receptor	mRNA
chr1	156742129	156742524	three_prime_	HDGF	heparin binding growth factor	mRNA
chr11	102231071	102231232	three_prime_	YAP1	Yes associated protein 1	mRNA
chr1	8861005	8861429	three_prime_	ENO1	enolase 1	mRNA

chr12	48936217	48936265	three_prime_	ARF3	ADP ribosylation factor 3	mRNA
chr12	48936294	48936423	three_prime_	ARF3	ADP ribosylation factor 3	mRNA
chr19	583064	583082	three_prime_	BSG	basigin (Ok blood group)	mRNA
chr14	102081399	102081821	three_prime_	HSP90AA1	heat shock protein 90 alpha family class A member 1	mRNA
chr8	97851472	97851593	three_prime_	LAPTM4B	lysosomal protein transmembrane 4 beta	mRNA
chr8	97851599	97851831	three_prime_	LAPTM4B	lysosomal protein transmembrane 4 beta	mRNA
chr5	151663074	151663177	three_prime_	SPARC	secreted protein acidic and cysteine rich	mRNA
chr5	151663180	151663352	three_prime_	SPARC	secreted protein acidic and cysteine rich	mRNA
chr5	151663377	151663419	three_prime_	SPARC	secreted protein acidic and cysteine rich	mRNA
chr1	161072130	161072569	three_prime_	NECTIN4	nectin cell adhesion molecule 4	mRNA
chr9	130135427	130135605	three_prime_	GPR107	G protein-coupled receptor 107	mRNA
chr9	130135779	130135880	three_prime_	GPR107	G protein-coupled receptor 107	mRNA
chr1	54851750	54852055	three_prime_	DHCR24	24-dehydrocholesterol reductase	mRNA
chr12	12913264	12913619	three_prime_	GPRC5A	G protein-coupled receptor, class C, group 5, member A	mRNA
chr1	54850883	54851273	three_prime_	DHCR24	24-dehydrocholesterol reductase	mRNA
chr1	160998147	160998350	three_prime_	F11R	F11 receptor	mRNA
chr1	160998354	160998630	three_prime_	F11R	F11 receptor	mRNA
chr17	59839906	59840184	three_prime_	VMP1	vacuole membrane protein 1	mRNA
chr14	102081031	102081398	three_prime_	HSP90AA1	heat shock protein 90 alpha family class A member 1	mRNA
chr11	18407320	18407826	three_prime_	LDHA	lactate dehydrogenase A	mRNA
chr12	6346817	6347300	three_prime_	SCNN1A	sodium channel epithelial 1 alpha subunit	mRNA
chr11	57817005	57817345	three_prime_	CTNND1	catenin delta 1	mRNA
chr3	195746895	195747207	three_prime_	MUC4	mucin 4, cell surface associated	mRNA
chr3	195747235	195747380	three_prime_	MUC4	mucin 4, cell surface associated	mRNA
chr7	74196253	74196339	three_prime_	EIF4H	eukaryotic translation initiation factor 4H	mRNA
chr16	68834529	68834670	three_prime_	CDH1	cadherin 1	mRNA
chr16	68834672	68834750	three_prime_	CDH1	cadherin 1	mRNA
chr16	68834764	68835011	three_prime_	CDH1	cadherin 1	mRNA
chr10	71816296	71816566	three_prime_	PSAP	prosaposin	mRNA
chr10	71816567	71816923	three_prime_	PSAP	prosaposin	mRNA
chr3	196049807	196050186	three_prime_	TFRC	transferrin receptor	mRNA
chr3	196050332	196050452	three_prime_	TFRC	transferrin receptor	mRNA
chr8	11844384	11844428	three_prime_	CTSB	cathepsin B	mRNA
chr8	11844429	11844637	three_prime_	CTSB	cathepsin B	mRNA
chr8	11844710	11844978	three_prime_	CTSB	cathepsin B	mRNA
chr8	11844991	11845059	three_prime_	CTSB	cathepsin B	mRNA
chr1	205713772	205713943	three_prime_	NUCKS1	nuclear casein kinase and cyclin-dependent kinase substrate 1	mRNA
chr1	205714103	205714217	three_prime_	NUCKS1	nuclear casein kinase and cyclin-dependent kinase substrate 1	mRNA
chr1	205714344	205714436	three_prime_	NUCKS1	nuclear casein kinase and cyclin-dependent kinase substrate 1	mRNA
chr20	37518009	37518190	three_prime_	BLCAP	BLCAP apoptosis inducing factor	mRNA
chr20	37518328	37518512	three_prime_	BLCAP	BLCAP apoptosis inducing factor	mRNA
chr11	102396665	102396968	three_prime_	TMEM123	transmembrane protein 123	mRNA
chr11	102396970	102397353	three_prime_	TMEM123	transmembrane protein 123	mRNA
chr11	102397354	102397485	three_prime_	TMEM123	transmembrane protein 123	mRNA
chr9	33441189	33441994	three_prime_	AQP3	aquaporin 3 (Gill blood group)	mRNA
chr7	45912779	45913715	three_prime_	IGFBP3	insulin like growth factor binding protein 3	mRNA
chr7	45913716	45913834	three_prime_	IGFBP3	insulin like growth factor binding protein 3	mRNA
chr13	110892942	110892959	three_prime_	ANKRD10	ankyrin repeat domain 10	mRNA
chr1	26877881	26877905	three_prime_	GPN2	GPN-loop GTPase 2	mRNA
chr19	44384808	44384834	three_prime_	ZNF285	zinc finger protein 285	mRNA
chr1	53084875	53084907	three_prime_	PODN	podocan	mRNA
chr13	36224964	36224980	three_prime_	CCDC169	coiled-coil domain containing 169	mRNA
chr1	161037684	161037733	three_prime_	TSTD1	thiosulfate sulfurtransferase like domain containing 1	mRNA
chr7	131502621	131502678	three_prime_	PODXL	podocalyxin like	mRNA
chr7	131502680	131502811	three_prime_	PODXL	podocalyxin like	mRNA
chr22	38483521	38483620	three_prime_	DDX17	DEAD-box helicase 17	mRNA
chr22	38485482	38485522	three_prime_	DDX17	DEAD-box helicase 17	mRNA
chr17	58004979	58005020	three_prime_	SRSF1	serine and arginine rich splicing factor 1	mRNA
chr17	58005038	58005220	three_prime_	SRSF1	serine and arginine rich splicing factor 1	mRNA
chr2	203427263	203427323	three_prime_	ABI2	abl interactor 2	mRNA
chr19	48197135	48197224	three_prime_	ZSWIM9	zinc finger SWIM-type containing 9	mRNA
