# Supplementary material for: Forming cytoplasmic stress granules PURα suppresses mRNA translation initiation of IGFBP3 to promote esophageal squamous cell carcinoma progression
Source: Oncogene. 2022 Aug 9;41(38):4336–48. doi: 10.1038/s41388-022-02426-3 (PMC9481463; doi:10.1038/s41388-022-02426-3)
Supplement: Supplementary file 3 — Supplementary Table S5 [file 41388_2022_2426_MOESM3_ESM.pdf]

PURα-interacting proteins

Protein ID	Gene name	Unique peptide	Score	Intensity	LFQ IgG	LFQ PURA	Ratio(PURA/IgG)
G3V311	PLEKHG3	4	32.224	73558000	0	10086000	100
P26640	VARS	1	6.1179	2971600	0	241050	100
P29372	MPG	3	17.64	33067000	0	17725000	100
P56192	MARS	1	12.402	5855000	0	1734200	100
P51610	HCFC1	1	8.369	6219200	0	504470	100
P54578	USP14	1	18.675	8729900	0	2191000	100
Q16540	MRPL23	2	13.779	17123000	0	7017200	100
A6NNP5	CCDC169	1	6.0578	132080000	0	2211300	100
Q9P2M7	CGN	3	27.78	36590000	0	2968000	100
Q8IXW7	FMR1	3	21.283	30929000	0	6773600	100
A8MT70	ZBBX	1	9.5805	228820000	0	28494000	100
Q8IYB3	SRRM1	5	81.588	108040000	0	8350900	100
Q9Y262	EIF3L	1	27.273	10004000	0	1787600	100
Q01844	EWSR1	3	31.383	52613000	0	15583000	100
O94905	ERLIN2	1	6.62	10861000	0	5822100	100
P12956	XRCC6	2	11.303	14753000	0	2593100	100
Q9BPZ7	MAPKAP1	1	6.8737	4588300	0	1359000	100
P49760	CLK2	2	13.127	20855000	0	5234100	100
Q9HB71	CACYBP	1	7.3171	10650000	0	8427000	100
Q92769	HDAC2	3	17.725	29801000	0	7479400	100
O00303	EIF3F	1	10.662	13603000	0	1102600	100
P55209	NAP1L1	1	20.063	49564000	0	12440000	100
P68431	H3C1	1	15.461	22977000	0	9416400	100
P27824	CANX	1	6.1697	5208600	0	1542700	100
O60583	CCNT2	3	34.044	21702000	0	6427900	100
O75569	PRKRA	1	6.6866	995770	0	533770	100
P36542	ATP5C1	3	17.196	2976500	0	1595500	100
P52815	MRPL12	3	19.047	19489000	0	7987200	100
Q9BY77	POLDIP3	3	27.283	26268000	0	6187600	100
O00472	ELL2	1	6.2258	3377800	0	1000500	100
Q9Y697	NFS1	1	26.714	62959000	0	5238800	100
P40227	CCT6A	4	24.447	46456000	0	11659000	100
P62191	PSMC1	3	23.974	29278000	0	6122300	100
Q96SB4	SRPK1	4	29.335	33267000	0	9143100	100
Q14103	HNRNPD	1	7.5842	56695000	0	4207800	100
P49368	CCT3	5	52.027	77228000	0	19383000	100
O60248	SOX15	1	9.3598	16712000	0	16463000	100
P61163	ACTR1A	2	29.356	20687000	0	2245900	100
Q9H078	CLPB	2	10.508	31036000	0	5834000	100
P51114	FXR1	6	72.236	86572000	0	21955000	100
O60812	HNRNPC	5	29.977	72611000	0	35143000	100
Q9H0D6	XRN2	4	24.432	30414000	0	9008300	100
P35606	COPB2	2	12.653	19676000	0	5827800	100
P29401	TKT	3	20.772	23527000	0	5904700	100
Q96ME7	ZNF512	1	12.124	21736000	0	5455200	100
P84103	SRSF3	2	11.918	88069000	0	59337000	100
Q8NEM7	SUPT20H	1	6.8153	8858400	0	2623800	100
P27105	STOM	2	10.734	13624000	0	7303100	100
Q9Y285	FARSA	1	6.0652	3183900	0	799090	100
Q15084	PDIA6	1	7	8428900	0	2115500	100
P62826	RAN	1	5.9917	10034000	0	9884900	100
P50991	CCT4	3	20.192	33218000	0	7020000	100
Q14151	SAFB2	2	43.1	19951000	0	1618300	100
Q6ZVM7	TOM1L2	2	25.616	21136000	0	4107900	100

P17174	GOT1	1	6.432	94889000	0	8874500	100
P22061	PCMT1	1	6.1587	2213500	0	2180600	100
Q9UBQ5	EIF3K	1	7.5824	4667700	0	4598300	100
P48643	CCT5	2	12.251	20672000	0	5188300	100
P61604	HSPE1	2	13.554	13305000	0	7877000	100
Q8IZL8	PELP1	2	29.334	40447000	0	3280900	100
Q9HAV0	GNB4	3	32.414	58989000	0	6982500	100
Q9BRJ6	C7orf50	3	24.315	28173000	0	23224000	100
Q9NX63	CHCHD3	4	29.761	36915000	0	33878000	100
Q9BWE0	REPIN1	1	7.3823	19486000	0	4890500	100
P35249	RFC4	2	12.752	19468000	0	10436000	100
P02538	KRT6A	1	46.672	303960000	0	75731000	100
P20930	FLG	1	7.9534	7671100	0	5745800	100
Q5D862	FLG2	4	54.647	53519000	0	14683000	100
Q8N1N4	KRT78	1	6.0117	8792900	0	1590100	100
Q7Z794	KRT77	1	13.893	17527000	0	3417300	100
P78344	EIF4G2	1	6.1125	7323100	0	2169000	100
P22234	PAICS	2	11.194	4104300	0	445580	100
Q8IWZ3	ANKHD1	1	61.791	547230000	0	58153000	100
O75122	CLASP2	2	15.951	9729400	0	2067900	100
P63104	YWHAZ	1	6.7236	11229000	0	8941600	100
Q15369	TCEB1	1	6.4135	6216900	0	10322000	100
P62888	RPL30	4	24.712	44944000	0	12818000	100
P63208	SKP1	1	6.5717	9416200	0	3859000	100
Q9P0K7	RAI14	4	93.337	79836000	0	7242000	100
Q96C57	CUSTOS	1	12.124	16356000	0	5958900	100
P50914	RPL14	1	6.7716	94932000	0	50259000	100
O60784	TOM1	2	23.84	5449900	0	851630	100
Q9Y520	PRRC2C	4	54.642	51296000	0	4160900	100
Q86VM9	ZC3H18	8	46.579	80265000	0	6599300	100
Q03001	DST	9	73.138	92335000	0	6981400	100
Q9BTC8	MTA3	2	12.831	17895000	0	4187100	100
Q15154	PCM1	2	20.806	25636000	0	1585600	100
P62847	RPS24	2	10.935	8658900	0	3097600	100
P68400	CSNK2A1	1	9.0469	5743700	0	623570	100
P78527	PRKDC	9	67.972	76261000	0	6185900	100
Q7Z5J4	RAI1	5	54.025	67873000	0	5505500	100
Q14203	DCTN1	1	6.6981	6762600	0	548550	100
E9PBL8	COG1	1	6.3801	22015000	0	1147400	100
Q5JNZ6	RBBP7	1	12.576	24084000	0	6044600	100
Q9P275	USP36	1	15.062	19331000	0	5273800	100
P27348	YWHAQ	1	6.7236	11066000	0	5872600	100
P28290	SSFA2	2	20.379	8557700	0	694150	100
P17980	PSMC3	2	17.122	11689000	0	2933800	100
P17480	UBTF	1	12.563	18451000	0	3661500	100
Q14444	CAPRIN1	3	21.173	57236000	0	16953000	100
P46776	RPL27A	4	27.705	438360000	0	179680000	100
Q9UHX1	PUF60	2	17.897	26049000	0	6537800	100
Q9NPD3	EXOSC4	2	12.794	10522000	0	10366000	100
O43159	RRP8	2	17.514	22292000	0	5594900	100
Q9Y4A5	TRRAP	1	8.1434	19949000	0	1122500	100
P78371	CCT2	6	66.03	70932000	0	13429000	100
Q9UJS0	SLC25A13	1	8.8467	12475000	0	3131000	100
Q66PJ3	ARL6IP4	2	11.665	4813600	0	1906700	100
P08195	SLC3A2	3	28.954	42390000	0	9208900	100
Q13523	PRPF4B	1	6.3118	11893000	0	964680	100

Q14152	EIF3A	4	23.56	17748000	0	1439600	100
Q5JTH9	RRP12	2	14.289	8414000	0	682500	100
Q9BSC4	NOL10	3	27.791	14577000	0	3479500	100
Q12931	TRAP1	2	16.199	7704800	0	2282100	100
P07237	P4HB	1	6.2758	8946100	0	1711200	100
P49790	NUP153	6	40.178	49100000	0	4420000	100
Q5T3P9	LGALS8	3	18.364	19017000	0	8980300	100
O14639	ABLIM1	2	15.183	25409000	0	5529800	100
Q9P031	CCDC59	3	19.038	25297000	0	13560000	100
P27708	CAD	2	13.977	16914000	0	1372000	100
Q14157	UBAP2L	2	10.774	13012000	0	1055400	100
P27635	RPL10	4	23.759	156710000	0	142550000	100
P46060	RANGAP1	3	25.424	38642000	0	11445000	100
P41091	EIF2S3	1	22.075	17896000	0	3541900	100
Q9Y6M1	IGF2BP2	1	7.1366	3948400	0	990980	100
O43663	PRC1	2	11.395	10713000	0	3173100	100
Q9H8H2	DDX31	1	8.617	12830000	0	2900700	100
Q8N1G0	ZNF687	1	7.2841	14391000	0	2697600	100
Q14257	RCN2	5	67.171	119010000	0	15136000	100
Q9BVJ6	UTP14A	1	6.0077	2384400	0	706230	100
Q9NY93	DDX56	4	56.272	58546000	0	14694000	100
P04792	HSPB1	1	10.437	7933800	0	7815800	100
Q86WX3	RPS19BP1	1	9.6468	7995000	0	2609100	100
Q9BRK5	SDF4	3	19.772	15285000	0	2733900	100
P84090	ERH	1	153.21	239830000	0	397200000	100
Q9GZT3	SLIRP	1	6.73	17330000	0	18177000	100
Q9NWN3	FBXO34	1	6.7031	3081600	0	912730	100
P36957	DLST	1	10.795	14226000	0	3570400	100
Q9GZR7	DDX24	7	39.102	47375000	0	11636000	100
P40926	MDH2	1	6.6727	2.622E+09	0	337480000	100
Q07157	TJP1	1	11.761	6501700	0	527380	100
H7C3G6	MPRIP	5	53.313	26900000	0	2010400	100
Q5SW79	CEP170	1	13.976	4057900	0	1201900	100
P12268	IMPDH2	3	24.171	13981000	0	3508800	100
Q7Z6Z7	HUWE1	1	6.0004	4178500	0	338940	100
O43852	CALU	1	6.5634	2394800	0	259990	100
P49736	MCM2	1	6.0224	2904900	0	235630	100
O94964	SOGA1	6	36.135	57883000	0	4695200	100
P46779	RPL28	6	43.887	54994000	0	20039000	100
Q9BZF9	UACA	3	20.927	26917000	0	2183400	100
O00161	SNAP23	2	12.227	15378000	0	15149000	100
Q8N1F7	NUP93	3	30.091	18384000	0	5445100	100
Q13421	MSLN	3	22.329	46432000	0	7153700	100
Q15287	RNPS1	1	7.3425	13614000	0	3416800	100
P12830	CDH1	1	8.7133	11170000	0	906090	100
P22695	UQCRC2	1	6.3981	2619100	0	284350	100
P04075	ALDOA	3	23.217	39462000	0	21153000	100
P07741	APRT	1	6.2578	4627500	0	1307200	100
Q7L2E3	DHX30	7	109.51	117720000	0	9267400	100
Q9Y5Q8	GTF3C5	1	6.8034	990340	0	248550	100
P17096	HMGA1	1	9.603	3506600	0	1437100	100
P51991	HNRNPA3	1	6.4648	5553200	0	2976700	100
Q99460	PSMD1	1	6.5962	2234300	0	661770	100
Q14156	EFR3A	1	6.3115	9765200	0	2892400	100
Q9HC36	MRM3	1	7.8749	41270000	0	10358000	100
Q02978	SLC25A11	1	10.093	84636000	0	34884000	100

Q9UPN4	CEP131	1	5.9413	1979900	0	160600	100
Q9UJV9	DDX41	2	35.97	40257000	0	7902600	100
P27448	MARK3	3	21.75	13372000	0	3960700	100
Q9Y4F5	CEP170B	6	61.182	78418000	0	6360900	100
Q9Y2R9	MRPS7	7	44.147	75320000	0	64183000	100
P51648	ALDH3A2	2	14.278	24188000	0	6070700	100
O00231	PSMD11	1	6.2305	2417200	0	262420	100
Q15029	EFTUD2	6	44.231	62801000	0	5094100	100
P63010	AP2B1	5	35.872	46948000	0	13906000	100
P26599	PTBP1	1	8.1786	7205100	0	1808300	100
Q9BYD3	MRPL4	1	14.793	15173000	0	5734500	100
O60437	PPL	2	17.291	4789000	0	388460	100
P35268	RPL22	2	11.118	37821000	0	15500000	100
P62841	RPS15	2	15.097	29787000	0	11489000	100
O14579	COPE	1	6.8772	7220700	0	2365600	100
Q86YD1	PTOV1	2	11.35	6919100	0	1844500	100
O43823	AKAP8	2	11.267	9918800	0	2937800	100
P46782	RPS5	6	54.8	151900000	0	141890000	100
Q9NQT4	EXOSC5	1	6.1767	3162200	0	3115200	100
Q02543	RPL18A	2	11.333	6269500	0	2569400	100
O00139	KIF2A	10	132.38	94776000	0	27227000	100
O00257	CBX4	2	16.368	15354000	0	4406500	100
O00411	POLRMT	5	67.228	54954000	0	3862800	100
O00425	IGF2BP3	4	50.268	37392000	0	8493900	100
O14556	GAPDHS	1	6.8782	7.257E+09	0	840980000	100
O15226	NKRF	1	8.2834	3398000	0	1006400	100
O15269	SPTLC1	3	19.304	18731000	0	4701200	100
O15446	POLR1G	1	5.9407	5951500	0	1762800	100
O43172	PRPF4	12	121.56	304030000	0	76305000	100
O43290	SART1	1	7.1203	3395100	0	1005600	100
O43293	DAPK3	1	9.5016	11422000	0	1956600	100
O43390	HNRNPR	2	11.068	52254000	0	14343000	100
O43734	TRAF3IP2	2	15.768	19031000	0	4776300	100
O60292	SIPA1L3	2	19.893	28350000	0	2299600	100
O60506	SYNCRIP	2	21.878	79627000	0	17040000	100
O60563	CCNT1	1	23.514	13561000	0	4016500	100
O60701	UGDH	1	11.834	5708600	0	1432700	100
O60884	DNAJA2	2	15.63	15311000	0	1379700	100
O75083	WDR1	2	12.267	6363600	0	1597100	100
O75319	DUSP11	3	22.701	23238000	0	10491000	100
O75330	HMMR	3	63.248	34649000	0	7269300	100
O75400	PRPF40A	4	36.311	55811000	0	3976800	100
O75486	SUPT3H	1	11.874	3034000	0	1626300	100
O75494	SRSF10	1	20.534	9956700	0	6736200	100
O75607	NPM3	1	7.9035	20608000	0	19551000	100
O75643	SNRNP200	6	64.55	58721000	0	4763100	100
O75683	SURF6	1	6.5082	5995000	0	650850	100
O75821	EIF3G	2	11.298	22573000	0	2450600	100
O75909	CCNK	2	21.857	25135000	0	7444600	100
O75934	BCAS2	3	18.961	24547000	0	24182000	100
O76031	CLPX	1	5.9838	12999000	0	3850200	100
O94761	RECQL4	1	10.659	8407700	0	681990	100
O94906	PRPF6	2	12.84	22089000	0	6542500	100
O95478	NSA2	1	6.5131	9554000	0	5121300	100
O95810	SDPR	2	19.404	12471000	0	3130100	100
O95831	AIFM1	2	11.113	16395000	0	4114800	100

P00367	GLUD1	5	45.466	94073000	0	21524000	100
P00966	ASS1	1	34.27	243170000	0	30059000	100
P01876	IGHA1	3	18.016	24663000	0	6190000	100
P02792	FTL	1	9.9961	11126000	0	4559600	100
P04083	ANXA1	4	25.553	24325000	0	13039000	100
P04350	TUBB4A	1	8.2718	3040600	0	763130	100
P04843	RPN1	5	38.004	54889000	0	12825000	100
P04844	RPN2	1	28.405	5973200	0	1499100	100
P04899	GNAI2	2	15.386	21542000	0	10009000	100
P05023	ATP1A1	4	33.198	40005000	0	7027000	100
P05198	EIF2S1	1	7.8441	19672000	0	10545000	100
P05386	RPLP1	2	40.393	158940000	0	43922000	100
P06858	LPL	1	12.154	4236800	0	459970	100
P07195	LDHB	3	16.747	23850000	0	11006000	100
P07948	LYN	6	53.049	98893000	0	20199000	100
P08651	NFIC	4	71.083	33330000	0	7965800	100
Q99878	HIST1H2AJ	2	83.135	105260000	0	40066000	100
P10696	ALPPL2	1	12.402	7417500	0	1861600	100
P12814	ACTN1	9	64.722	74118000	0	16977000	100
P13639	EEF2	3	18.44	7886700	0	2335900	100
P13674	P4HA1	2	11.426	16298000	0	4090500	100
P14618	PKM	7	61.894	105320000	0	26433000	100
P14625	HSP90B1	2	25.886	43384000	0	9426900	100
P14649	MYL6B	5	37.962	58358000	0	57490000	100
P16615	ATP2A2	5	49.812	75001000	0	18340000	100
P48741	HSPA7	1	6.8542	49342000	0	2949400	100
P17302	GJA1	1	19.31	25786000	0	2799400	100
P17987	TCP1	3	19.412	45634000	0	9673100	100
P20700	LMNB1	2	17.858	22629000	0	6333600	100
P22626	HNRNPA2B	3	17.272	40910000	0	21929000	100
P25205	MCM3	2	13.076	17726000	0	5250300	100
P26232	CTNNA2	1	13.127	53768000	0	10754000	100
P26641	EEF1G	5	42.849	116710000	0	22783000	100
P28066	PSMA5	1	15.549	6888400	0	5017500	100
P30414	NKTR	1	6.2669	285940000	0	31043000	100
P31689	DNAJA1	3	27.165	50069000	0	4637500	100
P31944	CASP14	2	11.052	8836400	0	5447100	100
P35232	PHB	3	19.053	10791000	0	10631000	100
P35250	RFC2	3	18.638	54271000	0	19422000	100
P35251	RFC1	6	48.559	66018000	0	5532600	100
P35658	NUP214	2	13.204	14430000	0	1170500	100
P35749	MYH11	2	19.432	37803000	0	298970	100
P40938	RFC3	1	152.17	21635000	0	6097900	100
P40939	HADHA	12	177.75	187530000	0	53177000	100
P42166	TMPO	10	72.139	93384000	0	27292000	100
P46013	MKI67	31	323.31	550150000	0	49180000	100
P46778	RPL21	2	15.853	25605000	0	15271000	100
P47929	LGALS7	1	6.7329	8689500	0	5353400	100
P48047	ATP5O	2	13.011	56709000	0	54085000	100
P49750	YLPM1	6	63.982	82754000	0	6016000	100
P49756	RBM25	2	11.547	9289400	0	2215700	100
P49792	RANBP2	5	38.18	41933000	0	3401400	100
P50402	EMD	2	22.789	37060000	0	15842000	100
P50416	CPT1A	1	11.555	18312000	0	5424000	100
P50750	CDK9	2	30.953	47925000	0	13180000	100
P50990	CCT8	4	25.767	47778000	0	9686100	100

P51116	FXR2	6	71.431	76739000	0	21005000	100
P51398	DAP3	5	35.855	82481000	0	35640000	100
P53597	SUCLG1	1	16.073	281700000	0	36778000	100
P53618	COPB1	1	6.0304	7232800	0	2142300	100
P53621	COPA	2	12.875	39388000	0	3194900	100
P53985	SLC16A1	1	6.94	7630200	0	828380	100
P53999	SUB1	3	21.9	75369000	0	30888000	100
P54793	ARSF	1	6.3765	3154200	0	139530	100
P55084	HADHB	3	21.73	66394000	0	6438200	100
P55884	EIF3B	3	18.97	19217000	0	5691800	100
P60174	TPI1	1	6.2932	9576300	0	7555800	100
P61160	ACTR2	5	30.236	68151000	0	6629800	100
P61254	RPL26	5	28.768	49613000	0	14384000	100
P61313	RPL15	4	22.839	98775000	0	97306000	100
P61626	LYZ	2	13.839	75956000	0	11000000	100
P61978	HNRNPK	9	165.43	215710000	0	49601000	100
P61981	YWHAG	2	13.678	19381000	0	11825000	100
P62081	RPS7	2	19.8	13341000	0	13142000	100
P62136	PPP1CA	1	7.2013	15553000	0	8337200	100
P62140	PPP1CB	3	39.158	104780000	0	49759000	100
P62273	RPS29	1	6.9579	6693500	0	5139200	100
P62333	PSMC6	1	6.1311	6556500	0	711810	100
P62805	HIST1H4A	5	28.992	38426000	0	19887000	100
P62851	RPS25	3	63.545	207380000	0	73260000	100
P62857	RPS28	4	32.389	121340000	0	86031000	100
P62891	RPL39	2	18.568	10380000	0	17234000	100
P62899	RPL31	5	63.628	189460000	0	76821000	100
P62995	TRA2B	3	94.089	61089000	0	32746000	100
P63092	GNAS	3	18.087	51398000	0	4737900	100
P63220	RPS21	3	16.928	17506000	0	16688000	100
P78316	NOP14	2	12.297	12762000	0	3779900	100
Q01650	SLC7A5	1	14.144	46343000	0	20925000	100
Q02880	TOP2B	10	104.93	180560000	0	12997000	100
Q03111	MLLT1	6	114.26	160970000	0	46570000	100
Q03701	CEBPZ	2	41.093	21451000	0	1740000	100
Q06210	GFPT1	1	11.35	5619100	0	1664300	100
Q06587	RING1	4	69.713	110250000	0	35068000	100
Q06830	PRDX1	3	18.321	29563000	0	25602000	100
Q07021	C1QBP	3	17.997	27981000	0	14999000	100
Q07666	KHDRBS1	2	15.803	17359000	0	3282500	100
Q08380	LGALS3BP	2	11.005	4973200	0	1473000	100
Q08945	SSRP1	3	29.088	63930000	0	17546000	100
Q12860	CNTN1	2	11.677	4168500	0	338120	100
Q12962	TAF10	1	8.0312	8015900	0	7896600	100
Q13155	AIMP2	1	10.598	7724000	0	4140400	100
Q13200	PSMD2	4	29.077	29795000	0	8825100	100
Q13243	SRSF5	3	23.51	122880000	0	56658000	100
Q13247	SRSF6	5	130.77	90518000	0	13995000	100
Q13263	TRIM28	5	104	87560000	0	25934000	100
Q13509	TUBB3	1	24.095	25009000	0	4072200	100
Q13601	KRR1	1	6.2017	14057000	0	2451600	100
Q13751	LAMB3	5	48.316	52591000	0	4999600	100
Q13823	GNL2	3	26.7	25732000	0	7621500	100
Q14134	TRIM29	3	16.972	20532000	0	5153200	100
Q14142	TRIM14	2	12.427	16889000	0	1833600	100
Q14204	DYNC1H1	6	67.423	38656000	0	1989500	100

Q14210	LY6D	2	24.118	50354000	0	17246000	100
Q14566	MCM6	2	11.694	11344000	0	3360000	100
Q14651	PLS1	2	20.208	19264000	0	3465400	100
Q14669	TRIP12	6	58.248	111020000	0	8633200	100
Q14676	MDC1	2	14.43	10724000	0	869880	100
Q14690	PDCD11	8	124.74	107390000	0	7226100	100
Q14966	ZNF638	3	20.166	20162000	0	2898400	100
Q14974	KPNB1	7	49.887	478060000	0	137660000	100
Q15007	WTAP	2	18.184	39064000	0	9398100	100
Q15046	KARS	1	7.8177	4464700	0	1322400	100
Q15050	RRS1	3	28.145	21319000	0	2314500	100
Q15056	EIF4H	1	18.375	3804400	0	3747800	100
Q15365	PCBP1	1	6.0388	6147800	0	3295500	100
Q15393	SF3B3	3	29.257	50943000	0	4132200	100
Q15398	DLGAP5	3	16.781	10976000	0	3251000	100
Q15427	SF3B4	1	6.9859	13762000	0	911850	100
Q16891	IMMT	11	82.894	103680000	0	29084000	100
Q1ED39	KNOP1	2	20.703	39123000	0	6945100	100
Q27J81	INF2	7	86.743	101280000	0	6276100	100
Q4G0S7	CCDC152	1	6.1732	2768500	0	694830	100
Q4KMQ1	TPRN	2	22.13	8831400	0	2615800	100
Q4VXZ8	DECR2	1	6.2322	1762700	0	1736500	100
Q53G59	KLHL12	5	34.73	69361000	0	15678000	100
Q562R1	ACTBL2	2	14.257	35909000	0	2646900	100
Q58FF8	HSP90AB2P	1	7.0414	4890200	0	1448400	100
Q5BKZ1	ZNF326	1	6.0522	19902000	0	4007400	100
Q5EBL8	PDZD11	1	11.471	16676000	0	6834300	100
Q5JR04	MOV10	2	11.962	12041000	0	3566400	100
Q5RKV6	EXOSC6	1	11.837	11524000	0	4067800	100
Q5SSJ5	HP1BP3	4	36.315	90169000	0	20683000	100
Q5T0W9	FAM83B	3	28.93	29519000	0	8457900	100
Q5VY93	ARHGEF2	2	11.946	6874700	0	1612200	100
Q68D10	SPTY2D1	1	6.6001	13931000	0	3839000	100
Q69YN4	VIRMA	7	45.963	59488000	0	4825400	100
Q69YQ0	SPECC1L	7	66.432	111330000	0	9367000	100
Q6P2Q9	PRPF8	7	59.123	74281000	0	5101600	100
Q6PKG0	LARP1	5	37.548	52123000	0	4227900	100
Q6SPF0	SAMD1	7	65.54	227360000	0	66354000	100
Q96J85	LARP4	4	34.768	50253000	0	13126000	100
Q7Z2T5	TRMT1L	2	18.926	18715000	0	4310800	100
Q7Z2W4	ZC3HAV1	2	17.147	26846000	0	4924600	100
Q7Z417	NUFIP2	2	10.813	3536400	0	1047500	100
Q7Z478	DHX29	1	6.0609	3213100	0	260630	100
Q7Z6E9	RBBP6	1	5.9623	3864200	0	313440	100
Q7Z7K6	CENPV	1	26.474	44795000	0	21462000	100
Q86VH2	KIF27	1	11.334	57862000	0	7765800	100
Q86XZ4	SPATS2	5	68.278	136500000	0	38363000	100
Q8IY81	FTSJ3	2	12.418	20980000	0	1701800	100
Q8N0Z8	PUSL1	1	8.7524	4760900	0	2552100	100
Q8N9B5	JMY	1	9.8219	12239000	0	3458700	100
Q8NDT2	RBM15B	3	26.693	27359000	0	8103400	100
Q8NDX5	PHC3	3	43.589	44856000	0	8603600	100
Q8NI77	KIF18A	1	16.691	15974000	0	4731200	100
Q8TA86	RP9	1	6.5448	6288100	0	3370700	100
Q8WWM7	ATXN2L	2	12.382	12592000	0	1021400	100
Q8WXF1	PSPC1	1	10.482	7470300	0	1874900	100

Q8WXX5	DNAJC9	2	10.877	32301000	0	13014000	100
Q8WY91	THAP4	1	9.1204	4101800	0	1214900	100
Q92499	DDX1	3	17.724	19218000	0	4468600	100
Q92616	GCN1L1	2	14.129	4929000	0	399810	100
Q92828	CORO2A	4	34.255	108790000	0	21365000	100
Q92979	EMG1	1	6.497	23747000	0	19141000	100
Q969L2	MAL2	1	22.141	21631000	0	7829100	100
Q96A35	MRPL24	1	6.0046	4182000	0	4119800	100
Q96GD4	AURKB	5	44.713	139730000	0	72355000	100
Q96GQ7	DDX27	4	22.286	36917000	0	10934000	100
Q96GY0	ZC2HC1A	3	28.393	25562000	0	12277000	100
Q9Y383	LUC7L2	1	8.557	8852800	0	4745500	100
Q96JM7	L3MBTL3	5	33.933	52127000	0	15439000	100
Q96PH1	NOX5	1	6.464	231050000	0	24193000	100
Q96QR8	PURB	6	36.551	148350000	0	79522000	100
Q96T37	RBM15	7	63.951	138370000	0	40984000	100
Q96T51	RUFY1	3	18.442	27362000	0	7904700	100
Q96TA2	YME1L1	1	6.3503	7377100	0	1851500	100
Q99459	CDC5L	4	24.684	22922000	0	6789200	100
Q99496	RNF2	4	35.813	76368000	0	38359000	100
Q99575	POP1	1	6.1404	5924900	0	480600	100
Q99832	CCT7	3	18.972	22817000	0	5726500	100
Q99959	PKP2	1	7.4748	8669800	0	2567900	100
Q9BPX5	ARPC5L	2	12.736	21567000	0	8838600	100
Q9BQ67	GRWD1	3	16.88	19233000	0	4827100	100
Q9BU76	MMTAG2	1	6.3448	14283000	0	7656400	100
F5H303	NOC4L	3	94.781	45659000	0	10192000	100
Q9BYX7	POTEKP	1	14.667	40332000	0	2693100	100
Q9GZR2	REXO4	1	8.4283	7741900	0	1943100	100
Q9H4H8	FAM83D	5	39.435	56561000	0	12444000	100
Q9H4P4	RNF41	1	6.5163	12161000	0	6519000	100
Q9H7Z6	KAT8	2	12.93	27659000	0	6941900	100
Q9HAU0	PLEKHA5	3	17.72	18388000	0	4599100	100
Q9HC52	CBX8	4	50.98	43753000	0	4750100	100
Q9NQT5	EXOSC3	1	11.611	23116000	0	8185200	100
Q9NQX4	MYO5C	4	45.121	64418000	0	3967400	100
Q9NR48	ASH1L	4	24.363	30039000	0	15694000	100
Q9NSI2	FAM207A	1	5.9472	9541700	0	9399700	100
Q9NUQ6	SPATS2L	6	47.344	78595000	0	20070000	100
Q9NW13	RBM28	9	55.423	79828000	0	22223000	100
Q9NZB2	FAM120A	1	9.782	10338000	0	838570	100
Q9NZM5	NOP53	4	34.073	59440000	0	12634000	100
Q9P015	MRPL15	2	13.076	9068000	0	4860800	100
Q9P0L2	MARK1	1	9.5168	5947400	0	1761600	100
Q9UBU9	NXF1	15	170.27	404060000	0	111050000	100
Q9UDY2	TJP2	7	56.216	52345000	0	3688000	100
Q9UHB7	AFF4	12	136.31	208980000	0	37557000	100
Q9UJW0	DCTN4	1	6.8032	10480000	0	2630200	100
Q9UK80	USP21	2	13.464	11641000	0	2921600	100
Q9UKS6	PACSIN3	3	23.123	32446000	0	6671100	100
Q9UKX7	NUP50	2	15.533	9940000	0	2494700	100
Q9UM54	MYO6	1	14.676	23798000	0	1930300	100
Q9UMN6	KMT2B	1	6.3134	3192500	0	945590	100
Q9UMS4	PRPF19	4	37.949	52487000	0	11999000	100
Q9UN86	G3BP2	1	10.805	9335100	0	2342900	100
Q9UQ35	SRRM2	32	323.31	1.094E+09	0	114630000	100

Q9Y230	RUVBL2	4	22.582	45917000	0	11524000	100
Q9Y2W1	THRAP3	4	23.368	22929000	0	2707000	100
Q9Y2X3	NOP58	6	58.946	33954000	0	7781100	100
Q9Y3A4	RRP7A	2	11.551	16507000	0	8848700	100
Q9Y3B9	RRP15	2	11.879	14964000	0	7005800	100
Q9Y3I0	RTCB	1	7.1517	6288700	0	1578300	100
Q9Y3T9	NOC2L	2	14.214	8465200	0	2507300	100
Q9Y3U8	RPL36	2	10.792	38533000	0	15792000	100
Q9Y446	PKP3	4	35.626	60049000	0	15974000	100
Q9Y4B5	MTCL1	2	12.299	14621000	0	1186000	100
Q9Y6J9	TAF6L	4	30.232	24860000	0	6530600	100
Q9Y6N5	SQRDL	2	20.914	23784000	0	2582100	100
Q00577	PURA	7	215.91	1.867E+09	6585600	239480000	36.36418853
Q9H6R0	DHX33	13	231.53	360190000	6465500	102470000	15.8487356
Q9NZT1	CALML5	6	101.44	576580000	33756000	404710000	11.98927598
Q9H6W3	RIOX1	7	68.56	142410000	4806600	38671000	8.045395914
P19013	KRT4	6	78.975	161490000	12590000	65496000	5.202223987
Q9NUL7	DDX28	11	173.35	252210000	12147000	57236000	4.711945336
Q5T3I0	GPATCH4	5	57.997	90929000	4582600	21457000	4.682276437
P62266	RPS23	5	29.56	116590000	11316000	51206000	4.525097207
P11940	PABPC1	13	284.93	933970000	58985000	219920000	3.728405527
P40429	RPL13A	6	88.062	405780000	91651000	330630000	3.60748928
P52292	KPNA2	6	46.339	107280000	6374400	22856000	3.585592369
Q16629	SRSF7	4	41.355	140950000	20761000	68829000	3.315302731
P42766	RPL35	5	41.953	114150000	12111000	38240000	3.15746016
Q96HP0	DOCK6	7	69.634	110070000	2372100	7187000	3.029804814
Q14980	NUMA1	57	323.31	3.724E+09	85515000	255350000	2.986025843
Q6YHK3	CD109	10	118.51	216530000	13457000	39847000	2.961061158
Q13310	PABPC4	8	70.062	248090000	21059000	58016000	2.754926635
Q9BXS6	NUSAP1	13	127.12	395550000	33647000	90629000	2.69352394
Q07955	SRSF1	6	57.822	179390000	29599000	73359000	2.478428325
P35222	CTNNB1	7	165.79	158200000	14464000	35496000	2.45409292
Q86YZ3	HRNR	9	156.94	454800000	89622000	213660000	2.384012854
P56539	CAV1	2	92.588	168420000	53625000	118350000	2.206993007
P62854	RPS26	2	14.499	25491000	4084600	8777000	2.14880282
O00567	NOP56	11	189.31	342030000	36329000	77016000	2.119959261
E9PPU1	RPS3	8	75.524	622500000	2.03E+08	419960000	2.070094149
P46781	RPS9	6	39.22	253460000	87774000	181050000	2.06268371
P38646	HSPA9	12	104.09	587430000	61009000	125210000	2.052320149
O43143	DHX15	9	81.622	133900000	13780000	28122000	2.040783745
P62861	FAU	2	11.269	39652000	17912000	35949000	2.006978562
P35221	CTNNA1	17	323.31	509290000	56743000	112660000	1.985443138
P62906	RPL10A	6	323.31	259550000	96949000	191430000	1.974543317
P46940	IQGAP1	6	72.271	86484000	3143600	6102200	1.941150274
Q9Y2R4	DDX52	7	80.503	117100000	11369000	22058000	1.940188231
P26373	RPL13	11	289.33	767290000	2.84E+08	536590000	1.892533418
Q5JR95	RPS8	7	255.95	240190000	1.04E+08	189860000	1.826279338
Q7KZI7	MARK2	5	60.641	56640000	6486900	11771000	1.814580154
